# Analysis of the *Mycoplasma genitalium* MgpB Adhesin to Predict Membrane Topology, Investigate Antibody Accessibility, Characterize Amino Acid Diversity, and Identify Functional and Immunogenic Epitopes

**DOI:** 10.1371/journal.pone.0138244

**Published:** 2015-09-18

**Authors:** Stefanie L. Iverson-Cabral, Gwendolyn E. Wood, Patricia A. Totten

**Affiliations:** 1 Department of Medicine, Division of Infectious Diseases, University of Washington, Seattle, WA, United States of America; 2 Department of Global Health, Pathobiology Interdisciplinary Program, University of Washington, Seattle, WA, United States of America; Miami University, UNITED STATES

## Abstract

*Mycoplasma genitalium* is a sexually transmitted pathogen and is associated with reproductive tract disease that can be chronic in nature despite the induction of a strong antibody response. Persistent infection exacerbates the likelihood of transmission, increases the risk of ascension to the upper tract, and suggests that *M*. *genitalium* may possess immune evasion mechanism(s). Antibodies from infected patients predominantly target the MgpB adhesin, which is encoded by a gene that recombines with homologous donor sequences, thereby generating sequence variation within and among strains. We have previously characterized *mgpB* heterogeneity over the course of persistent infection and have correlated the induction of variant-specific antibodies with the loss of that particular variant from the infected host. In the current study, we examined the membrane topology, antibody accessibility, distribution of amino acid diversity, and the location of functional and antigenic epitopes within the MgpB adhesin. Our results indicate that MgpB contains a single transmembrane domain, that the majority of the protein is surface exposed and antibody accessible, and that the attachment domain is located within the extracellular C-terminus. Not unexpectedly, amino acid diversity was concentrated within and around the three previously defined variable regions (B, EF, and G) of MgpB; while nonsynonymous mutations were twice as frequent as synonymous mutations in regions B and G, region EF had equal numbers of nonsynonymous and synonymous mutations. Interestingly, antibodies produced during persistent infection reacted predominantly with the conserved C-terminus and variable region B. In contrast, infection-induced antibodies reacted poorly with the N-terminus, variable regions EF and G, and intervening conserved regions despite the presence of predicted B cell epitopes. Overall, this study provides an important foundation to define how different segments of the MgpB adhesin contribute to functionality, variability, and immunogenicity during persistent *M*. *genitalium* infection.

## Introduction

*Mycoplasma genitalium* is a sexually transmitted pathogen associated with urethritis in men and urethritis, cervicitis, endometritis, pelvic inflammatory disease, tubal factor infertility, and pre-term birth in women (reviewed in [1–4]). Infections with *M*. *genitalium* can persist for months or even years when untreated or ineffectively treated [5–9], suggesting that the bacterium can evade the host immune response. The clinical importance of chronic infection is emphasized by an association with HIV transmission and acquisition [10] and the ineffectiveness of current treatment regimens [11–13]. Along with the potentially serious sequelae that may result from *M*. *genitalium*-associated reproductive tract disease, these findings underscore the need to improve our understanding of the molecular mechanisms involved in pathogenesis and persistence of this unique bacterial species.

*M*. *genitalium* cells exhibit several key features that distinguish them from other pathogens. For example, this fastidious bacterium contains a remarkably small genome (580kb) [14], lacks a cell wall, and displays a flask-shaped morphology that includes a tip structure known as the terminal organelle. This complex structure mediates motility and cell division [15] and is comprised of a unique set of proteins that includes the primary adhesin MgpB (also designated MgPa or P140) [15–18] and the cytadherence-associated MgpC (also known as P110) [15]. Importantly, MgpB and MgpC independently accumulate sequence diversity through recombination with homologous donor sequences (as described below) [19–24] and elicit humoral and cervicovaginal antibodies during natural human [25–28] and experimental animal [16, 29–32] infection.

We [20, 21] and others [19, 23] have hypothesized that *M*. *genitalium* evades antibody-mediated killing and other host immune defenses, in part, through antigenic variation of MgpB and MgpC. This antigenic variation is mediated by reciprocal recombination between the *mgpB* and *mgpC* expression sites and silent homologous donor sequences known as MgPar regions distributed throughout the chromosome [20, 21]. While conserved segments include sequences unique to the expression site, specific regions of *mgpB* (designated B, EF, and G) and *mgpC* (KL and LM) have homology, but not identity, to the MgPar sites allowing for homologous recombination. Indeed, using *in vitro* propagated strains, clinical samples, and an experimentally-infected non-human primate model, our group and others have demonstrated that a diverse repertoire of *mgpB* and *mgpC* variants are expressed within and among strains [20–24, 33].

Our current study focuses on characteristics of the primary adhesin MgpB. Nucleotide heterogeneity in *mgpB* has been extensively documented in previous studies [20, 21, 23, 32, 33], yet the resulting amino acid diversity has not been defined, nor has the relationship between amino acid diversity, immunogenicity, and membrane topology. In addition, studies aimed at identifying the attachment domain have yielded conflicting results [34, 35]. In an effort to characterize the structural and functional features of MgpB, we conducted a comprehensive study investigating membrane topology, antibody accessibility, location of functional epitopes, concentration of antigenic diversity, and immunogenicity of this protein. Our findings suggest a topological model in which the distal ~70 amino acids at the C-terminus are cytoplasmic, orienting the remainder of the protein on the cell surface where it is subject to antibody binding and immune pressure. Although a comparison of all available *mgpB* sequence variants reveals that amino acid diversity accumulates around the three previously defined variable regions, antibodies induced during *M*. *genitalium* infection were directed against variable region B and the extracellular portion of the conserved C-terminus, the latter of which was shown to contain attachment domains. Paradoxically, we found that variable regions EF and G were not recognized by immune serum despite a high degree of amino acid heterogeneity and the presence of predicted B cell epitopes. In addition, we detected other unique features within MgpB including homology to MgpC, periodic amino acid repeats, and an overabundance of certain amino acids, all of which may guide future studies regarding protein function. Altogether, the identification of regions that are surface exposed, functional, diverse, and immunogenic reported in this study provides an important foundation to understand how MgpB enhances *M*. *genitalium* pathogenicity.

## Materials & Methods

### *In silico* Analyses

The primary amino acid sequence of MgpB expressed by G37, the *M*. *genitalium* type strain (Genbank Accession #WP_010869366.1), was analyzed using various *in silico* algorithms in four independent studies to locate the putative signal sequence and transmembrane domains, identify regions of similarity to other Mycoplasma proteins, and characterize usual patterns of amino acids. First, to evaluate the location of a predicted signal peptide sequence, PrediSi (http://www.predisi.de/home.html) and various iterations of SignalP (http://www.cbs.dtu.dk/services/SignalP/) were utilized with the eukaryotic, Gram-negative, and Gram-positive predictors. Second, eight different algorithms were used to identify transmembrane domains, which were then compared to previous predictions made using the hydrophobic moment plot method of Eisenberg [36] (S1 Table); these algorithms included: TMpred (http://www.ch.embnet.org/software/TMPRED_form.html), HMMTOP (http://www.enzim.hu/hmmtop/), SCAMPI (http://scampi.cbr.su.se/), TOPCONS (http://topcons.cbr.su.se/), TopPred 1.10 (http://mobyle.pasteur.fr/cgi-bin/portal.py?#forms::toppred), DAS (http://mendel.imp.ac.at/sat/DAS/DAS.html), SOSUI (http://harrier.nagahama-i-bio.ac.jp/sosui/), and TMHMM 2.0 (http://www.cbs.dtu.dk/services/TMHMM-2.0/). Third, BLAST (http://blast.ncbi.nlm.nih.gov/) was used to identify significant regions of homology between portions of MgpB and other proteins of *M*. *genitalium* (including MgpC, Genbank Accession #AAC71411) or *Mycoplasma pneumoniae* (including the P1 and ORF6 sequences, Genbank Accession #M21519.1); alignments between regions of homology were generated with Clustal Omega (http://www.ebi.ac.uk/Tools/msa/clustalo/). Finally, in the fourth study, the Statistical Analysis of Protein (SAPS) program (http://www.ebi.ac.uk/Tools/seqstats/saps/) was employed to evaluate unusual sequence properties within MgpB including compositional biases, unique clusters and repeats of amino acids, and abnormal spacing between identical residues, using the default settings [37]. In this analysis, the full-length MgpB sequence as well as eleven overlapping fragments of approximately 240 amino acids each was compared to a random sample of proteins from the Swiss-Prot database or *Bacillus subtilis* and *Escherichia coli* protein reference sets.

### Cloning, Expression, and Purification of Recombinant MgpB Peptides

**Fig 1:**
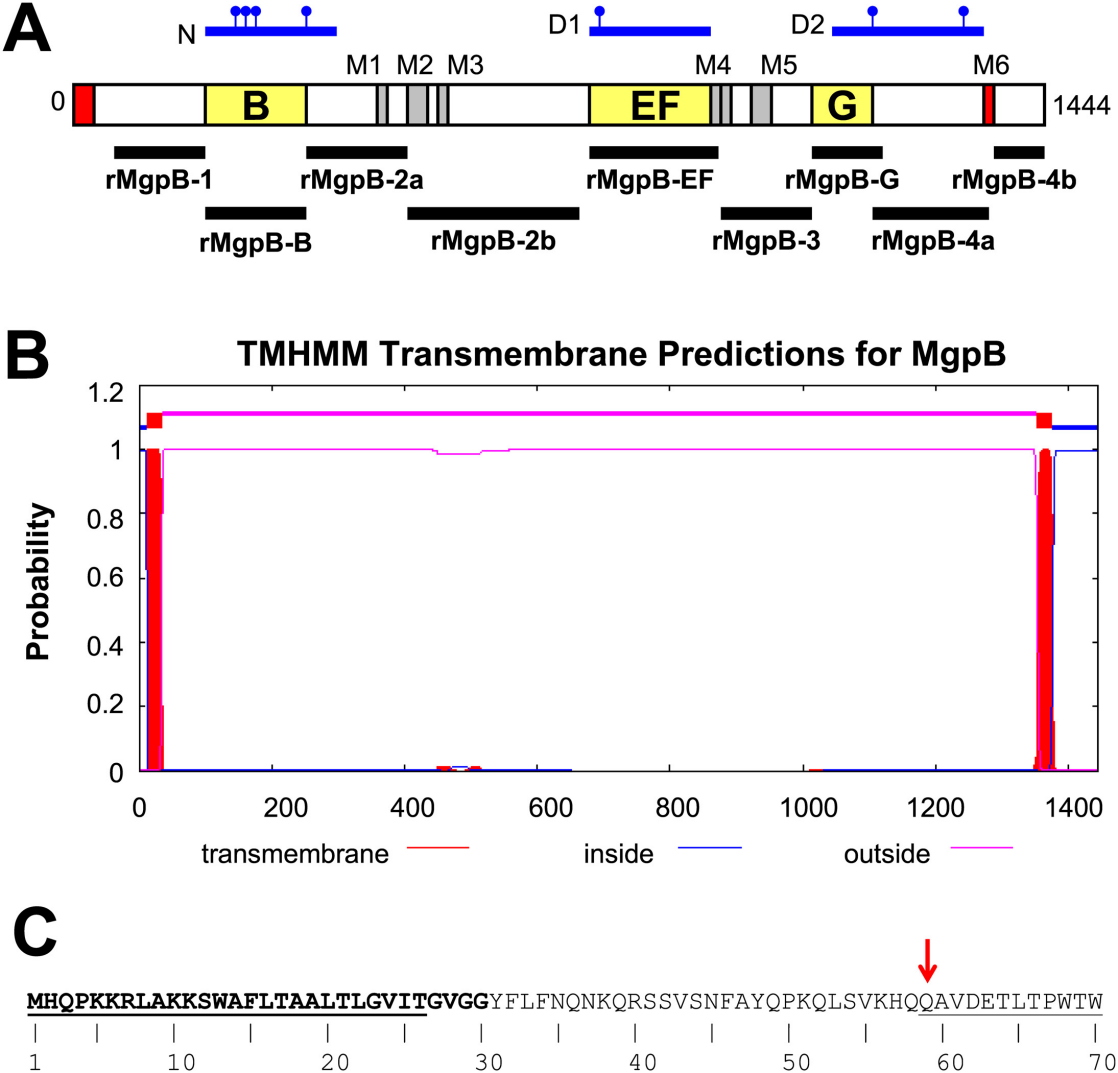
MgpB Contains a Single C-terminal Transmembrane Domain. (A) Schematic depiction of MgpB showing the location of the N-terminal segment absent in the mature protein (aa 1–58, red), variable regions B, EF, and G (yellow), and the position of the previously described transmembrane domains M1–M5 [39] (gray and red, see S1 Table for coordinates). The recombinant peptides spanning MgpB used for the production of rabbit antibodies are indicated with black bars. The N-, D1-, and D2-domains described by Opitz and Jacobs [34] spanning aa 200–384, 769–954, and 1,123–1,360, respectively, are shown in blue with the precise binding locations of attachment inhibiting monoclonal antibodies [34] indicated by lines with circles. (B) Transmembrane domains in MgpB predicted by the TMHMM program. The N-terminal signal peptide and a single C-terminal transmembrane domain (both red) are identified with high probability, predicting a topology in which the majority of the protein is extracellular (pink) with a short cytoplasmic domain (blue), a prediction corroborated by the majority of algorithms used. (C) The N-terminal transmembrane helix identified in panel B includes the predicted MgpB signal peptide. Sequencing of mature MgpB [39] indicates that the N-terminus begins at amino acid 59 (solid underline following red arrow). Using the Gram-positive network, all computational programs (see Materials & Methods) identify a signal peptide spanning amino acids 1–30 (bold) with the predicted cleavage site occurring between the amino acids VGG-YF; results using the Gram-negative network give a shorter signal peptide covering amino acids 1–26 with the cleavage site between VIT-GV (dotted underline).

Recombinant His-tagged peptides spanning conserved and variable regions of MgpB (S1A Fig and Fig 1A) were generated using the pET-30 Ek/LIC system (EMD Biosciences, Darmstadt, Germany) as previously described [32]. For the recombinant rMgpB-1,-B,-EF, and-G constructs, genomic DNA from strain G37-C, a single-colony, filter-cloned derivative of the G37 type strain [21] was amplified using primers and conditions detailed in S2 Table and ligated into the pET-30 Ek/LIC vector. Because *M*. *genitalium* uses an alternative genetic code [38], we converted the TGA codons (encoding tryptophan in *M*. *genitalium*, but a translational stop in *E*. *coli*) to TGG to allow expression of full-length rMgpB-B and-EF proteins in *E*. *coli* (rMgpB-1 and-G lack in-frame TGAs). This was accomplished with the QuikChange® Multi Site-Directed Mutagenesis kit (Stratagene, La Jolla, CA) and primers rMgpB-B TGA_321_: 5’-AGGAGTTTACTGAGGCCTGGAAACCATTGTTGACTACTG-3’, rMgpB-EF TGA_770_: 5’-CTTAGACTTTCTCCCCTGGATCGGCAATGGTAAACCC-3’, and rMgpB-EF TGA_829_: 5’-CTTTTCACCTGACATCTGGACAGGAGCAGGGTATCGC-3’ (A to G mutations are underlined). The remaining constructs (rMgpB-2a, -2b, -3, -4a, and -4b) were PCR amplified (S2 Table) from pBSK-*mgpB* ^*S*^, a pBluescript II SK (+) (Agilent, Santa Clara, CA) plasmid containing a synthetic *mgpB* gene optimized for expression in *E*. *coli* by altering the GC-content, codon preference, and mutation of TGAs to TGGs (Epoch Life Science, Missouri City, Texas; Genbank Accession #KP318804). Importantly, pBSK-*mgpB* ^*S*^ is predicted to express an MgpB protein that is 100% identical to wild-type even though the DNA sequences are only 75% identical. Amplicons were ligated into the pET-30 Ek/LIC vector and all plasmid constructs were verified by sequencing. His-tagged proteins were over-expressed in *E*. *coli* BL21 (DE3) (EMD Biosciences, Darmstadt, Germany) and purified using nickel chromatography under denaturing conditions as outlined previously [32]. After the elution buffer was exchanged for phosphate buffered saline (PBS) using a PD-10 column (GE Healthcare, Little Chalfont, United Kingdom), the recombinant peptides were quantitated using the BCA protein assay kit (Thermo Scientific, Waltham, MA) and used to immunize rabbits or in ELISAs, as described below.

### Production and Evaluation of Polyclonal Rabbit Anti-Sera against Recombinant MgpB Peptides

Rabbit antibodies were produced by Pacific Immunology (Ramona, CA) using 0.5–3.0 mg of the purified His-tagged recombinant proteins described above. Following their 13-week protocol, rabbits received a primary immunization with AdjuLite™ Complete Freund’s Adjuvant (CFA) and three subsequent boosts with AdjuLite™ Incomplete Freund’s Adjuvant (IFA). The specificity of the resulting antiserum for the intended antigen was confirmed by immunoblot analysis (S1B Fig) performed as described previously [32] using whole cell lysates (10 ug of total protein) of *M*. *genitalium* strains G37-C and Δmg191 (which lacks MgpB [15]). In these assays, primary antibodies were diluted 1:10,000 (α-rMgpB-1, -2a, -2b, and -3), 1:50,000 (α-rMgpB-EF,-G and -4b), 1:500,000 (α-rMgpB-B), or 1:1,000,000 (α-rMgpB-4a), and detected with a 1:10,000 dilution of peroxidase-conjugated goat anti-rabbit IgG (whole molecule; Sigma-Aldrich).

### Antibody Accessibility Assay

The surface exposure and antibody accessibility of the MgpB conserved and variable regions were determined using an antibody accessibility assay that measures the binding of antibodies to the surface of intact *M*. *genitalium* cells. For this experiment, *M*. *genitalium* strain G37-C was grown in SP-4 broth [40] in 75-cm^2^ tissue culture flasks. Adherent cells were washed once with 1x PBS, scraped into 5–10 ml of fresh SP-4 medium, passed twice through a 25-gauge needle to disrupt cell aggregates, and then seeded into 96-well plates (100 ul per well; Corning, Corning, NY). After overnight incubation at 37°C with 5% CO_2_ in a humidified chamber, the culture supernatant was discarded and adherent cells were washed twice with PBS and then fixed with 4% paraformaldehyde for 10 minutes at room temperature. Cells were permeabilized with 0.1% Triton X-100 in PBS for 5 minutes at room temperature; unpermeablized cells were treated with PBS alone. All wells were washed three times with PBS, blocked with 1% bovine serum albumin (BSA) in PBS for 15–30 minutes, and then incubated with 100 ul of the antibodies against the recombinant MgpB peptides described above after purification using the Nab Protein A/G antibody purification kit (Thermo Scientific, Rockford, IL) according to the manufacturer’s instructions. The purified antibodies were titered against whole-cell G37-C lysates by Western spot blots and antibody concentrations were adjusted by dilution in PBS (1:100 to 1:10,000) to normalize total reactivity between serum samples. Plates were incubated for one hour at room temperature, washed three times with PBS, and then incubated for 30 minutes at room temperature with goat anti-rabbit IgG conjugated to horseradish peroxidase (HRP; Sigma Aldrich, St. Louis, MO) diluted 1:1,000 in PBS. After three washes with PBS, HRP was detected with SureBlue^TM^ 1-Component TMB microwell peroxidase substrate (KPL, Gaithersburg, MD). Color development was stopped by adding 1N HCl after approximately 10 minutes at room temperature and the absorbance was determined using a microtiter plate reader (A_450 nm_). To account for background reactivity that varies between rabbits, pre-immune sera was purified and used at the same dilution as the corresponding immune sera; pre-immune reactivity was then subtracted from corresponding immune sera. Control wells confirmed negligible reactivity of primary antibodies to SP-4 media alone and of secondary antibodies to G37-C and SP-4. The significance for reactivity between intact and lysed bacteria was determined using student’s unpaired t test (http://www.graphpad.com/quickcalcs/ttest1.cfm).

### Quantitative Hemadsorption Inhibition Assay

We adapted a published quantitative hemadsorption inhibition assay [41] to determine the location of attachment domains within MgpB. *M*. *genitalium* strain G37-C was cultured in H-broth [42] in 75 cm^2^ flasks until color changed from red to orange/yellow (indicating late logarithmic growth), after which adherent cells from multiple flasks were scraped into the media, combined, and frozen at -80°C in 4 ml aliquots. These pooled frozen stocks served as the inoculum for all subsequent hemadsorption inhibition experiments. For each assay, bacterial stocks were thawed at room temperature, diluted with an equal volume of fresh H-broth, and then inoculated into 12-well flat-bottom tissue culture plates (Corning®, Lowell, MA). Overnight incubation at 37°C allowed adherence and formation of a bacterial monolayer; this step did not exceed 24 hours due to the variability of older cultures in binding red blood cells. The media was then removed and cells were gently washed twice with 1 ml of hemadsorption (HA) assay buffer (0.14 M NaCl, 1 mM MgCl_2_, 0.1 mM CaCl_2_, 0.01 M Tris HCl, pH 7.2) taking care to preserve the adherent monolayer. To standardize the results of our assay, rabbit antiserum was diluted between 1:20 and 1:640 (or as otherwise noted) based upon the titration results of Western spot blots testing reactivity between unpurified polyclonal rabbit sera and whole-cell G37-C lysates. Rabbit serum diluted in 750 ul HA assay buffer was added to appropriate wells and incubated for one hour at 37°C. Wells were washed three times with 1 ml HA assay buffer and then 750 ul of washed sheep red blood cells (Remel, Thermo Fisher Scientific, Lenexa, KS) diluted 1:10 in HA assay buffer was added. Hemadsorption was allowed to occur for one hour at 37°C, followed by four washes with 1 ml of HA assay buffer to remove non-adherent red blood cells. Bound erythrocytes were lysed by adding 500 ul of sterile dH_2_O; the A_450 nm_ of 200 ul of the resultant supernatant was then determined using a Fusion^TM^ microplate analyzer (Packard, Meriden, CT). Hemadsorption following antibody treatment was expressed as a percentage of the untreated control (bacteria mock treated with HA buffer) and a student’s unpaired t test was used to determine significance.

### Evaluation of MgpB Diversity among Strains

The nucleotide and amino acid sequence diversity of MgpB was evaluated using all available published MgpB sequences within Genbank, in addition to our own unpublished sequence data. These sequences were derived from a variety of sources including clinical strains propagated *in vitro*, as well as sequences amplified directly from patient specimens (S3 Table). Based on our previously defined boundaries of conserved and variable regions [20], sequences spanning the *mgpB* expression site were compared to *mgpB* from strain G37 (bp 1,066–5,400, Genbank Accession #M31431.1) using the Clustal Omega Multiple Sequence Aligner (http://www.ebi.ac.uk/Tools/msa/clustalo/) with subsequent nucleotide alignments manually adjusted. To evaluate amino acid diversity, nucleotide sequences (S3 Table) were translated *in silico* using EMBOSS Transeq (http://www.ebi.ac.uk/Tools/st/emboss_transeq/) and aligned using Clustal Omega. These alignments were used to calculate diversity scores (DS) [43] for overlapping segments of *mgpB* spanning 24 nucleotides or 8 amino acids by dividing the number of sequences different from G37 by the number of sequences analyzed. Thus, DS = 0 indicates 100% identity between strains while DS = 1 indicates 100% diversity. The DS was calculated for nucleotide (DS_nt_) and amino acid (DS_aa_) sequences, thereby identifying regions where DS_nt_ > DS_aa_. In addition to evaluating diversity using G37 as a reference, a theoretical consensus sequence was generated based upon the most common residue found at each position within MgpB (S2 Fig). This consensus sequence was used as a reference in a final analysis in which the percentage of sequences containing a variant amino acid, insertion, or deletion was calculated for each amino acid position along MgpB.

### Immunogenicity of the MgpB Adhesin

Linear and conformational B-cell epitopes were predicted with BepiPred 1.0 (http://www.cbs.dtu.dk/services/BepiPred/) using a 0.9 threshold cutoff for 25% sensitivity and 91% specificity [44] and CBTOPE (http://www.imtech.res.in/raghava/cbtope/) with the default support vector machine threshold of 0.3 [45], respectively. To complement the *in silico* epitope predictions, we evaluated the reactivity of recombinant MgpB peptides to antibodies generated during persistent *M*. *genitalium* infection of non-human primates. Sera were obtained from two primates: A01220 [32], inoculated cervically and in multiple salpingeal pockets, and J00106, inoculated only in the cervix. A persistent cervical infection was established in each primate as evidenced by the recovery of viable bacteria over the eight weeks of the experiment, at which time both primates were treated with antibiotics to clear the infection. ELISAs were performed as described previously [32] using a 1:50 dilution of serum against 5 ug/ml of recombinant MgpB peptide, testing each in triplicate. As a positive control, each recombinant peptide was reacted with polyclonal rabbit serum diluted 1:1,000 and detected with goat anti-rabbit IgG peroxidase-conjugated secondary antibody to confirm binding of antigen to the wells. Significance was determined using the student’s unpaired t test comparing peak reactivity to pre-immune values.

### Ethics Statement

The evaluation of MgpB diversity used *mgpB* sequences originating from patient samples collected from persistently infected men enrolled in our Seattle treatment trial (MGM treatment trial [46]) and women enrolled in our study of STDs among commercial sex workers in Nairobi, Kenya (KOR study [7]). Both studies were approved by the University of Washington Institutional Review Board and all enrollees gave written informed consent. The primate models of *M*. *genitalium* infection adhered to the Guide for the Care and Use of Laboratory Animals of the National Institutes of Health and were approved by the University of Washington Institutional Animal Care and Use Committee. All procedures were performed with appropriate anesthesia and pain relief as determined by a veterinarian in order to reduce pain and stress to the animals. The production of rabbit antibodies was performed by Pacific Immunology (Ramona, CA) following standard, approved animal protocols.

## Results

### The Predicted MgpB Membrane Topology Suggests the Majority of the Adhesin is Extracellular, with the Protein Anchored by a Single C-terminal Transmembrane Domain

Previous investigations of MgpB membrane topology have produced conflicting models [34, 35]. To investigate the topology in more detail, we used eight different algorithms to identify putative transmembrane domains and compared our results with those published previously [39]. While two of these programs suggested internal transmembrane domains, the majority of programs predicted only two transmembrane domains: one in the N- terminus and a second in the C-terminus (Fig 1). The N-terminal transmembrane domain identified by these programs likely represents the signal peptide as it corresponds to the signal sequence predicted in the first 26–30 amino acids (Fig 1C). Interestingly, N-terminal sequencing of CHAPS-solubilized MgpB determined that the mature protein begins at amino acid 59 [39], suggesting the removal of a larger fragment of the pre-protein than is typical for Gram-positive, Gram-negative, and eukaryotic signal peptides (Fig 1C). The consensus of our evaluation, therefore, is a confident prediction that MgpB includes a single C-terminal transmembrane domain and suggests a topological model in which the majority of MgpB is extracellular with only a short cytoplasmic domain (~70 amino acids) at the extreme C-terminus (Fig 1B). The internal transmembrane domains identified only by TMpred and TopPred (S1 Table) overlap with those identified previously by the hydrophobic moment plot method of Eisenberg [36] (designated M1–M6; Fig 1A and S1 Table) [34, 39]. The increased hydrophobicity (S3 Fig) and low complexity of sequences within these regions explains how they are recognized by these two programs but not by the remaining six algorithms tested. Based on these results, and the experiments described below, we conclude that these internal regions are not true membrane spanning domains. Because our model predicts that the conserved C-terminus is divided by a transmembrane region that separates the C-terminus into two distinct domains, one extracellular and the other intracellular, for clarity we will subsequently refer to these regions as “proximal” and “distal”, respectively.

### The Majority of MgpB is Antibody Accessible and Surface Exposed, Substantiating the Predicted Membrane Topology

**Fig 2:**
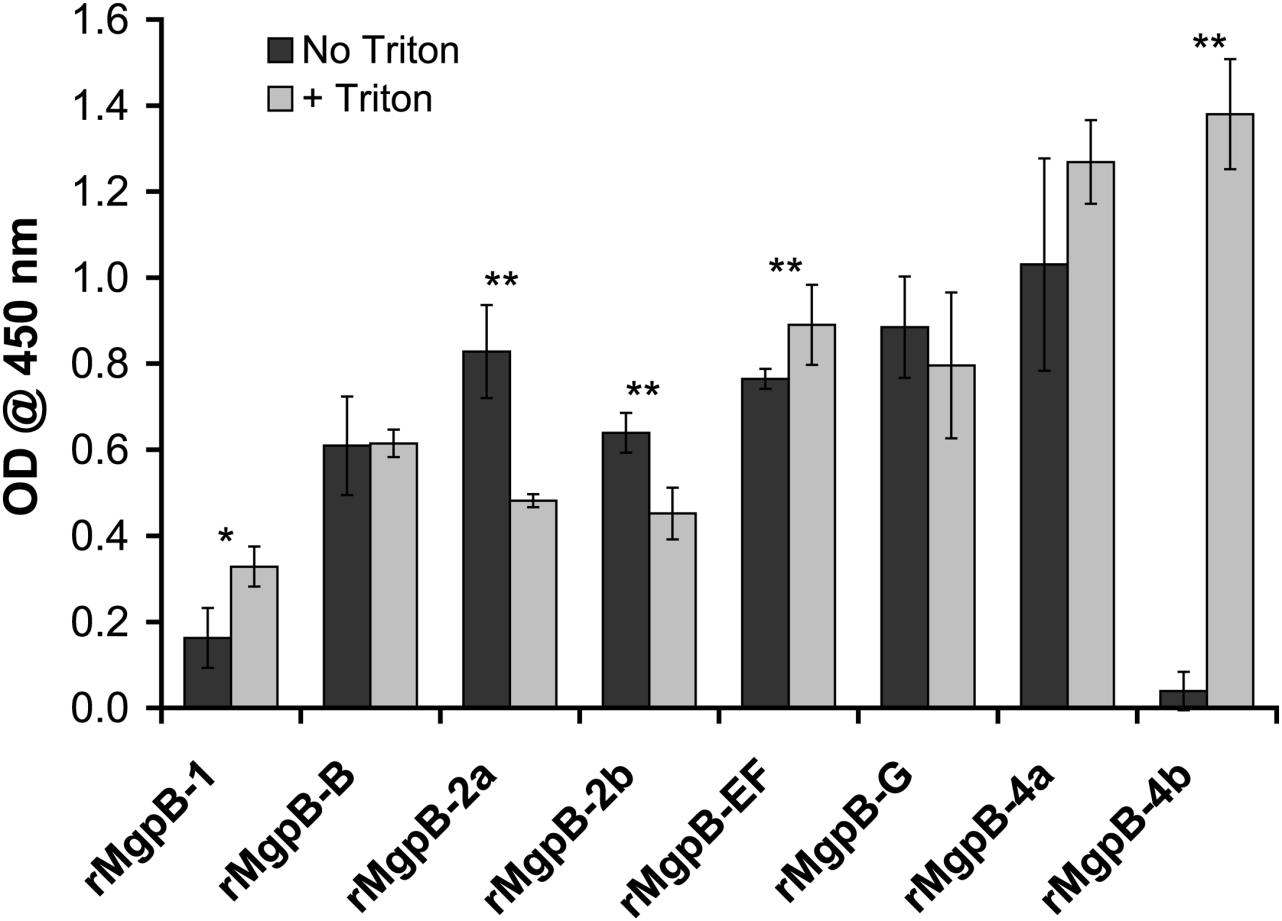
The Majority of MgpB-Reactive Antibodies Bind to the Surface of Intact *M*. *genitalium* Cells. Binding of region-specific antibodies to MgpB was measured using intact (PBS-treated, “No Triton” dark gray) and Triton X-100 permeabilized (“+ Triton”, light gray) *M*. *genitalium*. The increased reactivity with treated compared to untreated cells was significant for the conserved N-terminus (rMgpB-1; *, p = 0.024), variable region EF (rMgpB-EF; **, p < 0.001), and the putative cytoplasmic domain (rMgpB-4b; **, p = 0.0004); conversely there was a significant decrease in antibody binding in treated compared to untreated cells for the second conserved region (**, p = 0.007 and p = 0.0061 for rMgpB-2a and -2b, respectively). The assay was repeated in triplicate with results of a typical experiment shown above with each condition tested in quadruplicate; error bars indicate standard deviation.

To evaluate the membrane topology predicted above, we measured binding of antibodies specific for discrete segments of MgpB to intact or Triton X-100 permeabilized *M*. *genitalium* cells. We produced antibodies to recombinant peptides spanning specific regions of MgpB (Fig 1A) and found that those targeting variable regions B, EF, G, and the proximal conserved C-terminus bound to intact cells, indicating that these regions are antibody accessible and surface exposed. Similarly, antibodies to rMgpB-2a and -2b (both within the second conserved region) bound intact cells consistent with their surface accessibility and an extracellular location, although their reactivity decreased somewhat following Triton X-100 treatment (Fig 2), suggesting that their conformational epitopes were altered by the detergent. In stark contrast, antibodies to rMgpB-4b bound poorly to intact cells and reactivity increased >35-fold when cells were permeabilized (Fig 2), consistent with the predicted intracellular location of the distal conserved C-terminus. Antibody reactivity to rMgpB-1, although low overall, increased slightly less than 2-fold after permeabilization raising the possibility that the N-terminus is obscured by protein folding and/or interactions with other terminal organelle proteins. The likely exposure of the N-terminus on the cell surface is further supported by the absence of predicted transmembrane domains separating this region from extracellular portions of this protein (such as region B; Fig 1). Antibody accessibility of the third conserved region was not analyzed in this assay due to the cross reactivity of rabbit serum with an unknown *M*. *genitalium* protein (rMgpB-3; S1 Fig). Altogether, the antibody accessibility experiments agree with the consensus *in silico* prediction of a single C-terminal transmembrane domain and a topological model in which MgpB is predominantly surface exposed and anchored to the cell at the distal C-terminus.

### The Proximal Conserved C-terminus of MgpB Contains an Attachment Domain(s)

**Fig 3:**
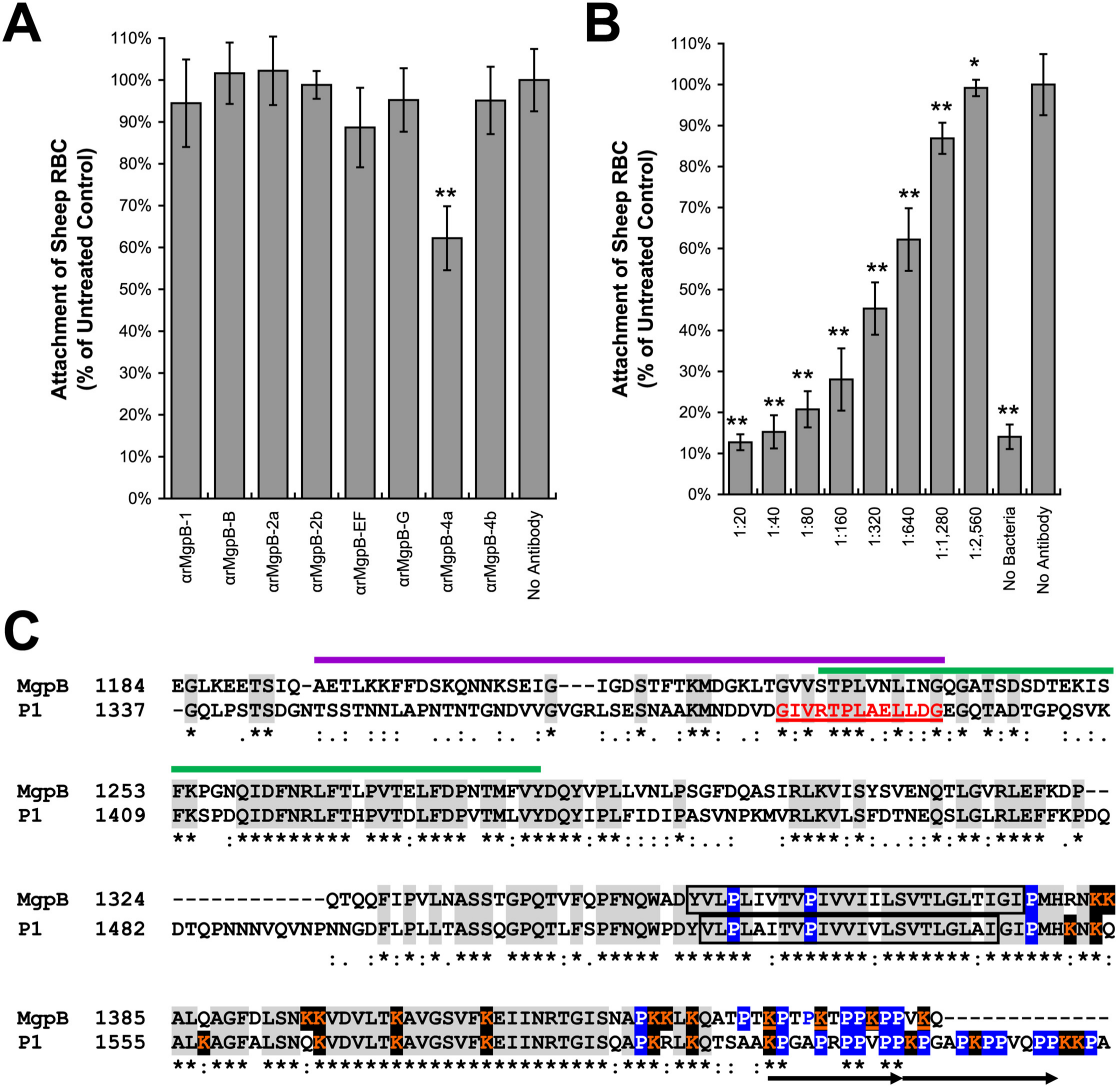
Attachment domains within MgpB are Located within the Proximal Conserved C-terminus and Share Homology with the Attachment Domain of the *M*. *pneumoniae* P1 Protein. (A) The results of the quantitative hemadsorption inhibition assay showing that antibodies reacting with the proximal conserved C-terminus (α-rMgpB-4a) diluted 1:640 inhibit hemadsorption by approximately 40% (**, p < 0.0001). At dilutions with comparable reactivity to MgpB (1:20, α-rMgpB-2b and-EF; 1:40, α-rMgpB-2a and-G; 1:80, α-rMgpB-1 and -4b; 1:320 α-rMgpB-B), antibodies reacting with other regions of MgpB have no statistical impact on the binding of red blood cells with results similar to untreated control wells not pretreated with antibodies. (B) Hemadsorption inhibition experiments demonstrating the inhibitory effect of α-rMgpB-4a antibodies was dose dependent; two-fold dilutions of this antibody ranging between 1:20 and 1:1,280 significantly decrease hemadsorption (**, p < 0.0001; *, p = 0.007) with lower dilutions completely inhibiting the binding of red blood cells with results comparable to the no bacteria control wells. In panels A and C, error bars represent standard deviation. (C) Alignment of the proximal and distal conserved C-termini of MgpB (aa 1,184–1,444) with the C-terminus of the homologous P1 adhesin (aa 1,337–1,627) of *M*. *pneumoniae*, for which the attachment domain has also been defined. Within P1, the location of peptides 7 and 8 [48] are noted with purple and green lines, respectively, and the binding epitope for the attachment inhibiting monoclonal antibody [49] (designated PepD) is underlined in red. Amino acids identically conserved between MgpB and P1 are highlighted in gray; additionally, predicted transmembrane domains predicted by TMHMM are boxed and the location of cytoplasmic proline (white/blue) and lysine (orange/black) residues are indicated. The arrows beneath the P1 sequence at the distal conserved C-termini note the location of a simple tandem repeat of unknown significance.

To identify the location of functional adhesin epitopes within MgpB, we used a quantitative hemadsorption inhibition assay [41] to determine if antibodies to individual regions of MgpB blocked binding of sheep red blood cells. As shown in Fig 3A, when polyclonal α-rMgpB antibodies are standardized to have similar reactivity to *M*. *genitalium* lysate, only α-rMgpB-4a antibodies statistically inhibited *M*. *genitalium* hemadsorption while pretreatment with antibodies binding other regions of MgpB had no effect. Furthermore, a two-fold dilution series of α-rMgpB-4a antibodies demonstrated this inhibitory effect was dose-dependent (Fig 3B). Although antibodies targeting the third conserved region (between EF and G) were not evaluated due to their cross-reactivity with an unknown *M*. *genitalium* antigen (S1 Fig, panel B), our results indicate that an attachment epitope(s) lies within the antibody accessible region of the conserved C-terminus between amino acids 1,187–1,368. These results are consistent with those reported previously for P1 [35, 47–49], the MgpB homologue expressed by *M*. *pneumoniae*, a closely related respiratory pathogen. Polyclonal antibodies specific for the proximal C-terminus of P1 inhibit binding of *M*. *pneumoniae* to MRC-5 (human fetal lung fibroblasts) and HBEpC (human bronchial epithelial cells) [47] and HEp-2 [35]. In addition, synthetic oligopeptides spanning this region decrease adherence to HEp-2 cells by 62–79% [48] (peptides 7 and 8, Fig 3C) while a monoclonal antibody that binds a 13 amino acid epitope (PepD, Fig 3C) inhibits attachment of *M*. *pneumoniae* to chicken erythrocytes [49, 50].

### Amino Acid Diversity is Concentrated in Previously Defined Variable Regions

**Fig 4:**
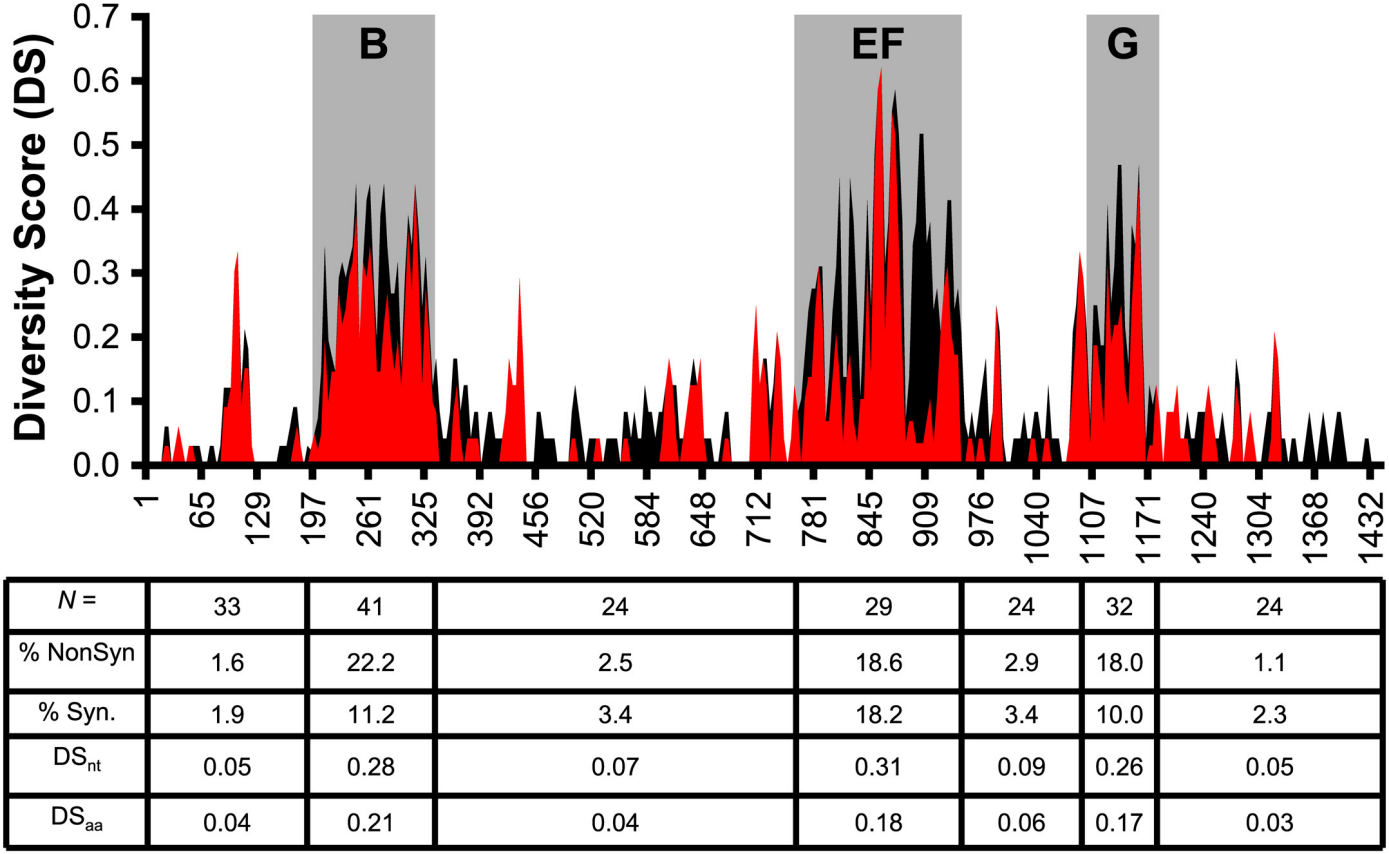
The Distribution of Synonymous and Non-Synonymous Mutations Differ within Defined Regions of the *mgpB* Expression Site. Nucleotide (DS_nt_, in black) and amino acid diversity scores (DS_aa_, in red) for overlapping windows of 12 nucleotides or 4 amino acids, respectively, are shown on the y-axis with MgpB amino acid position on the x-axis. As detailed in Materials & Methods, a DS = 0 indicates 100% sequence conservation while a DS = 1 indicates 100% variability. Synonymous mutations are apparent in regions where black peaks are visible indicating that the DS_nt_ is greater than DS_aa_ (for example, aa 880–920 of region EF). Below the x-axis, the total number of sequences analyzed per region (*N*), the average % of non-synonymous and synonymous mutations, and the average DS values for each region are listed.

Regions B, EF, and G in the *mgpB* expression site accumulate nucleotide diversity by recombination with homologous MgPar donor sites [20, 21], yet the extent to which these changes affect amino acid, and possibly antigenic, diversity compared to other regions of this protein has not been assessed. To explore this further, we aligned all available complete and partial *mgpB* nucleotide sequences (S3 Table) and determined that variable regions B, EF, and G contain more nucleotide substitutions (15–17% vs. 1.2%, p < 0.0001) and indels (2.6–3.5% vs. <0.6%, p = 0.001) than conserved regions, consistent with previous findings [22]. Importantly, with the exception of a single nucleotide insertion identified within region EF of strain M6320 (S3 Table), indels always occurred in triplicate thereby maintaining the correct reading frame. *In silico* amino acid predictions similarly indicated that indels were uncommon in conserved compared to variable regions (<0.6% vs. 3–4%, p = 0.0002). In this analysis, we also found that the conserved and variable regions differed in the ratios of synonymous and non-synonymous mutations: conserved regions had slightly more synonymous than non-synonymous mutations while regions B and G had nearly twice as many non-synonymous as synonymous mutations (Fig 4). Unlike the other variable regions, region EF contains equal numbers of synonymous and non-synonymous mutations (18.2% vs. 18.6%, Fig 4). To examine the distribution of non-synonymous and synonymous mutations across *mgpB* more precisely, we calculated diversity scores (DS, see Materials & Methods) for overlapping windows of 24 nucleotides (DS_nt_) or eight amino acids (DS_aa_); regions where DS_nt_ > DS_aa_ indicate high levels of synonymous mutations. In addition, this analysis (Fig 4) mapped areas of amino acid diversity within MgpB and showed a concentration of heterogeneity within and around variable regions B, EF, and G, as well as smaller peaks of diversity within conserved regions, including a highly variable region near amino acid 100 used to differentiate *M*. *genitalium* strains [8].

**Fig 5:**
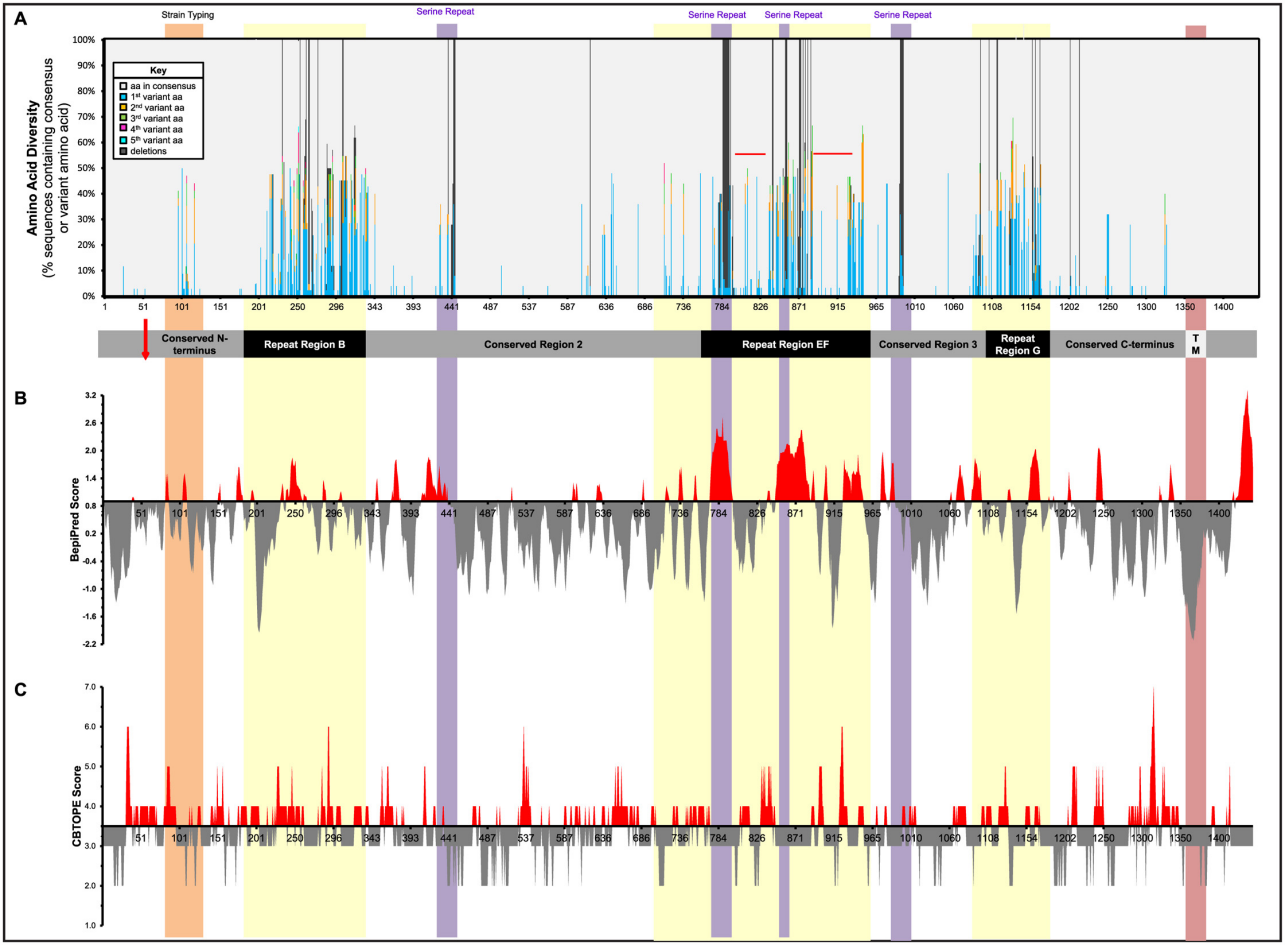
Amino Acid Diversity and Predicted B-cell Epitopes are Distributed throughout the MgpB Adhesin. (A) Amino acid diversity within the MgpB adhesin is concentrated within and around variable regions B, EF, and G. All available *mgpB* sequences were translated *in silico* and aligned to create a hypothetical consensus sequence; the percentage of sequences matching the consensus at each residue is shown in gray, while the percentage of sequences with variant amino acids is noted with colored bars as indicated in the key above and as follows: (1) blue, second most common residue (meaning the most common variant differing from the consensus), (2) orange, third most common residue, (3) green, fourth most common, (4) pink, fifth most common, and (5) aqua, sixth most common residue. Deletions (as compared to the consensus) are indicated by dark gray bars descending from the top of the graph. As an example, at the first amino acid, 100% of the sequences encode methionine (represented by a solid gray line), whereas five different amino acids were detected at position 117 [noted in gray (the consensus), blue, orange, green, and pink (four variants)]. The strain typing region and serine repeats are designated in orange and purple, respectively, while dashed red lines indicate the semi-conserved regions in EF that contain a high number of synonymous mutations. The location of the MgpB conserved and variable regions are noted below the x-axis in gray and black, respectively, as is the location of the C-terminal putative transmembrane domain (TM) and the experimentally determined start of the mature protein (red arrow). (B) The location of predicted linear B-cell epitopes, as determined using the BepiPred 1.0b Server. High (>0.9) and low (<0.9) scoring epitopes are indicated in red and gray, respectively. (C) The location of predicted conformational epitopes, as determined using the CBTOPE algorithm. Residues with scores above 4.0 indicate conformational epitopes and are shaded red (those below the 4.0 cutoff are in gray). Vertical shading indicates the following: strain typing region (orange), amino acid diversity including and surrounding the three variable regions (yellow), serine repeats (purple), and the C-terminal transmembrane domain (red).

To further explore such “hot-spots” of heterogeneity within the conserved and variable regions, we aligned all available MgpB sequences with a hypothetical consensus sequence containing the most common amino acid found at each position (S2 Fig). This consensus sequence was then used to determine the number of different residues found at each position revealing that many strains vary at a given amino acid position (as indicated by the height of each peak, Fig 5A) and furthermore, that certain positions contain a wide variety of different amino acids (as indicated by the number of different colors within each peak, Fig 5A). Region B is the most diverse with as many as six different amino acids found at some positions and stretches of sequence identity never exceeding more than four amino acids, a finding that is reflected in the elevated DS_aa_ score (Fig 4). Region EF and G were also highly diverse, but region EF contains two internal semi-conserved regions corresponding to stretches of synonymous mutations (aa 791–835 and 882–927, Fig 4 and dashed lines, Fig 5A). Small regions of variability were identified within conserved regions including the strain typing region and the variable serine repeats found in the second and third conserved regions and in region EF, which result from variation in the number of “TCT” and “AGT” repeats among strains, as previously reported [20, 21, 51].

### Variable Region B and the Conserved C-terminus Elicit an Antibody Response in Experimentally Infected Primates

As presented above, the *mgpB* variable regions contain regions of amino acid diversity (Figs 4 and 5) that are predicted to be surface exposed (Fig 1) and experimentally shown to be antibody accessible (Fig 2), suggesting these specific portions of MgpB play a role in immune evasion. To explore this hypothesis further, we first correlated amino acid diversity with potential antigenicity using *in silico* analyses to identify putative B-cell epitopes. Linear B-cell epitopes are distributed throughout MgpB and are often found in regions that vary between strains (Fig 5B). Conformational B cell epitopes predicted from the primary amino acid sequence (the structure of MgpB is unknown) are similarly distributed throughout MgpB, however, there is no significant overlap between predicted conformational epitopes and amino acid diversity (Fig 5C).

**Fig 6:**
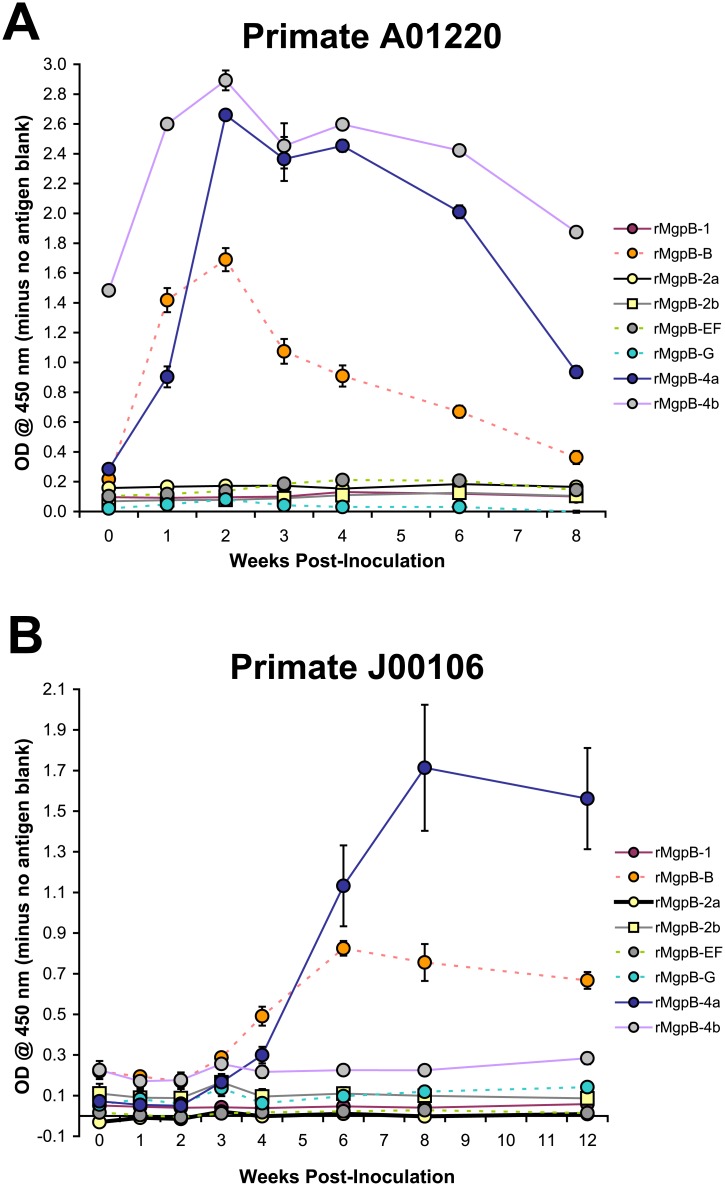
Antibodies Elicited During Persistent Experimental *M*. *genitalium* Infection of Non-Human Primates Recognize Variable Region B and the Conserved C-terminus. ELISA reactivity of primate serum collected at weekly time points over the course of infection measured against the recombinant MgpB proteins spanning the conserved (rMgpB-1, -2a, -2b, -4a, and -4b) and variable regions (rMgpB-B,-EF, and-G), as indicated in the legends to the right. ODs shown represent values after subtracting the readings from no antigen control wells. (A) In primate A01220 (cervical and salpingeal pocket infection), antibody reactivity to recombinant proteins spanning the variable rMgpB-B, as well as the proximal and distal conserved C-terminus (rMgpB-4a and -4b), increased during infection, peaking at week 2 post-inoculation. (B) Primate J00106 (cervical infection only) exhibited a similar pattern of reactivity against rMgpB-B and -4a, with the antibody response peaking around 8 weeks post-infection. Data above represents the average of two independent experiments, with each condition tested in triplicate.

To substantiate these *in silico* predictions, we next characterized the antibody response to conserved and variable regions of MgpB over the course of persistent *M*. *genitalium* infection in two pig-tailed macaques (*Macaca nemestrina* [32]) experimentally infected with G37. As detailed in materials and methods, serum specimens collected from primates A01220 (inoculated cervically and in abdominal salpingeal pockets) and J00106 (inoculated cervically only) were analyzed for reactivity to recombinant proteins spanning MgpB (Fig 1A). Antibodies arose earlier in A01220, which is likely due to this primate being exposed to a higher concentration of *M*. *genitalium* through inoculation at multiple sites (cervix and salpingeal pockets). Sera from both primates reacted to the proximal conserved C-terminus (rMgpB-4a, Fig 6) consistent with the immunogenicity of the conserved C-terminus documented previously in persistently infected humans [26, 28]; in addition, both primates demonstrated strong reactivity to variable region B (rMgpB-B, Fig 6). Primate A01220 serum reactivity to rMgpB-B and -4a increased eight- and nine-fold, respectively (comparing weeks 0 and 2) while similarly, three- and 23-fold increases in antibody reactivity to the same antigens was observed in primate J00106 (comparing weeks 0 and 8). Surprisingly, with the exception of a two-fold increase in reactivity to the distal conserved C-terminus exhibited by A01220 (rMgpB-4b, Fig 6A), primate sera failed to react in ELISAs with recombinant proteins spanning the other variable and conserved regions of MgpB. This was not due to poor binding of these antigens to ELISA plates, as polyclonal rabbit sera reacted strongly with the appropriate immunogens (S1C Fig)

### MgpB Exhibits Homology to MgpC, in Addition to Other Unique Features such as Compositional Biases and Periodic Repeats

**Fig 7:**
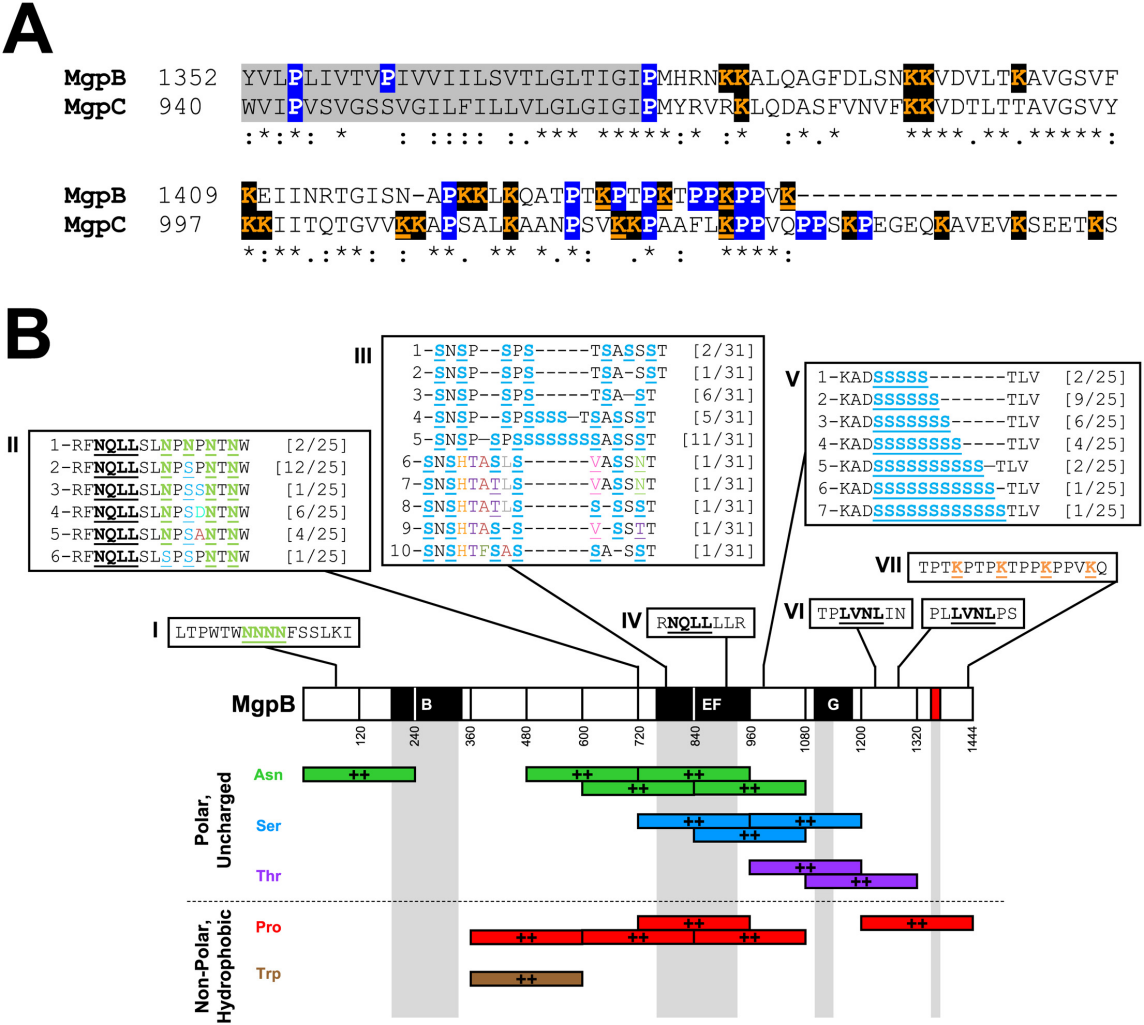
Unique Features of MgpB Including C-terminal Homology to MgpC, Periodic Repeats, and Overrepresented Amino Acids are Illustrated using *in silico* Analyses. (A) The distal C-termini of MgpB and MgpC are homologous, share periodic lysine repeats (every fourth or seventh amino acid; orange underline) and are proline-rich (blue). Remaining lysine residues not included within the periodic repeat are in orange/black. BLAST alignment of the predicted transmembrane domains (gray) and beyond for aa 1,352–1,443 and 940–1,053 of MgpB and MgpC, respectively, demonstrate the cytoplasmic domain of MgpB has significant homology to aa 940–1,032 of MgpC with an e-value of 1e-21. (B) Schematic diagram of MgpB showing amino acid repeat sequences and periodicity including (boxes): (I) asparagine repeats at aa 71 (green, underlined), (II) alternating asparagine repeats at aa 733 (green, underlined), (III) alternating serine repeats at aa 778 (blue, underlined), (IV) “NQLL” motif found twice at aa 727 and 908 (bold, dashed underline in boxes II and IV), (V) serine repeats at aa 989 (blue, underlined), (VI) “LVNL” sequence found at aa 1,233 and 1,288 (double underline), and (VII) lysine residues repeated every fourth amino acid in the threonine/proline-rich distal conserved C-terminus, aa 1,431 (orange, underlined; also shown in panel B). Sequences in boxes I, IV, VI, and VII were 100% conserved in all strains while sequences containing alternating asparagine and serine repeats varied, indicated by numbered sequences within boxes II, III, and V. Sequence 1 corresponds to G37 and the number of variants expressing each unique sequence is shown to the right in brackets (see S2 Fig for a complete alignment). Shown below is an illustration of the overrepresented amino acids within overlapping ~240 aa segments of MgpB, determined using the SAPS *B*. *subtilis* reference set; overrepresented residues (99% quantile point) are indicated by “++”. The complete SAPS analysis including underrepresented amino acids and comparisons to other reference sets (*E*. *coli* and a random sample of proteins) can be found in S4 Table.

To explore unusual features of MgpB which may provide insight for future experiments, we first explored homology between the primary adhesin MgpB and the cytadherence accessory protein MgpC. Interestingly, MgpC has a predicted membrane topology similar to MgpB with a short cytoplasmic C-terminus anchored in the membrane with the majority of the protein exposed on the cell surface [24]. Given this observation, in addition to other common features shared by MgpB and MgpC (for example, essential role in adherence, reciprocal stabilization [15], and ability to antigenically vary through recombination of the *mgpB* and *mgpC* genes with the MgPar sequences [20, 21, 23, 24]), we explored homology by aligning the MgpB and MgpC sequences with each other. In this analysis, we found that MgpB and MgpC share a high degree of homology in the distal cytoplasmic C-termini [45% (42/93) identity and 62% (58/93) similarity Fig 7A]. It is interesting to note that despite this high degree of homology, antibodies against the distal cytoplasmic C-termini of MgpB (α-rMgpB-4b) did not show cross-reactivity with the full-length MgpC protein (S1 Fig panel B) in immunoblots.

Finally, we analyzed MgpB using the SAPS algorithm, which evaluates protein sequences for significant differences in compositional biases compared to a reference set of proteins, as well as unique clusters/repeats of amino acids and abnormal spacing between identical residues [37, 52, 53]. Because mycoplasmas are thought to have evolved from a Gram-positive ancestor through genome reduction [54, 55], we compared MgpB to the *B*. *subtilis* reference set. As summarized in Fig 7B and S4 Table, MgpB contains significantly more proline, threonine, and asparagine residues than average, while methionine is underrepresented. Given the large size of MgpB (~140 kDa), to more precisely identify defined regions within MgpB with unusual amino acid composition, the SAPS analysis was repeated using overlapping ~240 aa segments revealing an overrepresentation of threonine, asparagine, and serine within the second half of the protein (comprising 29.9% of amino acids; Fig 7B and S4 Table). This is especially true within variable regions EF and G and the intervening conserved region (32.8–38.3%), as compared to the threonine, asparagine, and serine content for MgpB as a whole (26.3%). Proline and tryptophan are also enriched within the central region of MgpB, possibly explaining the misidentification of membrane spanning domains within this portion of the protein by a minority of prediction programs (S1 Table).

The SAPS analysis also identified seven regions within MgpB of G37 with repeated sequences or unusual periodicity (Fig 7B). These include four consecutive asparagine residues in the N-terminus, alternating asparagines upstream of variable region EF, and two motifs (NQLL and LVNL) each repeated twice (Fig 7B, boxes I, II, IV, and VI). Serine repeats identified within variable region EF and immediately downstream (Fig 5) were also identified by SAPS (Fig 7B, boxes III and V) although the serine repeats in conserved region 2 (Fig 5) were not detected because G37 contains only three serine residues at this location while other strains have as many as nine (S2 Fig). Interestingly, the serine and alternating asparagine repeats are located within regions that vary among *M*. *genitalium* strains (Fig 7B, boxes II, III, and V and S2 Fig). Finally, the distal conserved C-terminus of MgpB contains an abundance of proline residues (Fig 7B), previously suggested to provide rigidity to this portion of the protein [34], as well as periodic lysine repeats (Fig 7B, box VII). It is important to note that the periodic lysine repeats and abundance of proline residues found in MgpB are conserved in MgpC (Fig 7A).

## Discussion

In the genomically reduced pathogen *M*. *genitalium*, the multifunctional and antigenically variable MgpB protein is required for adherence and motility, and is possibly involved in the long-term survival of bacteria during persistent infection. In addition, the MgpB adhesin is the predominant antigen targeted by antibodies induced during naturally acquired persistent infection in men [27] and women [25–28] and in experimentally infected non-human primates [32] clearly demonstrating that a humoral response is insufficient to clear bacteria from the lower genital tract. Three distinct regions known as B, EF, and G within the *mgpB* expression site vary extensively within the type strain G37, *in vivo* in persistently infected men and women, and in our primate model of chronic infection [20, 21, 32]. While it has been hypothesized that MgpB variants are selected in response to the host antibody response, research regarding the relationship between antigenic variation and the location of functional and/or immunogenic epitopes is lacking. In this study, we provide a comprehensive analysis of the MgpB protein in terms of amino acid diversity, antibody accessibility, location of functional and immunogenic domains, as well as other unique properties related to homology and amino acid composition (summarized in Table 1). As expected, our results show that amino acid diversity is concentrated within the previously defined variable regions and can be explained by homologous recombination with MgPar sites, while the small segments of diversity within the conserved regions generally resulted from single nucleotide polymorphisms or from variation in the number of serine repeats. Interestingly, we found that the conserved regions flanking variable region G, and to a lesser extent region EF, contain clusters of amino acid diversity that we speculate may result from errors introduced during the recombination process, perhaps during the resolution of recombination intermediates.

**Table 1:** Summary of Antibody Accessibility, Hemadsorption Inhibition, Amino Acid Variation and Immunogenicity of MgpB.

Region	Conserved N-terminus	Variable Region B	Conserved Region 2	Variable Region EF	Conserved Region 3	Variable Region G	Proximal C-terminus	Extreme C-terminus
**Homology to MgPar Sites**	No	Yes	No	Yes	No	Yes	No	No
**Predicted Membrane Topology**	Extracellular	Extracellular	Extracellular	Extracellular	Extracellular	Extracellular	Extracellular	Intracellular
**Antibody Accessible**	Yes	Yes	Yes	Yes	ND^a^	Yes	Yes	No
**Hemadsorption Inhibition**	No	No	No	No	ND	No	Yes	No
**Diversity Score (DS**_**aa**_ **)**	0.04	0.21	0.04	0.18	0.06	0.17	0.03	
**Reactive with Antibodies from Infected Primates**	No	Yes	No	No	ND	No	Yes	No
**Periodic Repeats**	NNNN		NXNXNXNX^b^	SXSXSXSXSXSXS	SSSSS			KXXXKXXXKXXXK
**Overrepresented Amino Acids**	Asparagine		Asparagine Proline Tryptophan	Asparagine Serine Proline	Asparagine Serine Threonine Proline	Serine Threonine	Threonine Proline	Proline

^*a*^ ND—not determined

^*b*^ “X” indicates any amino acid

If MgpB variation functions to avoid antibody recognition, then we would predict variable regions to be surface-exposed and antibody accessible. Early experiments [34] predicted six transmembrane domains in MgpB suggesting a topology in which the protein crosses the membrane several times and forms a tertiary structure composed of domains that roughly correspond to the variable regions B, EF, and G, as well as the conserved C-terminus (N, D1, and D2, Fig 1A). In a subsequent study, however, only the C-terminus (also including region G) was determined to be surface exposed and required for attachment to HEp-2 cells [35]. Our data support a model in which MgpB is anchored in the membrane by a single C-terminal transmembrane domain with the majority of the protein exposed on the cell surface and a small intracellular domain in the distal conserved C-terminus. Antibodies specific for all regions of MgpB except the distal conserved C-terminus reacted to intact cells suggesting that most of the protein is surface exposed. While antibody accessibility may be masked by protein folding and/or interactions with other proteins and may therefore not definitively localize intra- and extracellular domains, the inaccessibility of the distal conserved C-terminus in intact cells in addition to its relative location to the predicted transmembrane domain is compelling and suggests this portion of MgpB is cytoplasmic.

The results of our quantitative hemadsorption inhibition assay indicate that the MgpB attachment domain(s) is located within the proximal, extracellular portion of the conserved C-terminus. While this finding is in contrast to the tertiary MgpB model proposed by Opitz, et al [34] discussed above, they are consistent with previous experiments [35] that mapped the adherence domain to amino acids 1,075–1,444 (region G and entire conserved C-terminus). Our results narrow the attachment domain to amino acids 1,187–1,368 as antibodies targeting flanking regions (region G and the distal conserved C-terminus) had no effect on hemadsorption. The attachment domain within the homologous P1 protein of the closely related respiratory pathogen *M*. *pneumoniae* similarly maps to the proximal C-terminus [48, 49, 56] as evidenced by: (1) antibodies to the C-terminus inhibit attachment of *M*. *pneumoniae* to various cell types [35, 47, 49, 50] (2) peptides corresponding to this region inhibit attachment 61–79% [48], and (3) an attachment-inhibiting monoclonal antibody binds an epitope within this region (PepD, Fig 3C;[49, 50]). Future studies are needed to more precisely map the location of attachment domain(s) within the adhesin of *M*. *genitalium* and to determine if P1 and MgpB bind a common oligosaccharide receptor on host cells. In addition, it is tempting to speculate that binding to different sialic acid containing receptors may be responsible, in part, to the differential host environments colonized by these closely related human pathogens. While the receptors involved in *M*. *genitalium* binding to spermatozoa [57], cultured epithelial cells [35, 58], and the surface and cilia of Fallopian tube epithelial cells in organ culture [59] have not been identified, hemadsorption of *M*. *genitalium* to guinea pig and sheep erythrocytes, used as surrogates for binding of these cell types, involves sialic acid receptors [60]. *M*. *pneumoniae* also interacts with sialic acid, in this case specifically α-2,3-linked sialic acid, which is present on many host proteins including membrane proteins of red blood cells [61].

The results presented herein provide an important foundation for understanding the role of MgpB in *M*. *genitalium* pathogenesis and will provide insights into future studies examining the interactions of MgpB with other proteins in the attachment organelle. For example, MgpB and MgpC reciprocally stabilize each other [15] suggesting an interaction between these proteins or a common intermediate. The homology between the distal conserved C-termini of MgpB and MgpC raises the possibility that these conserved intracellular domains may facilitate such an interaction, possibly in the electron dense core of the terminal organelle. At this point these observations are purely speculative and future studies are needed to experimentally determine the role of the MgpB and MgpC C-terminal cytoplasmic domains in the function of these two proteins and their role in stabilization of the terminal organelle, attachment, and motility. Additionally, our *in silico* identification of the periodic lysine repeats within the C-terminal region of MgpB and MgpC is also of interest and suggests a role in post-translational modification and regulation. Interestingly, recent studies with *M*. *pneumoniae* document lysine acetylation in many proteins in this species, which the authors suggest may be important to the cell biology of this organism and other Mycoplasma species [62]. Interestingly, P1 was not identified in the lysine acetylome [62] although P1 lacks the periodic lysines found in MgpB (Fig 3C). This observation potentially indicates that such modifications may be uniquely relevant to the MgpB protein in *M*. *genitalium*.

The amino acid sequence of MgpB contains a high proportion of serine residues, including stretches of serine repeats, yet the role of these amino acids and their involvement in the structure and function of MgpB, as well as MgpC, which also contains similar serine repeats [21, 22] remains unknown. In other bacterial species, proteins with serine-rich repeats have been better characterized including a family of adhesins known as serine-rich repeat proteins (SRRPs) expressed by Gram-positive bacteria. Among this family of proteins is the Fap1 protein of *Streptococcus parasanguinis* and the human platelet binding protein GspB of *Streptococcus gordonii*, which both undergo O-linked glycosylation [63]. Glycosylation of mycoplasma proteins has been documented, although the function of these modifications has not been defined; for example, an abundance of O-linked glycosylated serine and threonine residues was recently identified in proteins of *Mycoplasma arthritidis* [64]. In other bacterial species glycosylated bacterial proteins serve multiple purposes including enhanced attachment in *Chlamydia trachomatis* [65], immunomodulation through glycomimicry in group A and group B streptococci, *Neisseria meningitidis*, and *E*. *coli* [66], and enhanced microheterogeneity of human *Neisseria* species [67] with the expression of different glycoforms. We hypothesize that the high concentration of serine, threonine, and asparagine residues in MgpB may promote glycosylation of MgpB, potentially allowing an additional level of immune escape beyond that achieved through variation of the amino acid sequence alone.

As detailed above, we hypothesize that antigenic variation in regions targeted by antibodies in vivo allows *M*. *genitalium* to persist during chronic infections. Accordingly, we measured the reactivity of sera collected from non-human primates experimentally infected with *M*. *genitalium* to recombinant proteins spanning the conserved and variable regions of MgpB. Sera from both primates reacted to the proximal conserved C-terminus, consistent with previous observations that antibodies targeting this region are elicited in infected humans [26, 28]. Among the variable regions, reactivity was strongest to region B, which exhibits the highest diversity score and percentage of non-synonymous mutations. Supporting the role of variable region B in antigenic variation and avoidance of antibodies, in our initial study of primate A01220, we demonstrated that the original region B sequence of the infecting strain was replaced by a region B variant that predominated after eight weeks of infection and furthermore, that antibody reactivity to this variant was reduced [32]. In the current study, we unexpectedly observed poor antibody reactivity to variable regions EF and G in both primates, despite the presence of predicted linear B-cell epitopes in these highly variable regions. The absence of IgG reactivity to these two variable regions may reflect differences in conformational epitopes and/or post-translational modifications between the recombinant and native proteins. Glycosylation, for example, may interfere with the binding of antibodies to rMgpB-EF and-G in ELISA experiments, a distinct possibility considering the high percentage of asparagine, threonine, and serine residues within and around these two variable regions. Alternatively, the production of antibodies directed against regions EF and G may be inhibited by other bacterial factors. For example, in addition to being highly variable, regions EF and G may also be poorly immunogenic similar to the *Streptococcus pyogenes* fibrillar M protein that contains a non-immunogenic, hypervariable N-terminus and a conserved, highly immunogenic C-terminus [68]. In this case, anti-M antibodies produced during infection that predominantly recognize the conserved C-terminus are of little benefit, while antibodies recognizing the N-terminus (not elicited during active infection) are highly protective following passive immunization [68]. Likewise in *M*. *genitalium*, the proximal conserved C-terminus of MgpB is highly immunogenic and the mechanism used by bacteria to avoid clearance by these antibodies is of utmost interest.

The emergence of *M*. *genitalium* as an important sexually transmitted pathogen with potentially serious sequelae highlights the need for a molecular understanding of pathogenesis. The results of our analyses indicate that MgpB contains features that may impact the pathogenesis and persistence of this organism. The majority of the MgpB adhesin is antibody accessible and subject to immune pressure and while the immunogenicity and diversity of region B suggests that sequence changes facilitate bacteria survival, the lack of antibody reactivity with variable regions EF and G in *M*. *genitalium* persistent infections and the robust antibody response to the proximal conserved C-terminal region indicate other immune evasion mechanisms likely exist. In addition to the non-specific binding of IgG by the newly characterized MG281 protein [69], other immune evasion strategies may include phase variation, post-translational modification, protein folding and/or interactions with MgpC that obscures important MgpB epitopes. Additionally, important antibody-binding domains within the MgpB adhesin may be masked when bacteria intimately associate with host cells [70] when the attachment organelle is buried within the host cell membrane as bacteria create depressions or pits within the host cell membrane [71]. As reviewed by Taylor-Robinson and Jensen [4], this invagination of the plasma membrane is thought to precede host cell invasion and while the *in vivo* significance of *M*. *genitalium* intracellular infection [72] remains unknown, occupying an intracellular niche would facilitate avoidance of the immune response, thereby enhancing *M*. *genitalium* pathogenicity. There is no doubt much remains to be understood about the virulence and persistence of this parasitic urogenital tract bacterium. Our work to define the relationship between MgpB sequence diversity, membrane topology, and immunogenicity presented in this study provides important support to the overarching hypothesis that MgpB undergoes antigenic variation to promote chronic infection, illuminating the unique and complex role MgpB plays in the molecular biology and pathogenesis of this sexually transmitted pathogen.

## Supporting Information

S1 Fig Recombinant MgpB proteins and Reactivity of Antibodies against the full-length MgpB in *M*. *genitalium* G37. (A) Schematic of MgpB showing the location of sequences contained within recombinant MgpB proteins, designated below. Variable regions B, EF, and G (black boxes) contain sequences homologous to those present in the MgPar sites, while conserved regions (no fill) contain sequences unique to the *mgpB* expression site. Within the conserved C-terminus, we designed two recombinant proteins spanning either side of the putative transmembrane domain (M6, in red). The mature MgpB protein begins at amino acid 59 following the presumed signal peptide (red box, [39]); the remaining predicted transmembrane domains M1 through M5 initially described for MgpB [34, 39] are shown for reference. (B) Sera from rabbits immunized with each antigen react to the full-length MgpB protein present in lysates of *M*. *genitalium* G37 (left), but not with lysates of Δmg191, the MgpB deletion mutant [15] (right). Sera from two rabbits immunized rMgpB-3 both reacted to an unrelated ~70 kDa antigen (green arrow) expressed in both the wild-type and Δmg191 deletion mutant, in addition to the intended MgpB target (blue arrow). As a result, antibodies against this conserved region were omitted from subsequent experiments. Importantly, pre-immune serum from all rabbits did not react to *M*. *genitalium* lysate (results above are representative of one immunized rabbit). (C) ELISA assays using hyperimmune rabbit sera demonstrate efficient binding of recombinant antigens to microwells, used as a positive control for ELISA assays assessing primate sera reactivity (Fig 6). Data above represents the average OD from a single experiment with serum diluted 1:1,000, tested in quadruplicate, with the results of assays with no antigen subtracted as a blank; error bars indicate standard deviations.
(PDF)

S2 Fig *mgpB* sequence alignments for the evaluation of diversity within and among strains. All available *mgpB* sequences were compared to the *M*. *genitalium* G37 reference strain using Clustal Omega. Alignments were manually adjusted to evaluate the number of synonymous and non-synonymous mutations, with the predicted amino acid sequence and variants shown below. Nucleotides that differ from the G37 type strain are highlighted in gray if they are predicted to result in a silent, synonymous mutation; non-synonymous mutations are highlighted in yellow, green, aqua, pink, or blue. Indels are also noted with deletions (compared to G37) highlighted in black, and insertions (again compared to G37) noted in red. The dominant residue found in the majority of sequences at each amino acid position was included in a theoretical consensus sequence, shown in Fasta format at the top. Also noted is the variation within the unique amino acid sequences identified using the SAPS. This analysis was conducted on sequences spanning each conserved and variable region within the *mgpB* expression site; each section notes the total number of sequences included in the analysis.
(PDF)

S3 Fig Hydrophobicity of Internal Transmembrane Domains within MgpB Identified by TopPred. Internal transmembrane domains identified with the TopPred program display lower hydrophobicity scores than the single C-terminal transmembrane domain agreed upon by the other algorithms tested (S1 Table). Four of these internal transmembrane domains that overlap with the previously defined M3, M4, and M5 [34], in addition to a new domain spanning aa 780–800, are identified “putatively” with hydrophobicity scores exceeding the lower cutoff (green line); two others that overlap with M1 and M2 have slightly higher scores exceeding the upper cutoff (red line) and are predicted with “certainty”, although their hydrophobicity is still lower than that observed in the C-terminal transmembrane domain. These internal transmembrane domains were not identified by six of the eight algorithms tested; those overlapping with M1, M2 and M5 identified here by TopPred were also predicted by the TMpred program (S1 Table).
(PDF)

S1 References List of Additional References Used in Supplemental Figures & Tables not Cited in Main Text. (PDF)

S1 Table Signal Peptide Sequences, Transmembrane Domains, and the Resulting Membrane Topology Predictions for the MgpB Protein. (PDF)

S2 Table Primers Used for Construction of HIS-tagged Recombinant MgpB Proteins. (PDF)

S3 Table Genbank Accession Numbers for *mgpB* Sequences used in Sequence Analysis. (PDF)

S4 Table Statistical Analysis of Over- and Underrepresented Amino Acids throughout MgpB. The MgpB protein from *M*. *genitalium* G37, analyzed by SAPs, identifies over- or under-represented amino acids in the full-length sequence (shown to the left of the thick black line), or within overlapping fragments of ~240 aa each (shown individually to the right). In the chart above, red cells noted with a “+” or “++” identify amino acids overrepresented compared to >95% or >99% of proteins within the reference set, respectively. Similarly, gray cells highlighted with “-”or “—”indicate amino acids underrepresented compared to <5% or <1% of proteins within the reference set, respectively. Each region was compared to reference sets of *B*. *subtilis* (“*B*. *sub*”), *E*. *coli*, or a random sample of proteins in the PDB database (“all”) reference sets.
(PDF)
