# Supplementary material for: Analysis of the Mycoplasma genitalium MgpB Adhesin to Predict Membrane Topology, Investigate Antibody Accessibility, Characterize Amino Acid Diversity, and Identify Functional and Immunogenic Epitopes
Source: PLoS One. 2015 Sep 18;10(9):e0138244. doi: 10.1371/journal.pone.0138244 (PMC4575044; doi:10.1371/journal.pone.0138244)
Supplement: S1 Fig — (A) Schematic of MgpB showing the location of sequences contained within recombinant MgpB proteins, designated below. Variable regions B, EF, and G (black boxes) contain sequences homologous to those present in the MgPar sites, while conserved regions (no fill) contain sequences unique to the mgpB expression site. Within the conserved C-terminus, we designed two recombinant proteins spanning either side of the putative transmembrane domain (M6, in red). The mature MgpB protein begins at amino acid 59 following the presumed signal peptide (red box, [39]); the remaining predicted transmembrane domains M1 through M5 initially described for MgpB [34, 39] are shown for reference. (B) Sera from rabbits immunized with each antigen react to the full-length MgpB protein present in lysates of M. genitalium G37 (left), but not with lysates of Δmg191, the MgpB deletion mutant [15] (right). Sera from two rabbits immunized rMgpB-3 both reacted to an unrelated ~70 kDa antigen (green arrow) expressed in both the wild-type and Δmg191 deletion mutant, in addition to the intended MgpB target (blue arrow). As a result, antibodies against this conserved region were omitted from subsequent experiments. Importantly, pre-immune serum from all rabbits did not react to M. genitalium lysate (results above are representative of one immunized rabbit). (C) ELISA assays using hyperimmune rabbit sera demonstrate efficient binding of recombinant antigens to microwells, used as a positive control for ELISA assays assessing primate sera reactivity (Fig 6). Data above represents the average OD from a single experiment with serum diluted 1:1,000, tested in quadruplicate, with the results of assays with no antigen subtracted as a blank; error bars indicate standard deviations. (PDF) [file pone.0138244.s005.pdf]

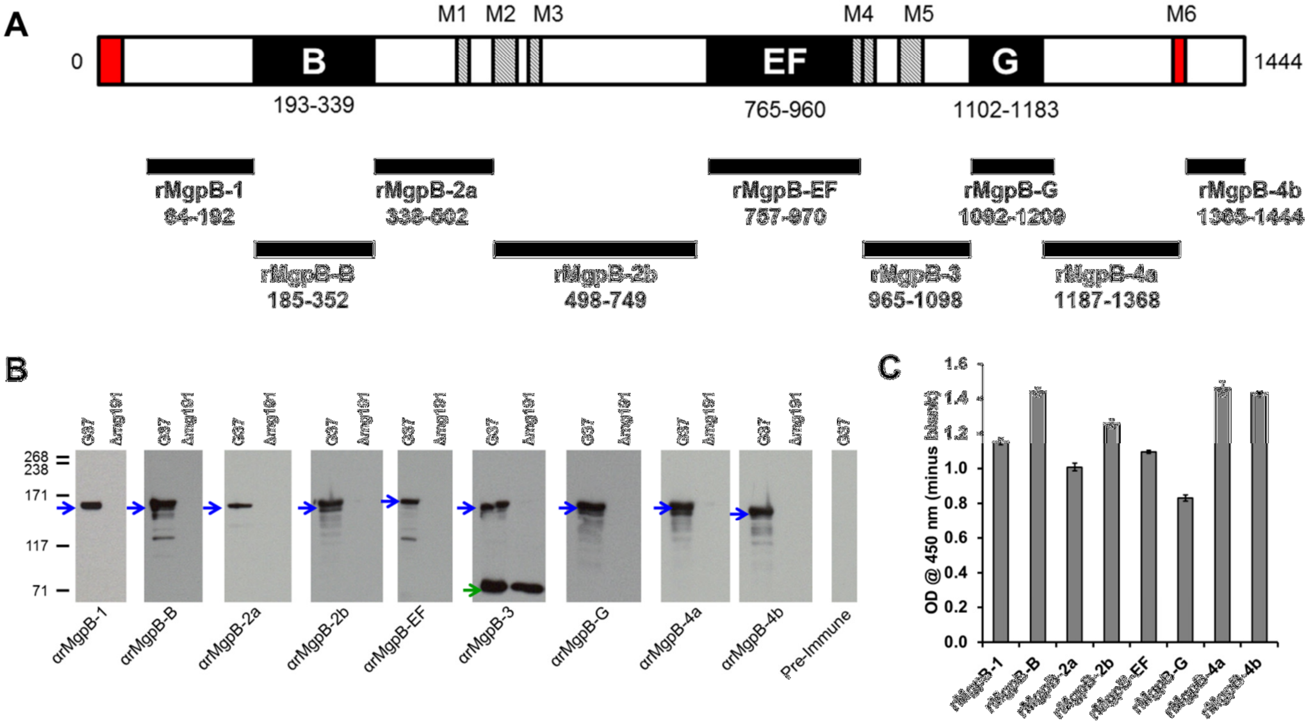

S1 Figure. Recombinant MgpB proteins and Reactivity of Antibodies against the full-length MgpB in *M. genitalium* G37

(A) Schematic of MgpB showing the location of sequences contained within recombinant MgpB proteins, designated below. Variable regions B, EF, and G (black boxes) contain sequences homologous to those present in the MgPar sites, while conserved regions (no fill) contain sequences unique to the *mgpB* expression site. Within the conserved C-terminus, we designed two recombinant proteins spanning either side of the putative transmembrane domain (M6, in red). The mature MgpB protein begins at amino acid 59 following the presumed signal peptide (red box, [39]); the remaining predicted transmembrane domains M1 through M5 initially described for MgpB [34, 39] are shown for reference. (B) Sera from rabbits immunized with each antigen react to the full-length MgpB protein present in lysates of *M. genitalium* G37 (left), but not with lysates of Δmg191, the MgpB deletion mutant [15] (right). Sera from two rabbits immunized rMgpB-3 both reacted to an unrelated ~70 kDa antigen (green arrow) expressed in both the wild-type and Δmg191 deletion mutant, in addition to the intended MgpB target (blue arrow). As a result, antibodies against this conserved region were omitted from subsequent experiments. Importantly, pre-immune serum from all rabbits did not react to *M. genitalium* lysate (results above are representative of one immunized rabbit). (C) ELISA assays using hyperimmune rabbit sera demonstrate efficient binding of recombinant antigens to microwells, used as a positive control for ELISA assays assessing primate sera reactivity (Fig. 6). Data above represents the average OD from a single experiment with serum diluted 1:1,000, tested in quadruplicate, with the results of assays with no antigen subtracted as a blank; error bars indicate standard deviations.
