# Supplementary material for: Analysis of the Mycoplasma genitalium MgpB Adhesin to Predict Membrane Topology, Investigate Antibody Accessibility, Characterize Amino Acid Diversity, and Identify Functional and Immunogenic Epitopes
Source: PLoS One. 2015 Sep 18;10(9):e0138244. doi: 10.1371/journal.pone.0138244 (PMC4575044; doi:10.1371/journal.pone.0138244)
Supplement: S2 Fig — All available mgpB sequences were compared to the M. genitalium G37 reference strain using Clustal Omega. Alignments were manually adjusted to evaluate the number of synonymous and non-synonymous mutations, with the predicted amino acid sequence and variants shown below. Nucleotides that differ from the G37 type strain are highlighted in gray if they are predicted to result in a silent, synonymous mutation; non-synonymous mutations are highlighted in yellow, green, aqua, pink, or blue. Indels are also noted with deletions (compared to G37) highlighted in black, and insertions (again compared to G37) noted in red. The dominant residue found in the majority of sequences at each amino acid position was included in a theoretical consensus sequence, shown in Fasta format at the top. Also noted is the variation within the unique amino acid sequences identified using the SAPS. This analysis was conducted on sequences spanning each conserved and variable region within the mgpB expression site; each section notes the total number of sequences included in the analysis. (PDF) [file pone.0138244.s006.pdf]

>MgpB_Consensus

```
MHQPKKRLAKKSWAFLTAALTLGVITGVGGYFLFNQNKQRSSVSNFAYQPKQLSVKHQQAVDETLTPWTWNNNNFSSLKITGENPGSFGLVRSQNDNLNISSVTKNVSDDNLKYLNAVEKYL
DGQQNFAIRRYDNNGRALYDINLAKMENPSTVQRGLNGEPIFDPFKGFGLTGNAPTDWNEIKGKVPVEVVQSPHSPNLYFVLLVPKVVLEYHNLNNQVVKESLEVEATSSFDPTQRLQKDSP
VKDSNKDSEKLSEAMSSMSSGGATSTRKALKIEVEKGSKVNQGELQSNDFAKKPLKHKSGSNVKLDASGEFAGDKAWKPVLKTDEIAKEKGMGATVVSFYDAPYSENHTAFGLVDHIDPKKM
VENYPPSWKTPKWNHHGIWDYNARNLLLQTTGFFNPRRHPEWFDEGQAKADNTSPGFKVGESDHKKDGFKDSSSSSSPIALPFEAYFANIGNMVAIGNSVFIFGGNGHATKMFTTNPLSIGV
FRIKYTDNFSKSSVTGWPYAVLFGGLINPQTNGLKDLPLGTNRWFEYVPRMAVSGVKWVGNQLVLAGTLTMGDTATVPRLKYDQLEKHLNLVAQGQGLLREDLQIFTPYGWANRPDIPVGAW
LQDEMGSKFGPHYFLNNPDIQDNVNNDTVEALISSYKNTDKLKHVYPYRYSGLYAWQLFNWSNKLTNTPLSANFVNENSYAPNSLFAAILNEDLLTGLSDKIVYGKENEFAENEADRFNQLL
SLNPSPNTNWARYLNVVQRFTTGPNLDGSTFDQFLDFLPWIGNGKPFSNSHTASLSVSSNTPLPTFSNINVGVKSMITKHLNKENTRWVFTPNSSPDIWTGAGYRKQGNNNGIPLTSVLPSS
NSSTPFNPNSDENQVTPSGGSSKKTTYDALPNSISPTSDWINALTFTNKNNPQRNQLLLRALLGTIPVLINKSGDSNDQFNKDSEQKWDKTETNEGNLPGFGEVNGLYNAALLHTYGFFGTN
TNSTDPKIGFKADSSSSSSSTLVGSGLNWTSQDVGNLVVINDTSFGFQLGGWFITFTDFIRPRTGYLGITLSSLQDQTIIWADQPWTSFKGSYLDSDGTPKSLWDPTALKSLPNSSTTSDTN
PTLSPSFLYFQPNKVKAYQTTNTYNRLIEPDKWNSTSDLTNMTNLLKLLTTKNIKQKLGKDTAQSQGNNGGGVSQTINTITTTGNISEGLKEETSIQAETLKKFFDSKQNNKSEIGIGDSTF
TKMDGKLTGVVSTPLVNLINGQGATSDSDTEKISFKPGNQIDFNRLFTLPVTELFDPNTMFVYDQYVPLLVNLPSGFDQASIRLKVISYSVENQTLGVRLEFKDPQTQQFIPVLNASSTGPQ
TVFQPFNQWADYVLPLIVTVPIVVIILSVTLGLTIGIPMHRNKKALQAGFDLSNKKVDVLTKAVGSVFKEIINRTGISNAPKKLKQATPTKPTPKTPPKPPVKQ
```

S2 Figure. ***mgpB*** **sequence alignments for the evaluation of diversity within and among strains**

All available *mgpB* sequences were compared to the *M. genitalium* G37 reference strain using Clustal Omega. Alignments were manually adjusted to evaluate the number of synonymous and non-synonymous mutations, with the predicted amino acid sequence and variants shown below. Nucleotides that differ from the G37 type strain are highlighted in gray if they are predicted to result in a silent, synonymous mutation; non-synonymous mutations are highlighted in yellow, green, aqua, pink, or blue. Indels are also noted with deletions (compared to G37) highlighted in black, and insertions (again compared to G37) noted in red. The dominant residue found in the majority of sequences at each amino acid position was included in a theoretical consensus sequence, shown in Fasta format at the top. Also noted is the variation within the unique amino acid sequences identified using the SAPS. This analysis was conducted on sequences spanning each conserved and variable region within the *mgpB* expression site; each section notes the total number of sequences included in the analysis.

Conserved N-terminus (bp 1-576, aa 1-192)

34 Sequences Analyzed

**G37**	**1**	**ATG**	**CAC**	**CAA**	**CCA**	**AAG**	**AAA**	**AGA**	**CTG**	**GCT**	**AAG**	**AAG**	**TCT**	**TGA**	**GCC**	**TTT**	**CTA**	**ACC**	**GCT**	**GCA**	**CTT**	**ACC**	**CTT**	**GGG**	**GTT**	**ATA**	**ACA**	**GGT**	**GTA**	**GGT**	**GGT**
M30		ATG	CAC	CAA	CCA	AAG	AAA	AGA	CTG	GCT	AAG	AAG	TCT	TGA	GCC	TTT	CTA	ACC	GCT	GCA	CTT	ACC	CTT	GGG	GTT	ATA	ACA	GGT	GTA	GGT	GGT
M2282		ATG	CAC	CAA	CCA	AAG	AAA	AGA	CTG	GCT	AAG	AAG	TCT	TGA	GCC	TTT	CTA	ACC	GCT	GCA	CTT	ACC	CTT	GGG	GTT	ATA	ACA	GGT	GTA	GGT	GGT
M2288		ATG	CAC	CAA	CCA	AAG	AAA	AGA	CTG	GCT	AAG	AAG	TCT	TGA	GCC	TTT	CTA	ACC	GCT	GCA	CTT	ACC	CTT	GGG	GTT	ATA	ACA	GGT	GTA	GGT	GGT
M2300		ATG	CAC	CAA	CCA	AAG	AAA	AGA	CTG	GCT	AAG	AAG	TCT	TGA	GCC	TTT	CTA	ACC	GCT	GCA	CTT	ACC	CTT	GGG	GTT	ATA	ACA	GGT	GTA	GGT	GGT
M2321		ATG	CAC	CAA	CCA	AAG	AAA	AGA	CTG	GCT	AAG	AAG	TCT	TGA	GCC	TTT	CTA	ACC	GCT	GCA	CTT	ACC	CTT	GGG	GTT	ATA	ACA	GGT	GTA	GGT	GGT
M2341		ATG	CAC	CAA	CCA	AAG	AAA	AGA	CTG	GCT	AAG	AAG	TCT	TGA	GCC	TTT	CTA	ACC	GCT	GCA	CTT	ACC	CTT	GGG	GTT	ATA	ACA	GGT	GTA	GGT	GGT
M6257		ATG	CAC	CAA	CCA	AAG	AAA	AGA	CTG	GCT	AAG	AAG	TCT	TGA	GCC	TTT	CTA	ACC	GCT	GCA	CTT	ACC	CTT	GGG	GTT	ATA	ACA	GGT	GTA	GGT	GGT
M6280		ATG	CAC	CAA	CCA	AAG	AAA	AGA	CTG	GCT	AAG	AAG	TCT	TGA	GCC	TTT	CTA	ACC	GCT	GCA	CTT	ACC	CTT	GGG	GTT	ATA	ACA	GGT	GTA	GGT	GGT
M6282		ATG	CAC	CAA	CCA	AAG	AAA	AGA	CTG	GCT	AAG	AAG	TCT	TGA	GCC	TTT	CTA	ACC	GCT	GCA	CTT	ACC	CTT	GGG	GTT	ATA	ACA	GGT	GTA	GGT	GGT
M6283		ATG	CAC	CAA	CCA	AAG	AAA	AGA	CTG	GCT	AAG	AAG	TCT	TGA	GCC	TTT	CTA	ACC	GCT	GCA	CTT	ACC	CTT	GGG	GTT	ATA	ACA	GGT	GTA	GGT	GGT
M6284		ATG	CAC	CAA	CCA	AAG	AAA	AGA	CTG	GCT	AAG	AAG	TCT	TGA	GCC	TTT	CTA	ACC	GCT	GCA	CTT	ACC	CTT	GGG	GTT	ATA	ACA	GGT	GTG	GGT	GGT
M6285		ATG	CAC	CAA	CCA	AAG	AAA	AGA	CTG	GCT	AAG	AAG	TCT	TGA	GCC	TTT	CTA	ACC	GCT	GCA	CTT	ACC	CTT	GGG	GTT	ATA	ACA	GGT	GTA	GGT	GGT
6286		ATG	CAC	CAA	CCA	AAG	AAA	AGA	CTG	GCT	AAG	AAG	TCT	TGA	GCC	TTT	CTA	ACC	GCT	GCA	CTT	ACC	CTT	GGG	GTT	ATA	ACA	GGT	GTA	GGT	GGT
M6320		ATG	CAC	CAA	CCA	AAG	AAA	AGA	CTG	GCT	AAG	AAG	TCT	TGA	GCC	TTT	CTA	ACC	GCT	GCA	CTT	ACC	CTT	GGG	GTT	ATA	ACA	GGT	GTA	GGT	GGT
64.0		ATG	CAC	CAA	CCA	AAG	AAA	AGA	CTG	GCT	AAG	AAG	TCT	TGA	GCC	TTT	CTA	ACC	GCT	GCA	CTT	ACC	CTT	GGG	GTT	ATA	ACA	GGT	GTA	GGT	GGT
64.1		ATG	CAC	CAA	CCA	AAG	AAA	AGA	CTG	GCT	AAG	AAG	TCT	TGA	GCC	TTT	CTA	ACC	GCT	GCA	CTT	ACC	CTT	GGG	GTT	ATA	ACA	GGT	GTA	GGT	GGT
199.0		ATG	CAC	CAA	CCA	AAG	AAA	AGA	CTG	GCT	AAG	AAG	TCT	TGA	GCC	TTT	CTA	ACC	GCT	GCA	CTT	ACC	CTT	GGG	GTT	ATA	ACA	GGT	GTA	GGT	GGT
199.1		ATG	CAC	CAA	CCA	AAG	AAA	AGA	CTG	GCT	AAG	AAG	TCT	TGA	GCC	TTT	CTA	ACC	GCT	GCA	CTT	ACC	CTT	GGG	GTT	ATA	ACA	GGT	GTA	GGT	GGT
KOR10046		ATG	CAC	CAA	CCA	AAG	AAA	AGA	CTG	GCT	AAG	AAG	TCT	TGA	GCC	TTT	CTA	ACC	GCT	GCA	CTT	ACC	CTT	GGG	GTT	ATA	ACA	GGT	GTA	GGT	GGT
KOR10081		ATG	CAC	CAA	CCA	AAG	AAA	AGA	CTG	GCT	AAG	AAG	TCT	TGA	GCC	TTT	CTA	ACC	GCT	GCA	CTT	ACC	CTT	GGG	GTT	ATA	ACA	GGT	GTA	GGT	GGT
KOR10089		ATG	CAC	CAA	CCA	AAG	AAA	AGA	CTG	GCT	AAG	AAG	TCT	TGA	GCC	TTT	CTA	ACC	GCT	GCA	CTT	ACC	CTT	GGG	GTT	GTA	ACA	GGT	GTA	GGT	GGT
KOR10090		ATG	CAC	CAA	CCA	AAG	AAA	AGA	CTG	GCT	AAG	AAG	TCT	TGA	GCC	TTT	CTA	ACC	GCT	GCA	CTT	ACC	CTT	GGG	GTT	ATA	ACA	GGT	GTA	GGT	GGT
KOR10114		ATG	CAC	CAA	CCA	AAG	AAA	AGA	CTG	GCT	AAG	AAG	TCT	TGA	GCC	TTT	CTA	ACC	GCT	GCA	CTT	ACC	CTT	GGG	GTT	ATA	ACA	GGT	GTA	GGT	GGT
KOR10139		ATG	CAC	CAA	CCA	AAG	AAA	AGA	CTG	GCT	AAG	AAG	TCT	TGA	GCC	TTT	CTA	ACC	GCT	GCA	CTT	ACC	CTT	GGG	GTT	GTA	ACA	GGT	GTA	GGT	GGT
KOR10163		ATG	CAC	CAA	CCA	AAG	AAA	AGA	CTG	GCT	AAG	AAG	TCT	TGA	GCC	TTT	CTA	ACC	GCT	GCA	CTT	ACC	CTT	GGG	GTT	ATA	ACA	GGT	GTA	GGT	GGT
KOR10177		ATG	CAC	CAA	CCA	AAG	AAA	AGA	CTG	GCT	AAG	AAG	TCT	TGA	GCC	TTT	CTA	ACC	GCT	GCA	CTT	ACC	CTT	GGG	GTT	GTA	ACA	GGT	GTA	GGT	GGT
KOR10274		ATG	CAC	CAA	CCA	AAG	AAA	AGA	CTG	GCT	AAG	AAG	TCT	TGA	GCC	TTT	CTA	ACC	GCT	GCA	CTT	ACC	CTT	GGG	GTT	ATA	ACA	GGT	GTA	GGT	GGT
MEGA10366		ATG	CAC	CAA	CCA	AAG	AAA	AGA	CTG	GCT	AAG	AAG	TCT	TGA	GCC	TTT	CTA	ACC	GCT	GCA	CTT	ACC	CTT	GGG	GTT	GTA	ACA	GGT	GTA	GGT	GGT
MEGA10378		ATG	CAC	CAA	CCA	AAG	AAA	AGA	CTG	GCT	AAG	AAG	TCT	TGA	GCC	TTT	CTA	ACC	GCT	GCA	CTT	ACC	CTT	GGG	GTT	ATA	ACA	GGT	GTA	GGT	GGT
MEGA10467		ATG	CAC	CAA	CCA	AAG	AAA	AGA	CTG	GCT	AAG	AAG	TCT	TGA	GCC	TTT	CTA	ACC	GCT	GCA	CTT	ACC	CTT	GGG	GTT	ATA	ACA	GGT	GTA	GGT	GGT
MEGA10477		ATG	CAC	CAA	CCA	AAG	AAA	AGA	CTG	GCT	AAG	AAG	TCT	TGA	GCC	TTT	CTA	ACC	GCT	GCA	CTT	ACC	CTT	GGG	GTT	ATA	ACA	GGT	GTA	GGT	GGT
Sea1		ATG	CAC	CAA	CCA	AAG	AAA	AGA	CTG	GCT	AAG	AAG	TCT	TGA	GCC	TTT	CTA	ACC	GCT	GCA	CTT	ACC	CTT	GGG	GTT	ATA	ACA	GGT	GTA	GGT	GGT
Sea2		ATG	CAC	CAA	CCA	AAG	AAA	AGA	CTG	GCT	AAG	AAG	TCT	TGA	GCC	TTT	CTA	ACC	GCT	GCA	CTT	ACC	CTT	GGG	GTT	ATA	ACA	GGT	GTA	GGT	GGT
		***	***	***	***	***	***	***	***	***	***	***	***	***	***	***	***	***	***	***	***	***	***	***	***	**	***	***	**	***	***
**G37**	**1**	**M**	**H**	**Q**	**P**	**K**	**K**	**R**	**L**	**A**	**K**	**K**	**S**	**W**	**A**	**F**	**L**	**T**	**A**	**A**	**L**	**T**	**L**	**G**	**V**	**I**	**T**	**G**	**V**	**G**	**G**
																										**V**					

**Consensus**	**1**	**M**	**H**	**Q**	**P**	**K**	**K**	**R**	**L**	**A**	**K**	**K**	**S**	**W**	**A**	**F**	**L**	**T**	**A**	**A**	**L**	**T**	**L**	**G**	**V**	**I**	**T**	**G**	**V**	**G**	**G**

**G37**	**91**	**TAT TTT CTC TTT AAC CAA AAT AAG CAA CGT AGT AGC GTG AGC AAC TTT GCT TAC CAA CCC AAG CAG TTA AGT GTT AAA CAC CAA CAA GCA**
M30		TAT TTT CTC TTT AAC CAA AAT AAG CAA CGT AGT AGC GTG AGC AAC TTT GCT TAC CAA CCC AAG CAG TTA AGT GTT AAA CAC CAA CAA GCA
M2282		TAT TTT CTC TTT AAC CAA AAT AAG CAA CGT AGT AGC GTG AGC AAC TTT GCT TAC CAA CCC AAG CAG TTA AGT GTT AAA CAC CAA CAA GCA
M2288		TAT TTT CTC TTT AAC CAA AAT AAG CAA CGT AGT AGC GTG AGC AAC TTT GCT TAC CAA CCC AAG CAG TTA AGT GTT AAA CAC CAA CAA GCA
M2300		TAT TTT CTC TTT AAC CAA AAT AAG CAA CGT AGT AGC GTG AGC AAC TTT GCT TAC CAA CCC AAG CAG TTA AGT GTT AAA CAC CAA CAA GCA
M2321		TAT TTT CTC TTT AAC CAA AAT AAG CAA CGT AGT AGC GTG AGC AAC TTT GCT TAC CAA CCC AAG CAG TTA AGT GTT AAA CAC CAA CAA GCA
M2341		TAT TTT CTC TTT AAC CAA AAT AAG CAA CGT AGT AGC GTG AGC AAC TTT GCT TAC CAA CCC AAG CAG TTA AGT GTT AAA CAC CAA CAA GCA
M6257		TAT TTT CTC TTT AAC CAA AAT AAG CAA CGT AGT AGC GTG AGC AAC TTT GCT TAC CAA CCC AAG CAG TTA AGT GTT AAA CAC CAA CAA GCA
M6280		TAT TTT CTC TTT AAC CAA AAT AAG CAA CGT AGT AGC GTG AGC AAC TTT GCT TAC CAA CCC AAG CAG TTA AGT GTT AAA CAC CAA CAA GCA
M6282		TAT TTT CTC TTT AAC CAA AAT AAG CGA CGT AGT AGC GTG AGC AAC TTT GCT TAC CAA CCC AAG CAG TTA AGT GTT AAA CAC CAA CAA GCA
M6283		TAT TTT CTC TTT AAC CAA AAT AAG CAA CGT AGT AGC GTG AGC AAC TTT GCT TAC CAA CCC AAG CAG TTA AGT GTT AAA CAC CAA CAA GCA
M6284		TAT TTT CTC TTT AAC CAA AAT AAG CAA CGT AGT AGC GTG AGC AAC TTT GCT TAC CAA CCC AAG CAG TTA AGT GTT AAA CAC CAA CAA GCA
M6285		TAT TTT CTC TTT AAC CAA AAT AAG CAA CGT AGT AGC GTG AGC AAC TTT GCT TAC CAA CCC AAG CAG TTA AGT GTT AAA CAC CAA CAA GCA
6286		TAT TTT CTC TTT AAC CAA AAT AAG CAA CGT AGT AGC GTG AGC AAC TTT GCT TAC CAA CCC AAG CAG TCA AGT GTT AAA CAC CAA CAA GCA
M6320		TAT TTT CTC TTT AAC CAA AAT AAG CAA CGT AGT AGC GTG AGC AAC TTT GCT TAC CAA CCC AAG CAG TTA AGT GTT AAA CAC CAA CAA GCA
64.0		TAT TTT CTC TTT AAC CAA AAT AAG CAA CGT AGT AGC GTG AGC AAC TTT GCT TAC CAA CCC AAG CAG TTA AGT GTT AAA CAC CAA CAA GCA
64.1		TAT TTT CTC TTT AAC CAA AAT AAG CAA CGT AGT AGC GTG AGC AAC TTT GCT TAC CAA CCC AAG CAG TTA AGT GTT AAA CAC CAA CAA GCA
199.0		TAT TTT CTC TTT AAC CAA AAT AAG CAA CGT AGT AGC GTG AGC AAC TTT GCT TAC CAA CCC AAG CAG TTA AGT GTT AAA CAC CAA CAA GCA
199.1		TAT TTT CTC TTT AAC CAA AAT AAG CAA CGT AGT AGC GTG AGC AAC TTT GCT TAC CAA CCC AAG CAG TTA AGT GTT AAA CAC CAA CAA GCA
KOR10046		TAT TTT CTC TTT AAC CAA AAT AAG CAA CGT AGT AGC GTG AGC AAC TTT GCT TAC CAA CCC AAG CAG TTA AGT GTT AAA CAC CAA CAA GCA
KOR10081		TAT TTT CTC TTT AAC CAA AAT AAG CAA CGT AGT AGC GTG AGC AAC TTT GCT TAC CAA CCC AAG CAG TTA AGT GTT AAA CAC CAA CAA GCA
KOR10089		TAT TTT CTC TTT AAC CAA AAT AAG CAA CGT AGT AGC GTG AGC AAC TTT GCT TAC CAA CCC AAG CAG TTA AGT GTT AAA CAC CAA CAA GCA
KOR10090		TAT TTT CTC TTT AAC CAA AAT AAG CAA CGT AGT AGC GTG AGC AAC TTT GCT TAC CAA CCC AAG CAG TTA AGT GTT AAA CAC CAA CAA GCA
KOR10114		TAT TTT CTC TTT AAC CAA AAT AAG CAA CGT AGT AGC ATG AGC AAC TTT GCT TAC CAA CCC AAG CAG TTA AGT GTT AAA CAC CAA CAA GCA
KOR10139		TAT TTT CTC TTT AAC CAA AAT AAG CAA CGT AGT AGC GTG AGC AAC TTT GCT TAC CAA CCC AAG CAG TTA AGT GTT AAA CAC CAA CAA GCA
KOR10163		TAT TTT CTC TTT AAC CAA AAT AAG CAA CGT AGT AGC GTG AGC AAC TTT GCT TAC CAA CCC AAG CAG TTA AGT GTT AAA CAC CAA CAA GCA
KOR10177		TAT TTT CTC TTT AAC CAA AAT AAG CAA CGT AGT AGC GTG AGC AAC TTT GCT TAC CAA CCC AAG CAG TTA AGT GTT AAA CAC CAA CAA GCA
KOR10274		TAT TTT CTC TTT AAC CAA AAT AAG CAA CGT AGT AGC GTG AGC AAC TTT GCT TAC CAA CCC AAG CAG TTA AGT GTT AAA CAC CAA CAA GCA
MEGA10366		TAT TTT CTC TTT AAC CAA AAT AAG CAA CGT AGT AGC GTG AGC AAC TTT GCT TAC CAA CCC AAG CAG TTA AGT GTT AAA CAC CAA CAA GCA
MEGA10378		TAT TTT CTC TTT AAC CAA AAT AAG CAA CGT AGT AGC GTG AGC AAC TTT GCT TAC CAA CCC AAG CAG TTA AGT GTT AAA CAC CAA CAA GCA
MEGA10467		TAT TTT CTC TTT AAC CAA AAT AAG CAA CGT AGT AGC GTG AGC AAC TTT GCT TAC CAA CCC AAG CAG TTA AGT GTT AAA CAC CAA CAA GCA
MEGA10477		TAT TTT CTC TTT AAC CAA AAT AAG CAA CGT AGT AGC GTG AGC AAC TTT GCT TAC CAA CCC AAG CAG TTA AGT GTT AAA CAC CAA CAA GCA
Sea1		TAT TTT CTC TTT AAC CAA AAT AAG CAA CGT AGT AGC GTG AGC AAC TTT GCT TAC CAA CCC AAG CAG TTA AGT GTT AAA CAC CAA CAA GCA
Sea2		TAT TTT CTC TTT AAC CAA AAT AAG CAA CGT AGT AGC GTG AGC AAC TTT GCT TAC CAA CCC AAG CAG TTA AGT GTT AAA CAC CAA CAA GCA
		*** *** *** *** *** *** *** *** * * *** *** *** ** *** *** *** *** *** *** *** *** *** * * *** *** *** *** *** *** ***
**G37**	**31**	**Y F L F N Q N K Q R S S V S N F A Y Q P K Q L S V K H Q Q A**
		**R** (position 9), **M** (position 13), **S** (position 23)
**Consensus**	**31**	**Y F L F N Q N K Q R S S V S N F A Y Q P K Q L S V K H Q Q A**

**G37**	**181**	**GTT**	**GAT**	**GAA**	**ACC**	**TTA**	**ACC**	**CCT**	**TGG**	**ACT**	**TGA**	**AAC**	**AAT**	**AAC**	**AAC**	**TTC**	**TCT**	**TCA**	**CTA**	**AAG**	**ATT**	**ACT**	**GGA**	**GAG**	**AAC**	**CCA**	**GGA**	**TCA**	**TTT**	**GGA**	**TTA**
M30		GTT	GAT	GAA	ACC	TTA	ACC	CCT	TGG	ACT	TGA	AAC	AAT	AAC	AAC	TTC	TCT	TCA	CTA	AAG	ATT	ACT	GGA	GAG	AAC	CCA	GGA	TCA	TTT	GGA	TTA
M2282		GTT	GAT	GAA	ACC	TTA	ACC	CCT	TGG	ACT	TGA	AAC	AAT	AAC	AAC	TTC	TCT	TCA	CTA	AAG	ATT	ACT	GGA	GAG	AAC	CCA	GGA	TCA	TTT	GGA	TTA
M2288		GTT	GAT	GAA	ACC	TTA	ACC	CCT	TGG	ACT	TGA	AAC	AAT	AAC	AAC	TTC	TCT	TCA	CTA	AAG	ATT	ACT	GGA	GAG	AAC	CCA	GGA	TCA	TTT	GGA	TTA
M2300		GTT	GAT	GAA	ACC	TTA	ACC	CCT	TGG	ACT	TGA	AAC	AAT	AAC	AAC	TTC	TCT	TCA	CTA	AAG	ATT	ACT	GGA	GAG	AAC	CCA	GGA	TCA	TTT	GGA	TTA
M2321		GTT	GAT	GAA	ACA	TTA	ACC	CCT	TGG	ACT	TGA	AAC	AAT	AAC	AAC	TTC	TCT	TCA	CTA	AAG	ATT	ACT	GGA	GAG	AAC	CCA	GGA	TCA	TTT	GGA	TTA
M2341		GTT	GAT	GAA	ACC	TTA	ACC	CCT	TGG	ACT	TGA	AAC	AAT	AAC	AAC	TTC	TCT	TCA	CTA	AAG	ATT	ACT	GGA	GAG	AAC	CCA	GGA	TCA	TTT	GGA	TTA
M6257		GTT	GAT	GAA	ACC	TTA	ACC	CCT	TGG	ACT	TGA	AAC	AAT	AAC	AAC	TTC	TCT	TCA	CTG	AAG	ATT	ACT	GGA	GAG	AAC	CCA	GGA	TCA	TTT	GGA	TTA
M6280		GTT	GAT	GAA	ACC	TTA	ACC	CCT	TGG	ACT	TGA	AAC	AAT	AAC	AAC	TTC	TCT	TCA	CTG	AAG	ATT	ACT	GGA	GAG	AAC	CCA	GGA	TCA	TTT	GGA	CTA
M6282		GTT	GAT	GAA	ACC	TTA	ACC	CCT	TGG	ACT	TGA	AAC	AAT	AAC	AAC	TTC	TCT	TCA	CTG	AAG	ATT	ACT	GGA	GAG	AAC	CCA	GGA	TCA	TTT	GGA	CTA
M6283		GTT	GAT	GAA	ACC	TTA	ACC	CCT	TGG	ACT	TGA	AAC	AAT	AAC	AAC	TTC	TCT	TCA	CTG	AAG	ATT	ACT	GGA	GAG	AAC	CCA	GGA	TCA	TTT	GGA	CTA
M6284		GTT	GAT	GAA	ACC	TTA	ACC	CCT	TGG	ACT	TGA	AAC	AAT	AAC	AAC	TTC	TCT	TCA	CTA	AAG	ATT	ACT	GGA	GAG	AAC	CCA	GGA	TCA	TTT	GGA	TTA
M6285		GTT	GAT	GAA	ACC	TTA	ACC	CCT	TGG	ACT	TGA	AAC	AAT	AAC	AAC	TTC	TCT	TCA	CTA	AAG	ATT	ACT	GGA	GAG	AAC	CCA	GGA	TCA	TTT	GGA	TTA
6286		GTT	GAT	GAA	ACC	TTA	ACC	CCT	TGG	ACT	TGA	AAC	AAT	AAC	AAC	TTC	TCT	TCA	CTG	AAG	ATT	ACT	GGA	GAG	AAC	CCA	GGA	TCA	TTT	GGA	CTA
M6320		GTT	GAT	GAA	ACC	TTA	ACC	CCT	TGG	ACT	TGA	AAC	AAT	AAC	AAC	TTC	TCT	TCA	CTA	AAG	ATT	ACT	GGA	GAG	AAC	CCA	GGA	TCA	TTT	GGA	TTA
64.0		GTT	GAT	GAA	ACC	TTA	ACC	CCT	TGG	ACT	TGA	AAC	AAT	AAC	AAC	TTC	TCT	TCA	CTG	AAG	ATT	ACT	GGA	GAG	AAC	CCA	GGA	TCA	TTT	GGA	CTA
64.1		GTT	GAT	GAA	ACC	TTA	ACC	CCT	TGG	ACT	TGA	AAC	AAT	AAC	AAC	TTC	TCT	TCA	CTG	AAG	ATT	ACT	GGA	GAG	AAC	CCA	GGA	TCA	TTT	GGA	CTA
199.0		GTT	GAT	GAA	ACC	TTA	ACC	CCT	TGG	ACT	TGA	AAC	AAT	AAC	AAC	TTC	TCT	TCA	CTA	AAG	ATT	ACT	GGA	GAG	AAC	CCA	GGA	TCA	TTT	GGA	TTA
199.1		GTT	GAT	GAA	ACC	TTA	ACC	CCT	TGG	ACT	TGA	AAC	AAT	AAC	AAC	TTC	TCT	TCA	CTA	AAG	ATT	ACT	GGA	GAG	AAC	CCA	GGA	TCA	TTT	GGA	TTA
KOR10046		GTT	GAT	GAA	ACC	TTA	ACC	CCT	TGG	ACT	TGA	AAC	AAT	AAC	AAC	TTC	TCT	TCA	CTA	AAG	ATT	ACT	GGA	GAG	AAC	CCA	GGA	TCA	TTT	GGA	TTA
KOR10081		GTT	GAT	GAA	ACC	TTA	ACC	CCT	TGG	ACT	TGA	AAC	AAT	AAC	AAC	TTC	TCT	TCA	CTA	AAG	ATT	ACT	GGA	GAG	AAC	CCA	GGA	TCA	TTT	GGA	TTA
KOR10089		GTT	GAT	GAA	ACA	TTA	ACC	CCT	TGG	ACT	TGA	AAC	AAT	AAC	AAC	TTC	TCT	TCA	CTG	AAG	ATT	ACT	GGA	GAG	AAC	CCA	GGA	TCA	TTT	GGA	CTA
KOR10090		GTT	GAT	GAA	ACA	TTA	ACC	CCT	TGG	ACT	TGA	AAC	AAT	AAC	AAC	TTC	TCT	TCA	CTA	AAG	ATT	ACT	GGA	GAG	AAC	CCA	GGA	TCA	TTT	GGA	TTA
KOR10114		GTT	GAT	GAA	ACA	TTA	ACC	CCT	TGG	ACT	TGA	AAC	AAT	AAC	AAC	TTC	TCT	TCA	CTA	AAG	ATT	ACT	GGA	GAG	AAC	CCA	GGA	TCA	TTT	GGA	TTA
KOR10139		GTT	GAT	GAA	ACA	TTA	ACC	CCT	TGG	ACT	TGA	AAC	AAT	AAC	AAC	TTC	TCT	TCA	CTG	AAG	ATT	ACT	GGA	GAG	AAC	CCA	GGA	TCA	TTT	GGA	CTA
KOR10163		GTT	GAT	GAA	ACC	TTA	ACC	CCT	TGG	ACT	TGA	AAC	AAT	AAC	AAC	TTC	TCT	TCA	CTA	AAG	ATT	ACT	GGA	GAG	AAC	CCA	GGA	TCA	TTT	GGA	TTA
KOR10177		GTT	GAT	GAA	ACA	TTA	ACC	CCT	TGG	ACT	TGA	AAC	AAT	AAC	AAC	TTC	TCT	TCA	CTG	AAG	ATT	ACT	GGA	GAG	AAC	CCA	GGA	TCA	TTT	GGA	TTA
KOR10274		GTT	GAT	GAA	ACC	TTA	ACC	CCT	TGG	ACT	TGA	AAC	AAT	AAC	AAC	TTC	TCT	TCA	CTA	AAG	ATT	ACT	GGA	GAG	AAC	CCA	GGA	TCA	TTT	GGA	TTA
MEGA10366		GTT	GAT	GAA	ACA	TTA	ACC	CCT	TGG	ACT	TGA	AAC	AAT	AAC	AAC	TTC	TCT	TCA	CTG	AAG	ATT	ACT	GGA	GAG	AAC	CCA	GGA	TCA	TTT	GGA	CTA
MEGA10378		GTT	GAT	GAA	ACC	TTA	ACC	CCT	TGG	ACT	TGA	AAC	AAT	AAC	AAC	TTC	TCT	TCA	CTA	AAG	ATT	ACT	GGA	GAG	AAC	CCA	GGA	TCA	TTT	GGA	TTA
MEGA10467		GTT	GAT	GAA	ACC	TTA	ACC	CCT	TGG	ACT	TGA	AAC	AAT	AAC	AAC	TTC	TCT	TCA	CTA	AAG	ATT	ACT	GGA	GAG	AAC	CCA	GGA	TCA	TTT	GGA	TTA
MEGA10477		GTT	GAT	GAA	ACC	TTA	ACC	CCT	TGG	ACT	TGA	AAC	AAT	AAC	AAC	TTC	TCT	TCA	CTG	AAG	ATT	ACT	GGA	GAG	AAC	CCA	GGA	TCA	TTT	GGA	TTA
Sea1		GTT	GAT	GAA	ACC	TTA	ACC	CCT	TGG	ACT	TGA	AAC	AAT	AAC	AAC	TTC	TCT	TCA	CTG	AAG	ATT	ACT	GGA	GAG	AAC	CCA	GGA	TCA	TTT	GGA	CTA
Sea2		GTT	GAT	GAA	ACA	TTA	ACC	CCT	TGG	ACT	TGA	AAC	AAT	AAC	AAC	TTC	TCT	TCA	CTA	AAG	ATT	ACT	GGA	GAG	AAC	CCA	GGA	TCA	TTT	GGA	TTA
		***	***	***	**	***	***	***	***	***	***	***	***	***	***	***	***	***	**	***	***	***	***	***	***	***	***	***	***	***	**
**G37**	**61**	**V**	**D**	**E**	**T**	**L**	**T**	**P**	**W**	**T**	**W**	**N**	**N**	**N**	**N**	**F**	**S**	**S**	**L**	**K**	**I**	**T**	**G**	**E**	**N**	**P**	**G**	**S**	**F**	**G**	**L**
**Consensus**	**61**	**V**	**D**	**E**	**T**	**L**	**T**	**P**	**W**	**T**	**W**	**N**	**N**	**N**	**N**	**F**	**S**	**S**	**L**	**K**	**I**	**T**	**G**	**E**	**N**	**P**	**G**	**S**	**F**	**G**	**L**

Asparagine Repeat Identified in SAPS Analysis Shown in Box I, Figure 7B **N N N N**

**G37**	**271**	**GTA**	**AGA**	**AGC**	**CAA**	**AAT**	**GAC**	**AAC**	**TTA**	**AAT**	**ATT**	**TCA**	**AGT**	**GTT**	**ACA**	**AAG**	**AAT**	**TCT**	**AGT**	**GAT**	**GAT**	**AAT**	**CTC**	**AAG**	**TAT**	**CTC**	**AAT**	**GCT**	**GTT**	**GAG**	**AAA**
M30		GTA	AGA	AGC	CAA	AAT	GAC	AAC	TTA	AAT	ATT	TCA	AGT	GTT	ACA	AAG	AAT	GTT	AGT	GAT	GAT	AAT	CTC	AAG	TAT	CTC	AAT	GCT	GTT	GAG	AAA
M2282		GTA	AGA	AGC	CAA	AAT	GAC	AAC	TTA	AAT	ATT	TCA	AGT	GTT	ACA	AAG	AAT	GTT	AGT	GAT	GAT	AAT	CTC	AAG	TAT	CTC	AAT	GAT	GTT	GAG	AAA
M2288		GTA	AGA	AGC	CAA	AAT	GAC	AAC	TTA	AAT	ATT	TCA	AGT	GTT	ACA	AAG	AAT	CCT	AAT	GAT	GAT	AAT	CTC	GAG	TAT	CTC	AAT	GCT	GTT	GAG	AAA
M2300		GTA	AGA	AGC	CAA	AAT	GAC	AAC	TTA	AAT	ATT	TCA	AGT	GTT	ACA	AAG	AAT	GTT	AGT	TAT	GAT	AAT	CTC	AAG	TAT	CTC	AAT	GCT	GTT	GAG	AAA
M2321		GTA	AGA	AGC	CAA	AAT	GAC	AAC	TTA	AAC	ATC	GCA	AGT	GTT	ACA	AAG	AAT	GGT	AGT	GAT	GAT	AAT	CTC	AAG	TAT	CTC	AAT	GCT	GTT	GAG	AAA
M2341		GTA	AGA	AGC	CAA	AAT	GAC	AAC	TTA	AAT	ATT	TCA	AGT	GTT	ACA	AAG	AAT	GTT	AGT	GAT	GAT	AAT	CTC	AAG	TAT	CTC	AAT	GCT	GTT	GAG	AAA
M6257		GTA	AGA	AGC	CAA	AAT	GCG	AAC	TTA	AAT	ATC	GCA	AGT	GTT	CAA	AAG	CAA	TCA	GGT	GAT	GAT	AAT	CTC	AAG	TAT	CTT	AAT	GCT	GTT	GAG	AAA
M6280		GTA	AGA	AGC	CAA	AAT	GAG	AAC	TTA	AAC	ATC	GCA	AGT	GTT	ACA	AAG	AAT	GAT	AGT	GAT	GAT	AAT	CTC	AAG	TAT	CTT	AAT	TCT	GTT	GAG	AAA
M6282		GTA	AGA	AGC	CAA	AAT	GAG	AAC	TTA	AAC	ATC	GCA	AGT	GTT	ACA	AAG	AAT	GGT	AGT	GAT	GAT	AAT	CTC	AAG	TAT	CTT	AAT	GCT	GTT	GAG	AAG
M6283		GTA	AGA	AGC	CAA	AAT	GAG	AAC	TTA	AAC	ATC	GCA	AGT	GTT	ACA	AAG	AAT	GAT	AGT	GAT	GAT	AAT	CTC	AAG	TAT	CTT	AAT	TCT	GTT	GAG	AAA
M6284		GTA	AGA	AGC	CAA	AAT	GAC	AAC	TTA	AAT	ATT	TCA	AGT	GTT	ACA	AAG	AAT	GTT	AGT	GAT	GAT	AAT	CTC	AAG	TAT	CTC	AAT	GCT	GTT	GAG	AAA
M6285		GTA	AGA	AGC	CAA	AAT	GGC	AAC	TTA	AAT	ATT	TCA	AGT	GTT	ACA	AAG	AAT	GTT	AGT	GAT	GAT	AAT	CTC	AAG	TAT	CTC	AAT	GAT	GTT	GAG	AAA
6286		GTA	AGA	AGC	CAA	AAT	GAG	AAC	TTA	AAC	ATC	GCA	AGT	GTT	ACA	AAG	AAT	GAT	AGT	GAT	GAT	AAT	CTC	AAG	TAT	CTC	AAT	GCT	GTT	GAG	AAA
M6320		GTA	AGA	AGC	CAA	AAT	GAC	AAC	TTA	AAT	ATT	TCA	AGT	GTT	ACA	AAG	AAA	GTT	AGT	GAT	GAT	AAT	CTC	AAG	TAT	CTC	AAT	GAT	GTT	GAG	AAA
64.0		GTA	AGA	AGC	CAA	AAT	GAG	AAC	TTA	AAC	ATC	GCA	AGT	GTT	ACA	AAG	AAT	GAT	AGT	GAT	GAT	AAT	CTC	AAG	TAT	CTT	AAT	TCT	GTT	GAG	AAA
64.1		GTA	AGA	AGC	CAA	AAT	GAG	AAC	TTA	AAC	ATC	GCA	AGT	GTT	ACA	AAG	AAT	GAT	AGT	GAT	GAT	AAT	CTC	AAG	TAT	CTT	AAT	TCT	GTT	GAG	AAA
199.0		GTA	AGA	AGC	CAA	AAT	GAC	AAC	TTA	AAT	ATT	TCA	AAT	GTT	ACA	AAG	AAT	GTT	AGT	GAT	GAT	AAT	CTC	AAG	TAT	CTC	AAT	GAT	GTT	GAG	AAA
199.1		GTA	AGA	AGC	CAA	AAT	GAC	AAC	TTA	AAT	ATT	TCA	AAT	GTT	ACA	AAG	AAT	GTT	AGT	GAT	GAT	AAT	CTC	AAG	TAT	CTC	AAT	GAT	GTT	GAG	AAA
KOR10046		GTA	AGA	AGC	CAA	AAT	GAC	AAC	TTA	AAT	ATT	TCA	AGT	GTT	ACA	AAG	AAT	GTT	AGT	GAT	GAT	AAT	CTC	AAG	TAT	CTC	AAT	GCT	GTT	GAG	AAA
KOR10081		GTA	AGA	AGC	CAA	AAT	GAC	AAC	TTA	AAT	ATT	TCA	AAT	GTT	ACA	AAG	AAT	GTT	AGT	AAT	GAT	AAT	CTC	AAG	TAT	CTC	AAT	GAT	GTT	GAG	AAA
KOR10089		GTA	AGA	AGC	CAA	AAT	GAG	AAC	TTA	AAC	ATC	GCA	AGT	GTT	ACA	AAG	CAT	AGT	AGT	GAT	GAT	AAT	CTC	AAG	TAT	CTT	AAT	GCT	GTT	GAG	AAG
KOR10090		GTA	AGA	AGC	CAA	AAT	GAC	AAC	TTA	AAC	ATC	GCA	AGT	GTT	ACA	AAG	AAT	GTT	AGT	GAT	GAT	AAT	CTC	AAG	TAT	CTC	AAT	GCT	GTT	GAG	AAA
KOR10114		GTA	AGA	AGC	CAA	AAT	GAC	AAC	TTA	AAC	ATC	GCA	AGT	GCT	ACA	AAG	AAT	GTT	AAT	GAT	GAT	AAT	CTC	AAG	TAT	CTC	AAT	ACT	GTT	GAG	AAA
KOR10139		GTA	AGA	AGC	CAA	AAT	GAG	AAC	TTA	AAC	ATC	GCA	AGT	GTT	ACA	AAG	AAT	GGT	AGT	GAT	GAT	AAT	CTC	AAG	TAT	CTT	AAT	GCT	GTT	GAG	AAG
KOR10163		GTA	AGA	AGC	CAA	AAT	GAC	AAC	TTA	AAT	ATT	TCA	AGT	GTT	ACA	AAG	AAT	GTT	AGT	GAT	GAT	AAT	CTC	AAG	TAT	CTC	AAT	AAT	GTT	GAG	AAA
KOR10177		GTA	AGA	AGC	CAA	AAT	GAG	AAC	TTA	AAC	ATC	GCA	AGT	GTT	ACA	AAG	CAT	GGT	AGT	GAT	GAT	AAT	CTC	AAG	TAT	CTT	AAT	GCT	GTT	GAG	AAG
KOR10274		GTA	AGA	AGC	CAA	AAT	GAC	AAC	TTA	AAT	ATT	TCA	AGT	GTT	ACA	AAG	AAT	GTT	AGT	GAT	GAT	AAT	CTC	AAG	TAT	CTC	AAT	GCT	GTT	GAG	AAA
MEGA10366		GTA	AGA	AGC	CAA	AAT	GAG	AAC	TTA	AAC	ATC	GCA	AGT	GTT	ACA	AAG	AAT	GGT	AGT	GAT	GAT	AAT	CTC	AAG	TAT	CTT	AAT	GCT	GTT	GAG	AAG
MEGA10378		GTA	AGA	AGC	CAA	AAT	GAC	AAC	TTA	AAT	ATT	TCA	AGT	GTT	ACA	AAG	AAT	GTT	AGT	GAT	GAT	AAT	CTC	AAG	TAT	CTC	AAT	GCT	GTT	GAG	AAA
MEGA10467		GTA	AGA	AGC	CAA	AAT	GAC	AAC	TTA	AAT	ATT	TCA	AGT	GTT	ACA	AAG	AAT	GTT	AGT	GAT	GAT	AAT	CTC	AAG	TAT	CTC	AAT	GAT	GTT	GAG	AAA
MEGA10477		GTA	AGA	AGC	CAA	AAT	GAG	AAC	TTA	AAC	ATC	GCA	AGT	GTT	ACA	AAG	AAT	GAT	AGT	GAT	GAT	AAT	CTC	AAG	TAT	CTT	AAT	TCT	GTT	GAG	AAA
Sea1		GTA	AGA	AGC	CAA	AAT	GAG	AAC	TTA	AAC	ATC	GCA	AGT	GTT	ACA	AAG	AAT	GAT	AGT	GAT	GAT	AAT	CTC	AAG	TAT	CTT	AAT	TCT	GTT	GAG	AAA
Sea2		GTA	AGA	AGC	CAA	AAT	GAC	AAC	TTA	AAC	ATC	GCA	AGT	GTT	ACA	AAG	AAT	GTT	AGT	GAT	GAT	AAT	CTC	AAG	TAT	CTC	AAT	GCT	GTT	GAG	AAA
		***	***	***	***	***	*	***	***	**	**	**	* *	* *	*	***	*		*	**	***	***	***	**	***	**	***	*	***	***	**
**G37**	**91**	**V**	**R**	**S**	**Q**	**N**	**D**	**N**	**L**	**N**	**I**	**S**	**S**	**V**	**T**	**K**	**N**	**S**	**S**	**D**	**D**	**N**	**L**	**K**	**Y**	**L**	**N**	**A**	**V**	**E**	**K**
							**A**					**A**	**N**	**A**	**Q**		**Q**	**V**	**N**	**Y**				**E**				**D**			
							**E**										**K**	**P**	**G**	**N**								**S**			
							**G**										**H**	**G**										**T**			
																		**D**										**N**			
**Consensus**	**91**	**V**	**R**	**S**	**Q**	**N**	**D**	**N**	**L**	**N**	**I**	**S**	**S**	**V**	**T**	**K**	**N**	**V**	**S**	**D**	**D**	**N**	**L**	**K**	**Y**	**L**	**N**	**A**	**V**	**E**	**K**

**G37**	**361**	**TAC CTT GAT GGT CAG CAA AAC TTT GCA ATC AGA AGG TAT GAT AAC AAC GGT AGA GCT TTA TAT GAT ATT AAC TTA GCA AAA ATG GAA AAC**
M30		TAC CTT GAT GGT CAG CAA AAC TTT GCA ATC AGA AGG TAT GAT AAC AAC GGT AGA GCT TTA TAT GAT ATT AAC TTA GCA AAA ATG GAA AAC
M2282		TAC CTT GAT GGT CAG CAA AAC TTT GCA ATC AGA AGG TAT GAT AAC AAC GGT AGA GCT TTA TAT GAT ATT AAC TTA GCA AAA ATG GAA AAC
M2288		TAC CTT GAT GGT CAG CAA AAC TTT GCA ATC AGA AGG TAT GAT AAC AAC GGT AGA GCT TTA TAT GAT ATT AAC TTA GCA AAA ATG GAA AAC
M2300		TAC CTT GAT GGT CAG CAA AAC TTT GCA ATC AGA AGG TAT GAT AAC AAC GGT AGA GCT TTA TAT GAT ATT AAC TTA GCA AAA ATG GAA AAC
M2321		TAC CTT GAT GGT CAG CAA AAC TTT GCA ATC AGA AGG TAT GAT AAC AAC GGT AGA GCT TTA TAT GAT ATT AAC TTA GCA AAA ATG GAA AAC
M2341		TAC CTT GAT GGT CAG CAA AAC TTT GCA ATC AGA AGG TAT GAT AAC AAC GGT AGA GCT TTA TAT GAT ATT AAC TTA GCA AAA ATG GAA AAC
M6257		TAC CTT GAT GGT CAG CAA AAC TTT GCA ATC AGA AGG TAT GAT AAC AAC GGT AGA GCT TTA TAT GAT ATT AAC TTA GCA AAA ATG GAA AAC
M6280		TAC CTT GAT GGT CAG CAA AAC TTT GCA ATC AGA AGG TAT GAT AAC AAC GGT AGA GCT TTA TAT GAT ATT AAC TTA GCA AAA ATG GAA AAC
M6282		TAC CTT GAT GGT CAG CAA AAC TTT GCA ATC AGA AGG TAT GAT AAC AAC GGT AGA GCT TTA TAT GAT ATT AAC TTA GCA AAA ATG GAA AAC
M6283		TAC CTT GAT GGT CAG CAA AAC TTT GCA ATC AGA AGG TAT GAT AAC AAC GGT AGA GCT TTA TAT GAT ATT AAC TTA GCA AAA ATG GAA AAC
M6284		TAC CTT GAT GGT CAG CAA AAC TTT GCA ATC AGA AGG TAT GAT AAC AAC GGT AGA GCT TTA TAT GAT ATT AAC TTA GCA AAA ATG GAA AAC
M6285		TAC CTT GAT GGT CAG CAA AAC TTT GCA ATC AGA AGG TAT GAT AAC AAC GGT AGA GCT TTA TAT GAT ATT AAC TTA GCA AAA ATG GAA AAC
6286		TAC CTT GAT GGT CAG CAA AAC TTT GCA ATC AGA AGG TAT GAT AAC AAC GGT AGA GCT TTA TAT GAT ATT AAC TTA GCA AAA ATG GAA AAC
M6320		TAC CTT GAT GGT CAG CAA AAC TTT GCA ATC AGA AGG TAT GAT AAC AAC GGT AGA GCT TTA TAT GAT ATT AAC TTA GCA AAA ATG GAA AAC
64.0		TAC CTT GAT GGT CAG CAA AAC TTT GCA ATC AGA AGG TAT GAT AAC AAC GGT AGA GCT TTA TAT GAT ATT AAC TTA GCA AAA ATG GAA AAC
64.1		TAC CTT GAT GGT CAG CAA AAC TTT GCA ATC AGA AGG TAT GAT AAC AAC GGT AGA GCT TTA TAT GAT ATT AAC TTA GCA AAA ATG GAA AAC
199.0		TAC CTT GAT GGT CAG CAA AAC TTT GCA ATC AGA AGG TAT GAT AAC AAC GGT AGA GCT TTA TAT GAT ATT AAC TTA GCA AAA ATG GAA AAC
199.1		TAC CTT GAT GGT CAG CAA AAC TTT GCA ATC AGA AGG TAT GAT AAC AAC GGT AGA GCT TTA TAT GAT ATT AAC TTA GCA AAA ATG GAA AAC
KOR10046		TAC CTT GAT GGT CAG CAA AAC TTT GCA ATC AGA AGG TAT GAT AAC AAC GGT AGA GCT TTA TAT GAT ATT AAC TTA GCA AAA ATG GAA AAC
KOR10081		TAC CTT GAT GGT CAG CAA AAC TTT GCA ATC AGA AGG TAT GAT AAC AAC GGT AGA GCT TTA TAT GAT ATT AAC TTA GCA AAA ATG GAA AAC
KOR10089		TAC CTT GAT GGT CAG CAA AAC TTT GCA ATC AGA AGG TAT GAT AAC AAC GGT AGA GCT TTA TAT GAT ATT AAC TTA GCA AAA ATG GAA AAC
KOR10090		TAC CTT GAT GGT CAG CAA AAC TTT GCA ATC AGA AGG TAT GAT AAC AAC GGT AGA GCT TTA TAT GAT ATT AAC TTA GCA AAA ATG GAA AAC
KOR10114		TAC CTT GAT GGT CAG CAA AAC TTT GCA ATC AGA AGG TAT GAT AAC AAC GGT AGA GCT TTA TAT GAT ATT AAC TTA GCA AAA ATG GAA AAC
KOR10139		TAC CTT GAT GGT CAG CAA AAC TTT GCA ATC AGA AGG TAT GAT AAC AAC GGT AGA GCT TTA TAT GAT ATT AAC TTA GCA AAA ATG GAA AAC
KOR10163		TAC CTT GAT GGT CAG CAA AAC TTT GCA ATC AGA AGG TAT GAT AAC AAC GGT AGA GCT TTA TAT GAT ATT AAC TTA GCA AAA ATG GAA AAC
KOR10177		TAC CTT GAT GGT CAG CAA AAC TTT GCA ATC AGA AGG TAT GAT AAC AAC GGT AGA GCT TTA TAT GAT ATT AAC TTA GCA AAA ATG GAA AAC
KOR10274		TAC CTT GAT GGT CAG CAA AAC TTT GCA ATC AGA AGG TAT GAT AAC AAC GGT AGA GCT TTA TAT GAT ATT AAC TTA GCA AAA ATG GAA AAC
MEGA10366		TAC CTT GAT GGT CAG CAA AAC TTT GCA ATC AGA AGG TAT GAT AAC AAC GGT AGA GCT TTA TAT GAT ATT AAC TTA GCA AAA ATG GAA AAC
MEGA10378		TAC CTT GAT GGT CAG CAA AAC TTT GCA ATC AGA AGG TAT GAT AAC AAC GGT AGA GCT TTA TAT GAT ATT AAC TTA GCA AAA ATG GAA AAC
MEGA10467		TAC CTT GAT GGT CAG CAA AAC TTT GCA ATC AGA AGG TAT GAT AAC AAC GGT AGA GCT TTA TAT GAT ATT AAC TTA GCA AAA ATG GAA AAC
MEGA10477		TAC CTT GAT GGT CAG CAA AAC TTT GCA ATC AGA AGG TAT GAT AAC AAC GGT AGA GCT TTA TAT GAT ATT AAC TTA GCA AAA ATG GAA AAC
Sea1		TAC CTT GAT GAT CAG CAA AAC TTT GCA ATC AGA AGG TAT GAT AAC AAC GGT AGA GCT TTA TAT GAT ATT AAC TTA GCA AAA ATG GAA AAC
Sea2		TAC CTT GAT GGT CAG CAA AAC TTT GCA ATC AGA AGG TAT GAT AAC AAC GGT AGA GCT TTA TAT GAT ATT AAC TTA GCA AAA ATG GAA AAC
		*** *** *** * * ***
**G37**	**121**	**Y L D G Q Q N F A I R R Y D N N G R A L Y D I N L A K M E N**
		**D**

**Consensus**	**121**	**Y L D G Q Q N F A I R R Y D N N G R A L Y D I N L A K M E N**

```
G37        451  CCC TCA ACG GTG CAA AGG GGT TTA AAT GGC GAG CCT ATC TTT GAT CCT TTT AAA GGC TTT GGT TTA ACT GGT AAT GCC CCT ACT GAT TGG
M30             CCC TCA ACG GTG CAA AGG GGT TTA AAT GGC GAG CCT ATC TTT GAT CCT TTT AAA GGC TTT GGT TTA ACT GGT AAT GCC CCT ACT GAT TGG
M2282           CCC TCA ACG GTG CAA AGG GGT TTA AAT GGC GAG CCT ATC TTT GAT CCT TTT AAA GGC TTT GGT TTA ACT GGT AAT GCC CCT ACT GAT TGG
M2288           CCC TCA ACG GTG CAA AGG GGT TTA AAT GGC GAG CCT ATC TTT GAT CCT TTT AAA GGC TTT GGT CTA ACT GGT AAT GCC CCT ACT GAT TGG
M2300           CCC TCA ACG GTG CAA AGG GGT TTA AAT GGC GAG CCT ATC TTT GAT CCT TTT AAA GGC TTT GGT TTA ACT GGT AAT GCC CCT AAT GAT TGG
M2321           CCC TCA ACG GTG CAA AGG GGT TTA AAT GGC GAG CCT ATC TTT GAT CCT TTT AAA GGC TTT GGT TTA ACT GGT AAT GCT CCT ACT GAT TGG
M2341           CCC TCA ACG GTG CAA AGG GGT TTA AAT GGC GAG CCT ATC TTT GAT CCT TTT AAA GGC TTT GGT TTA ACT GGT AAT GCC CCT ACT GAT TGG
M6257           CCC TCA ACG GTG CAA AGG GGT TTA AAT GGC GAG CCT ATC TTT GAT CCT TTT AAA GGC TTT GGT TTA ACT GGT AAT GTC CCT ACT GAT TGG
M6280           CCC TCA ACG GTG CAA AGG GGT TTA AAT GGT GAG CCT ATC TTT GAT CCT TTT AAA GGC TTT GGT TTA ACT GGT AAT GCT CCT ACT GAT TGG
M6282           CCC TCA ACG GTG CAA AGG GGT TTA AAT GGC GAG CCT ATC TTT GAT CCT TTT AAA GGC TTT GGT TTA ACT GGT AAT GCC CCT ACT GAT TGG
M6283           CCC TCA ACG GTG CAA AGG GGT TTA AAT GGT GAG CCT ATC TTT GAT CCT TTT AAA GGC TTT GGT TTA ACT GGT AAT GCT CCT ACT GAT TGG
M6284           CCC TCA ACG GTG CAA AGG GGT TTA AAT GGC GAG CCT ATC TTT GAT CCT TTT AAA GGC TTT GGT TTA ACT GGT AAT GCC CCT ACT GAT TGG
M6285           CCC TCA ACG GTG CAA AGG GGT TTA AAT GGC GAG CCT ATC TTT GAT CCT TTT AAA GGC TTT GGT TTA ACT GGT AAT GCT CCT ACT GAT TGG
6286            CCC TCA ACG GTG CAA AGG GGT TTA AAT GGC GAG CCT ATC TTT GAT CCT TTT AAA GGC TTT GGT TTA ACT GGT AAT GCT CCT ACT GAT TGG
M6320           CCC TCA ACG GTG CAA AGG GGT TTA AAT GGC GAG CCT ATC TTT GAT CCT TTT AAA GGC TTT GGT TTA ACT GGT AAT GCC CCT ACT GAT TGG
64.0            CCC TCA ACG GTG CAA AGG GGT TTA AAT GGT GAG CCT ATC TTT GAT CCT TTT AAA GGC TTT GGT TTA ACT GGT AAT GCT CCT ACT GAT TGG
64.1            CCC TCA ACG GTG CAA AGG GGT TTA AAT GGT GAG CCT ATC TTT GAT CCT TTT AAA GGC TTT GGT TTA ACT GGT AAT GCT CCT ACT GAT TGG
199.0           CCC TCA ACG GTG CAA AGG GGT TTA AAT GGC GAG CCT ATC TTT GAT CCT TTT AAG GGC TTT GGT CTA ACT GGT AAT GCC CCT ACT GAT TGG
199.1           CCC TCA ACG GTG CAA AGG GGT TTA AAT GGC GAG CCT ATC TTT GAT CCT TTT AAG GGC TTT GGT CTA ACT GGT AAT GCC CCT ACT GAT TGG
KOR10046        CCC TCA ACG GTG CAA AGG GGT TTA AAT GGC GAG CCT ATC TTT GAT CCT TTT AAA GGC TTT GGT TTA ACT GGT AAT GCC CCT ACT GAT TGG
KOR10081        CCC TCA ACG GTG CAA AGG GGT TTA AAT GGC GAG CCT ATC TTT GAT CCT TTT AAA GGC TTT GGT TTA ACT GGT AAT GCC CCT ACT GAT TGG
KOR10089        CCC TCA ACG GTG CAA AGG GGT TTA AAT GGC GAG CCT ATC TTT GAT CCT TTT AAA GGC TTT GGT TTA ACT GGT AAT GCT CCT ACT GAT TGG
KOR10090        CCC TCA ACG GTG CAA AGG GGT TTA AAT GGC GAG CCT ATC TTT GAT CCT TTT AAA GGC TTT GGT TTA ACT GGT AAT GCT CCT ACT GAT TGG
KOR10114        CCC TCA ACG GTG CAA AGG GGT TTA AAT GGC GAG CCT ATC TTT GAT CCT TTT AAA GGC TTT GGT TTA ACT GGT AAT GCT CCT ACT GAT TGG
KOR10139        CCC TCA ACG GTG CAA AGG GGT TTA AAT GGC GAG CCT ATC TTT GAT CCT TTT AAA GGC TTT GGT TTA ACT GGT AAT GCC CCT ACT GAT TGG
KOR10163        CCC TCA ACG GTG CAA AGG GGT TTA AAT GGC GAG CCT ATC TTT GAT CCT TTT AAA GGC TTT GGT TTA ACT GGT AAT GCC CCT ACT GAT TGG
KOR10177        CCC TCA ACG GTG CAA AGG GGT TTA AAT GGC GAG CCT ATC TTT GAT CCT TTT AAA GGC TTT GGT TTA ACT GGT AAT GCT CCT ACT GAT TGG
KOR10274        CCC TCA ACG GTG CAA AGG GGT TTA AAT GGC GAG CCT ATC TTT GAT CCT TTT AAA GGC TTT GGT TTA ACT GGT AAT GCC CCT ACT GAT TGG
MEGA10366       CCC TCA ACG GTG CAA AGG GGT TTA AAT GGC GAG CCT ATC TTT GAT CCT TTT AAA GGC TTT GGT TTA ACT GGT AAT GCT CCT ACT GAT TGG
MEGA10378       CCC TCA ACG GTG CAA AGG GGT TTA AAT GGC GAG CCT ATC TTT GAT CCT TTT AAA GGC TTT GGT TTA ACT GGT AAT GCC CCT ACT GAT TGG
MEGA10467       CCC TCA ACG GTG CAA AGG GGT TTA AAT GGC GAG CCT ATC TTT GAT CCT TTT AAA GGC TTT GGT TTA ACT GGT AAT GCC CCT ACT GAT TGG
MEGA10477       CCC TCA ACG GTG CAA AGG GGT TTA AAT GGT GAG CCT ATC TTT GAT CCT TTT AAA GGC TTT GGT TTA ACT GGT AAT GCT CCT ACT GAT TGG
Sea1            CCC TCA ACG GTG CAA AGG GGT TTA AAT GGT GAG CCT ATC TTT GAT CCT TTT AAA GGC TTT GGT TTA ACT GGT AAT GCT CCT ACT GAT TGG
Sea2            CCC TCA ACG GTG CAA AGG GGT TTA AAT GGC GAG CCT ATC TTT GAT CCT TTT AAA GGC TTT GGT TTA ACT GGT AAT GCT CCT ACT GAT TGG
                *** *** *** *** *** *** *** *** *** **  *** *** *** *** *** *** *** **  *** *** ***  ** *** *** *** *   *** * * *** ***
G37        151   P   S   T   V   Q   R   G   L   N   G   E   P   I   F   D   P   F   K   G   F   G   L   T   G   N   A   P   T   D   W
                                                                                                                     V       L

Consensus  151   P   S   T   V   Q   R   G   L   N   G   E   P   I   F   D   P   F   K   G   F   G   L   T   G   N   A   P   T   D   W
```

**G37**	**541**	**AAT**	**GAG**	**ATC**	**AAA**	**GGT**	**AAA**	**GTT**	**CCA**	**GTA**	**GAA**	**GTA**	**GTT**
M30		AAT	GAG	ATC	AAA	GGT	AAA	GTT	CCA	GTA	GAA	GTA	GTT
M2282		AAT	GAG	ATC	AAA	GGT	AAA	GTT	CCA	GTA	GAA	GTA	GTT
M2288		AAT	GAG	ATC	AAA	GGT	AAA	GTT	CCA	GTA	GAA	GTA	GTT
M2300		AAT	GAG	ATC	AAA	GGT	AAA	GTT	CCA	GTA	GAA	GTA	GTT
M2321		AAT	GAG	ATC	AAA	GGT	AAA	GTT	CCA	GTA	GAA	GTA	GTC
M2341		AAT	GAG	ATC	AAA	GGT	AAA	GTT	CCA	GTA	GAA	GTA	GTC
M6257		AAT	GAG	ATC	AAA	GGT	AAA	GTT	CCA	GTA	GAA	GTA	GTT
M6280		AAT	GAG	ATC	AAA	GGT	AAA	GTT	CCA	GTA	GAA	GTA	GTC
M6282		AAT	GAG	ATC	AAA	GGT	AAA	GTT	CCA	GTA	GAA	GTA	GTT
M6283		AAT	GAG	ATC	AAA	GGT	AAA	GTT	CCA	GTA	GAA	GTA	GTC
M6284		AAT	GAG	ATC	AAA	GGT	AAA	GTT	CCA	GTA	GAA	GTA	GTT
M6285		AAT	GAG	ATC	AAA	GGT	AAA	GTT	CCA	GTA	GAA	GTA	GTC
6286		AAT	GAG	ATC	AAA	GGT	AAA	GTT	CCA	GTA	GAA	GTA	GTC
M6320		AAT	GAG	ATC	AAA	GGT	AAA	GTT	CCA	GTA	GAA	GTA	GTT
64.0		AAT	GAG	ATC	AAA	GGT	AAA	GTT	CCA	GTA	GAA	GTA	GTC
64.1		AAT	GAG	ATC	AAA	GGT	AAA	GTT	CCA	GTA	GAA	GTA	GTC
199.0		AAT	GAG	ATC	AAA	GGT	AAA	GTT	CCA	GTA	GAA	GTA	GTT
199.1		AAT	GAG	ATC	AAA	GGT	AAA	GTT	CCA	GTA	GAA	GTA	GTT
KOR10046		AAT	GAG	ATC	AAA	GGT	AAA	GTT	CCA	GTA	GAA	GTA	GTT
KOR10081		AAT	GAG	ATC	AAA	GGT	AAA	GTT	CCA	GTA	GAA	GTA	GTT
KOR10089		AAT	GAG	ATC	AAA	GGT	AAA	GTT	CCA	GTA	GAA	GTA	GTT
KOR10090		AAT	GAG	ATC	AAA	GGT	AAA	GTT	CCA	GTA	GAA	GTA	GTT
KOR10114		AAT	GAG	ATC	AAA	GGT	AAA	GTT	CCA	GTA	GAA	GTA	GTT
KOR10139		AAT	GAG	ATC	AAA	GGT	AAA	GTT	CCA	GTA	GAA	GTA	GTT
KOR10163		AAT	GAG	ATC	AAA	GGT	AAA	GTT	CCA	GTA	GAA	GTA	GTT
KOR10177		AAT	GAG	ATC	AAA	GGT	AAA	GTT	CCA	GTA	GAA	GTA	GTT
KOR10274		AAT	GAG	ATC	AAA	GGT	AAA	GTT	CCA	GTA	GAA	GTA	GTT
MEGA10366		AAT	GAG	ATC	AAA	GGT	AAA	GTT	CCA	GTA	GAA	GTA	GTC
MEGA10378		AAT	GAG	ATC	AAA	GGT	AAA	GTT	CCA	GTA	GAA	GTA	GTT
MEGA10467		AAT	GAG	ATC	AAA	GGT	AAA	GTT	CCA	GTA	GAA	GTA	GTT
MEGA10477		AAT	GAG	ATC	AAA	GGT	AAA	GTT	CCA	GTA	GAA	GTA	GTC
Sea1		AAT	GAG	ATC	AAA	GGT	AAA	GTT	CCA	GTA	GAA	GTA	GTC
Sea2		AAT	GAG	ATC	AAA	GGT	AAA	GTT	CCA	GTA	GAA	GTA	GTC
		***	***	***	***	***	***	***	***	***	***	***	**
**G37**	**181**	**N**	**E**	**I**	**K**	**G**	**K**	**V**	**P**	**V**	**E**	**V**	**V**

**Consensus**	**181**	**N**	**E**	**I**	**K**	**G**	**K**	**V**	**P**	**V**	**E**	**V**	**V**

Repeat Region B (bp 577-1,017, aa 193-339)
42 Sequences Analyzed

**G37**	**577**	**CAA**	**TCC**	**CCC**	**CAT**	**TCC**	**CCC**	**AAC**	**CTC**	**TAT**	**TTT**	**GTG**	**TTA**	**CTA**	**GTG**	**CCT**	**AAG**	**GTG**	**GCA**	**TTA**	**GAG**	**TAT**	**CAC**	**AAC**	**CTG**	**AAT**	**AAC**	**CAA**	**GTA**	**GTC**	**AAA**
G371-2		CAA	TCC	CCC	CAT	TCC	CCC	AAC	CTC	TAT	TTT	GTG	TTA	CTA	GTG	CCT	AAG	GTG	GCA	TTA	GAG	TAT	CAC	AAC	CTG	AAT	AAC	CAA	GTA	GTC	AAA
G371-3		CAA	TCC	CCC	CAT	TCC	CCC	AAC	CTC	TAT	TTT	GTG	TTA	CTA	GTG	CCT	AAG	GTG	GTA	TTG	GAG	TAT	CAC	AAC	CTG	AAT	AAC	CAA	GTA	GTC	AAA
G37-vB		CAA	TCC	CCC	CAT	TCC	CCC	AAC	CTC	TAT	TTT	GTG	TTA	CTA	GTG	CCT	AAG	GTG	GCA	TTA	GAG	TAT	CAC	AAC	CTG	AAT	AAC	CAA	GTA	GTC	AAA
G37-DK		CAA	TCC	CCC	CAT	TCC	CCC	AAC	CTC	TAT	TTT	GTG	TTA	CTA	GTG	CCT	AAG	GTG	GCA	TTA	GAG	TAT	CAC	AAC	CTG	AAT	AAC	CAA	GTA	GTC	AAA
G37.A01120vB		CAA	TCC	CCC	CAT	TCC	CCC	AAC	CTC	TAT	TTT	GTG	TTA	CTA	GTG	CCT	AAG	GTG	GTA	TTG	GAG	TAT	CAC	AAC	CTG	AAT	AAC	CAA	GTA	GTC	AAA
M30		CAA	TCC	CCC	CAT	TCC	CCC	AAC	CTC	TAT	TTT	CTG	TTA	CTA	GTG	CCT	AAG	GTG	GTA	TTA	GAG	TAC	CAC	AAC	CTG	AAT	AAC	CAA	GTA	GTC	AAA
M2282		CAA	TCC	CCC	CAT	TCC	CCC	AAC	CTC	TAT	TTT	GTG	TTA	CTA	GTG	CCT	AAG	GTG	GTA	GTG	GAG	TAT	CAC	AAG	CTC	AGT	AAG	GAT	GTA	GTC	AAA
M2288		CAA	TCC	CCC	CAT	TCC	CCC	AAC	CTC	TAT	TTT	GTG	TTA	CTA	GTG	CCT	AAG	GTG	GTA	GTG	GAG	TAC	CAC	AAG	CTC	AGT	AAG	AAG	GTT	GTC	AAA
M2300		CAA	TCC	CCC	CAT	TCC	CCC	AAC	CTC	TAT	TTT	CTG	TTA	CTA	GTG	CCT	AAG	GTG	GCA	TTA	GAG	TAT	CAC	AAC	CTG	AAT	AAC	CAA	GTA	GTC	AAA
M2321		CAA	TCC	CCC	CTC	AAC	CCC	AAC	CTC	TAT	TTT	GTG	TTA	CTA	GTG	CCT	AAG	GTG	GTA	TTG	GAG	TAT	CAC	AAC	CTG	AAT	AAC	CAA	GTA	GTC	AAA
M2341		CAA	TCC	CCC	CAT	TCC	CCC	AAC	CTC	TAT	TTT	GTG	TTA	CTA	GTG	CCT	AAG	GTG	GCA	TTA	GAG	TAC	CAC	CAA	CTT	GAT	AAG	AAA	GTA	GTC	AAA
M6257		CAA	TCC	CCC	CAT	TCC	CCC	AAC	CTC	TAT	TTT	GTG	TTA	CTA	GTG	CCT	AAG	GTG	GTA	TTG	GAG	TAT	CAC	AAC	CTG	AAT	AAC	CAA	GTA	GTC	AAA
M6280		CAA	TCC	CCC	CAT	TCC	CCC	AAC	CTC	TAT	TTT	GTG	TTA	CTA	GTG	CCT	AAG	GTG	GTA	TTA	GAG	TAT	CAC	CAA	CTT	GAT	AAG	---	GTT	GTC	AAA
M6282		CAA	TCC	CCC	CAT	TCC	CCC	AAC	CTC	TAT	TTT	GTG	TTA	CTA	GTG	CCT	AAG	GTG	GTA	GTG	GAG	TAC	CAC	CAA	CTT	GAT	AAG	---	GTT	GTC	AAA
M6283		CAA	TCC	CCC	CAT	TCC	CCC	AAC	CTC	TAT	TTT	CTG	TTA	CTA	GTG	CCA	AAA	GTG	GTA	TTA	GAG	TAT	CAC	AAC	CTG	AAT	AAC	CAA	GTA	GTC	AAA
M6284		CAA	TCC	CCC	CAT	TCC	CCC	AAC	CTC	TAT	TTT	CTG	TTA	CTA	GTG	CCT	AAG	GTG	GTA	GTG	GAG	TAT	CAC	AAG	CTC	AGT	AAG	GAT	GTA	GTC	AAA
M6285		CAA	TCC	CCC	CTC	AAC	CCC	AAC	CTC	TAT	TTT	CTG	TTA	CTA	GTG	CCT	AAG	GTG	GTA	TTA	GAG	TAC	CAC	CAA	CTT	GAT	AAG	AAG	GTT	GTC	AAA
6286		CAA	TCC	CCC	CAT	TCC	CCC	AAC	CTC	TAT	TTT	GTG	TTA	CTA	GTG	CCT	AAG	GTA	GTA	TTA	GAG	TAT	CAC	CAA	CTT	GAT	AAG	AAG	GTT	GTC	AAA
M6320		CAA	TCC	CCC	CAT	TCC	CCC	AAC	CTC	TAT	TTT	CTG	TTA	CTA	GTG	CCT	AAG	GTG	GTA	TTG	GAG	TAT	CAC	AAG	CTC	AGT	AAG	GAT	GTA	GTC	AAA
64.0		CAA	TCC	CCC	CAT	TCC	CCC	AAC	CTC	TAT	TTT	CTG	TTA	CTA	GTG	CCT	AAG	GTG	GTA	TTG	GAG	TAT	CAC	AAC	CTG	AAT	AAC	CAA	GTA	GTC	AAA
64.1		CAA	TCC	CCC	CAT	TCC	CCC	AAC	CTC	TAT	TTT	CTG	TTA	CTA	GTG	CCT	AAG	GTG	GTA	TTG	GAG	TAT	CAC	AAC	CTG	AAT	AAC	CAA	GTA	GTC	AAA
199.0		CAA	TCC	CCC	CAT	TCC	CCC	AAC	CTC	TAT	TTT	GTG	TTA	CTA	GTG	CCT	AAG	GTG	GTA	GTG	GAG	TAT	CAC	CAA	CTT	GAT	AAG	---	GTT	GTC	AAA
199.1		CAA	TCC	CCC	CAT	TCC	CCC	AAC	CTC	TAT	TTT	GTG	TTA	CTA	GTG	CCT	AAG	GTG	GTA	GTG	GAG	TAT	CAC	CAA	CTT	GAT	AAG	---	GTT	GTC	AAA
KOR10139.a		CAA	TCC	CCC	CAT	TCC	CCC	AAC	CTC	TAT	TTT	GTG	TTA	CTA	GTG	CCA	AAA	GTG	GTA	TTG	GAG	TAT	CAC	AAC	CTG	AAT	AAC	CAA	GTA	GTC	AAA
KOR10139.b		CAA	TCC	CCC	CAT	TCC	CCC	AAC	CTC	TAT	TTT	GTG	TTA	CTA	GTG	CCA	AAA	GTG	GTA	TTG	GAG	TAT	CAC	AAC	CTG	AAT	AAC	CAA	GTA	GTC	AAA
KOR10139.c		CAA	TCC	CCC	CAT	TCC	CCC	AAC	CTC	TAT	TTT	GTG	TTA	CTA	GTG	CCT	AAG	GTG	GTA	GTG	GAG	TAC	CAC	CAA	CTT	GAT	AAG	---	GTT	GTC	AAA
KOR10139.d		CAA	TCC	CCC	CAT	TCC	CCC	AAC	CTC	TAT	TTT	GTG	TTA	CTA	GTG	CCA	AAA	GTG	GTA	TTG	GAG	TAT	CAC	AAC	CTG	AAT	AAC	CAA	GTA	GTC	AAA
KOR10139.e		CAA	TCC	CCC	CAT	TCC	CCC	AAC	CTC	TAT	TTT	GTG	TTA	CTA	GTG	CCA	AAA	GTG	GTA	TTG	GAG	TAC	CAC	CAA	CTT	GAT	AAG	---	GTT	GTC	AAA
KOR10139.f		CAA	TCC	CCC	CAT	TCC	CCC	AAC	CTC	TAT	TTT	GTG	TTA	CTA	GTG	CCT	AAG	GTG	GTA	GTG	GAG	TAC	CAC	CAA	CTT	GAT	AAG	---	GTT	GTC	AAA
KOR10139.g		CAA	TCC	CCC	CAT	TCC	CCC	AAC	CTC	TAT	TTT	GTG	TTA	CTA	GTG	CCT	AAG	GTG	GTA	GTG	GAG	TAC	CAC	CAA	CTT	GAT	AAG	---	GTT	GTC	AAA
KOR10139.h		CAA	TCC	CCC	CAT	TCC	CCC	AAC	CTC	TAT	TTT	GTG	TTA	CTA	GTG	CCT	AAG	GTG	GTA	GTG	GAG	TAC	CAC	CAA	CTT	GAT	AAG	---	GTT	GTC	AAA
KOR10139.j		CAA	TCC	CCC	CAT	TCC	CCC	AAC	CTC	TAT	TTT	GTG	TTA	CTA	GTG	CCA	AAA	GTG	GTA	TTG	GAG	TAT	CAC	AAC	CTG	AAT	AAC	CAA	GTA	GTC	GAA
KOR10139.k		CAA	TCC	CCC	CAT	TCC	CCC	AAC	CTC	TAT	TTT	GTG	TTA	CTA	GTG	CCT	AAG	GTG	GTA	GTG	GAG	TAC	CAC	CAA	CTT	GAT	AAG	---	GTT	GTC	AAA
KOR10139.l		CAA	TCC	CCC	CAT	TCC	CCC	AAC	CTC	TAT	TTT	GTG	TTA	CTA	GTG	CCA	AAA	GTG	GTA	TTG	GAG	TAT	CAC	AAC	CTG	AAT	AAC	CAA	GTA	GTC	GAA
KOR10139.m		CAA	TCC	CCC	CAT	TCC	CCC	AAC	CTC	TAT	TTT	GTG	TTA	CTA	GTG	CCA	AAA	GTG	GTA	TTG	GAG	TAT	CAC	AAC	CTG	AAT	AAC	CAA	GTA	GTC	AAA
KOR10139.n		CAA	TCC	CCC	CAT	TCC	CCC	AAC	CTC	TAT	TTT	GTG	TTA	CTA	GTG	CCA	AAA	GTG	GTA	TTG	GAG	TAC	CAC	CAA	CTT	GAT	AAG	---	GTT	GTC	AAA
KOR10139.o		CAA	TCC	CCC	CAT	TCC	CCC	AAC	CTC	TAT	TTT	GTG	TTA	CTA	GTG	CCT	AAG	GTG	GTA	TTG	GAG	TAT	CAC	AAC	CTG	AAT	AAC	CAA	GTA	GTC	GTA
KOR10139.p		CAA	TCC	CCC	CAT	TCC	CCC	AAC	CTC	TAT	TTT	GTG	TTA	CTA	GTG	CCT	AAG	GTG	GTA	GTG	GAG	TAC	CAC	CAA	CTT	GAT	AAG	---	GTT	GTC	AAA
KOR10139.q		CAA	TCC	CCC	CAT	TCC	CCC	AAC	CTC	TAT	TTT	GTG	TTA	CTA	GTG	CCT	AAG	GTG	GTA	GTG	GAG	TAC	CAC	CAA	CTT	GAT	AAG	---	GTT	GTC	AAA
Sea1		CAA	TCC	CCC	CAT	TCC	CCC	AAC	CTC	TAT	TTT	GTG	TTA	CTA	GTG	CCA	AAA	GTG	GTA	TTG	GAG	TAT	CAC	AAC	CTG	AAT	AAC	CAA	GTA	GTC	AAA
Sea2		CAA	TCC	CCC	CAT	TCC	CCC	AAC	CTC	TAT	TTT	GTG	TTA	CTA	GTG	CCT	AAG	GTG	GTA	GTG	GAG	TAT	CAC	AAC	CTG	AAT	AAC	CAA	GTA	GTC	AAA
		***	***	***	*	*	***	***	***	***	***	**	***	***	***	**	**	**	* *	*	***	**	***	*	**		**		**	***	*
**G37**	**193**	**Q**	**S**	**P**	**H**	**S**	**P**	**N**	**L**	**Y**	**F**	**V**	**L**	**L**	**V**	**P**	**K**	**V**	**A**	**L**	**E**	**Y**	**H**	**N**	**L**	**N**	**N**	**Q**	**V**	**V**	**K**
					**L**	**N**						**L**							**V**	**V**				**K**		**S**	**K**	**-**			**E**
																								**Q**		**D**		**D**			**V**
																												**K**			
**Consensus**	**193**	**Q**	**S**	**P**	**H**	**S**	**P**	**N**	**L**	**Y**	**F**	**V**	**L**	**L**	**V**	**P**	**K**	**V**	**V**	**L**	**E**	**Y**	**H**	**N**	**L**	**N**	**N**	**Q**	**V**	**V**	**K**

Variable Region B (bp 577-1,017; aa 193-339)

**G37**	**667**	**GAG**	**AGT**	**TTG**	**GAA**	**GTG**	**AAA**	**GCA**	**ACC**	**CAA**	**TCA**	**TCC**	**TTC**	**AAC**	**CCC**	**ACC**	**CAA**	**AGG**	**TTG**	**CAA**	**AAA**	**GAT**	**AGT**	**CCA**	**GTG**	**AAG**	**GAT**	**TCA**	**AGT**	**AAA**	**CAA**
G371-2		GAG	AGT	TTG	GAA	GTG	GAA	GCA	ACT	AAT	TCT	---	TTT	GAT	CCC	ACC	CAA	GGG	TTG	CAA	AAA	GAT	AGT	CCA	GTG	AAG	GAT	TCA	AGT	AAA	CAA
G371-3		GAG	AGT	TTG	GAA	GTG	GAA	GCA	ACT	GAT	TCT	---	TTT	GAT	CCC	ACC	CAA	AGG	TTG	CAA	AAA	GAT	AGT	CCA	AAG	AAG	GAT	ACA	GGA	AAG	ATG
G37-vB		GAG	AGT	TTG	GAA	GTG	GAA	GCA	ACT	GAT	TCT	---	TTT	GAT	CCC	ACC	CAA	GGG	TTG	CAA	AAA	GAT	AGT	CCA	GTG	AAG	GAT	TCA	AGT	AAA	CAA
G37-DK		GAG	AGT	TTG	GAA	GTG	AAA	GCA	ACC	CAA	TCA	TCC	TTC	ACC	CCC	ACC	CAA	AGG	TTG	CAA	AAA	GAT	AGT	CCA	GTG	AAG	GAT	TCA	AGT	AAA	CAA
G37.A01120vB		GAG	AGT	TTG	GAA	GTG	GAA	GCA	ACT	GAT	TCT	---	TTT	GAT	CCC	ACC	CAA	AGG	TTG	CAA	AAA	GAT	AGT	CCA	ATG	AAG	GAT	ACA	GGA	AAG	ATG
M30		GAG	AGT	TTG	GAA	GTG	GAA	GCA	ACT	GAT	TCT	---	TTT	GAT	CCC	ACC	CAA	AGG	TTG	AAG	AGT	GGG	AGT	CCA	ATG	AAG	GAT	ACA	GGA	AAG	ATG
M2282		GAG	AGT	TTG	GAA	GTG	GAA	GCA	ACT	GAT	TCT	---	TTT	GAT	CCC	ACC	CAA	AGG	TTG	CAA	AAA	GAT	AGT	CCG	GTG	AAG	GAT	TCA	AAC	AAA	GAC
M2288		GAG	AGT	TTG	GAA	GTG	GAA	GCA	ACT	GAT	TCC	---	TTT	GAT	CCC	ACC	CAA	AGG	TTG	AAA	AAA	GAT	AGT	CCA	GTG	AAG	GAT	TCA	ACA	AAA	ACA
M2300		GAG	AGT	TTG	GAA	GTG	GAA	GCA	ACC	CAA	TCA	TCC	TTC	AAC	CCC	ACC	CAA	AGG	TTG	AAG	AGT	GGG	AGT	CCA	ATG	AAG	GAT	ACA	GGA	AAG	ATG
M2321		GAG	AGT	TTG	GGG	GTG	GAT	ACA	TCA	GGT	TCA	ACT	TTT	GAT	CCC	ACC	CAA	AGG	TTG	CAA	AAA	GAT	AGT	CCG	GTG	AAG	GAT	TCA	AGT	AAA	CAA
M2341		GAG	AGT	TTG	GAA	GTG	GAA	GCA	ACT	GAT	TCT	---	TTT	GAT	CCA	ACT	AAA	AGG	TTG	CAA	AAA	GAT	AGT	CCA	ATG	AAG	GAT	TCA	AGT	AAA	CAA
M6257		GAG	AGT	TTG	GGG	GTG	GAA	GCA	ACT	---	TCC	ACC	TTC	AAC	CCC	ACC	CAA	AGA	TTG	CAA	AAA	GAG	AGT	CCA	ATG	AAG	GAT	TCA	AAC	AAA	GAC
M6280		GAG	AGT	TTG	GAA	ATG	GAA	GCA	ACT	---	TCC	TCT	TTC	AAC	CCC	ACC	CAA	AGG	TTG	AAG	AGT	GAT	AGT	CCA	GAA	AGC	GAT	TCA	AGT	AAA	CAA
M6282		GAG	AGT	TTG	GAA	GTG	GAA	GCA	ACT	---	TCC	AAC	TTT	GAT	CCA	ACT	AAA	AGG	TTG	CAA	AAA	GAG	AGT	CCA	CAA	AAA	GAT	CCA	GCA	AAA	GAA
M6283		GAG	AGT	TTG	GAA	GTG	GAA	TCT	TCT	---	TCC	TCT	TTC	AAC	CCC	ACC	CAA	AGG	TTG	CAA	AGT	GGG	AGT	CCA	GAA	AAG	GAT	GAT	AGT	AAA	ACA
M6284		GAG	AGT	TTG	GGG	GTG	GAT	ACA	TCA	GGT	TCA	ACT	TTT	GAT	CCA	ACT	AAA	AGG	TTG	AAA	AAA	GAT	AGT	CCA	GTG	AAG	GAT	TCA	AAC	AAA	GAC
M6285		GAG	AGT	TTG	GGG	GTG	GAA	TCT	TCT	---	TCC	TCT	TTC	AAC	CCC	ACC	CAA	AGG	TTG	AAG	AAA	GAG	AGT	CCA	ATG	AAG	GAT	TCA	AGT	AAA	CAA
6286		GAG	AGT	TTG	GAA	GTG	GAA	GCA	ACT	GAT	TCC	---	TTT	GAT	CCA	ACT	AAA	AGG	TTG	CAA	AAA	GAT	AGT	CCA	ATG	AAG	GAT	TCA	ACA	AAA	ACA
M6320		GAG	AGT	TTG	GGG	GTG	GAA	GCA	ACC	CAA	TCA	TCC	TTC	AAC	CCC	ACC	CAA	AGG	TTG	CAA	AAA	GAG	AGT	CCG	GTG	ATG	GAT	ACA	GGA	AAG	ATG
64.0		GAG	AGT	TTG	GAA	GTG	GAA	TCT	TCT	---	TCC	TCT	TTC	AAC	CCC	ACC	CAA	AGG	TTG	AAG	AGT	GAT	AGT	CCA	GTG	AAG	GAT	TCA	AAC	AAA	GAC
64.1		GAG	AGT	TTG	GAA	GTG	GAA	TCT	TCT	---	TCC	TCT	TTC	AAC	CCC	ACC	CAA	AGG	TTG	AAG	AGT	GAT	AGT	CCA	GTG	AAG	GAT	TCA	AAC	AAA	GAC
199.0		GAG	AGT	TTG	GAA	GTG	GAA	GCA	ACT	---	TCC	ACC	TTC	AAC	CCC	ACC	CAA	GGG	TTG	CAA	AGT	ATG	GGG	CCT	GAA	AAA	GAT	TCA	ACA	AAA	ACA
199.1		GAG	AGT	TTG	GAA	GTG	GAA	GCA	ACT	---	TCC	ACC	TTC	AAC	CCC	ACC	CAA	GGG	TTG	CAA	AGT	ATG	GGG	CCT	GAA	AAA	GAT	TCA	ACA	AAA	ACA
KOR10139.a		GAG	AGT	TTG	GAA	GTG	GAA	GCA	ACT	GAT	TCT	---	TTT	GAT	CCC	ACC	CAA	AGG	TTG	AAG	AAA	GAG	AGT	CCA	ATG	AAG	GAT	TCA	AAC	AAA	GAC
KOR10139.b		GAG	AGT	TTG	GAA	GTG	GAA	GCA	ACT	GAT	TCT	---	TTT	GAT	CCC	ACC	CAA	AGG	TTG	AAG	AGT	GAT	AGT	CCA	GTG	AAG	GAT	TCA	AAC	AAA	AAC
KOR10139.c		GAG	AGT	TTG	GAA	GTG	GAA	GCA	ACT	GAT	TCT	---	TTT	GAT	CCC	ACC	CAA	AGG	TTG	AAG	AAA	GAG	AGT	CCA	ATG	AAG	GAT	TCA	AAC	AAA	GAC
KOR10139.d		GAG	AGT	TTG	GAA	GTG	GAA	TCT	TCT	---	TCC	TCC	TTT	GAT	CCC	ACC	CAA	AGG	TTG	CAA	AAA	GAT	AGT	CCA	GAA	AGC	GAT	TCA	AAC	GAA	GAC
KOR10139.e		GAG	AGT	TTG	GAA	GTG	GAA	TCT	TCT	---	TCC	TCC	TTT	GAT	CCC	ACC	CAA	AGG	TTG	AAG	AGT	GAT	AGT	CCA	GTG	AAG	GAT	TCA	AAC	AAA	AAC
KOR10139.f		GAG	AGT	TTG	GAA	GTG	GAA	GCA	ACT	GAT	TCT	---	TTT	GAT	CCC	ACC	CAA	AGG	TTG	CAA	AAA	GAT	AGT	CCA	GAA	AGC	GAT	TCA	AAC	AAA	GAC
KOR10139.g		GAG	AGT	TTG	GAA	GTG	GAA	TCT	TCT	---	TCC	TCC	TTT	GAT	CCC	ACC	CAA	AGG	TTG	CAA	AAA	GAT	AGT	CCA	GAA	AGC	GAT	TCA	AAC	AAA	GAC
KOR10139.h		GAG	AGT	TTG	GAA	GTG	GAA	TCT	TCT	---	TCC	TCC	TTT	GAT	CCC	ACC	CAA	AGG	TTG	AAG	AGT	GAT	AGT	CCA	GTG	AAG	GAT	TCA	AAC	AAA	AAC
KOR10139.j		GAG	AGT	TTG	GAA	GTG	GAA	TCT	TCT	---	TCC	TCC	TTT	GAT	CCC	ACC	CAA	AGG	TTG	CAA	AAA	GAT	AGT	CCA	GTG	AAG	GAT	TCA	AAC	AAA	GAC
KOR10139.k		GAG	AGT	TTG	GAA	GTG	GAA	TCT	TCT	---	TCC	TCC	TTT	GAT	CCC	ACC	CAA	AGG	TTG	AAG	AGT	GAT	AGT	CCA	GTG	AAG	GAT	TCA	AAC	AAA	AAC
KOR10139.l		GAG	AGT	TTG	GAA	GTG	GAA	TCT	TCT	---	TCC	TCC	TTT	GAT	CCC	ACC	CAA	GGG	TTG	AAG	AGT	GAT	AGT	CCA	GTG	AAG	GAT	TCA	AAC	AAA	AAC
KOR10139.m		GAG	AGT	TTG	GAA	GTG	GAA	TCT	TCT	---	TCC	TCC	TTT	GAT	CCC	ACC	CAA	AGG	TTG	AAG	AGT	GAT	AGT	CCA	GTG	AAG	GAT	TCA	AAC	AAA	GAC
KOR10139.n		GAG	AGT	TTG	GAA	GTG	GAA	GCA	ACT	GAT	TCT	---	TTT	GAT	CCC	ACC	CAA	AGG	TTG	AAG	AGT	GAT	AGT	CCA	GTG	AAG	GAT	TCA	AAC	AAA	GAC
KOR10139.o		GAG	AGT	TTG	GAA	GTG	GAA	TCT	TCT	---	TCC	TCC	TTT	GAT	CCC	ACC	CAA	GGG	TTG	AAG	AGT	GAT	AGT	CCA	GTG	AAG	GAT	TCA	AAC	AAA	AAC
KOR10139.p		GAG	AGT	TTG	GAA	GTG	GAA	TCT	TCT	---	TCC	TCT	TTC	AAC	CCC	ACC	CAA	AGG	TTG	AAG	AGT	GAT	AGT	CCA	GTG	AAG	GAT	TCA	AAC	AAA	GAC
KOR10139.q		GAG	AGT	TTG	GAA	GTG	GAA	GCA	ACT	GAT	TCT	---	TTT	GAT	CCC	ACC	CAA	GGG	TTG	AAG	AGT	GAT	AGT	CCA	GTG	AAG	GAT	TCA	AAC	AAA	AAC
Sea1		GAG	AGT	TTG	GGG	GTG	GAA	GCA	ACC	CAA	TCA	TCC	TTC	AAC	CCC	ACC	CAA	AGG	TTG	AAA	AAA	GAT	AGT	CCA	GTG	AAG	GAT	TCA	AGT	AAA	CAA
Sea2		GAG	AGT	TTG	GGG	GTG	GAA	GCA	ACT	GAT	TCT	---	TTT	GAT	CCC	ACC	CAA	GGG	TTG	CAA	AAA	GAT	AGT	CCA	ATG	AAG	GAT	TCA	AGT	AAA	ACA
		***	***	***	*	**	*	*	*				**		**	**	**	*	***	*	*		*	**		*	***			*	
**G37**	**223**	**E**	**S**	**L**	**E**	**V**	**K**	**A**	**T**	**Q**	**S**	**S**	**F**	**N**	**P**	**T**	**Q**	**R**	**L**	**Q**	**K**	**D**	**S**	**P**	**V**	**K**	**D**	**S**	**S**	**K**	**Q**
					**G**	**M**	**E**	**T**	**S**	**-**		**-**		**D**			**K**	**G**		**K**	**S**	**G**	**G**		**K**	**S**		**T**	**G**	**E**	**M**
							**D**	**S**		**N**		**T**		**T**								**E**			**M**	**M**		**P**	**N**		**D**
										**D**												**M**			**E**			**D**	**T**		**T**
										**G**															**Q**				**A**		**E**
																															**N**
**Consensus**	**223**	**E**	**S**	**L**	**E**	**V**	**E**	**A**	**T**	**-**	**S**	**S**	**F**	**D**	**P**	**T**	**Q**	**R**	**L**	**Q**	**K**	**D**	**S**	**P**	**V**	**K**	**D**	**S**	**N**	**K**	**D**

**G37**	**757**	**GGG**	**---**	**GAG**	**AAA**	**CTC**	**AGT**	**GAA**	**ACA**	**ACT**	**GCT**	**TCA**	**TCC**	**---**	**---**	**ATG**	**AGT**	**AGT**	**GGT**	**ATG**	**GCT**	**ACA**	**TCC**	**ACT**	**CGA**	**GCC**	**AAG**	**GCC**	**CTC**	**AAA**	**GTG**
G371-2		GGG	---	GAG	AAA	CTC	CAA	GAG	ACC	ATG	---	TCA	TCA	---	---	ATG	AGT	---	GGT	ATG	GCT	ACC	TCT	ACA	AGA	GAT	AAA	GCC	CTC	AAG	ATT
G371-3		GGG	---	GAG	AAA	CTC	CAA	GAG	ACC	ATG	---	TCA	TCA	---	---	ATG	AGT	GGT	GGT	GGG	GCT	ACA	TCT	CCT	CGC	---	AAA	GCC	CTC	ACC	ATT
G37-vB		GGG	---	GAG	AAA	CTC	CAA	GAG	ACC	ATG	---	TCA	TCA	---	---	ATG	AGT	GGT	---	ATG	GCT	ACC	TCT	ACA	AGA	GAT	AAA	GCC	CTC	AAA	GTG
G37-DK		GGG	---	GAG	AAA	CTC	AGT	GAA	ACA	ACT	GCT	TCA	TCC	---	---	ATG	AGT	AGT	GGT	ATG	GCT	ACA	TCC	ACT	CGA	GCC	AAG	GCC	CTC	AAA	GTG
G37.A01120vB		GGG	---	GAG	AAA	CTC	CAA	GAG	ACC	ATG	---	TCA	TCA	---	---	ATG	AGT	GGT	GGT	GGG	GCT	ACA	TCT	CCT	CGC	---	AAA	GCC	CTC	ACC	ATT
M30		GGG	---	GAG	AAA	CTC	AGT	GAA	GCA	ACT	GCT	TCA	TCC	---	---	ATG	AGT	GGT	---	ATG	GCT	ACA	TCT	CCT	CGC	---	AAG	GCC	CTT	AAG	ATA
M2282		AGT	---	GAG	AAA	CTC	GAA	---	ACA	ACT	GCT	TCA	TCC	---	---	ATG	AGT	AGT	GGT	GGG	GCT	ACA	TCC	ACT	CGC	---	AAG	GCC	CTC	AAG	GTT
M2288		GGG	---	GAG	AAA	CTC	GAA	---	ACA	ACT	GCT	TCA	TCG	---	---	ATG	AGT	AGT	GGT	ATG	GCT	ACA	TCC	ACT	CGC	---	AAG	GCC	CTC	AAA	GTG
M2300		GGG	---	GAG	AAA	CTC	CAA	GAG	ACC	ATG	---	TCA	TCA	---	---	ATG	AGT	AGT	GGT	GGG	GCT	ACA	TCC	ACT	CGC	---	AAG	GCC	CTC	AAG	ATT
M2321		GGG	---	GAG	AAA	CTC	AGT	---	---	TTG	GCA	GAT	GGT	GGG	TCA	ATG	AGT	---	---	GGG	GCT	ACA	TCT	CTT	CGC	---	AAG	GCC	CTC	AAG	GTT
M2341		GGG	---	GAG	AAA	CTC	AGT	GAA	GCA	ATG	---	TCA	TCA	GTG	GGT	ATG	AGT	AGT	GGT	GGG	GCT	ACA	TCT	CCT	CGC	---	AAG	GCC	CTC	AAG	ATA
M6257		AGT	---	GAG	AAA	CTC	AGT	TTG	GCA	GAT	GGT	GGG	TCA	---	---	ATG	AGT	AGT	GGT	GGG	GCT	ACA	TCC	ACT	CGA	GCC	AAG	GCC	CTT	AAG	ATA
M6280		GGG	---	GAG	AAA	CTC	GAA	---	ACA	ACT	GCT	TCA	TCA	---	---	ATG	AGT	---	GGT	ATG	GCT	ACA	TCC	ACT	CGC	---	AAG	GCC	CTC	ACC	ATT
M6282		TCA	ACA	GAG	AAA	CTC	ACC	GAA	ACA	ACT	---	TCA	TCG	---	---	ATG	AGT	AGT	GGT	GGG	GCT	ACA	TTC	ACT	CGA	GCC	AAG	GCC	CTC	AAG	GTT
M6283		GGG	---	GAG	AAA	CTC	GAA	TCA	ACT	GCT	---	TCA	TCA	---	---	ATG	AGT	AGT	GGT	GGG	GCT	ACA	TCC	ACT	CGC	---	AAG	GCC	CTC	AAG	GTT
M6284		AGT	---	GAG	AAA	CTC	AGT	GAA	ACA	ACT	GCT	TCA	TCG	---	---	ATG	AGT	AGT	---	GGG	GCT	ACA	TCC	ACT	CGC	---	AAG	GCC	CTC	AAG	GTT
M6285		GGG	---	GAG	AAA	CTC	GCT	TTG	GCA	GAT	GGT	GGG	TCA	---	---	AGT	GGC	AGT	---	ATG	GCT	ACA	TCC	ACT	CGA	GCC	AAG	GCC	CTT	AAG	ATA
6286		GGG	---	GAG	AAA	CTC	CAA	GAG	ACC	ATG	---	TCA	TCA	---	---	ATG	AGT	---	GGT	ATG	GCT	ACA	TCC	ACT	CGA	GCC	AAG	GCC	CTT	AAA	GTG
M6320		GGG	---	GAG	AAA	CTC	AGT	GAA	ACA	ACT	---	TCA	TCC	---	---	ATG	AGT	AGT	GGT	GGG	GCT	ACA	TCC	ACT	CGC	---	AAG	GCC	CTT	AAG	ATA
64.0		AGT	---	GAG	AAA	CTC	AGT	GAA	GCA	ATG	---	TCA	TCA	---	---	ATG	AGT	AGT	GGT	GGG	GCT	ACA	TCC	ACT	CGC	---	AAA	GCC	CTT	AAG	ATA
64.1		AGT	---	GAG	AAA	CTC	AGT	GAA	GCA	ATG	---	TCA	TCA	---	---	ATG	AGT	AGT	GGT	GGG	GCT	ACA	TCC	ACT	CGC	---	AAA	GCC	CTT	AAG	ATA
199.0		GGG	---	GAG	AAA	CTC	AGT	GAA	GCA	ATG	---	TCA	TCC	---	---	ATG	AGT	AGT	---	GGC	CCT	ACC	TCT	CCT	CGC	---	AAG	GCC	CTC	ACC	ATT
199.1		GGG	---	GAG	AAA	CTC	AGT	GAA	GCA	ATG	---	TCA	TCC	---	---	ATG	AGT	AGT	---	GGC	CCT	ACC	TCT	CCT	CGC	---	AAG	GCC	CTC	ACC	ATT
KOR10139.a		AGT	---	GAG	AAA	CTC	AGT	TTG	GCA	GAT	GGT	GGG	TCA	---	---	ATG	AGT	AGT	GGT	GGG	GCT	ACA	TCC	ACT	CGC	---	AAG	GCC	CTC	AAG	ATT
KOR10139.b		AGT	---	GAG	AAA	CTC	AGT	TTG	GCA	GAT	GGT	GGG	TCA	---	---	ATG	AGT	AGT	GGT	GGG	GCT	ACA	TCC	ACT	CGA	GCC	AAG	GCC	CTT	AAG	ATA
KOR10139.c		AGT	---	GAG	AAA	CTC	AGT	TTG	GCA	GAT	GGT	GGG	TCA	---	---	ATG	AGT	AGT	GGT	GGG	GCT	ACA	TCC	ACT	CGC	---	AAG	GCC	CTC	AAG	ATT
KOR10139.d		AGT	---	GAG	AAA	CTC	AGT	TTG	GCA	GAT	GGT	GGG	TCA	---	---	ATG	AGT	AGT	GGT	GGG	GCT	ACA	TCC	ACT	CGC	---	AAG	GCC	CTC	AAG	ATT
KOR10139.e		AGT	---	GAG	AAA	CTC	AGT	TTG	GCA	GAT	GGT	GGG	TCA	---	---	ATG	AGT	AGT	GGT	GGG	GCT	ACA	TCC	ACT	CGA	GCC	AAG	GCC	CTC	AAG	ATT
KOR10139.f		AGT	---	GAG	AAA	CTC	AGT	TTG	GCA	GAT	GGT	GGG	TCA	---	---	ATG	AGT	AGT	GGT	GGG	GCT	ACA	TCC	ACT	CGC	---	AAG	GCC	CTC	AAG	ATT
KOR10139.g		AGT	---	GAG	AAA	CTC	AGT	TTG	GCA	GAT	GGT	GGG	TCA	---	---	ATG	AGT	AGT	GGT	GGG	GCT	ACA	TCC	ACT	CGC	---	AAG	GCC	CTC	AAG	ATT
KOR10139.h		AGT	---	GAG	AAA	CTC	AGT	TTG	GCA	GAT	GGT	GGG	TCA	---	---	ATG	AGT	AGT	GGT	GGG	GCT	ACA	TCC	ACT	CGA	GCC	AAG	GCC	CTT	AAG	ATA
KOR10139.j		AGT	---	GAG	AAA	CTC	GAA	GAA	---	---	---	TCA	TCC	---	---	ATG	AGT	---	---	GGG	GCT	ACA	TCC	ACT	CGA	GCC	AAG	GCC	CTT	AAG	ATA
KOR10139.k		AGT	---	GAG	AAA	CTC	AGT	TTG	GCA	GAT	GGT	GGG	TCA	---	---	ATG	AGT	AGT	GGT	GGG	GCT	ACA	TCC	ACT	CGA	GCC	AAG	GCC	CTT	AAG	ATA
KOR10139.l		AGT	---	GAG	AAA	CTC	GAA	GAA	---	---	---	TCA	TCC	---	---	ATG	AGT	---	---	GGG	GCT	ACA	TCC	ACT	CGC	---	AAG	GCC	CTC	AAG	ATT
KOR10139.m		AGT	---	GAG	AAA	CTC	GAA	GAA	---	---	---	TCA	TCC	---	---	ATG	AGT	---	---	GGG	GCT	ACA	TCC	ACT	CGA	GCC	AAG	GCC	CTT	AAG	ATA
KOR10139.n		AGT	---	GAG	AAA	CTC	GAA	GAA	---	---	---	TCA	TCC	---	---	ATG	AGT	---	---	GGG	GCT	ACA	TCC	ACT	CGA	GCC	AAG	GCC	CTT	AAG	ATA
KOR10139.o		AGT	---	GAG	AAA	CTC	GAA	GAA	---	---	---	TCA	TCC	---	---	ATG	AGT	---	---	GGG	GCT	ACA	TCC	ACT	CGC	---	AAG	GCC	CTC	AAG	ATT
KOR10139.p		AGT	---	GAG	AAA	CTC	GAA	GAA	---	---	---	TCA	TCC	---	---	ATG	AGT	---	---	GGG	GCT	ACA	TCC	ACT	CGA	GCC	AAG	GCC	CTC	AAG	ATT
KOR10139.q		AGT	---	GAG	AAA	CTC	GAA	GAA	---	---	---	TCA	TCC	---	---	ATG	AGT	---	---	GGG	GCT	ACA	TCC	ACT	CGA	GCC	AAG	GCC	CTC	AAG	ATT
Sea1		GGG	---	GAG	AAA	CTC	AGT	GAA	GCA	ATG	---	TCA	TCA	---	---	ATG	AGT	AGT	GGT	GGG	GCT	ACA	TCC	ACT	CGC	---	AAA	GCC	CTT	AAA	GTG
Sea2		GGG	---	GAG	AAA	CTC	AGT	GAA	GCA	ATG	---	TCA	TCG	---	---	ATG	AGT	---	GGT	ATG	GCT	ACC	TCT	ACA	AGA	GAT	AAA	GCC	CTC	AAA	GTG
				***	***	***											***				**	**	**		*		**	***	**	*	*
**G37**	**253**	**G**	**-**	**E**	**K**	**L**	**S**	**E**	**T**	**T**	**A**	**S**	**S**	**-**	**-**	**M**	**S**	**S**	**G**	**M**	**A**	**T**	**S**	**T**	**R**	**A**	**K**	**A**	**L**	**K**	**V**
		S	T				Q	-	-	-	-	D	G	G	S	S	G	-	-	G	P		F	P		-				T	I
							E	L	A	M	G	G		V	G			G						L		D					
							T	S		L																					
							A			D																					
										A																					
**G37**	**252**	**S**	**-**	**E**	**K**	**L**	**S**	**E**	**A**	**M**	**-**	**S**	**S**	**-**	**-**	**M**	**S**	**S**	**G**	**G**	**A**	**T**	**S**	**T**	**R**	**-**	**K**	**A**	**L**	**K**	**I**

Variable Region B (bp 577-1,017; aa 193-339)

**G37**	**838**	**GAG**	**GTG**	**GAA**	**AGG**	**GGG**	**AGT**	**CAA**	**---**	**AGT**	**GAT**	**TCA**	**CTT**	**TTA**	**AAA**	**---**	**AAC**	**GAC**	**TTT**	**GCT**	**AAA**	**AAG**	**CCA**	**CTA**	**AAG**	**CAT**	**AAG**	**AAC**	**AGT**	**AGT**	**GGG**
G371-2		GAG	GTG	GAA	AGG	GGG	AGT	CAA	---	AGT	GAT	TCA	CTT	TTA	AAA	---	AAC	GAC	TTT	GCT	AAA	AAG	CCA	CTG	AAA	CAT	AAG	AAC	AGT	AGT	GGG
G371-3		GAG	GTG	GAA	AGG	GGG	AGT	CAA	---	AGT	GAT	TCA	CTT	TTA	AAA	---	AAC	GAC	TTT	GCT	AAA	AAG	CCA	CTA	AAG	CAT	AAG	AAC	AGT	AGT	GGG
G37-vB		GAG	GTG	GAA	AGG	GGG	AGT	CAA	---	AGT	GAT	TCA	CTT	TTA	AAA	---	AAC	GAC	TTT	GCT	AAA	AAG	CCA	CTA	AAG	CAT	AAG	AAC	AGT	AGT	GGG
G37-DK		GAG	GTG	GAA	AGG	GGG	AGT	CAA	---	AGT	GAT	TCA	CTT	TTA	AAA	---	AAC	GAC	TTT	GCC	AAA	AAG	CCA	CTG	AAA	CAT	AAG	AAC	AGT	AGT	GGG
G37.A01120vB		GAG	GTG	GAA	AGG	GGG	AGT	CAA	---	AGT	GAT	TCA	CTT	TTA	AAA	---	AAC	GAC	TTT	GCC	AAA	AAG	CCA	CTG	AAA	CAT	AAG	AAC	AGT	AGT	GGG
M30		GAG	GTG	GAG	AAA	GGT	TCT	TCA	GGG	TCT	GAC	ACC	CTC	ACC	AAA	TCC	---	GAC	TTT	GCT	AAA	AAG	CCA	CTG	AAA	CAT	AAA	GAA	AAT	AGT	GGG
M2282		GAG	GTG	GAA	AAA	GGT	TCT	TCA	GGG	TCT	GAC	ACC	CTC	ACC	AAA	TCC	---	GAC	TTT	GCT	AAA	AAG	CCG	TTT	AAA	---	---	---	---	GAT	GAG
M2288		GAG	GTG	GAA	AGG	GGG	AGT	AAA	GTC	AAT	CAA	GGC	GAA	CTA	CAA	TCC	AAC	GAC	TTT	GCT	AAA	AAG	CCA	CTA	AAG	CAT	AAG	AAC	AGT	AGT	GGG
M2300		GAG	GTG	GAA	AGG	GGG	AAT	AAT	GTC	AAT	CAA	GGC	GAA	CTA	CAA	TCC	AAC	GAC	TTT	GCC	AAA	AAG	CCG	TTT	AAA	---	---	---	---	GAT	GAG
M2321		GAG	GTG	GAA	AGG	GGG	AGT	AAA	GTC	AAT	CAA	GGC	GAA	CTA	CAA	TCC	AAC	GAC	TTT	GCT	AAA	AAG	CCA	CTA	AAG	CAT	AAG	AAC	AGT	AGT	GGG
M2341		GAG	GTG	GAG	AAA	GGC	AGT	AAT	GTC	AAT	CAA	GGC	GAA	CTA	GCA	AAA	AAC	GAC	TTT	GCT	AAA	AAG	CCA	CTG	AAA	CAT	AAA	GAA	AAT	AGT	GGG
M6257		GAG	GTG	GAA	AGG	GGG	AGT	AAT	GTC	AAT	CAA	GGC	GAA	CTA	GCA	AAA	AAC	GAC	TTT	GCT	AAA	AAG	CCG	TTT	AAA	---	---	---	---	GAT	GAG
M6280		GAG	GTG	GAG	AAA	GGT	TCT	TCA	GGG	TCT	GAC	ACC	CTA	ACC	AAA	TCC	---	GAC	TTT	GCT	AAA	AAG	CCA	CTG	AAA	CAT	AAA	GAC	AAC	AGT	GGG
M6282		GAG	GTG	GAG	AAA	CAA	AGT	---	GGA	TCA	AGT	GAC	ACC	CTC	AGC	AAA	TCC	GAC	TTT	GCC	AAA	AAG	CCG	TTT	AAA	---	---	---	---	GAT	GAG
M6283		GAG	GTG	GAG	AAA	CAA	AGT	---	GGA	TCA	ACT	GAT	TCA	CTT	TTA	AAA	AAC	GAC	TTT	GCC	AAA	AAG	CCA	CTG	CAG	CAT	AAA	GAA	AAT	AGT	GGG
M6284		GAG	GTG	GAG	AAA	GGC	AGT	AAT	GTC	AAT	CAA	GGC	GAA	CTA	GCA	AAA	AAC	GAC	TTT	GCT	AAA	AAG	CCA	CTG	AAA	CAT	AAG	AAC	AGT	AGT	GGG
M6285		GAG	GTG	GAA	AGG	GGG	AGT	CAA	---	AGT	GAT	TCA	CTT	TTA	AAA	---	AAC	GAC	TTT	GCT	AAA	AAG	CCA	CTG	AAA	CAT	AAG	AAC	AGT	AGT	GGG
6286		GAG	GTG	GAA	AGG	GGG	AGT	CAA	---	AGT	GAT	TCA	CTT	TTA	AAA	---	AAC	GAC	TTT	GCC	AAA	AAG	CCA	CTG	CAG	CAT	AAA	GAA	AAT	AGT	GGG
M6320		GAG	GTG	GAA	AGG	GGA	TCA	---	---	AGT	GAC	ACC	CTA	ACC	AAA	TCC	---	GAC	TTT	GCT	AAA	AAG	CCA	CTG	AAA	CAT	AAG	AAC	AGT	AGT	GGG
64.0		GAG	GTG	GAA	AGG	GGT	AGT	AAA	GTC	AAT	CAA	GGC	GAA	CTG	CAA	TCC	AAC	GAC	TTT	GCT	AAA	AAG	CCA	CTG	AAA	CAT	AAA	GAA	AAT	AGT	GGG
64.1		GAG	GTG	GAA	AGG	GGT	AGT	AAA	GTC	AAT	CAA	GGC	GAA	CTG	CAA	TCC	AAC	GAC	TTT	GCT	AAA	AAG	CCA	CTG	AAA	CAT	AAA	GAA	AAT	AGT	GGG
199.0		GAG	GTG	GAG	AAA	CAA	AGT	---	GGA	TCA	ACT	GAT	TCA	CTT	TTA	AAA	AAC	GAC	TTT	GCT	AAA	AAG	CCG	TTT	AAA	---	---	---	---	GAT	GAG
199.1		GAG	GTG	GAG	AAA	CAA	AGT	---	GGA	TCA	ACT	GAT	TCA	CTT	TTA	AAA	AAC	GAC	TTT	GCT	AAA	AAG	CCG	TTT	AAA	---	---	---	---	GAT	GAG
KOR10139.a		GAG	GTG	GAG	AAA	CAA	AGT	---	GGA	TCA	ACT	GAT	TCA	CTT	TTA	AAA	AAC	GAC	TTT	GCC	AAA	GAG	CCA	CTG	CAG	CAT	AAA	GAA	AAT	AGT	GGG
KOR10139.b		GAG	GTG	GAG	AAA	GGC	AGT	AAA	GTC	AAT	CAA	GGC	GAA	CTA	CAA	TCC	AAC	GAC	TTT	GCC	AAA	AAG	CCG	TTT	AAA	---	---	---	---	GAT	GAG
KOR10139.c		GAG	GTG	GAG	AAA	CAA	AGT	---	GGA	TCA	ACT	GAT	TCA	CTT	TTA	AAA	AAC	GAC	TTT	GCC	AAA	AAG	CCA	CTG	CAG	CAT	AAA	GAA	AAT	AGT	GGG
KOR10139.d		GAG	GTG	GAG	AAA	GGC	AGT	AAA	GTC	AAT	CAA	GGC	GAA	CTA	CAA	TCC	AAC	GAC	TTT	GCT	AAA	AAG	CCG	TTT	AAA	---	---	---	---	GAT	GAG
KOR10139.e		GAG	GTG	GAA	AGG	GGA	TCA	---	---	ACT	GAT	TCA	CTT	TTA	AAA	---	AAC	GAC	TTT	GCC	AAA	AAG	CCA	CTG	CAG	CAT	AAA	GAA	AAT	AGT	GGG
KOR10139.f		GAG	GTG	GAG	AAA	GGC	AGT	AAA	GTC	AAT	CAA	GGC	GAA	CTA	CAA	TCC	AAC	GAC	TTT	GCT	AAA	AAG	CCG	TTT	AAA	---	---	---	---	GAT	GAG
KOR10139.g		GAG	GTG	GAG	AAA	GGC	AGT	AAA	GTC	AAT	CAA	GGC	GAA	CTA	CAA	TCC	AAC	GAC	TTT	GCT	AAA	AAG	CCG	TTT	AAA	---	---	---	---	GAT	GAG
KOR10139.h		GAG	GTG	GAG	AAA	GGC	AGT	AAA	GTC	AAT	CAA	GGC	GAA	CTA	CAA	TCC	AAC	GAC	TTT	GCT	AAA	AAG	CCG	TTT	AAA	---	---	---	---	GAT	GAG
KOR10139.j		GAG	GTG	GAG	AAA	GGC	AGT	AAA	GTC	AAT	CAA	GGC	GAA	CTA	CAA	TCC	AAC	GAC	TTT	GCC	AAA	AAG	CCG	TTT	AAA	---	---	---	---	GAT	GAG
KOR10139.k		GAG	GTG	GAG	AAA	GGC	AGT	AAA	GTC	AAT	CAA	GGC	GAA	CTA	CAA	TCC	AAC	GAC	TTT	GCC	AAA	AAG	CCG	TTT	AAA	---	---	---	---	GAT	GAG
KOR10139.l		GAG	GTG	GAG	AAA	GGC	AGT	AAA	GTC	AAT	CAA	GGC	GAA	CTA	CAA	TCC	AAC	GAC	TTT	GCT	AAA	AAG	CCG	TTT	AAA	---	---	---	---	GAT	GAG
KOR10139.m		GAG	GTG	GAG	AAA	GGC	AGT	AAA	GTC	AAT	CAA	GGC	GAA	CTA	CAA	TCC	AAC	GAC	TTT	GCT	AAA	AAG	CCG	TTT	AAA	---	---	---	---	GAT	GAG
KOR10139.n		GAG	GTG	GAG	AAA	GGC	AGT	AAA	GTC	AAT	CAA	GGC	GAA	CTA	CAA	TCC	AAC	GAC	TTT	GCT	AAA	AAG	CCG	TTT	AAA	---	---	---	---	GAT	GAG
KOR10139.o		GAG	GTG	GAG	AAA	GGC	AGT	AAA	GTC	AAT	CAA	GGC	GAA	CTA	CAA	TCC	AAC	GAC	TTT	GCC	AAA	AAG	CCG	TTT	AAA	---	---	---	---	GAT	GAG
KOR10139.p		GAG	GTG	GAG	AAA	GGC	AGT	AAA	GTC	AAT	CAA	GGC	GAA	CTA	CAA	TCC	AAC	GAC	TTT	GCC	AAA	AAG	CCG	TTT	AAA	---	---	---	---	GAT	GAG
KOR10139.q		GAG	GTG	GAG	AAA	GGC	AGT	AAA	GTC	AAT	CAA	GGC	GAA	CTA	CAA	TCC	AAC	GAC	TTT	GCC	AAA	AAG	CCG	TTT	AAA	---	---	---	---	GAT	GAG
Sea1		GAG	GTG	GAG	AAA	---	---	---	---	---	---	---	---	---	---	---	AAC	GAC	TTT	GCT	AAA	AAG	CCA	CTA	AAG	CAT	AAG	AAC	AGT	AGT	GGG
Sea2		GAG	GTG	GAA	AAA	CAA	AGT	---	GGA	TCA	ACT	GAT	TCA	CTT	TTA	AAA	AAC	GAC	TTT	GCC	AAA	AAG	CCA	CTG	AAA	CAT	AAA	GAA	AAT	AGT	GGG
		***	***	**	*													***	***	**	***	**	**	*	*					*	* *
**G37**	**280**	E	V	E	R	G	S	Q	-	S	D	S	L	L	K	-	N	D	F	A	K	K	P	L	K	H	K	N	S	S	G
					K	-	-	-	G	-	-	-	-	-	-	S	-					E		F	Q	-	-	-	-	D	E
						Q	N	S	V	N	Q	T	E	T	Q	K	S											E	N		
								K		T	S	G	T		A													D			
								N			T	D	S		S																
															L																
**Consensus**	**277**	E	V	E	K	G	S	K	V	N	Q	G	E	L	Q	S	N	D	F	A	K	K	P	L	K	H	K	-	-	S	G

**G37 922**	**---**	**GAG**	**GTG**	**AAG**	**TTA**	**GAG**	**GCA**	**GAG**	**AAG**	**GAG**	**TTT**	**---**	**---**	**ACT**	**GAG**	**GCC**	**TGA**	**AAA**	**CCA**	**TTG**	**TTG**	**ACT**	**ACT**	**GAT**	**CAA**	**ATA**	**GCA**	**AGA**	**GAG**	**AAG**
G371-2	ACA	GAG	GTG	AAG	TTG	GAT	TCA	CAG	AAG	GAT	TTT	CCC	CAA	GGA	AAG	GTT	TGA	AAA	CCG	GTG	TTG	ACT	ACT	GAT	CAA	ATA	GCA	AGA	GAG	AAG
G371-3	---	GAG	GTG	AAG	TTA	GAG	GCA	GAG	AAG	GAG	TTT	---	---	ACT	GAG	GCC	TGA	AAA	CCA	TTG	TTG	ACT	ACT	GAT	CAA	ATA	GCA	AGA	GAG	AAG
G37-vB	---	GAG	GTG	AAG	TTA	GAG	GCA	GAG	AAG	GAG	TTT	---	---	ACT	GAG	GCC	TGA	AAA	CCA	TTG	TTG	ACT	ACT	GAT	CAA	ATA	GCA	AGA	GAG	AAG
G37-DK	---	GAG	GTG	AAG	TTG	GAT	GCG	AGT	GGG	GAG	TTT	---	---	ACT	GAG	GCC	TGA	AAA	CCA	TTG	TTG	ACT	ACT	GAT	CAA	ATA	GCA	AGA	GAG	AAG
G37.A01120vB	---	GAG	GTG	AAG	TTG	GAT	GCG	AGT	GGG	GAG	TTT	---	---	ACT	GAG	GCC	TGA	AAA	CCA	TTG	TTG	ACT	ACT	GAT	CAA	ATA	GCA	AGA	GAG	AAG
M30	ACA	GAG	GTG	AAG	TTG	GAT	TCA	CAG	AAG	GAT	TTT	CCC	CAA	GGA	AAG	GTT	TGA	AAA	CCG	GTG	TTG	AAA	ACA	GAT	GAG	ATA	ACA	AGA	GAG	AGG
M2282	AGC	AAT	AAG	AAG	TTG	GAT	GCA	CAG	AAG	GAT	TTT	GCC	GGA	GAC	AAG	GCC	TGA	AAA	CCG	GTG	TTG	AAA	ACA	GAT	GAG	ATA	GAA	AAA	AAT	AGG
M2288	ACA	GAT	GTG	AAG	TTA	GAT	TCA	CAG	AAG	GAT	TTT	ACT	GAA	GGA	AAG	GTT	TGA	AAA	CCG	GTG	TTG	ACT	ACG	GAT	GAG	ATA	GAA	AAA	AAT	AGG
M2300	AGC	AAT	AAG	AAG	TTG	GAT	GCA	CAG	AAG	GAT	TTT	CCC	CAA	GGA	AAG	GTT	TGA	AAA	CCG	GTG	TTG	AAA	ACA	GAT	GAG	ATA	GAA	AAA	AAT	AGG
M2321	---	GAG	GTG	AAG	TTA	GAT	GCG	AGT	GGG	GAG	TTT	GGT	GAC	AAC	AAA	GCC	TGA	AAG	CCA	TTG	TTG	ACT	ACT	GAT	CAA	ATA	AAA	GAT	AAT	AGG
M2341	ACA	GAG	GTG	AAG	TTG	GAT	GCG	AAT	GGG	GAG	TTT	GCC	AAT	GAT	AAA	GCC	TGA	AAA	CCA	TTG	TTG	ACT	ACT	GAT	CAA	ATA	GCA	AAA	GAG	AAG
M6257	AGC	AAT	AAG	AAG	TTG	GAT	GCG	AGT	GGG	GAG	TTT	GCC	AAT	GAT	AAA	GCC	TGA	AAA	CCA	TTG	TTG	ACT	ACC	GAG	CAA	ATA	AAA	GAT	AAT	AGG
M6280	---	GAG	GTG	AAG	TTA	GAG	GCA	CAG	AAG	GAT	TTT	---	---	ACT	GAG	GCC	TGA	AAA	CCA	TTG	TTG	ACT	ACC	GAG	CAA	ATA	AAA	GAT	AAT	AGG
M6282	AGC	AAT	AAG	AAG	TTG	GAT	GCG	AGT	GGG	GAG	TTT	GCA	GGA	GAC	AAG	GCC	TGA	AAA	CCA	TTG	TTG	AAA	ACA	GAT	GAG	ATA	GCG	AAG	GAG	AAG
M6283	---	GAA	GTG	AAG	TTA	GAT	TCA	CAG	AAG	GAT	TTT	CCC	---	---	AAA	GCC	TGA	AAA	CCG	GTG	TTG	ACT	ACC	GAG	CAA	ATA	ACA	AAA	GAG	AAG
M6284	ACA	GAG	GTG	AAG	TTG	GAT	GCA	CAG	AAG	GAG	TTT	---	---	ACT	GAG	GCC	TGA	AAA	CCA	TTG	TTG	AAA	ACA	GAT	SAR	ATA	GAA	AAA	AAT	AGG
M6285	ACA	GAG	GTG	AAG	TTA	GAG	GCA	GAG	AAG	GAG	TTT	---	---	ACC	AAA	GCC	TGA	AAA	CCG	GTG	TTG	AAA	ACA	GAT	GAG	ATA	GAA	AAA	AAT	AGG
6286	ACA	GAT	GTG	AAG	TTG	GAT	GCA	GAG	AAG	GAT	TTT	---	---	ACT	GAG	GCC	TGA	AAA	CCG	GTG	TTG	ACT	ACC	GAG	CAA	ATA	GCA	AGA	GAG	AAG
M6320	ACA	GAT	GTG	AAG	TTA	GAG	GCA	GAG	AAG	GAG	TTT	---	---	ACT	GAG	GCC	TGA	AAA	CCA	TTG	TTG	ACT	ACT	GAG	CAG	ATA	AAA	GAT	AAT	AGG
64.0	ACA	GAT	GTG	AAG	TTA	GCT	GCG	AGT	GGG	GAG	TTT	GCA	GGA	GAC	AAA	GCC	TGA	AAA	CCG	GTG	TTG	AAA	ACA	GAT	GAG	ATA	GAA	AAA	AAT	AGG
64.1	ACA	GAT	GTG	AAG	TTA	GCT	GCG	AGT	GGG	GAG	TTT	GCA	GGA	GAC	AAA	GCC	TGA	AAA	CCG	GTG	TTG	AAA	ACA	GAT	GAG	ATA	GAA	AAA	AAT	AGG
199.0	AGC	AAT	AAG	AAG	TTA	GCT	GCG	AGT	GGG	GAT	TTT	CCT	---	---	GAG	GCC	TGA	AAA	CCG	GTG	TTG	AAA	ACA	GAT	GAG	ATA	ACA	AAA	GAG	AAG
199.1	AGC	AAT	AAG	AAG	TTA	GCT	GCG	AGT	GGG	GAT	TTT	CCT	---	---	GAG	GCC	TGA	AAA	CCG	GTG	TTG	AAA	ACA	GAT	GAG	ATA	ACA	AAA	GAG	AAG
KOR10139.a	ACA	GAT	GTG	AAG	TTG	GAT	GCG	AGT	GGG	GAG	TTT	GCA	GGA	GAC	AAG	GCC	TGA	AAA	CCA	TTG	TTG	ACT	ACC	GAG	CAA	ATA	AAA	GAT	AAT	AGG
KOR10139.b	AGC	AAT	AAG	AAG	TTG	GAT	GCG	AGT	GGG	GAT	TTT	CCC	CAA	GGA	AAG	GTT	TGA	AAA	CCG	GTG	TTG	AAA	ACA	GAT	GAG	ATA	GCG	AAG	GAG	AAG
KOR10139.c	ACA	GAT	GTG	AAG	TTG	GAT	GCA	CAG	AAG	GAT	TTT	CCC	CAA	GGA	AAG	GTT	TGA	AAA	CCG	GTG	TTG	AAA	ACA	GAT	GAG	ATA	GCG	AAG	GAG	AAG
KOR10139.d	AGC	AAT	AAG	AAG	TTG	GAT	GCA	CAG	AAG	GAT	TTT	CCC	CAA	GGA	AAG	GTT	TGA	AAA	CCG	GTG	TTG	AAA	ACA	GAT	GAG	ATA	GCG	AAG	GAG	AAG
KOR10139.e	ACA	GAG	GTG	AAG	TTA	GCT	GCG	AGT	GGG	GAG	TTT	GCA	GGA	GAC	AAG	GCC	TGA	AAA	CCG	GTG	TTG	AAA	ACA	GAT	GAG	ATA	GCG	AAG	GAG	AAG
KOR10139.f	AGC	AAT	AAG	AAG	TTG	GAT	GCA	CAG	AAG	GAT	TTT	CCC	CAA	GGA	AAG	GTT	TGA	AAA	CCG	GTG	TTG	AAA	ACA	GAT	GAG	ATA	GCG	AAG	GAG	AAG
KOR10139.g	AGC	AAT	AAG	AAG	TTG	GAT	GCA	CAG	AAG	GAT	TTT	CCC	CAA	GGA	AAG	GTT	TGA	AAA	CCG	GTG	TTG	AAA	ACA	GAT	GAG	ATA	GCG	AAG	GAG	AAG
KOR10139.h	AGC	AAT	AAG	AAG	TTG	GAT	GCG	AGT	GGG	GAG	TTT	GCA	GGA	GAC	AAG	GCC	TGA	AAA	CCG	GTG	TTG	AAA	ACA	GAT	GAG	ATA	GCG	AAG	GAG	AAG
KOR10139.j	AGC	AAT	AAG	AAG	TTG	GAT	GCG	AGT	GGG	GAG	TTT	GCA	GGA	GAC	AAG	GCC	TGA	AAA	CCG	GTG	TTG	AAA	ACA	GAT	GAG	ATA	GCG	AAG	GAG	AAG
KOR10139.k	AGC	AAT	AAG	AAG	TTG	GAT	GCG	AGT	GGG	GAG	TTT	GCA	GGA	GAC	AAG	GTC	TGA	AAG	CCA	TTG	TTG	ACT	ACC	GAA	CAA	ATA	AAA	GAT	AAT	AGG
KOR10139.l	AGC	AAT	AAG	AAG	TTG	GAT	GCG	AGT	GGG	GAG	TTT	GCA	GGA	GAC	AAG	GCC	TGA	AAA	CCG	GTG	TTG	AAA	ACA	GAT	GAG	ATA	GCG	AAG	GAG	AAG
KOR10139.m	AGC	AAT	AAG	AAG	TTA	GAT	GCG	AGT	GGG	GAG	TTT	GCA	GGA	GAC	AAG	GCC	TGA	AAA	CCG	GTG	TTG	AAA	ACA	GAT	GAG	ATA	GCG	AAG	GAG	AAG
KOR10139.n	AGC	AAT	AAG	AAG	TTG	GAT	GCG	AGT	GGG	GAG	TTT	GCA	GGA	GAC	AAG	GCC	TGA	AAA	CCG	GTG	TTG	AAA	ACA	GAT	GAG	ATA	GCG	AAG	GAG	AAG
KOR10139.o	AGC	AAT	AAG	AAG	TTG	GAT	GCG	AGT	GGG	GAG	TTT	CCC	CAA	GGA	AAG	GTT	TGA	AAA	CCG	GTG	TTG	AAA	ACA	GAT	GAG	ATA	GCG	AAG	GAG	AAG
KOR10139.p	AGC	AAT	AAG	AAG	TTG	GAT	GCG	AGT	GGG	GAG	TTT	GCA	GGA	GAC	AAG	GCC	TGA	AAG	CCA	TTG	TTG	ACT	ACC	GAA	CAA	ATA	AAA	GAT	AAT	AGG
KOR10139.q	AGC	AAT	AAG	AAG	TTG	GAT	GCG	AGT	GGG	GAG	TTT	GCA	GGA	GAC	AAG	GCC	TGA	AAG	CCA	TTG	TTG	ACT	ACC	GAA	CAA	ATA	AAA	GAT	AAT	AGG
Sea1	---	GAG	GTG	AAG	TTG	GAT	GCG	AGT	GGG	GAG	TTT	GGT	GAC	AAC	AAA	GCC	TGA	AAA	CCA	TTG	TTG	ACT	ACT	GAG	CAG	ATA	AAA	GAT	AAT	AGG
Sea2	---	GAA	GTG	AAG	TTA	GAG	GCA	GAG	AAG	GAT	TTT	CCC	CAA	GGA	AAG	GTT	TGA	AAA	CCG	GTG	TTG	ACT	ACG	GAC	CAA	CTT	TCA	AAA	AAT	AGA
		*	*	***	**	*	*		*	**	***				*	*	***	**	**	**	***	*	**	**	*	*			*	*
**G37 308**	**-**	**E**	**V**	**K**	**L**	**E**	**A**	**E**	**K**	**E**	**F**	**-**	**-**	**T**	**E**	**A**	**W**	**K**	**P**	**L**	**L**	**T**	**T**	**D**	**Q**	**I**	**A**	**R**	**E**	**K**
	T	N	K			D	S	Q	G	D		P	Q	-	K	V				V		K		E	E	L	T	K	N	R
	S	D				A		S				A	G	G													E	D		
								N				T	E	D													K			
												G	D	N													S			
													N																	
**Consensus 305**	S	N	V	K	L	D	A	S	G	E	F	A	G	D	K	A	W	K	P	V	L	K	T	D	E	I	A	K	E	K

**G37**	**1,103**	**GGG**	**ATG**	**GGG**	**GCG**	**ACG**
G371-2		GGG	ATG	GGG	GCG	ACG
G371-3		GGG	ATG	GGG	GCG	ACG
G37-vB		GGG	ATG	GGG	GCG	ACG
G37-DK		GGG	ATG	GGG	GCG	ACG
G37.A01120vB		GGG	ATG	GGG	GCG	ACG
M30		GGG	ATG	GGG	GCG	ACG
M2282		GGG	ATG	GGG	GCG	ACG
M2288		GGG	ATG	GGG	GCG	ACG
M2300		GGG	ATG	GGG	GCG	ACG
M2321		GGG	ATG	GGG	GCG	ACG
M2341		GGG	ATG	GGG	GCG	ACG
M6257		GGG	ATG	GGG	GCG	ACG
M6280		GGG	ATG	GGG	GCG	ACG
M6282		GGG	ATG	GGG	GCG	ACG
M6283		GGG	ATG	GGG	GCG	ACG
M6284		GGG	ATG	GGG	GCG	ACG
M6285		GAG	ATG	GGG	GCG	ACG
6286		GGG	ATG	GGG	GCG	ACG
M6320		GGG	ATG	GGG	GCG	ACG
64.0		GGG	ATG	GGG	GCG	ACG
64.1		GGG	ATG	GGG	GCG	ACG
199.0		GGG	ATG	GGG	GCG	ACG
199.1		GGG	ATG	GGG	GCG	ACG
KOR10139.a		GGG	ATG	GGG	GCG	ACG
KOR10139.b		GGG	ATG	GGG	GCG	ACG
KOR10139.c		GGG	ATG	GGG	GCG	ACG
KOR10139.d		GGG	ATG	GAG	GCG	ACG
KOR10139.e		GGG	ATG	GGG	GCG	ACG
KOR10139.f		GGG	ATG	GAG	GCG	ACG
KOR10139.g		GGG	ATG	GGG	GCG	ACG
KOR10139.h		GGG	ATG	GGG	GCG	ACG
KOR10139.j		GGG	ATG	GGG	GCG	ACG
KOR10139.k		GGG	ATG	GGG	GCG	ACG
KOR10139.l		GGG	ATG	GGG	GCG	ACG
KOR10139.m		GGG	ATG	GGG	GCG	ACG
KOR10139.n		GGG	ATG	GGG	GCG	ACG
KOR10139.o		GGG	ATG	GGG	GCG	ACG
KOR10139.p		GGG	ATG	GGG	GCG	ACG
KOR10139.q		GGG	ATG	GGG	GCG	ACG
Sea1		GGG	ATG	GGG	GCG	ACG
Sea2		GGG	ATG	GGG	GCG	ACG
		* *	***	* *	***	***
**G37**	**335**	**G**	**M**	**G**	**A**	**T**
		**E**		**E**		
**Consensus**	**335**	**G**	**M**	**G**	**A**	**T**

Conserved Region 2 (bp 1,018-2,292, aa 340-764)
25 Sequences Analyzed

**G37**	**1,018**	**GTG**	**GTT**	**AGT**	**TTC**	**TAT**	**GAT**	**GCA**	**CCC**	**TAC**	**AGT**	**GAA**	**AAC**	**CAT**	**ACT**	**GCC**	**TTT**	**GGA**	**CTT**	**GTT**	**GAT**	**CAC**	**ATC**	**GAT**	**CCT**	**AAA**	**AAG**	**ATG**	**GTT**	**GAA**	**AAC**
M30		GTG	GTT	AGT	TTC	TAT	GAT	GCA	CCC	TAC	AGT	GAA	AAC	CAT	ACT	GCC	TTT	GGA	CTT	GTT	GAT	CAC	ATC	GAT	CCT	AAA	AAG	ATG	GTT	GAA	AAC
M2282		GTG	GTT	AGT	TTC	TAT	GAT	GCA	CCC	TAC	AGT	GAA	AAC	CAT	ACT	GCC	TTT	GGA	CTT	GTT	GAT	CAC	ATC	GAT	CCT	AAA	AAG	ATG	GTT	GAA	AAC
M2288		GTG	GTT	AGT	TTC	TAT	GAT	GCA	CCC	TAC	AGT	GAA	AAC	CAT	ACT	GCC	TTT	GGA	CTT	GTT	GAT	CAC	ATC	GAT	CCT	AAA	AAG	ATG	GTT	GAA	AAC
M2300		GTG	GTT	AGT	TTC	TAT	GAT	GCA	CCC	TAC	AGT	GAA	AAC	CAT	ACT	GCC	TTT	GGA	CTT	GTT	GAT	CAC	ATC	GAT	CCT	AAA	AAG	ATG	GTT	GAA	AAC
M2321		GTG	GTT	AGT	GTC	TAT	GAT	GCA	CCC	TAC	AGC	GAA	AAC	CAT	ACT	GCC	TTT	GGG	CTT	GTT	GAT	CAC	ATC	GAT	CCT	AAA	AAA	ATG	ATT	GAG	AAC
M2341		GTG	GTT	AGT	TTC	TAT	GAT	GCA	CCC	TAC	AGT	GAA	AAC	CAT	ACT	GCC	TTT	GGA	CTT	GTT	GAT	CAC	ATC	GAT	CCT	AAA	AAG	ATG	GTT	GAA	AAC
M6257		GTG	GTT	AGT	TTC	TAT	GAT	GCA	CCC	TAC	AGT	GAA	AAC	CAT	ACT	GCC	TTT	GGA	CTT	GTT	GAT	CAC	ATC	GAT	CCT	AAA	AAG	ATG	GTT	GAA	AAC
M6280		GTG	GTT	AGT	ATC	TAT	GAT	GCG	CCC	TAC	AGT	GAA	AAC	CAT	ACT	GCC	TTT	GGA	CTT	GTT	GAT	CAT	ATT	GAT	CCT	AAA	AAG	ATG	GTT	GAA	AAC
M6282		GTG	GTT	AGT	TTC	TAT	GAT	GCA	CCC	TAC	AGT	GAA	AAC	CAT	ACT	GCC	TTT	GGA	CTT	GTT	GAT	CAC	ATC	GAT	CCT	AAA	AAG	ATG	GTT	GAA	AAC
M6283		GTG	GTT	AGT	ATC	TAT	GAT	GCG	CCC	TAC	AGT	GAA	AAC	CAT	ACT	GCC	TTT	GGA	CTT	GTT	GAT	CAC	ATC	GAT	CCT	AAA	AAG	ATG	GTT	GAA	AAC
M6284		GTG	GTT	AGT	TTC	TAT	GAT	GCA	CCC	TAC	AGT	GAA	AAC	CAT	ACT	GCC	TTT	GGA	CTT	GTT	GAT	CAC	ATC	GAT	CCT	AAA	AAG	ATG	GTT	GAA	AAC
M6285		GTG	GTT	AGT	GTC	TAT	GAT	GCA	CCC	TAC	AGC	GAA	AAC	CAT	ACT	GCC	TTT	GGG	CTT	GTT	GAT	CAC	ATC	GAT	CCT	AGA	AAA	ATG	ATT	GAG	AAC
6286		GTG	GTT	AGT	ATC	TAT	GAT	GCA	CCC	TAC	AGC	GAA	AAC	CAT	ACT	GCC	TTT	GGG	CTT	GTT	GAT	CAC	ATC	GAT	CCT	AAA	AAA	ATG	GTT	GAG	AAC
M6320		GTG	GTT	AGT	TTC	TAT	GAT	GCA	CCC	TAC	AGT	GAA	AAC	CAT	ACT	GCC	TTT	GGA	CTT	GTT	GAT	CAC	ATC	GAT	CCT	AAA	AAG	ATG	GTT	GAA	AAC
64.0		GTG	GTT	AGT	ATC	TAT	GAT	GCG	CCC	TAC	AGT	GAA	AAC	CAT	ACT	GCC	TTT	GGA	CTT	GTT	GAT	CAT	ATT	GAT	CCT	AAA	AAG	ATG	GTT	GAA	AAC
64.1		GTG	GTT	AGT	ATC	TAT	GAT	GCG	CCC	TAC	AGT	GAA	AAC	CAT	ACT	GCC	TTT	GGA	CTT	GTT	GAT	CAT	ATT	GAT	CCT	AAA	AAG	ATG	GTT	GAA	AAC
199.0		GTG	GTT	AGT	TTC	TAT	GAT	GCG	CCC	TAC	AGT	GAA	AAC	CAT	ACT	GCC	TTT	GGA	CTT	GTT	GAT	CAT	ATT	GAT	CCT	AAA	AAG	ATG	GTT	GAA	AAC
199.1		GTG	GTT	AGT	TTC	TAT	GAT	GCG	CCC	TAC	AGT	GAA	AAC	CAT	ACT	GCC	TTT	GGA	CTT	GTT	GAT	CAT	ATT	GAT	CCT	AAA	AAG	ATG	GTT	GAA	AAC
MEGA10378		GTG	GTT	AGT	TTC	TAT	GAT	GCA	CCC	TAC	AGT	GAA	AAC	CAT	ACT	GCC	TTT	GGA	CTT	GTT	GAT	CAC	ATC	GAT	CCT	AAA	AAG	ATG	GTT	GAA	AAC
MEGA10378		GTG	GTT	AGT	TTC	TAT	GAT	GCA	CCC	TAC	AGT	GAA	AAC	CAT	ACT	GCC	TTT	GGA	CTT	GTT	GAT	CAC	ATC	GAT	CCT	AAA	AAG	ATG	GTT	GAA	AAC
MEGA10467		GTG	GTT	AGT	TTC	TAT	GAT	GCA	CCC	TAC	AGT	GAA	AAC	CAT	ACT	GCC	TTT	GGA	CTT	GTT	GAT	CAC	ATC	GAT	CCT	AAA	AAG	ATG	GTT	GAA	AAC
MEGA10477		GTG	GTT	AGT	ATC	TAT	GAT	GCG	CCC	TAC	AGT	GAA	AAC	CAT	ACT	GCC	TTT	GGA	CTT	GTT	GAT	CAC	ATC	GAT	CCT	AAA	AAG	ATG	GTT	GAA	AAC
Sea1		GTG	GTT	AGT	ATC	TAT	GAT	GCG	CCC	TAC	AGT	GAA	AAC	CAT	ACT	GCC	TTT	GGA	CTT	GTT	GAT	CAT	ATT	GAT	CCT	AAA	AAG	ATG	GTT	GAA	AAC
Sea2		GTG	GTT	AGT	GTC	TAT	GAT	GCA	CCC	TAC	AGC	GAA	AAC	CAT	ACT	GCC	TTT	GGG	CTT	GTT	GAT	CAC	ATC	GAT	CCT	AAA	AAA	ATG	ATT	GAG	AAC
		***	***	***	**	***	***	**	***	***	**	***	***	***	***	***	***	**	***	***	***	**	**	***	***	* *	**	***	**	**	***
**G37**	**340**	**V**	**V**	**S**	**F**	**Y**	**D**	**A**	**P**	**Y**	**S**	**E**	**N**	**H**	**T**	**A**	**F**	**G**	**L**	**V**	**D**	**H**	**I**	**D**	**P**	**K**	**K**	**M**	**V**	**E**	**N**
					**V**																					**R**			**I**		
					**I**																										
**Consensus**	**340**	**V**	**V**	**S**	**F**	**Y**	**D**	**A**	**P**	**Y**	**S**	**E**	**N**	**H**	**T**	**A**	**F**	**G**	**L**	**V**	**D**	**H**	**I**	**D**	**P**	**K**	**K**	**M**	**V**	**E**	**N**

		1	2	3	4	5	6	7	8	9	10	11	12	13	14	15	16	17	18	19	20	21	22	23	24	25	26	27	28	29	30
**G37**	**1,108**	**TAC**	**CCA**	**CCA**	**AGT**	**TGA**	**AAG**	**ACC**	**CCG**	**AAG**	**TGA**	**AAC**	**CAC**	**CAT**	**GGG**	**ATC**	**TGG**	**GAT**	**TAC**	**AAC**	**GCA**	**AGA**	**AAC**	**CTC**	**TTG**	**TTA**	**CAA**	**ACA**	**ACA**	**GGG**	**TTC**
M30		TAC	CCA	CCA	AGT	TGA	AAG	ACC	CCG	AAG	TGA	AAC	CAA	CAT	GGG	ATC	TGG	GAT	TAC	AAC	GCA	AGA	AAC	CTC	TTG	TTA	CAA	ACA	ACA	GGG	TTC
M2282		TAC	CCA	CCA	AGT	TGA	AAG	ACC	CCG	AAG	TGA	AAC	CAC	CAT	GGG	ATC	TGG	GAT	TAC	AAC	GCA	AGA	AAC	CTC	TTG	TTA	CAA	ACA	ACA	GGG	TTC
M2288		TAC	CCA	CCA	AGT	TGA	AAG	ACC	CCG	AAG	TGA	AAC	CAC	CAT	GGG	ATC	TGG	GAT	TAC	AAC	GCA	AGA	AAC	CTC	TTG	TTA	CAA	ACA	ACA	GGG	TTC
M2300		TAC	CCA	CCA	AGT	TGA	AAG	ACC	CCG	AAG	TGA	AAC	CAC	CAT	GGG	ATC	TGG	GAT	TAC	AAC	GCA	AGA	AAC	CTC	TTG	TTA	CAA	ACA	ACA	GGG	TTC
M2321		TAC	CCA	CCA	AGC	TGA	AAA	ACC	CCC	AAG	TGA	AAC	CAC	CAT	GGG	ATC	TGG	GAT	TAC	AAC	GCA	AGA	AAC	CTC	TTG	TTA	CAA	ACA	ACA	GGG	TTC
M2341		TAC	CCA	CCA	AGT	TGA	AAG	ACC	CCG	AAG	TGA	AAC	CAC	CAT	GGG	ATC	TGG	GAT	TAC	AAC	GCA	AGA	AAC	CTC	TTG	TTA	CAA	ACA	ACA	GGG	TTC
M6257		TAC	CCA	CCA	AGT	TGA	AAG	ACC	CCG	AAG	TGA	AAC	CAC	CAT	GGG	ATC	TGG	GAT	TAC	AAC	GCA	AGA	AAC	CTC	TTG	TTA	CAA	ACA	ACA	GGG	TTC
M6280		TAC	TCA	CCA	AGT	TGA	AAG	ACC	CCG	AAG	TGA	AAC	CAC	CAT	GGG	ATC	TGG	GAT	TAC	AAC	GCA	AGA	AAC	CTC	TTG	TTA	CAA	ACA	ACA	GGG	TTC
M6282		TAC	CCA	CCA	AGT	TGA	AAG	ACC	CCG	AAG	TGA	AAC	CAC	CAT	GGG	ATC	TGG	GAT	TAC	AAC	GCA	AGA	AAC	CTC	TTG	TTA	CAA	ACA	ACA	GGG	TTC
M6283		TAC	CCA	CCA	AGT	TGA	AAG	ACC	CCG	AAG	TGA	AAC	CAC	CAT	GGG	ATC	TGG	GAT	TAC	AAC	GCA	AGA	AAC	CTC	TTG	TTA	CAA	ACA	ACA	GGG	TTC
M6284		TAC	CCA	CCA	AGT	TGA	AAG	ACC	CCG	AAG	TGA	AAC	CAC	CAT	GGG	ATC	TGG	GAT	TAC	AAC	GCA	AGA	AAC	CTC	TTG	TTA	CAA	ACA	ACA	GGG	TTC
M6285		TAC	CCA	CCA	AGC	TGA	AAA	ACC	CCC	AAG	TGA	AAC	CAC	CAT	GGG	ATC	TGG	GAT	TAC	AAC	GCA	AGA	AAC	CTC	TTG	TTA	CAA	ACA	ACA	GGG	TTC
6286		TAC	CCA	CCA	AGC	TGA	AAA	ACT	CCC	AAG	TGA	AAC	CAC	CAT	GGG	ATC	TGG	GAT	TAC	AAC	GCA	AGG	AAC	CTC	TTG	TTA	CAA	ACA	ACA	GGG	TTC
M6320		TAC	CCA	CCA	AGT	TGA	AAG	ACC	CCG	AAG	TGA	AAC	CAC	CAT	GGG	ATC	TGG	GAT	TAC	AAC	GCA	AGA	AAC	CTC	TTG	TTA	CAA	ACA	ACA	GGG	TTC
64.0		TAC	CCA	CCA	AGT	TGA	AAG	ACC	CCG	AAG	TGA	AAC	CAC	CAT	GGG	ATC	TGG	GAT	TAC	AAC	GCA	AGA	AAC	CTC	TTG	TTA	CAA	ACA	ACA	GGG	TTC
64.1		TAC	CCA	CCA	AGT	TGA	AAG	ACC	CCG	AAG	TGA	AAC	CAC	CAT	GGG	ATC	TGG	GAT	TAC	AAC	GCA	AGA	AAC	CTC	TTG	TTA	CAA	ACA	ACA	GGG	TTC
199.0		TAC	CCA	CCA	AGT	TGA	AAG	ACC	CCG	AAG	TGA	AAC	CAC	CAT	GGG	ATC	TGG	GAT	TAC	AAC	GCA	AGA	GAC	CTC	TTG	TTA	CAA	ACA	ACA	GGG	TTC
199.1		TAC	CCA	CCA	AGT	TGA	AAG	ACC	CCG	AAG	TGA	AAC	CAC	CAT	GGG	ATC	TGG	GAT	TAC	AAC	GCA	AGA	GAC	CTC	TTG	TTA	CAA	ACA	ACA	GGG	TTC
MEGA10378		TAC	CCA	CCA	AGT	TGA	AAG	ACC	CCG	AAG	TGA	AAC	CAC	CAT	GGG	ATC	TGG	GAT	TAC	AAC	GCA	AGA	AAC	CTC	TTG	TTA	CAA	ACA	ACA	GGG	TTC
MEGA10378		TAC	CCA	CCA	AGT	TGA	AAG	ACC	CCG	AAG	TGA	AAC	CAC	CAT	GGG	ATC	TGG	GAT	TAC	AAC	GCA	AGA	AAC	CTC	TTG	TTA	CAA	ACA	ACA	GGG	TTC
MEGA10467		TAC	CCA	CCA	AGT	TGA	AAG	ACC	CCG	AAG	TGA	AAC	CAC	CAT	GGG	ATC	TGG	GAT	TAC	AAC	GCA	AGA	AAC	CTC	TTG	TTA	CAA	ACA	ACA	GGG	TTC
MEGA10477		TAC	CCA	CCA	AGT	TGA	AAG	ACC	CCG	AAG	TGA	AAC	CAC	CAT	GGG	ATC	TGG	GAT	TAC	AAC	GCA	AGA	AAC	CTC	TTG	TTA	CAA	ACA	ACA	GGG	TTC
Sea1		TAC	CCA	CCA	AGT	TGA	AAG	ACC	CCG	AAG	TGA	AAC	CAC	CAT	GGG	ATC	TGG	GAT	TAC	AAC	GCA	AGA	AAC	CTC	TTG	TTA	CAA	ACA	ACA	GGG	TTC
Sea2		TAC	CCA	CCA	AGC	TGA	AAA	ACC	CCC	AAG	TGA	AAC	CAC	CAT	GGG	ATC	TGG	GAT	TAC	AAC	GCA	AGA	AAC	CTC	TTG	TTA	CAA	ACA	ACA	GGG	TTC
		***	**	***	**	***	**	**	**	***	***	***	**	***	***	***	***	***	***	***	***	**	**	***	***	***	***	***	***	***	**
**G37**	**370**	**Y**	**P**	**P**	**S**	**W**	**K**	**T**	**P**	**K**	**W**	**N**	**H**	**H**	**G**	**I**	**W**	**D**	**Y**	**N**	**A**	**R**	**N**	**L**	**L**	**L**	**Q**	**T**	**T**	**G**	**F**
			**S**										**Q**										**D**								
**Consensus**	**370**	**Y**	**P**	**P**	**S**	**W**	**K**	**T**	**P**	**K**	**W**	**N**	**H**	**H**	**G**	**I**	**W**	**D**	**Y**	**N**	**A**	**R**	**N**	**L**	**L**	**L**	**Q**	**T**	**T**	**G**	**F**

**G37**	**1,198**	**TTT**	**AAC**	**CCA**	**AGA**	**AGA**	**CAC**	**CCG**	**GAG**	**TGG**	**TTT**	**GAT**	**GAA**	**GGA**	**CAA**	**GCT**	**AAG**	**GCA**	**GAT**	**AAC**	**ACT**	**AGC**	**CCT**	**GGC**	**TTT**	**AAG**	**GTA**	**GGG**	**GAT**	**ACT**	**GAT**
M30		TTT	AAC	CCA	AGA	AGA	CAC	CCG	GAG	TGG	TTT	GAT	GAA	GGA	CAA	GCT	AAG	GCA	GAT	AAC	ACT	AGC	CCT	GGC	TTT	AAG	GTA	GGG	GAT	ACT	GAT
M2282		TTT	AAC	CCA	AGA	AGA	CAC	CCG	GAG	TGG	TTT	GAT	GAA	GGA	CAA	GCT	AAG	GCA	GAT	AAC	ACT	AGC	CCT	GGC	TTT	AAG	GTA	GGG	GAT	ACT	GAT
M2288		TTT	AAC	CCA	AGA	AGA	CAC	CCA	GAG	TGG	TTT	GAT	GAA	GGA	CAA	GCT	AAG	GCA	GAT	AAC	ACT	AGC	CCT	GGC	TTT	AAG	GTA	GGG	GAA	AGT	GAT
M2300		TTT	AAC	CCA	AGA	AGA	CAC	CCG	GAG	TGG	TTT	GAT	GAA	GGA	CAA	GCT	AAG	GCA	GAT	AAC	ACT	AGC	CCT	GGC	TTT	AAG	GTA	GGG	GAT	ACT	GAT
M2321		TTT	AAT	CCA	AGA	AGA	CAC	CCT	GAG	TGG	TTT	GAT	GAA	GGG	CAA	GCT	AAG	GCA	GAT	AAC	ACT	AGC	CCT	GGC	TTT	AAG	GTA	GGG	GAA	AAT	GAT
M2341		TTT	AAC	CCA	AGA	AGA	CAC	CCA	GAG	TGG	TTT	GAT	GAA	GGA	CAA	GCT	AAG	GCA	GAT	AAC	ACT	AGC	CCT	GGC	TTT	AAG	GTA	GGG	GAA	AGT	GAT
M6257		TTT	AAC	CCA	AGA	AGA	CAC	CCA	GAG	TGG	TTT	GAT	GAA	GGA	CAA	GCT	AAG	GCA	GAT	AAC	ACT	AGC	CCT	GGC	TTT	AAG	GTA	GGG	GAT	ACT	GAT
M6280		TTT	AAC	CCA	AGA	AGA	CAC	CCA	GAG	TGG	TTT	GAT	GAA	GGA	CAA	GCT	AAG	GCA	GAT	AAC	ACT	AGC	CCT	GGC	TTT	AAG	GTA	GGG	GAA	AGT	GAT
M6282		TTT	AAC	CCA	AGA	AGA	CAC	CCA	GAG	TGG	TTT	GAT	GAA	GGA	CAA	GCT	AAG	GCA	GAT	AAC	ACT	AGC	CCT	GGC	TTT	AAG	GTA	GGG	GAA	AGT	GAT
M6283		TTT	AAC	CCA	AGA	AGA	CAC	CCA	GAG	TGG	TTT	GAT	GAA	GGA	CAA	GCT	AAG	GCA	GAT	AAC	ACT	AGC	CCT	GGC	TTT	AAG	GTA	GGG	GAA	AGT	GAT
M6284		TTT	AAC	CCA	AGA	AGA	CAC	CCG	GAG	TGG	TTT	GAT	GAA	GGA	CAA	GCT	AAG	GCA	GAT	AAC	ACT	AGC	CCT	GAC	TTT	AAG	GTA	GGG	AAT	ACT	GAT
M6285		TTT	AAT	CCA	AGA	AGA	CAC	CCT	GAG	TGG	TTT	GAT	GAA	GGG	CAA	GCT	AAG	GCA	GAT	AAC	ACT	AGC	CCT	GGC	TTT	AAG	GTA	GGG	GAA	AAT	GAT
6286		TTT	AAC	CCA	AGA	AGA	CAC	CCG	GAG	TGG	TTT	GAT	GAA	GGG	CAA	GCT	AAG	GCA	GAT	AAC	ACT	AGC	CCT	GGC	TTT	AAG	GTA	GGG	GAA	AGT	GAT
M6320		TTT	AAC	CCA	AGA	AGA	CAC	CCA	GAG	TGG	TTT	GAT	GAA	GGA	CAA	GCT	AAG	GCA	GAT	AAC	ACT	AGC	CCT	GGC	TTT	AAG	GTA	GGG	GAA	AGT	GAT
64.0		TTT	AAC	CCA	AGA	AGA	CAC	CCA	GAG	TGG	TTT	GAT	GAA	GGA	CAA	GCT	AAG	GCA	GAT	AAC	ACT	AGC	CCT	GGC	TTT	AAG	GTA	GGG	GAA	AGT	GAT
64.1		TTT	AAC	CCA	AGA	AGA	CAC	CCA	GAG	TGG	TTT	GAT	GAA	GGA	CAA	GCT	AAG	GCA	GAT	AAC	ACT	AGC	CCT	GGC	TTT	AAG	GTA	GGG	GAA	AGT	GAT
199.0		TTT	AAC	CCA	AGA	AGA	CAC	CCA	GAG	TGG	TTT	GAT	GAA	GGA	CAA	GCT	AAG	GCA	GAT	AAC	ACT	AGC	CCT	GGC	TTT	AAG	GTA	GGG	GAA	AGT	GAT
199.1		TTT	AAC	CCA	AGA	AGA	CAC	CCA	GAG	TGG	TTT	GAT	GAA	GGA	CAA	GCT	AAG	GCA	GAT	AAC	ACT	AGC	CCT	GGC	TTT	AAG	GTA	GGG	GAA	AGT	GAT
MEGA10366		TTT	AAC	CCA	AGA	AGA	CAC	CCG	GAG	TGG	TTT	GAT	GAA	GGG	CAA	GCT	AAG	GCA	GAT	AAC	ACT	AGC	CCT	GGC	TTT	AAG	GTA	GGG	GAA	AGT	GAT
MEGA10378		TTT	AAC	CCA	AGA	AGA	CAC	CCG	GAG	TGG	TTT	GAT	GAA	GGA	CAA	GCT	AAG	GCA	GAT	AAC	ACT	AGC	CCT	GGC	TTT	AAG	GTA	GGG	GAT	ACT	GAT
MEGA10467		TTT	AAC	CCA	AGA	AGA	CAC	CCA	GAG	TGG	TTT	GAT	GAA	GGA	CAA	GCT	AAG	GCA	GAT	AAC	ACT	AGC	CCT	GGC	TTT	AAG	GTA	GGG	GAA	AGT	GAT
MEGA10477		TTT	AAC	CCA	AGA	AGA	CAC	CCA	GAG	TGG	TTT	GAT	GAA	GGA	CAA	GCT	AAG	GCA	GAT	AAC	ACT	AGC	CCT	GGC	TTT	AAG	GTA	GGG	GAA	AGT	GAT
Sea1		TTT	AAC	CCA	AGA	AGA	CAC	CCA	GAG	TGG	TTT	GAT	GAA	GGA	CAA	GCT	AAG	GCA	GAT	AAC	ACT	AGC	CCT	GGC	TTT	AAG	GTA	GGG	GAA	AGT	GAT
Sea2		TTT	AAT	CCA	AGA	AGA	CAC	CCT	GAG	TGG	TTT	GAT	GAA	GGG	CAA	GCT	AAG	GCA	GAT	AAC	ACT	AGC	CCT	GGC	TTT	AAG	GTA	GGG	GAA	AAT	GAT
		***	**	***	***	***	***	**	***	***	***	***	***	**	***	***	***	***	***	***	***	***	***	* *	***	***	***	***	*	* *	***
**G37**	**400**	**F**	**N**	**P**	**R**	**R**	**H**	**P**	**E**	**W**	**F**	**D**	**E**	**G**	**Q**	**A**	**K**	**A**	**D**	**N**	**T**	**S**	**P**	**G**	**F**	**K**	**V**	**G**	**D**	**T**	**D**
																								**D**					**E**	**S**	
																													**N**	**N**	

**Consensus**	**400**	**F**	**N**	**P**	**R**	**R**	**H**	**P**	**E**	**W**	**F**	**D**	**E**	**G**	**Q**	**A**	**K**	**A**	**D**	**N**	**T**	**S**	**P**	**G**	**F**	**K**	**V**	**G**	**E**	**S**	**D**

**G37**	**1,288**	**CAC**	**AAA**	**AAA**	**GAC**	**GGG**	**TTT**	**AAA**	**AAA**	**AAC**	**TCT**	**TCT**	**TCT**	**---**	**---**	**---**	**---**	**---**	**---**	**CCA**	**ATA**	**GCT**	**TTA**	**CCA**	**TTT**	**GAA**	**GCA**	**TAC**	**TTT**	**GCT**	**AAC**
M30		CAC	AAA	AAA	GAC	GGG	TTT	AAA	GAA	AAC	TCT	TCT	TCT	---	---	---	---	---	---	CCA	ATA	GCT	TTA	CCA	TTT	GAA	GCA	TAC	TTT	GCT	AAC
M2282		TAC	AAA	AAA	GAC	GGG	TTT	AAA	GAA	AAC	TCT	TCT	TCT	---	---	---	---	---	---	CCA	ATA	GCT	TTA	CCA	TTT	GAA	GCA	TAC	TTT	GCT	AAC
M2288		CAC	AAA	AAA	GAC	GGC	TTT	AAA	GAC	---	TCT	TCT	TCT	TCT	TCT	TCT	TCT	---	---	CCA	ATA	GCT	TTA	CCA	TTT	GAA	GCA	TAC	TTT	GCT	AAC
M2300		CAC	AAA	AAA	GAC	GGG	TTT	AAA	GAA	AAC	TCT	TCT	TCT	---	---	---	---	---	---	CCA	ATA	GCT	TTA	CCA	TTT	GAA	GCA	TAC	TTT	GCT	AAC
M2321		CAC	AAA	AAA	GAC	GGC	TTT	AAA	GAC	---	TCT	TCT	TCT	TCT	TCT	TCT	TCT	TCT	TCT	CCA	ATA	GCT	TTA	CCA	TTT	GAA	GCA	TAC	TTT	GCT	AAC
M2341		CAC	AAA	AAA	GAC	GGC	TTT	AAA	GAC	---	TCT	TCT	TCT	TCT	TCT	TCT	TCT	---	---	CCA	ATA	GCT	TTA	CCA	TTT	GAA	GCA	TAC	TTT	GCT	AAC
M6257		CAC	AAA	AAA	GAC	GGG	TTT	AAA	GAA	AAC	TCT	TCT	TCT	TCT	TCT	---	---	---	---	CCA	ATA	GCT	TTA	CCA	TTT	GAA	GCA	TAC	TTT	GCT	AAC
M6280		CAC	AAA	AAA	GAC	GGC	TTT	AAA	GAC	---	TCT	TCT	TCT	TCT	TCT	TCT	TCT	---	---	CCA	ATA	GCT	TTA	CCA	TTT	GAA	GCA	TAC	TTT	GCT	AAC
M6282		CAC	AAA	AAA	GAC	GGC	TTT	AAA	GAC	---	TCT	TCT	TCT	TCT	TCT	TCT	---	---	---	CCA	ATA	GCT	TTA	CCA	TTT	GAA	GCA	TAC	TTT	GCT	AAC
M6283		CAC	AAA	AAA	GAC	GGC	TTT	AAA	GAC	---	TCT	TCT	TCT	TCT	TCT	---	---	---	---	CCA	ATA	GCT	TTA	CCA	TTT	GAA	GCA	TAC	TTT	GCT	AAC
M6284		CAC	AAA	AAA	GAC	GGG	TTT	AAA	GAA	AAC	TCT	TCT	TCT	---	---	---	---	---	---	CCA	ATA	GCT	TTA	CCA	TTT	GAA	GCA	TAC	TTT	GCT	AAC
M6285		CAC	AAA	AAA	GAC	GGC	TTT	AAA	GAC	---	TCT	TCT	TCT	TCT	TCT	---	---	---	---	CCA	ATA	GCT	TTA	CCA	TTT	GAA	GCA	TAC	TTT	GCT	AAC
6286		CAC	AAA	AAA	GAC	GGC	TTT	AAA	GAC	---	TCT	TCT	TCT	TCT	TCT	TCT	TCT	TCT	TCT	CCA	ATA	GCT	TTA	CCA	TTT	GAA	GCA	TAC	TTT	GCT	AAC
M6320		CAC	AAA	AAA	GAC	GGC	TTT	AAA	AAA	GAC	TCT	TCT	TCT	---	---	---	---	---	---	CCA	ATA	GCT	TTA	CCA	TTT	GAA	GCA	TAC	TTT	GCT	AAC
64.0		CAC	AAA	AAA	GAC	GGC	TTT	AAA	GAC	---	TCT	TCT	TCT	TCT	TCT	TCT	---	---	---	CCA	ATA	GCT	TTA	CCA	TTT	GAA	GCA	TAC	TTT	GCT	AAC
64.1		CAC	AAA	AAA	GAC	GGC	TTT	AAA	GAC	---	TCT	TCT	TCT	TCT	TCT	TCT	---	---	---	CCA	ATA	GCT	TTA	CCA	TTT	GAA	GCA	TAC	TTT	GCT	AAC
199.0		CAC	AAA	AAA	GAC	GGC	TTT	AAA	GAC	---	TCT	TCT	TCT	TCT	TCT	TCT	TCT	---	---	CCA	ATA	GCT	TTA	CCA	TTT	GAA	GCA	TAC	TTT	GCT	AAC
199.1		CAC	AAA	AAA	GAC	GGC	TTT	AAA	GAC	---	TCT	TCT	TCT	TCT	TCT	TCT	---	---	---	CCA	ATA	GCT	TTA	CCA	TTT	GAA	GCA	TAC	TTT	GCT	AAC
MEGA10366		CAC	AAA	AAA	GAC	GGC	TTT	AAA	GAC	---	TCT	TCT	TCT	TCT	TCT	TCT	TCT	---	---	CCA	ATA	GCT	TTA	CCA	TTT	GAA	GCA	TAC	TTT	GCT	AAC
MEGA10378		CAC	AAA	AAA	GAC	GGG	TTT	AAA	GAA	AAC	TCT	TCT	TCT	---	---	---	---	---	---	CCA	ATA	GCT	TTA	CCA	TTT	GAA	GCA	TAC	TTT	GCT	AAC
MEGA10467		CAC	AAA	AAA	GAC	GGC	TTT	AAA	GAC	---	TCT	TCT	TCT	TCT	TCT	---	---	---	---	CCA	ATA	GCT	TTA	CCA	TTT	GAA	GCA	TAC	TTT	GCT	AAT
MEGA10477		CAC	AAA	AAA	GAC	GGC	TTT	AAA	GAC	---	TCT	TCT	TCT	TCT	TCT	TCT	TCT	---	---	CCA	ATA	GCT	TTA	CCA	TTT	GAA	GCA	TAC	TTT	GCT	AAT
Sea1		CAC	AAA	AAA	GAC	GGC	TTT	AAA	GAC	---	TCT	TCT	TCT	TCT	TCT	TCT	---	---	---	CCA	ATA	GCT	TTA	CCA	TTT	GAA	GCA	TAC	TTT	GCT	AAT
Sea2		CAC	AAA	AAA	GAC	GGC	TTT	AAA	GAC	---	TCT	TCT	TCT	TCT	TCT	TCT	TCT	---	---	CCA	ATA	GCT	TTA	CCA	TTT	GAA	GCA	TAC	TTT	GCT	AAC
		**	***	***	***	**	***	***	*		***	***	***							***	***	***	***	***	***	***	***	***	***	***	**
**G37**	**430**	**H**	**K**	**K**	**D**	**G**	**F**	**K**	**K**	**N**	**S**	**S**	**S**	**-**	**-**	**-**	**-**	**-**	**-**	**P**	**I**	**A**	**L**	**P**	**F**	**E**	**A**	**Y**	**F**	**A**	**N**
		**Y**							**E**	**-**				**S**	**S**	**S**	**S**	**S**	**S**												
									**D**	**D**																					
**Consensus**	**430**	**H**	**K**	**K**	**D**	**G**	**F**	**K**	**D**	**-**	**S**	**S**	**S**	**S**	**S**	**S**	**-**	**-**	**-**	**P**	**I**	**A**	**L**	**P**	**F**	**E**	**A**	**Y**	**F**	**A**	**N**

**G37**	**1,360**	**ATT GGT AAC ATG GTT GCT ATT GGT AAC TCG GTA TTT ATC TTT GGT GGT AAT GGT CAT GCT ACT AAG ATG TTT ACC ACC AAT CCC TTA AGT**
M30		ATT GGT AAC ATG GTT GCT ATT GGT AAC TCG GTA TTT ATC TTT GGT GGT AAT GGT CAT GCT ACT AAG ATG TTT ACC ACC AAT CCC TTA AGT
M2282		ATT GGT AAC ATG GTT GCT ATT GGT AAC TCG GTA TTT ATC TTT GGT GGT AAT GGT CAT GCT ACT AAG ATG TTT ACC ACC AAT CCC TTA AGT
M2288		ATT GGT AAC ATG GTT GCT ATT GGC AAC TCG GTA TTT ATC TTT GGT GGT AAT GGT CAT GCT ACT AAG ATG TTT ACC ACC AAT CCC TTA AGT
M2300		ATT GGT AAC ATG GTT GCT ATT GGT AAC TCG GTA TTT ATC TTT GGT GGT AAT GGT CAT GCT ACT AAG ATG TTT ACC ACC AAT CCC TTA AGT
M2321		ATT GGT AAC ATG GTT GCT ATT GGC AAC TCA GTA TTT ATC TTT GGT GGT AAT GGT CAT GCT ACT AAG ATG TTT ACC ACT AAT CCC TTA AGT
M2341		ATT GGT AAC ATG GTT GCT ATT GGC AAC TCG GTA TTT ATC TTT GGT GGT AAT GGT CAT GCT ACT AAG ATG TTT ACC ACC AAT CCC TTA AGT
M6257		ATT GGT AAC ATG GTT GCT ATT GGT AAC TCG GTA TTT ATC TTT GGC GGT AAT GGT CAT GCT ACT AAG ATG TTT ACC ACC AAT CCC TTA AGT
M6280		ATT GGT AAC ATG GTT GCT ATT GGC AAC TCG GTA TTT ATC TTT GGT GGT AAT GGT CAT GCT ACT AAG ATG TTT ACC ACC AAT CCC TTA AGT
M6282		ATT GGT AAC ATG GTT GCT ATT GGC AAC TCG GTA TTT ATC TTT GGT GGT AAT GGT CAT GCT ACT AAG ATG TTT ACC ACC AAT CCC TTA AGT
M6283		ATT GGT AAC ATG GTT GCT ATT GGC AAC TCG GTA TTT ATC TTT GGT GGT AAT GGT CAT GCT ACT AAG ATG TTT ACC ACT AAT CCC TTA AGT
M6284		ATT GGT AAC ATG GTT GCT ATT GGT AAC TCG GTA TTT ATC TTT GGT GGT AAT GGT CAT GCT ACT AAG ATG TTT ACC ACC AAT CCC TTA AGT
M6285		ATT GGT AAC ATG GTT GCT ATT GGC AAC TCA GTA TTT ATC TTT GGT GGT AAT GGT CAT GCT ACT AAG ATG TTT ACC ACT AAT CCC TTA AGT
6286		ATT GGT AAC ATG GTT GCT ATT GGC AAC TCA GTA TTT ATC TTT GGT GGT AAT GGT CAT GCT ACT AAG ATG TTT ACC ACT AAT CCC TTA AGT
M6320		ATT GGT AAC ATG GTT GCT ATT GGC AAC TCG GTA TTT ATC TTT GGT GGT AAT GGT CAT GCT ACT AAG ATG TTT ACC ACC AAT CCC TTA AGT
64.0		ATT GGT AAC ATG GTT GCT ATT GGC AAC TCG GTA TTT ATC TTT GGT GGT AAT GGT CAT GCT ACT AAG ATG TTT ACC ACC AAT CCC TTA AGT
64.1		ATT GGT AAC ATG GTT GCT ATT GGC AAC TCG GTA TTT ATC TTT GGT GGT AAT GGT CAT GCT ACT AAG ATG TTT ACC ACC AAT CCC TTA AGT
199.0		ATT GGT AAC ATG GTT GCT ATT GGC AAC TCG GTA TTT ATC TTT GGT GGT AAT GGT CAT GCT ACT AAG ATG TTT ACC ACC AAT CCC TTA AGT
199.1		ATT GGT AAC ATG GTT GCT ATT GGC AAC TCG GTA TTT ATC TTT GGT GGT AAT GGT CAT GCT ACT AAG ATG TTT ACC ACC AAT CCC TTA AGT
MEGA10366		ATT GGT AAC ATG GTT GCT ATT GGC AAC TCA GTA TTT ATC TTT GGT GGT AAT GGT CAT GCT ACT AAG ATG TTT ACC ACT AAT CCC TTA AGT
MEGA10378		ATT GGT AAC ATG GTT GCT ATT GGT AAC TCG GTA TTT ATC TTT GGT GGT AAT GGT CAT GCT ACT AAG ATG TTT ACC ACC AAT CCC TTA AGT
MEGA10467		ATT GGT AAC ATG GTT GCT ATT GGC AAC TCG GTA TTT ATC TTT GGT GGT AAT GGT CAT GCT ACT AAG ATG TTT ACC ACC AAT CCC TTA AGT
MEGA10477		ATT GGT AAC ATG GTT GCT ATT GGC AAC TCG GTA TTT ATC TTT GGT GGT AAT GGT CAT GCT ACT AAG ATG TTT ACC ACT AAT CCC TTA AGT
Sea1		ATT GGT AAC ATG GTT GCT ATT GGC AAC TCG GTA TTT ATC TTT GGT GGT AAT GGT CAT GCT ACT AAG ATG TTT ACC ACC AAT CCC TTA AGT
Sea2		ATT GGT AAC ATG GTT GCT ATT GGC AAC TCA GTA TTT ATC TTT GGT GGT AAT GGT CAT GCT ACT AAG ATG TTT ACC ACT AAT CCC TTA AGT
		*** *** *** *** *** *** *** ** *** ** *** *** *** *** ** *** *** *** *** *** *** *** *** *** *** ** *** *** *** ***
**G37**	**454**	**I G N M V A I G N S V F I F G G N G H A T K M F T T N P L S**
**Consensus**	**456**	**I G N M V A I G N S V F I F G G N G H A T K M F T T N P L S**

**G37**	**1,450**	**ATT**	**GGG**	**GTA**	**TTT**	**AGG**	**ATT**	**AAA**	**TAC**	**ACT**	**GAT**	**AAC**	**TTT**	**AGT**	**AAG**	**TCA**	**TCA**	**GTA**	**ACA**	**GGT**	**TGA**	**CCA**	**TAT**	**GCA**	**GTG**	**TTA**	**TTT**	**GGG**	**GGA**	**TTA**	**ATT**
M30		ATT	GGG	GTA	TTT	AGG	ATT	AAA	TAC	ACT	GAT	AAC	TTT	AGT	AAG	TCA	TCA	GTA	ACA	GGT	TGA	CCA	TAT	GCA	GTG	TTA	TTT	GGG	GGA	TTA	ATT
M2282		ATT	GGG	GTA	TTT	AGG	ATT	AAA	TAC	ACT	GAT	AAC	TTT	AGT	AAG	TCA	TCA	GTA	ACA	GGT	TGA	CCA	TAT	GCA	GTG	TTA	TTT	GGG	GGA	TTA	ATT
M2288		ATT	GGG	GTA	TTT	AGG	ATT	AAA	TAC	ACT	GAT	AAC	TTT	AGT	AAG	TCA	TCA	GTA	ACT	GGT	TGA	CCA	TAT	GCA	GTG	TTA	TTT	GGA	GGA	TTA	ATT
M2300		ATT	GGG	GTA	TTT	AGG	ATT	AAA	TAC	ACT	GAT	AAC	TTT	AGT	AAG	TCA	TCA	GTA	ACA	GGT	TGA	CCA	TAT	GCA	GTG	TTA	TTT	GGG	GGA	TTA	ATT
M2321		ATT	GGG	GTA	TTT	AGG	ATT	AAA	TAC	ACT	GAT	AAC	TTT	AGT	AAG	TCA	TCA	GTA	ATA	GGT	TGA	CCA	TAT	GCA	GTG	TTA	TTT	GGG	GGA	TTA	ATT
M2341		ATT	GGG	GTA	TTT	AGG	ATT	AAA	TAC	ACT	GAT	AAC	TTT	AGT	AAG	TCA	TCA	GTA	ACT	GGT	TGA	CCA	TAT	GCA	GTG	TTA	TTT	GGA	GGA	TTA	ATT
M6257		ATT	GGG	GTA	TTT	AGG	ATT	AAA	TAC	ACT	GAT	AAC	TTT	AGT	AAG	TCA	TCA	GTA	ACT	GGT	TGA	CCA	TAT	GCA	GTG	TTA	TTT	GGA	GGA	TTA	ATT
M6280		ATT	GGG	GTA	TTT	AGG	ATT	AAA	TAC	ACT	GAT	AAC	TTT	AGT	AAG	TCA	TCA	GTA	ACT	GGT	TGA	CCA	TAT	GCA	GTG	TTA	TTT	GGA	GGA	TTA	ATT
M6282		ATT	GGG	GTA	TTT	AGG	ATT	AAA	TAC	ACT	GAT	AAC	TTT	AGT	AAG	TCA	TCA	GTA	ACT	GGT	TGA	CCA	TAT	GCA	GTG	TTA	TTT	GGA	GGA	TTA	ATT
M6283		ATT	GGG	GTA	TTT	AGG	ATT	AAA	TAC	ACT	GAT	AAC	TTT	AGT	AAG	TCA	TCA	GTA	ACA	GGT	TGA	CCA	TAT	GCA	GTG	TTA	TTT	GGG	GGA	TTA	ATT
M6284		ATT	GGG	GTA	TTT	AGG	ATT	AAA	TAC	ACT	GAT	AAC	TTT	AGT	AAG	TCA	TCA	GTA	ACA	GGT	TGA	CCA	TAT	GCA	GTG	TTA	TTT	GGG	GGA	TTA	ATT
M6285		ATT	GGG	GTA	TTT	AGG	ATT	AAA	TAC	ACT	GAT	AAC	TTT	AGT	AAG	TCA	TCA	GTA	ATA	GGT	TGA	CCA	TAT	GCA	GTG	TTA	TTT	GGG	GGA	TTA	ATT
6286		ATT	GGG	GTA	TTT	AGG	ATT	AAA	TAC	ACT	GAT	AAC	TTT	AGT	AAG	TCA	TCA	GTA	ACA	GGT	TGA	CCA	TAT	GCA	GTG	TTA	TTT	GGG	GGA	TTA	ATT
M6320		ATT	GGG	GTA	TTT	AGG	ATT	AAA	TAC	ACT	GAT	AAC	TTT	AGT	AAG	TCA	TCA	GTA	ACT	GGT	TGA	CCA	TAT	GCA	GTG	TTA	TTT	GGA	GGA	TTA	ATT
64.0		ATT	GGG	GTA	TTT	AGG	ATT	AAA	TAC	ACT	GAT	AAC	TTT	AGT	AAG	TCA	TCA	GTA	ACT	GGT	TGA	CCA	TAT	GCA	GTG	TTA	TTT	GGA	GGA	TTA	ATT
64.1		ATT	GGG	GTA	TTT	AGG	ATT	AAA	TAC	ACT	GAT	AAC	TTT	AGT	AAG	TCA	TCA	GTA	ACT	GGT	TGA	CCA	TAT	GCA	GTG	TTA	TTT	GGA	GGA	TTA	ATT
199.0		ATT	GGG	GTA	TTT	AGG	ATT	AAA	TAC	ACT	GAT	AAC	TTT	AGT	AAG	TCA	TCA	GTA	ACT	GGT	TGA	CCA	TAT	GCA	GTG	TTA	TTT	GGA	GGA	TTA	ATT
199.1		ATT	GGG	GTA	TTT	AGG	ATT	AAA	TAC	ACT	GAT	AAC	TTT	AGT	AAG	TCA	TCA	GTA	ACT	GGT	TGA	CCA	TAT	GCA	GTG	TTA	TTT	GGA	GGA	TTA	ATT
MEGA10366		ATT	GGG	GTA	TTT	AGG	ATT	AAA	TAC	ACT	GAT	AAC	TTT	AGT	AAG	TCA	TCA	GTA	ACA	GGT	TGA	CCA	TAT	GCA	GTG	TTA	TTT	GGG	GGA	TTA	ATT
MEGA10378		ATT	GGG	GTA	TTT	AGG	ATT	AAA	TAC	ACT	GAT	AAC	TTT	AGT	AAG	TCA	TCA	GTA	ACA	GGT	TGA	CCA	TAT	GCA	GTG	TTA	TTT	GGG	GGA	TTA	ATT
MEGA10467		ATT	GGG	GTA	TTT	AGG	ATT	AAA	TAC	ACT	GAT	AAC	TTT	AGT	AAG	TCA	TCA	GTA	ACT	GGT	TGA	CCA	TAT	GCA	GTG	TTA	TTT	GGA	GGA	TTA	ATT
MEGA10477		ATT	GGG	GTA	TTT	AGG	ATT	AAA	TAC	ACT	GAT	AAC	TTT	AGT	AAG	TCA	TCA	GTA	ACA	GGT	TGA	CCA	TAT	GCA	GTG	TTA	TTT	GGG	GGA	TTA	ATT
Sea1		ATT	GGG	GTA	TTT	AGG	ATT	AAA	TAC	ACT	GAT	AAC	TTT	AGT	AAG	TCA	TCA	GTA	ACT	GGT	TGA	CCA	TAT	GCA	GTG	TTA	TTT	GGA	GGA	TTA	ATT
Sea2		ATT	GGG	GTA	TTT	AGG	ATT	AAA	TAC	ACT	GAT	AAC	TTT	AGT	AAG	TCA	TCA	GTA	ATA	GGT	TGA	CCA	TAT	GCG	GTG	TTA	TTT	GGG	GGA	TTA	ATT
		***	***	***	***	***	***	***	***	***	***	***	***	***	***	***	***	***	*	***	***	***	***	**	***	***	***	**	***	***	***
**G37**	**484**	**I**	**G**	**V**	**F**	**R**	**I**	**K**	**Y**	**T**	**D**	**N**	**F**	**S**	**K**	**S**	**S**	**V**	**T**	**G**	**W**	**P**	**Y**	**A**	**V**	**L**	**F**	**G**	**G**	**L**	**I**
																			**I**												

**Consensus**	**486**	**I**	**G**	**V**	**F**	**R**	**I**	**K**	**Y**	**T**	**D**	**N**	**F**	**S**	**K**	**S**	**S**	**V**	**T**	**G**	**W**	**P**	**Y**	**A**	**V**	**L**	**F**	**G**	**G**	**L**	**I**

```
G37       1,540  AAT CCC CAA ACC AAT GGC TTG AAA GAT CTT CCC CTT GGT ACC AAC AGG TGG TTT GAA TAT GTA CCA AGA ATG GCA GTT AGT GGG GTG AAA
M30              AAT CCC CAA ACC AAT GGC TTG AAA GAT CTT CCC CTT GGT ACC AAC AGG TGG TTT GAA TAT GTA CCA AGA ATG GCA GTT AGT GGG GTG AAA
M2282            AAT CCC CAA ACC AAT GGC TTG AAA GAT CTT CCC CTT GGT ACC AAC AGG TGG TTT GAA TAT GTA CCA AGA ATG GCA GTT AGT GGG GTG AAA
M2288            AAT CCC CAA ACC AAT GGC TTG AAG GAT CTT CCC CTT GGT ACC AAC AGG TGG TTT GAA TAT GTA CCA AGA ATG GCA GTT AGT GGG GTA AAA
M2300            AAT CCC CAA ACC AAT GGC TTG AAA GAT CTT CCC CTT GGT ACC AAC AGG TGG TTT GAA TAT GTA CCA AGA ATG GCA GTT AGT GGG GTG AAA
M2321            AAT CCC CAA ACC AAT GGC TTG AAG GAT CTT CCC CTT GGT ACC AAC AGG TGG TTT GAA TAT GTA CCA AGA ATG GCA GTT AGT GGG GTG AAA
M2341            AAT CCC CAA ACC AAT GGC TTG AAG GAT CTT CCC CTT GGT ACC AAC AGG TGG TTT GAA TAT GTA CCA AGA ATG GCA GTT AGT GGG GTA AAA
M6257            AAT CCC CAA ACC AAT GGC TTG AAA GAT CTT CCC CTT GGT ACC AAC AGG TGG TTT GAA TAT GTA CCA AGA ATG GCA GTT AGT GGG GTA AAA
M6280            AAT CCC CAA ACC AAT GGC TTG AAG GAT CTT CCC CTT GGT ACC AAC AAG TGG TTT GAA TAT GTA CCA AGA ATG GCA GTT AGT GGG GTA AAA
M6282            AAT CCC CAA ACC AAT GGC TTG AAG GAT CTT CCC CTT GGT ACC AAC AGG TGG TTT GAA TAT GTA CCA AGA ATG GCA GTT AGT GGG GTA AAA
M6283            AAT CCC CAA ACC AAT GGC TTG AAA GAT CTT CCC CTT GGT ACC AAC AGG TGG TTT GAA TAT GTA CCA AGA ATG GCA GTT AGT GGG GTG AAA
M6284            AAT CCC CAA ACC AAT GGC TTG AAA GAT CTT CCC CTT GGT ACC AAC AGG TGG TTT GAA TAT GTA CCA AGA ATG GCA GTT AGT GGG GTG AAA
M6285            AAT CCC CAA ACC AAT GGC TTG AAG GAT CTT CCC CTT GGT ACC AAC AGG TGG TTT GAA TAT GTA CCA AGA ATG GCA GTT AGT GGG GTG AAA
6286             AAT CCC CAA ACC AAT GGC TTG AAA GAT CTT CCC CTT GGT ACC AAC AGG TGG TTT GAA TAT GTA CCA AGA ATG GCA GTT AGT GGG GTG AAA
M6320            AAT CCC CAA ACC AAT GGC TTG AAG GAT CTT CCC CTT GGT ACC AAC AGG TGG TTT GAA TAT GTA CCA AGA ATG GCA GTT AGT GGG GTA AAA
64.0             AAT CCC CAA ACC AAT GGC TTG AAG GAT CTT CCC CTT GGT ACC AAC AGG TGG TTT GAA TAT GTA CCA AGA ATG GCA GTT AGT GGG GTA AAA
64.1             AAT CCC CAA ACC AAT GGC TTG AAG GAT CTT CCC CTT GGT ACC AAC AGG TGG TTT GAA TAT GTA CCA AGA ATG GCA GTT AGT GGG GTA AAA
199.0            AAT CCC CAA ACC AAT GGC TTG AAG GAT CTT CCC CTT GGT ACC AAC AGG TGG TTT GAA TAT GTA CCA AGA ATG GCA GTT AGT GGG GTA AAA
199.1            AAT CCC CAA ACC AAT GGC TTG AAG GAT CTT CCC CTT GGT ACC AAC AGG TGG TTT GAA TAT GTA CCA AGA ATG GCA GTT AGT GGG GTA AAA
MEGA10366        AAT CCC CAA ACC AAT GGC TTG AAA GAT CTT CCC CTT GGT ACC AAC AGG TGG TTT GAA TAT GTA CCA AGA ATG GCA GTT AGT GGG GTG AAA
MEGA10378        AAT CCC CAA ACC AAT GGC TTG AAA GAT CTT CCC CTT GGT ACC AAC AGG TGG TTT GAA TAT GTA CCA AGA ATG GCA GTT AGT GGG GTG AAA
MEGA10467        AAT CCC CAA ACC AAT GGC TTG AAG GAT CTT CCC CTT GGT ACC AAC AGG TGG TTT GAA TAT GTA CCA AGA ATG GCA GTT AGT GGG GTA AAA
MEGA10477        AAT CCC CAA ACC AAT GGC TTG AAA GAT CTT CCC CTT GGT ACC AAC AGG TGG TTT GAA TAT GTA CCA AGA ATG GCA GTT AGT GGG GTG AAA
Sea1             AAT CCC CAA ACC AAT GGC TTG AAG GAT CTT CCC CTT GGT ACC AAC AGG TGG TTT GAA TAT GTA CCA AGA ATG GCA GTT AGT GGG GTA AAA
Sea2             AAT CCC CAA ACC AAT GGC TTG AAG GAT CTT CCC CTT GGT ACC AAC AGG TGG TTT GAA TAT GTA CCA AGA ATG GCA GTT AGT GGG GTG AAA
                 *** *** *** *** *** *** *** **  *** *** *** *** *** *** *** * * *** *** *** *** *** *** *** *** *** *** *** *** **  ***
G37         514   N   P   Q   T   N   G   L   K   D   L   P   L   G   T   N   R   W   F   E   Y   V   P   R   M   A   V   S   G   V   K
                                                                              K

G37         516   N   P   Q   T   N   G   L   K   D   L   P   L   G   T   N   R   W   F   E   Y   V   P   R   M   A   V   S   G   V   K
```

```
G37       1,630  TGG GTT GGT AAT CAA CTA GTG TTA GCA GGA ACA CTA ACA ATG GGT GAT ACA GCT ACT GTA CCT AGG TTA AAG TAT GAT CAA CTA GAA AAA
M30              TGG GTT GGT AAT CAA CTA GTG TTA GCA GGA ACA CTA ACA ATG GGT GAT ACA GCT ACT GTA CCT AGG TTA AAG TAT GAT CAA CTA GAA AAA
M2282            TGG GTT GGT AAT CAA CTA GTG TTA GCA GGA ACA CTA ACA ATG GGT GAT ACA GCT ACT GTA CCT AGG TTA AAG TAT GAT CAA CTA GAA AAA
M2288            TGG GTT GGT AAT CAA CTA GTG TTA GCA GGA ACA CTA ACA ATG GGT GAT ACA GCT ACT GTA CCT AGG TTA AAG TAT GAT CAA CTA GAA AAA
M2300            TGG GTT GGT AAT CAA CTA GTG TTA GCA GGA ACA CTA ACA ATG GGT GAT ACA GCT ACT GTA CCT AGG TTA AAG TAT GAT CAA CTA GAA AAA
M2321            TGG GTT GGT AAT CAA CTA GTA TTA GCA GGA ACA CTA ACA ATG GGT GAT ACA GCA ACT GTA CCT AGG TTA AAG TAT GAC CAA CTA GAA AAA
M2341            TGG GTT GGT AAT CAA CTA GTG TTA GCA GGA ACA CTA ACA ATG GGT GAT ACA GCT ACT GTA CCT AGG TTA AAG TAT GAC CAA CTA GAA AAA
M6257            TGG GTT GGT AAT CAA CTA GTG TTA GCA GGA ACA CTA ACA ATG GGT GAT ACA GCA ACT GTA CCT AGG TTA AAG TAT GAC CAA CTA GAA AAA
M6280            TGG GTT GGT AAT CAA CTA GTG TTA GCA GGA ACA CTA ACA ATG GGT GAT ACA GCT ACT GTA CCT AGG TTA AAG TAT GAC CAA CTA GAA AAA
M6282            TGG GTT GGT AAT CAA CTA GTG TTA GCA GGA ACA CTA ACA ATG GGT GAT ACA GCT ACT GTA CCT AGG TTA AAG TAT GAC CAA CTA GAA AAA
M6283            TGG GTT GGT AAT CAA CTA GTG TTA GCA GGA ACA CTA ACA ATG GGT GAT ACA GCT ACT GTA CCT AGG TTA AAG TAT GAC CAA CTA GAA AAA
M6284            TGG GTT GGT AAT CAA CTA GTG TTA GCA GGA ACA CTA ACA ATG GGT GAT ACA GCT ACT GTA CCT AGG TTA AAG TAT GAT CAA CTA GAA AAA
M6285            TGG GTT GGT AAT CAA CTA GTA TTA GCA GGA ACA CTA ACA ATG GGT GAT ACA GCA ACT GTA CCT AGG TTA AAG TAT GAC CAA CTA GAA AAA
6286             TGG GTT GGT AAT CAA CTA GTG TTA GCA GGA ACA CTA ACA ATG GGT GAT ACA ACT ACT GTA CCT AGG TTA AAG TAT GAT CAA CTA GAA AAA
M6320            TGG GTT GGT AAT CAA CTA GTG TTA GCA GGA ACA CTA ACA ATG GGT GAT ACA GCT ACT GTA CCT AGG TTA AAG TAT GAT CAA CTA GAA AAA
64.0             TGG GTT GGT AAT CAA CTA GTG TTA GCA GGA ACA CTA ACA ATG GGT GAT ACA GCT ACT GTA CCT AGG TTA AAG TAT GAC CAA CTA GAA AAA
64.1             TGG GTT GGT AAT CAA CTA GTG TTA GCA GGA ACA CTA ACA ATG GGT GAT ACA GCT ACT GTA CCT AGG TTA AAG TAT GAC CAA CTA GAA AAA
199.0            TGG GTT GGT AAT CAA CTA GTG TTA GCA GGA ACA CTA ACA ATG GGT GAT ACA GCT ACT GTA CCT AGG TTA AAG TAT GAC CAA CTA GAA AAA
199.1            TGG GTT GGT AAT CAA CTA GTG TTA GCA GGA ACA CTA ACA ATG GGT GAT ACA GCT ACT GTA CCT AGG TTA AAG TAT GAC CAA CTA GAA AAA
MEGA10366        TGG GTT GGT AAT CAA CTA GTG TTA GCA GGA ACA CTA ACA ATG GGT GAT ACA GCT ACT GTA CCT AGG TTA AAG TAT GAT CAA CTA GAA AAA
MEGA10378        TGG GTT GGT AAT CAA CTA GTG TTA GCA GGA ACA CTA ACA ATG GGT GAT ACA GCT ACT GTA CCT AGG TTA AAG TAT GAT CAA CTA GAA AAA
MEGA10467        TGG GTT GGT AAT CAA CTA GTG TTA GCA GGA ACA CTA ACA ATG GGT GAT ACA GCT ACT GTA CCT AGG TTA AAG TAT GAT CAA CTA GAA AAA
MEGA10477        TGG GTT GGT AAT CAA CTA GTG TTA GCA GGA ACA CTA ACA ATG GGT GAT ACA GCT ACT GTA CCT AGG TTA AAG TAT GAC CAA CTA GAA AAA
Sea1             TGG GTT GGT AAT CAA CTA GTG TTA GCA GGA ACA CTA ACA ATG GGT GAT ACA GCT ACT GTA CCT AGG TTA AAG TAT GAC CAA CTA GAA AAA
Sea2             TGG GTT GGT AAT CAA CTA GTA TTA GCA GGA ACA CTA ACA ATG GGT GAT ACA GCA ACT GTA CCT AGG TTA AAG TAT GAC CAA CTA GAA AAA
                 *** *** *** *** *** *** **  *** *** *** *** *** *** *** *** *** ***  *  *** *** *** *** *** *** *** **  *** *** *** ***
G37        544    W   V   G   N   Q   L   V   L   A   G   T   L   T   M   G   D   T   A   T   V   P   R   L   K   Y   D   Q   L   E   K
                                                                                          T

Consensus  546    W   V   G   N   Q   L   V   L   A   G   T   L   T   M   G   D   T   A   T   V   P   R   L   K   Y   D   Q   L   E   K
```

**G37**	**1,720**	**CAC TTA AAC CTA GTT GCT CAA GGC CAG GGA CTA TTG AGA GAA GAC TTG CAG ATC TTC ACT CCC TAT GGG TGA GCT AAT CGT CCT GAT ATT**
M30		CAC TTA AAC CTA GTT GCT CAA GGC CAG GGA CTA TTG AGA GAA GAC TTG CAG ATC TTC ACT CCC TAT GGG TGA GCT AAT CGT CCT GAT ATT
M2282		CAC TTA AAC CTA GTT GCT CAA GGC CAG GGA CTA TTG AGA GAA GAC TTG CAG ATC TTC ACT CCC TAT GGG TGA GCT AAT CGT CCT GAT ATT
M2288		CAC TTA AAC CTA GTT GCT CAA GGC CAG GGA CTA TTG AGA GAA GAC TTG CAG ATC TTC ACT CCC TAT GGG TGA GCT AAT CGT CCT GAT ATT
M2300		CAC TTA AAC CTA GTT GCT CAA GGC CAG GGA CTA TTG AGA GAA GAC TTG CAG ATC TTC ACT CCC TAT GGG TGA GCT AAT CGT CCT GAT ATT
M2321		CAC TTA AAC CTA GTT GCT CAA GGC CAG GGA CTT TTG AGA GAA GAC TTA CAG ATC TTC ACT CCC TAT GGG TGA GCT AAC CGT CCT GAT ATT
M2341		CAC CTA AAC CTA GTT GCT CAA GGC CAG GGA CTA TTG AGA GAA GAC TTG CAG ATC TTC ACT CCC TAT GGG TGA GCT AAT CGC CCT GAT ATT
M6257		CAC CTA AAC CTA GTT GCT CAA GGC CAG GGA CTA TTG AGA GAA GAC TTG CAG ATC TTC ACT CCC TAT GGG TGA GCT AAT CGC CCT GAT ATT
M6280		CAC CTA AAC CTA GTT GCT CAA GGC CAG GGA CTT TTA AGA GAA GAT TTG CAG ATC TTC ACT CCC TAT GGG TGA GCT AAC CGT CCT GAT ATT
M6282		CAC CTA AAC CTA GTT GCT CAA GGC CAG GGA CTA TTG AGA GAA GAC TTG CAG ATC TTC ACT CCC TAT GGG TGA GCT AAT CGC CCT GAT ATT
M6283		CAC CTA AAC CTA GTT GCT CAA GGC CAG GGA CTT TTA AGA GAA GAT TTG CAG ATC TTC ACT CCC TAT GGG TGA GCT AAC CGT CCT GAT ATT
M6284		CAC TTA AAC CTA GTT GCT CAA GGC CAG GGA CTA TTG AGA GAA GAC TTG CAG ATC TTC ACT CCC TAT GGG TGA GCT AAT CGT CCT GAT ATT
M6285		CAC TTA AAC CTA GTT GCT CAA GGC CAG GGA CTT TTG AGA GAA GAC TTA CAG ATC TTC ACT CCC TAT GGG TGA GCT AAC CGT CCT GAT ATT
6286		CAC TTA AAC CTA GTT GCT CAA GGC CAG GGA CTA TTG AGA GAA GAC TTG CAG ATC TTC ACT CCC TAT GGG TGA GCT AAT CGT CCT GAT ATT
M6320		CAC TTA AAC CTA GTT GCT CAA GGC CAG GGA CTA TTG AGA GAA GAC TTG CAG ATC TTC ACT CCC TAT GGG TGA GCT AAT CGT CCT GAT ATT
64.0		CAC CTA AAC CTA GTT GCT CAA GGC CAG GGA CTT TTA AGA GAA GAT TTG CAG ATC TTC ACT CCC TAT GGG TGA GCT AAC CGT CCT GAT ATT
64.1		CAC CTA AAC CTA GTT GCT CAA GGC CAG GGA CTT TTA AGA GAA GAT TTG CAG ATC TTC ACT CCC TAT GGG TGA GCT AAC CGT CCT GAT ATT
199.0		CAC CTA AAC CTA GTT GCT CAA GGC CAG GGA CTT TTA AGA GAA GAT TTG CAG ATC TTC ACT CCC TAT GGG TGA GCT AAC CGT CCT GAT ATT
199.1		CAC CTA AAC CTA GTT GCT CAA GGC CAG GGA CTT TTA AGA GAA GAT TTG CAG ATC TTC ACT CCC TAT GGG TGA GCT AAC CGT CCT GAT ATT
MEGA10366		CAC TTA AAC CTA GTT GCT CAA GGC CAG GGA CTA TTG AGA GAA GAC TTG CAG ATC TTC ACT CCC TAT GGG TGA GCT AAT CGT CCT GAT ATT
MEGA10378		CAC TTA AAC CTA GTT GCT CAA GGC CAG GGA CTA TTG AGA GAA GAC TTG CAG ATC TTC ACT CCC TAT GGG TGA GCT AAT CGT CCT GAT ATT
MEGA10467		CAC TTA AAC CTA GTT GCT CAA GGT CAG GGA CTA TTG AGA GAA GAC TTG CAG ATC TTC ACT CCC TAT GGG TGA GCT AAT CGT CCT GAT ATT
MEGA10477		CAC CTA AAC CTA GTT GCT CAA GGC CAG GGA CTT TTA AGA GAA GAT TTG CAG ATC TTC ACT CCC TAT GGG TGA GCT AAC CGT CCT GAT ATT
Sea1		CAC CTA AAC CTA GTT GCT CAA GGC CAG GGA CTT TTA AGA GAA GAT TTG CAG ATC TTC ACT CCC TAT GGG TGA GCT AAC CGT CCT GAT ATT
Sea2		CAC TTA AAC CTA GTT GCT CAA GGC CAG GGA CTT TTG AGA GAA GAC TTA CAG ATC TTC ACT CCC TAT GGG TGA GCT AAC CGT CCT GAT ATT
		*** ** *** *** *** *** *** ** *** *** ** ** *** *** ** ** *** *** *** *** *** *** *** *** *** ** ** *** *** ***
**G37**	**574**	**H L N L V A Q G Q G L L R E D L Q I F T P Y G W A N R P D I**
**Consensus**	**576**	**H L N L V A Q G Q G L L R E D L Q I F T P Y G W A N R P D I**

**G37**	**1,810**	**CCT**	**GTA**	**GGA**	**GCA**	**TGA**	**CTC**	**CAA**	**GAT**	**GAA**	**ATG**	**GGC**	**AGT**	**---**	**AAA**	**TTT**	**GGT**	**CCC**	**CAT**	**TAC**	**TTC**	**TTA**	**AAT**	**AAC**	**CCT**	**GAT**	**ATC**	**CAG**	**GAC**	**AAT**	**GTT**
M30		CCT	GTA	GGA	GCA	TGA	CTC	CAA	GAT	GAA	ATG	GGC	AGT	---	AAA	TTT	GGT	CCC	CAT	TAC	TTC	TTA	AAT	AAC	CCT	GAT	ATC	CAG	GAC	AAT	GTT
M2282		CCT	GTA	GGA	GCA	TGA	CTC	CAA	GAT	GAA	ATG	GGC	AGT	---	AAA	TTT	GGT	CCC	CAT	TAC	TTC	TTA	AAT	AAC	CCT	GAT	ATC	CAG	GAC	AAT	GTT
M2288		CCT	GCA	GGA	GCA	TGA	CTC	CAA	GAT	GAA	ATG	GGC	AGT	---	AAA	TTT	GGT	CCC	CAT	TAC	TTT	TTG	AAT	AAC	CCT	GAT	ATC	CAG	GAC	AAT	GTT
M2300		CCT	GTA	GGA	GCA	TGA	CTC	CAA	GAT	GAA	ATG	GGC	AGT	---	AAA	TTT	GGT	CCC	CAT	TAC	TTC	TTA	AAT	AAC	CCT	GAT	ATC	CAG	GAC	AAT	GTT
M2321		CCT	GCA	GGA	GCA	TGA	CTC	CAA	GAT	GAA	GCG	GGC	AGC	---	AAG	TTT	GGT	CCC	CAT	TAC	TTC	TTA	AAT	AAC	CCT	GAT	ATC	CAG	GAC	AAT	GTT
M2341		CCT	GTA	GGA	GCA	TGA	CTC	CAA	GAT	GAA	ATG	GGC	AGT	---	AAA	TTT	GGT	CCC	CAT	TAC	TTC	TTA	AAT	AAC	CCT	GAT	ATC	CAG	GAC	AAT	GTT
M6257		CCT	GTA	GGA	GCA	TGA	CTC	CAA	GAT	GAA	ATG	GGC	AGT	---	AAA	TTT	GGT	CCC	CAT	TAC	TTC	TTA	AAT	AAC	CCT	GAT	ATC	CAG	GAC	AAT	GTT
M6280		CCT	GTA	GGA	GCA	TGA	CTC	CAA	GAT	GAA	ATG	GGC	AGT	---	AAA	TTT	GGT	CCC	CAT	TAC	TTC	TTA	AAT	AAC	CCT	GAT	ATC	CAG	GAC	GAT	GTT
M6282		CCT	GTA	GGA	GCA	TGA	CTC	CAA	GAT	GAA	ATG	GGC	AGT	---	AAA	TTT	GGT	CCC	CAT	TAC	TTC	TTA	AAT	AAC	CCT	GAT	ATC	CAG	GAC	AAT	GTT
M6283		CCT	GCA	GGA	GCA	TGA	CTC	CAA	GAT	GAA	ATG	GGC	AGT	---	AAA	TTT	GGT	CCC	CAT	TAC	TTT	TTG	AAT	AAC	CCT	GAT	ATC	CAG	GAC	AAT	GTT
M6284		CCT	GTA	GGA	GCA	TGA	CTC	CAA	AAT	GAA	ATG	GGC	AGT	---	AAA	TTT	GGT	CCC	CAT	TAC	TTC	TTA	AAT	AAC	CCT	GAT	ATC	CAG	GAC	AAT	GTT
M6285		CCT	GCA	GGA	GCA	TGA	CTC	CAA	AAT	GAA	GCG	GGC	AGC	---	AAG	TTT	GGT	CCC	CAT	TAC	TTC	TTA	AAT	AAC	CCT	GAT	ATC	CAG	GAC	AAT	GTT
6286		CCT	GCA	GGA	GCA	TGA	CTC	CAA	GAT	GAA	ATG	GGC	AGT	AAT	AAA	TTT	GGT	CCC	CAT	TAC	TTT	TTG	AAT	AAC	CCT	GAT	ATC	CAG	GAC	AAT	GTT
M6320		CCT	GTA	GGA	GCA	TGA	CTC	CAA	GAT	GAA	ATG	GGC	AGT	---	AAA	TTT	GGT	CCC	CAT	TAC	TTC	TTA	AAT	AAC	CCT	GAT	ATC	CAG	GAC	AAT	GTT
64.0		CCT	GTA	GGA	GCA	TGA	CTC	CAA	GAT	GAA	ATG	GGC	AGT	---	AAA	TTT	GGT	CCC	CAT	TAC	TTC	TTA	AAT	AAC	CCT	GAT	ATC	CAG	GAC	GAT	GTT
64.1		CCT	GTA	GGA	GCA	TGA	CTC	CAA	GAT	GAA	ATG	GGC	AGT	---	AAA	TTT	GGT	CCC	CAT	TAC	TTC	TTA	AAT	AAC	CCT	GAT	ATC	CAG	GAC	GAT	GTT
199.0		CCT	GTA	GGA	GCA	TGA	CTC	CAA	GAT	GAA	ATG	GGC	AGT	---	AAA	TTT	GGT	CCC	CAT	TAC	TTC	TTA	AAT	AAC	CCT	GAT	ATC	CAG	GAC	GAT	GTT
199.1		CCT	GTA	GGA	GCA	TGA	CTC	CAA	GAT	GAA	ATG	GGC	AGT	---	AAA	TTT	GGT	CCC	CAT	TAC	TTC	TTA	AAT	AAC	CCT	GAT	ATC	CAG	GAC	GAT	GTT
MEGA10366		CCT	GCA	GGA	GCA	TGA	CTC	CAA	GAT	GAA	ATG	GGC	AGT	---	AAA	TTT	GGT	CCC	CAT	TAC	TTT	TTG	AAT	AAC	CCT	GAT	ATC	CAG	GAC	AAT	GTT
MEGA10378		CCT	GTA	GGA	GCA	TGA	CTC	CAA	GAT	GAA	ATG	GGC	AGT	---	AAA	TTT	GGT	CCC	CAT	TAC	TTC	TTA	AAT	AAC	CCT	GAT	ATC	CAG	GAC	AAT	GTT
MEGA10467		CCT	GCA	GGA	GCA	TGA	CTC	CAA	GAT	GAA	ATG	GGC	AGT	---	AAA	TTT	GGT	CCC	CAT	TAC	TTT	TTG	AAT	AAC	CCT	GAT	ATC	CAG	GAC	AAT	GTT
MEGA10477		CCT	GCA	GGA	GCA	TGA	CTC	CAA	GAT	GAA	ATG	GGC	AGT	---	AAA	TTT	GGT	CCC	CAT	TAC	TTT	TTG	AAT	AAC	CCT	GAT	ATC	CAG	GAC	AAT	GTT
Sea1		CCT	GTA	GGA	GCA	TGA	CTC	CAA	GAT	GAA	ATG	GGC	AGT	---	AAA	TTT	GGT	CCC	CAT	TAC	TTC	TTA	AAT	AAC	CCT	GAT	ATC	CAG	GAC	GAT	GTT
Sea2		CCT	GCA	GGA	GCA	TGA	CTC	CAA	GAT	GAA	GAG	GGC	AGC	---	AAG	TTT	GGT	CCC	CAT	TAC	TTC	TTA	AAT	AAC	CCT	GAT	ATC	CAG	GAC	AAT	GTT
		***	* *	***	***	***	***	***	**	***	*	***	**		**	***	***	***	***	***	**	**	***	***	***	***	***	***	***	**	***
**G37**	**604**	**P**	**V**	**G**	**A**	**W**	**L**	**Q**	**D**	**E**	**M**	**G**	**S**	**–**	**K**	**F**	**G**	**P**	**H**	**Y**	**F**	**L**	**N**	**N**	**P**	**D**	**I**	**Q**	**D**	**N**	**V**
			**A**						**N**		**A**			**N**																**D**	
											**E**																				
**Consensus**	**606**	**P**	**V**	**G**	**A**	**W**	**L**	**Q**	**D**	**E**	**M**	**G**	**S**	**–**	**K**	**F**	**G**	**P**	**H**	**Y**	**F**	**L**	**N**	**N**	**P**	**D**	**I**	**Q**	**D**	**N**	**V**

**G37**	**1,897**	**AAT**	**AAT**	**GAT**	**ACG**	**GTT**	**GAA**	**GCA**	**TTA**	**ATC**	**AGT**	**AGT**	**TAC**	**AAA**	**AAC**	**ACT**	**GAT**	**AAG**	**TTA**	**AAA**	**CAC**	**GTT**	**TAT**	**CCT**	**TAT**	**CGA**	**TAC**	**AGT**	**GGT**	**TTG**	**TAT**
M30		AAT	AAT	GAT	ACG	GTT	GAA	GCA	TTA	ATC	AGT	AGT	TAC	AAA	AAC	ACT	GAT	AAG	TTA	AAA	CAC	GTT	TAT	CCT	TAT	CGA	TAC	AGT	GGT	TTG	TAT
M2282		AAT	AAT	GAT	ACG	GTT	GAA	GCA	TTA	ATC	AGT	AGT	TAC	AAA	AAC	ACT	GAT	AAG	TTA	AAA	CAC	GTT	TAT	CCT	TAT	CGA	TAC	AGT	GGT	TTG	TAT
M2288		AAT	AAT	GAT	ACG	GTT	GAA	GCA	TTA	ATC	AAT	ACC	TAT	ACA	AAC	ACT	GAT	AAG	TTA	AAA	CAC	GTT	TAT	CCT	TAT	AGA	TAC	AGT	GGT	TTG	TAT
M2300		AAT	AAT	GAT	ACG	GTT	GAA	GCA	TTA	ATC	AGT	AGT	TAC	AAA	AAC	ACT	GAT	AAG	TTA	AAA	CAC	GTT	TAT	CCT	TAT	CGA	TAC	AGT	GGT	TTG	TAT
M2321		AAT	AAT	GAT	ACG	GTT	GAA	GCA	TTA	ATC	AGT	ACC	TAT	ACA	AAC	ACT	GAT	GAG	TTA	AAA	CAC	GTT	TAT	CCT	TAT	AGA	TAC	AGT	GGT	TTG	TAT
M2341		AAT	AAT	GAT	ACG	GTT	GAA	GCA	TTA	ATC	AAT	ACC	TAT	ACA	AAC	ACT	GAT	AAG	TTA	AAA	CAC	GTT	TAT	CCT	TAT	CGA	TAC	AGT	GGT	TTG	TAT
M6257		AAT	AAT	GAT	ACG	GTT	GAA	GCA	TTA	ATC	AAT	ACC	TAT	ACA	AAC	ACT	GAT	AAG	TTA	AAA	CAC	GTT	TAT	CCT	TAT	CGA	TAC	AGT	GGT	TTG	TAT
M6280		AGG	GAT	GAT	ACA	GTT	GAA	GCA	TTA	ATC	AGT	AGT	TAC	AAA	AAC	ACT	GAT	AAG	TTA	AAA	CAC	GTT	TAT	CCT	TAT	CGA	TAC	AGT	GGT	TTG	TAT
M6282		AAT	AAT	TAT	ACG	GTT	GAA	GTA	TTA	ATC	AAT	ACC	TAT	ACA	AAC	ACT	GAT	AAG	TTA	AAA	CAC	GTT	TAT	CCT	TAT	CGA	TAC	AGT	GGT	TTG	TAT
M6283		AAT	AAT	GAT	ACG	GTT	GAA	GCA	TTA	ATC	AAT	ACC	TAT	ACA	AAC	ACT	GAT	AAG	TTA	AAA	CAC	GTT	TAT	CCT	TAT	AGA	TAC	AGT	GGT	TTG	TAT
M6284		AAT	AAT	GAT	ACG	GTT	GAA	GCA	TTA	ATC	AGT	AGT	TAC	AAA	AAC	ACT	GAT	AAG	TTA	AAA	CAC	GTT	TAT	CCT	TAT	CGA	TAC	AGT	GGT	TTG	TAT
M6285		AAT	AAT	GAT	ACG	GTT	GAA	GCA	TTA	ATC	AGT	ACC	TAT	ACA	AAC	ACT	GAT	GAG	TTA	AAA	CAC	GTT	TAT	CCT	TAT	AGA	TAC	AGT	GGT	TTG	TAT
6286		AAT	AAT	GAT	ACG	GTT	GAA	GCA	TTA	ATC	AAT	ACC	TAT	ACA	AAC	ACT	GAT	AAG	TTA	AAA	CAC	GTT	TAT	CCT	TAT	AGA	TAC	AGT	GGT	TTG	TAT
M6320		AAT	AAT	GAT	ACG	GTT	GAA	GCA	TTA	ATC	AGT	AGT	TAC	AAA	AAC	ACT	GAT	AAG	TTA	AAA	CAC	GTT	TAT	CCT	TAT	CGA	TAC	AGT	GGT	TTG	TAT
64.0		AGG	GAT	GAT	ACA	GTT	GAA	GCA	TTA	ATC	AGT	AGT	TAC	AAA	AAC	ACT	GAT	AAG	TTA	AAA	CAC	GTT	TAT	CCT	TAT	CGA	TAC	AGT	GGT	TTG	TAT
64.1		AGG	GAT	GAT	ACA	GTT	GAA	GCA	TTA	ATC	AGT	AGT	TAC	AAA	AAC	ACT	GAT	AAG	TTA	AAA	CAC	GTT	TAT	CCT	TAT	CGA	TAC	AGT	GGT	TTG	TAT
199.0		AGG	GAT	GAT	ACA	GTT	GAA	GCA	TTA	ATC	AGT	AGT	TAC	AAA	AAC	ACT	GAT	AAG	TTA	AAA	CAC	GTT	TAT	CCT	TAT	CGA	TAC	AGT	GGT	TTG	TAT
199.1		AGG	GAT	GAT	ACA	GTT	GAA	GCA	TTA	ATC	AGT	AGT	TAC	AAA	AAC	ACT	GAT	AAG	TTA	AAA	CAC	GTT	TAT	CCT	TAT	CGA	TAC	AGT	GGT	TTG	TAT
MEGA10366		AAT	AAT	GAT	ACG	GTT	GAA	GCA	TTA	ATC	AAT	ACC	TAT	ACA	AAC	ACT	GAT	AAG	TTA	AAA	CAC	GTT	TAT	CCT	TAT	AGA	TAC	AGT	GGT	TTG	TAT
MEGA10378		AAT	AAT	GAT	ACG	GTT	GAA	GCA	TTA	ATC	AGT	AGT	TAC	AAA	AAC	ACT	GAT	AAG	TTA	AAA	CAC	GTT	TAT	CCT	TAT	CGA	TAC	AGT	GGT	TTG	TAT
MEGA10467		AAT	AAT	GAT	ACG	GTT	GAA	GCA	TTA	ATC	AAT	ACC	TAT	ACA	AAC	ACT	GAT	AAG	TTA	AAA	CAC	GTT	TAT	CCT	TAT	AGA	TAC	AGT	GGT	TTG	TAT
MEGA10477		AAT	AAT	GAT	ACG	GTT	GAA	GCA	TTA	ATC	AAT	ACC	TAT	ACA	AAC	ACT	GAT	AAG	TTA	AAA	CAC	GTT	TAT	CCT	TAT	AGA	TAC	AGT	GGT	TTG	TAT
Sea1		AGG	GAT	GAT	ACA	GTT	GAA	GCA	TTA	ATC	AGT	AGT	TAC	AAA	AAC	ACT	GAT	AAG	TTA	AAA	CAC	GTT	TAT	CCT	TAT	CGA	TAC	AGT	GGT	TTG	TAT
Sea2		GAT	AAT	GAT	ACG	GTT	GAA	GCA	TTA	ATC	AGT	ACC	TAT	ACA	AAC	ACT	GAT	GAG	TTA	AAA	CAC	GTT	TAT	CCT	TAT	AGA	TAC	AGT	GGT	TTG	TAT
			**	**	**	***	***	* *	***	***	* *	*	**	* *	***	***	***	**	***	***	***	***	***	***	***	**	***	***	***	***	***
**G37**	**633**	**N**	**N**	**D**	**T**	**V**	**E**	**A**	**L**	**I**	**S**	**S**	**Y**	**K**	**N**	**T**	**D**	**K**	**L**	**K**	**H**	**V**	**Y**	**P**	**Y**	**R**	**Y**	**S**	**G**	**L**	**Y**
		**R**	**D**	**Y**				**V**			**N**	**T**		**T**				**E**													
		**D**																													

**G37**	**635**	**N**	**N**	**D**	**T**	**V**	**E**	**A**	**L**	**I**	**S**	**S**	**Y**	**K**	**N**	**T**	**D**	**K**	**L**	**K**	**H**	**V**	**Y**	**P**	**Y**	**R**	**Y**	**S**	**G**	**L**	**Y**

**G37**	**1,987**	**GCT**	**TGA**	**CAG**	**TTA**	**TTT**	**AAC**	**TGG**	**TCT**	**AAC**	**AAA**	**CTA**	**ACC**	**AAC**	**ACT**	**CCC**	**CTA**	**TCA**	**GCT**	**AAC**	**TTT**	**GTT**	**AAT**	**GAA**	**AAC**	**AGT**	**TAT**	**GCA**	**CCA**	**AAC**	**AGT**
M30		GCT	TGA	CAG	TTA	TTT	AAC	TGG	TCT	AAC	AAA	CTA	ACC	AAC	ACT	CCC	CTA	TCA	GCT	AAC	TTT	GTT	AAT	GAA	AAC	AGT	TAT	GCA	CCA	AAC	AGT
M2282		GCT	TGA	CAG	TTA	TTT	AAC	TGG	TCT	AAC	AAA	CTA	ACC	AAC	ACT	CCC	CTA	TCA	GCT	AAC	TTT	GTT	AAT	GAA	AAC	AGT	TAT	GCA	CCA	AAC	AGT
M2288		GCT	TGA	CAG	TTA	TTT	AAC	TGG	TCT	AAC	AAA	CTA	ACC	AAT	ACT	TCC	CTT	TCC	GCT	AAC	TTT	GTT	AAT	GAA	AAC	AGT	TAT	GCA	CCA	AAC	AGT
M2300		GCT	TGA	CAG	TTA	TTT	AAC	TGG	TCT	AAC	AAA	CTA	ACC	AAC	ACT	CCC	CTA	TCA	GCT	AAC	TTT	GTT	AAT	GAA	AAC	AGT	TAT	GCA	CCA	AAC	AGT
M2321		GCT	TGA	CAG	TTA	TTT	AAC	TGG	TCT	AAC	AAA	CTA	ACC	AAT	ACT	CCC	CTT	TCC	GCT	AAC	TTT	GTT	AAT	GAA	AAC	AGT	TAT	GCA	CCA	AAC	AGT
M2341		GCT	TGA	CAG	TTA	TTT	AAC	TGG	TCT	AAC	AAA	CTA	ACC	AAT	ACT	TCC	CTT	TCC	GCT	AAC	TTT	GTT	AAT	GAA	AAC	AGT	TAT	GCA	CCA	AAC	AGT
M6257		GCT	TGA	CAG	TTA	TTT	AAC	TGG	TCT	AAC	AAA	CTA	ACC	AAT	ACT	TCC	CTT	TCC	GCT	AAC	TTT	GTT	AAT	GAA	AAC	AGT	TAT	GCA	CCA	AAC	AGT
M6280		GCT	TGA	CAG	TTA	TTT	AAC	TGG	TCT	AAC	AAA	CTA	ACC	AAC	ACT	CCC	CTA	TCA	GCT	AAC	TTT	GTT	AAT	GAA	AAC	AGT	TAT	GCA	CCA	AAC	AGT
M6282		GCT	TGA	CAG	TTA	TTT	AAC	TGG	TCT	AAC	AAA	CTA	ACC	AAT	ACT	TCC	CTT	TCC	GCT	AAC	TTT	GTT	AAT	GAA	AAC	AGT	TAT	GCA	CCA	AAC	AGT
M6283		GCT	TGA	CAG	TTA	TTT	AAC	TGG	TCT	AAC	AAA	CTA	ACC	AAT	ACT	TCC	CTT	TCC	GCT	AAC	TTT	GTT	AAT	GAA	AAC	AGT	TAT	GCA	CCA	AAC	AGT
M6284		GCT	TGA	CAG	TTA	TTT	AAC	TGG	TCT	AAC	AAA	CTA	ACC	AAC	ACT	CCC	CTA	TCA	GCT	AAC	TTT	GTT	AAT	GAA	AAC	AGT	TAT	GCA	CCA	AAC	AGT
M6285		GCT	TGA	CAG	TTA	TTT	AAC	TGG	TCT	AAC	AAA	CTA	ACC	AAT	ACT	CCC	CTT	TCC	GCT	AAC	TTT	GTT	AAT	GAA	AAC	AGT	TAT	GCA	CCA	AAC	AGT
6286		GCT	TGA	CAG	TTA	TTT	AAC	TGG	TCT	AAC	AAA	CTA	ACC	AAT	ACT	TCC	CTT	TCC	GCT	AAC	TTT	GTT	AAT	GAA	AAC	AGT	TAT	GCA	CCA	AAC	AGT
M6320		GCT	TGA	CAG	TTA	TTT	AAC	TGG	TCT	AAC	AAA	CTA	ACC	AAC	ACT	CCC	CTA	TCA	GCT	AAC	TTT	GTT	AAT	GAA	AAC	AGT	TAT	GCA	CCA	AAC	AGT
64.0		GCT	TGA	CAG	TTA	TTT	AAC	TGG	TCT	AAC	AAA	CTA	ACC	AAC	ACT	CCC	CTA	TCA	GCT	AAC	TTT	GTT	AAT	GAA	AAC	AGT	TAT	GCA	CCA	AAC	AGT
64.1		GCT	TGA	CAG	TTA	TTT	AAC	TGG	TCT	AAC	AAA	CTA	ACC	AAC	ACT	CCC	CTA	TCA	GCT	AAC	TTT	GTT	AAT	GAA	AAC	AGT	TAT	GCA	CCA	AAC	AGT
199.0		GCT	TGA	CAG	TTA	TTT	AAC	TGG	TCT	AAC	AAA	CTA	ACC	AAC	ACT	CCC	CTA	TCA	GCT	AAC	TTT	GTT	AAT	GAA	AAC	AGT	TAT	GCA	CCA	AAC	AGT
199.1		GCT	TGA	CAG	TTA	TTT	AAC	TGG	TCT	AAC	AAA	CTA	ACC	AAC	ACT	CCC	CTA	TCA	GCT	AAC	TTT	GTT	AAT	GAA	AAC	AGT	TAT	GCA	CCA	AAC	AGT
MEGA10366		GCT	TGA	CAG	TTA	TTT	AAC	TGG	TCT	AAC	AAA	CTA	ACC	AAT	ACT	TCC	CTT	TCC	GCT	AAC	TTT	GTT	AAT	GAA	AAC	AGT	TAT	GCA	CCA	AAC	AGT
MEGA10378		GCT	TGA	CAG	TTA	TTT	AAC	TGG	TCT	AAC	AAA	CTA	ACC	AAC	ACT	CCC	CTA	TCA	GCT	AAC	TTT	GTT	AAT	GAA	AAC	AGT	TAT	GCA	CCA	AAC	AGT
MEGA10467		GCT	TGA	CAG	TTA	TTT	AAC	TGG	TCT	AAC	AAA	CTA	ACC	AAT	ACT	TCC	CTT	TCC	GCT	AAC	TTT	GTT	AAT	GAA	AAC	AGT	TAT	GCA	CCA	AAC	AGT
MEGA10477		GCT	TGA	CAG	TTA	TTT	AAC	TGG	TCT	AAC	AAA	CTA	ACC	AAT	ACT	TCC	CTT	TCC	GCT	AAC	TTT	GTT	AAT	GAA	AAC	AGT	TAT	GCA	CCA	AAC	AGT
Sea1		GCT	TGA	CAG	TTA	TTT	AAC	TGG	TCT	AAC	AAA	CTA	ACC	AAC	ACT	CCC	CTA	TCA	GCT	AAC	TTT	GTT	AAT	GAA	AAC	AGT	TAT	GCA	CCA	AAC	AGT
Sea2		GCT	TGA	CAG	TTA	TTT	AAC	TGG	TCT	AAC	AAA	CTA	ACC	AAT	ACT	CCC	CTT	TCC	GCT	AAC	TTT	GTT	AAT	GAA	AAC	AGT	TAT	GCA	CCA	AAC	AGT
		***	***	***	***	***	***	***	***	***	***	***	***	**	***	**	**	**	***	***	***	***	***	***	***	***	***	***	***	***	***
**G37**	**663**	**A**	**W**	**Q**	**L**	**F**	**N**	**W**	**S**	**N**	**K**	**L**	**T**	**N**	**T**	**P**	**L**	**S**	**A**	**N**	**F**	**V**	**N**	**E**	**N**	**S**	**Y**	**A**	**P**	**N**	**S**
																**S**															
**Consensus**	**665**	**A**	**W**	**Q**	**L**	**F**	**N**	**W**	**S**	**N**	**K**	**L**	**T**	**N**	**T**	**P**	**L**	**S**	**A**	**N**	**F**	**V**	**N**	**E**	**N**	**S**	**Y**	**A**	**P**	**N**	**S**

```
G37       2,077   TTG TTT GCT GCT ATC TTA AAT GAA GAT CTG TTA ACA GGG CTA AGT GAT AAG ATT TTC TAT GGT AAG GAG AAT GAG TTT GCT GAA AAT GAA
M30               TTG TTT GCT GCT ATC TTA AAT GAA GAT CTG TTA ACA GGG CTA AGT GAT AAG ATT GTC TAT GGT AAG GAG AAT AAG TTT GCT GAA AAT GAA
M2282             TTG TTT GCT GCT ATC TTA AAT GAA GAT CTG TTA ACA GGG CTA AGT GAT AAG ATT GTC TAT GGT AAG GAG AAT GAG TTT GCT GAA AAT GAA
M2288             TTG TTT GCT GCT ATC TTA AAT GAA GAT CTG TTA ACA GGG CTA AGT GAT AAG ATT GGC TAT GGT AAG GAG AAT GAG TTT GCT GAT AAT GAA
M2300             TTG TTT GCT GCT ATC TTA AAT GAA GAT CTG TTA ACA GGG CTA AGT GAT AAG ATT GTC TAT GGT AAG GAG AAT GAG TTT GCT GAA AAT GAA
M2321             TTG TTT GCT GCT ATC TTA AAT GAA GAT CTG TTA ACA GGG CTA AGT GAT AAG ATT GGC TAT GGT AAG GAG AAT GAG TTT GCT GAT AAT GAA
M2341             TTG TTT GCT GCT ATC TTA AAT GAA GAT CTG TTA ACA GGG CTA AGT GAT AAG ATA ATG TAT GGA AGT GAA AAT GAA TAT GCA GCA AAT GAA
M6257             TTG TTT GCT GCT ATC TTA AAT GAA GAT CTG TTA ACA GGG CTA AGT GAT AAG ATA ATG TAT GGA AGT GAA AAT GAA TAT GCA GCA AAT GAA
M6280             TTG TTT GCT GCT ATC TTA AAT GAA GAT CTG TTA ACA GGG CTA AGT GAT AAG ATT GTC TAT GGT AAG GAG AAT GAG TTT GCT GAA AAT GAA
M6282             TTG TTT GCT GCT ATC TTA AAT GAA GAT CTG TTA ACA GGG CTA AGT GAT AAG ATT GTC TAT GGT AAG GAG AAT GAG TTT GCT GAA AAT GAA
M6283             TTG TTT GCT GCT ATC TTA AAT GAA GAT CTG TTA ACA GGG CTA AGT GAT AAG ATT GGC TAT GGT AAG GAG AAT GAG TTT GCT GAT AAT GAA
M6284             TTG TTT GCT GCT ATC TTA AAT GAA GAT CTG TTA ACA GGG CTA AGT GAT AAG ATT GTC TAT GGT AAG GAG AAT GAG TTT GCT GAA AAT GAA
M6285             TTG TTT GCT GCT ATC TTA AAT GAA GAT CTG TTA ACA GGG CTA AGT GAT AAG ATT GGC TAT GGT AAG GAG AAT GAG TTT GCT GAT AAT GAA
6286              TTG TTT GCT GCT ATC TTA AAT GAA GAT CTG TTA ACA GGG CTA AGT GAT AAG ATT GAC TAT GGT AAG GAG AAT GAG TTT GCT GAT AAT GAA
M6320             TTG TTT GCT GCT ATC TTA AAT GAA GAT CTG TTA ACA GGG CTA AGT GAT AAG ATT GTC TAT GGT AAG GAG AAT GAG TTT GCT GAA AAT GAA
64.0              TTG TTT GCT GCT ATC TTA AAT GAA GAT CTG TTA ACA GGG CTA AGT GAT AAG ATT GTC TAT GAT AAG GAG AAT GAG TTT GCT GAA AAT GAA
64.1              TTG TTT GCT GCT ATC TTA AAT GAA GAT CTG TTA ACA GGG CTA AGT GAT AAG ATT GTC TAT GAT AAG GAG AAT GAG TTT GCT GAA AAT GAA
199.0             TTG TTT GCT GCT ATC TTA AAT GAA GAT CTG TTA ACA GGG CTA AGT GAT AAG ATT GTC TAT GGT AAG GAG AAT GAG TTT GCT GAA AAT GAA
199.1             TTG TTT GCT GCT ATC TTA AAT GAA GAT CTG TTA ACA GGG CTA AGT GAT AAG ATT GTC TAT GGT AAG GAG AAT GAG TTT GCT GAA AAT GAA
MEGA10366         TTG TTT GCT GCT ATC TTA AAT GAA GAT CTG TTA ACA GGG CTA AGT GAT AAG ATT GGC TAT GGT AAG GAG AAT GAG TTT GCT GAT AAT GAA
MEGA10378         TTG TTT GCT GCT ATC TTA AAT GAA GAT CTG TTA ACA GGG CTA AGT GAT AAG ATT GTC TAT GGT AAG GAG AAT GAG TTT GCT AAA AAT GAA
MEGA10467         TTG TTT GCT GCT ATC TTA AAT GAA GAT CTG TTA ACA GGG CTA AGT GAT AAG ATT GAC TAT GGT AAG GAG AAT GAG TTT GCT GAT AAT GAA
MEGA10477         TTG TTT GCT GCT ATC TTA AAT GAA GAT CTG TTA ACA GGG CTA AGT GAT AAG ATT GGC TAT GGT AAG GAG AAT GAG TTT GCT GAT AAT GAA
Sea1              TTG TTT GCT GCT ATC TTA AAT GAA GAT CTG TTA ACA GGG CTA AGT GAT AAG ATT TTC TAT GAT AAG GAG AAT GAG TTT GCT GAA AAT GAA
Sea2              TTG TTT GCT GCT ATC TTA AAT GAA GAT CTG TTA ACA GGG CTA AGT GAT AAG ATT GAC TAT GGT AAG GAG AAT GAG TTT GCT GAT AAT GAA
                  *** *** *** *** *** *** *** *** *** *** *** *** *** *** *** *** *** **      *** *   *   **  ***  *  * * **      *** ***
G37         693    L   F   A   A   I   L   N   E   D   L   L   T   G   L   S   D   K   I   F   Y   G   K   E   N   E   F   A   E   N   E
                                                                                           V       D   S           K   Y       D
                                                                                           G                                   A
                                                                                           M                                   K
                                                                                           D

Consensus   695    L   F   A   A   I   L   N   E   D   L   L   T   G   L   S   D   K   I   V   Y   G   K   E   N   E   F   A   E   N   E
```

```
G37        2,167  GCA GAT AGG TTT AAC CAA CTT TTA AGT TTA AAT CCT AAT CCT AAC ACT AAC TGA GCT AGG TAT TTA AAC GTA GTA CAA CGT TTT ACT ACC
M30               GCA GAT AGG TTT AAC CAA CTT TTA AGT TTA AAT CCT AGT CCT AAC ACT AAC TGA GCT AGG TAT TTA AAC GTA GTA CAA CGT TTT ACT ACC
M2282             GCA GAT AGG TTT AAC CAA CTT TTA AGT TTA AAT CCT AGT CCT AAC ACT AAC TGA GCT AGG TAT TTA AAC GTA GTA CAA CGT TTT ACT ACC
M2288             GCA GAT AGG TTT AAC CAA CTT TTA AGT TTA AAT CCT AGT GAT AAC ACC AAC TGA GCT AGG TAT TTA AAC GTA GTA CAA CGT TTT ACT ACC
M2300             GCA GAT AGG TTT AAC CAA CTT TTA AGT TTA AAT CCT AGT CCT AAC ACT AAC TGA GCT AGG TAT TTA AAC GTA GTA CAA CGT TTT ACT ACC
M2321             GCA GAT AGG TTT AAC CAA CTT TTA AGT TTA AAT CCT AGT TCT AAC ACT AAC TGA GCT AGG TAT TTA AAC GTA GTA CAA CGT TTT ACT ACC
M2341             GCA GAT AGG TTT AAC CAA CTG TTA AGT TTA AAT CCT AGT TCT AAC ACT AAC TGA GCT AGG TAT TTA AAC GTA GTA CAA CGT TTT ACT ACC
M6257             GCA GAT AGG TTT AAC CAA CTG TTA AGT TTA AAT CCT AGT TCT AAC ACT AAC TGA GCT AGC TAT TTA AAC GTA GTA CAA CGT TTT ACT ACC
M6280             GCA GAT AGG TTT AAC CAA CTT TTA AGT TTA AAT CCT AGT CCT AAC ACT AAC TGA GCT AGG TAT TTA AAC GTA GTA CAA CGT TTT ACT ACC
M6282             GCA GAT AGG TTT AAC CAA CTT TTA AGT TTA AAT CCT AGT CCT AAC ACT AAC TGA GCT AGG TAT TTA AAC GTA GTA CAA CGT TTT ACT ACC
M6283             GCA GAT AGG TTT AAC CAA CTT TTA AGT TTA AAT CCT AGT GCT AAC ACC AAC TGA GCT AGG TAT TTA AAC GTA GTA CAA CGT TTT ACT ACC
M6284             GCA GAT AGG TTT AAC CAA CTT TTA AGT TTA AAT CCT AGT TCT AAC ACT AAC TGA GCT AGG TAT TTA AAC GTA GTA CAA CGT TTT ACT ACC
M6285             GCA GAT AGG TTT AAC CAA CTT TTA AGT TTA AAT CCT AGT TCT AAC ACT AAC TGA GCT AGG TAT TTA AAC GTA GTA CAA CGT TTT ACT ACC
6286              GCA GAT AGG TTT AAC CAA CTT TTA AGT TTA AAT CCT AGT GCT AAC ACC AAC TGA GCT AGG TAT TTA AAC GTA GTA CAA CGT TTT ACT ACC
M6320             GCA GAT AGG TTT AAC CAA CTT TTA AGT TTA AAT CCT AAT CCT AAC ACT AAC TGA GCT AGG TAT TTA AAC GTA GTA CAA CGT TTT ACT ACC
64.0              GCA GAT AGG TTT AAT CAA CTT TTA AGT TTA AGT CCT AGT CCT AAC ACT AAC TGA GCT AGG TAT TTA AAC GTA GTA CAA CGT TTT ACT ACC
64.1              GCA GAT AGG TTT AAT CAA CTT TTA AGT TTA AAT CCT AGT CCT AAC ACT AAC TGA GCT AGG TAT TTA AAC GTA GTA CAA CGT TTT ACT ACC
199.0             GCA GAT AGG TTT AAC CAA CTT TTA AGT TTA AAT CCT AGT CCT AAC ACT AAC TGA GCT AGG TAT TTA AAC GTA GTA CAA CGT TTT ACT ACC
199.1             GCA GAT AGG TTT AAC CAA CTT TTA AGT TTA AAT CCT AGT CCT AAC ACT AAC TGA GCT AGG TAT TTA AAC GTA GTA CAA CGT TTT ACT ACC
MEGA10366         GCA GAT AGG TTT AAC CAA CTT TTA AGT TTA AAT CCT AGT GCT AAC ACC AAC TGA GCT AGG TAT TTA AAC GTA GTA CAA CGT TTT ACT ACC
MEGA10378         GCA GAT AGG TTT AAC CAA CTT TTA AGT TTA AAT CCT AGT CCT AAC ACT AAC TGA GCT AGG TAT TTA AAC GTA GTA CAA CGT TTT ACT ACC
MEGA10467         GCA GAT AGG TTT AAC CAA CTT TTA AGT TTA AAT CCT AGT CCT AAC ACT AAC TGA GCT AGG TAT TTA AAC GTA GTA CAA CGT TTT ACT ACC
MEGA10477         GCA GAT AGG TTT AAC CAA CTT TTA AGT TTA AAT CCT AGT GCT AAC ACC AAC TGA GCT AGG TAT TTA AAC GTA GTA CAA CGT TTT ACT ACC
Sea1              GCA GAT AGG TTT AAT CAA CTT TTA AGT TTA AAT CCT AGT CCT AAC ACT AAC TGA GCT AGG TAT TTA AAC GTA GTA CAA CGT TTT ACT ACC
Sea2              GCA GAT AGG TTT AAC CAA CTT TTA AGT TTA AAT CCT AGT TCT AAC ACT AAC TGA GCT AGG TAT TTA AAC GTA GTA CAA CGT TTT ACT ACC
                  *** *** *** *** **  *** **  *** *** *** * * *** * *   * *** **  *** *** *** **  *** *** *** *** *** *** *** *** *** ***
G37          723   A   D   R   F   N   Q   L   L   S   L   N   P   N   P   N   T   N   W   A   R   Y   L   N   V   V   Q   R   F   T   T
                                                           S       S   D                       S
                                                                       S
                                                                       A

Consensus    725   A   D   R   F   N   Q   L   L   S   L   N   P   S   P   N   T   N   W   A   R   Y   L   N   V   V   Q   R   F   T   T
```

Asparagine Repeat Identified in SAPS Analysis
Shown in Box II of Figure 7B

N	Q	L	L	S	L	N	P	N	P	N	T	N	W	Sequence 1
N	Q	L	L	S	L	N	P	S	P	N	T	N	W	Sequence 2
N	Q	L	L	S	L	N	P	S	D	N	T	N	W	Sequence 3
N	Q	L	L	S	L	N	P	S	S	N	T	N	W	Sequence 4
N	Q	L	L	S	L	N	P	S	A	N	T	N	W	Sequence 5
N	Q	L	L	S	L	S	P	S	P	N	T	N	W	Sequence 6

Sequence 1: G37 & M6320
Sequence 2: M30, M2282, M2300, M6280, M6282, M6320, 64.1, 199.0, 199.1, MEGA10378, MEGA10467, & Sea1
Sequence 3: M2288
Sequence 4: M2321, M2341, M6257, M6284, M6285, & Sea2
Sequence 5: M6283, 6286, MEGA10366, & MEGA10477
Sequence 6: 64.0

**G37**	**2,257**	**GGA**	**CCT**	**AAC**	**CTT**	**GAT**	**AGT**	**TCT**	**ACC**	**TTC**	**GAT**	**CAG**	**TTC**
M30		GGA	CCT	AAC	CTT	GAT	AGT	TCT	ACC	TTC	GAT	CAG	TTC
M2282		GGA	CCT	AAC	CTT	GAT	AGT	TCT	ACC	TTC	GAT	CAG	TTC
M2288		GGA	CCT	AAC	CTT	GAT	GGT	TCT	ACC	TTC	GAT	CAG	TTC
M2300		GGA	CCT	GAC	CTT	GAT	AGT	TCT	ACC	TTC	GAT	CAG	TTC
M2321		GGA	CCT	AAC	CTT	GAT	GGT	TCT	ACC	TTC	GAT	CAG	TTC
M2341		GGA	CCT	AAC	TTT	GAT	GGT	TCT	ACC	TTC	GAT	CAG	TTC
M6257		GGA	CCT	AAC	TTT	GAT	GGT	TCT	ACC	TTC	GAT	CAG	TTC
M6280		GGA	CCT	AAC	CTT	GAT	AGT	TCT	ACC	TTC	GAT	CAG	TTC
M6282		GGA	CCT	AAC	CTT	GAT	AGT	TCT	ACC	TTC	GAT	CAG	TTC
M6283		GGA	CCT	AAC	CTT	GAT	GGT	TCT	ACC	TTC	GAT	CAG	TTC
M6284		GGA	CCT	AAC	CTT	GAT	AGT	TCT	ACC	TTC	GAT	CAG	TTC
M6285		GGA	CCT	AAC	CTT	GAT	GGT	TCT	ACC	TTC	GAT	CAG	TTC
6286		GGA	CCT	AAC	CTT	GAT	GGT	TCT	ACC	TTC	GAT	CAG	TTC
M6320		GGA	CCT	AAC	CTT	GAT	AGT	TCT	ACC	TTC	GAT	CAG	TTC
64.0		GGA	CCT	AAC	CTT	GAT	AGT	TCT	ACC	TTC	GAT	CAG	TTC
64.1		GGA	CCT	AAC	CTT	GAT	AGT	TCT	ACC	TTC	GAT	CAG	TTC
199.0		GGA	CCT	AAC	CTT	GAT	GGT	TCT	ACC	TTC	GAT	CAG	TTC
199.1		GGA	CCT	AAC	CTT	GAT	GGT	TCT	ACC	TTC	GAT	CAG	TTC
MEGA10366		GGA	CCT	AAC	CTT	GAT	GGT	TCT	ACC	TTC	GAT	CAG	TTC
MEGA10378		GGA	CCT	AAC	CTT	GAT	AGT	TCT	ACC	TTC	GAT	CAG	TTC
MEGA10467		GGA	CCT	AAC	CTT	GAT	GGT	TCT	ACC	TTC	GAT	CAG	TTC
MEGA10477		GGA	CCT	AAC	CTT	GAT	GGT	TCT	ACC	TTC	GAT	CAG	TTC
Sea1		GGA	CCT	AAC	CTT	GAT	AGT	TCT	ACC	TTC	GAT	CAG	TTC
Sea2		GGA	CCT	AAC	CTT	GAT	GGT	TCT	ACC	TTC	GAT	CAG	TTC
		***	***	**	**	***	**	***	***	***	***	***	***
**G37**	**753**	**G**	**P**	**N**	**L**	**D**	**S**	**S**	**T**	**F**	**D**	**Q**	**F**
				**D**	**F**		**G**						

**Consensus**	**755**	**G**	**P**	**N**	**L**	**D**	**G**	**S**	**T**	**F**	**D**	**Q**	**F**

Repeat Region EF (bp 2,293-2,877, aa 765-959)
30 Sequences Analyzed

**G37**	**2,293**	**TTA**	**GAC**	**TTT**	**CTC**	**CCC**	**TGA**	**ATC**	**GGC**	**AAT**	**GGT**	**AAA**	**CCC**	**TTT**	**TCC**	**AAC**	**TCC**	**CCC**	**---**	**---**	**TCC**	**CCT**	**TCA**	**---**	**---**	**---**	**---**	**---**	**---**	**---**	**ACT**
G37-vB		TTA	GAC	TTT	CTC	CCC	TGA	ATC	GGC	AAC	AAC	AAA	CCC	TTC	TCC	AAC	TCC	CCC	---	---	TCC	CCT	TCA	---	---	---	---	---	---	---	ACT
M30		TTA	GAC	TTT	CTC	CCC	TGA	ATC	GGC	AAC	AAC	AAA	CCC	TTC	TCC	AAC	TCC	CCC	---	---	TCC	CCT	TCA	---	---	---	---	---	---	---	ACT
M2282		TTA	GAC	TTT	CTC	CCC	TGA	ATC	GGC	AAT	GGT	AAA	CCC	TTC	TCC	AAC	TCC	CAC	ACT	GCT	TCC	CTT	TCT	---	---	---	---	---	---	---	---
M2288		TTA	GAC	TTT	CTC	CCC	TGA	ATC	GGC	AAC	AAC	AAA	CCC	TTC	TCC	AAC	TCC	CAC	ACT	GCT	ACC	CTT	TCT	---	---	---	---	---	---	---	---
M2300		TTA	GAC	TTT	CTC	CCC	TGA	ATC	GGC	AAC	AAC	AAA	CCC	TTC	TCC	AAC	TCC	CAC	ACT	GCT	ACC	CTT	TCT	---	---	---	---	---	---	---	---
M2321		TTA	GAC	TTT	CTC	CCC	TGA	ATC	GGC	AAT	GGT	TAT	TCC	TTC	TCC	AAC	TCC	CCC	---	---	TCC	CCT	TCA	---	---	---	---	---	---	---	ACT
M2341		TTA	GAC	TTT	CTC	CCC	TGA	ATC	GGC	AAT	GGT	AAA	GCT	TTT	TCC	AAC	TCC	CCC	---	---	TCC	CCT	TCT	TCT	TCT	TCT	---	---	---	---	ACT
M6257		TTA	GAC	TTT	CTC	CCC	TGA	ATC	GGC	AAT	GGT	AAA	GCT	TTC	TCC	AAC	TCC	CAC	ACT	GCT	TCT	---	TCT	TCT	---	---	---	---	---	---	---
M6280		TTA	GAC	TTT	CTC	CCC	TGA	ATT	GGC	AAT	GGT	AAA	GCT	TTT	TCC	AAC	TCC	CCC	---	---	TCC	CCT	TCT	TCT	TCT	TCT	TCT	TCT	TCT	TCT	ACT
M6282		TTA	GAT	TTT	CTC	CCC	TGA	ATC	GGC	AAT	GGT	AAA	CCC	TTC	TCC	AAC	TCC	CAC	ACT	GCT	ACC	CTT	TCT	---	---	---	---	---	---	---	---
M6283		TTA	GAC	TTT	CTC	CCC	TGA	ATC	GGC	AAT	GGT	TAT	TCC	TTC	TCC	AAC	TCC	CAC	ACC	TTT	TCT	GCT	TCT	TCT	---	---	---	---	---	---	---
M6284		TTA	GAC	TTT	CTC	CCC	TGA	ATC	GGC	AAT	GGT	AAA	CCC	TTC	TCC	AAC	TCC	CAC	ACT	GCT	ACC	CTT	TCT	---	---	---	---	---	---	---	---
M6285		TTA	GAC	TTT	CTC	CCC	TGA	ATC	GGC	AAC	AAC	AAA	CCC	TAT	TCC	AAC	TCC	CAC	ACT	GCT	ACC	CTT	TCT	---	---	---	---	---	---	---	---
6286		TTA	GAC	TTT	CTC	CCC	TGA	ATC	GGC	AAT	GGT	AAA	GCT	TTT	TCC	AAC	TCC	CAC	ACT	GCT	ACC	CTT	TCT	---	---	---	---	---	---	---	---
64.0		TTA	GAC	TTT	CTC	CCC	TGA	ATC	GGT	AAC	AAC	AAA	CCC	TTC	TCC	AAC	TCC	CCC	---	---	TCC	CCT	TCA	---	---	---	---	---	---	---	ACT
64.1		TTA	GAC	TTT	CTC	CCC	TGA	ATC	GGC	AAC	AAC	AAA	CCC	TTT	TCC	AAC	TCC	CAC	ACT	GCT	TCC	CTT	TCT	---	---	---	---	---	---	---	---
64.a		TTA	GAC	TTT	CTC	CCC	TGA	ATC	GGT	AAC	AAC	AAA	CCC	TTC	TCC	AAC	TCC	CCC	---	---	TCC	CCT	TCA	---	---	---	---	---	---	---	ACT
64.b		TTA	GAC	TTT	CTC	CCC	TGA	ATC	GGT	AAC	AAC	AAA	CCC	TTC	TCC	AAC	TCC	CCC	---	---	TCC	CCT	TCA	---	---	---	---	---	---	---	ACT
64.c		TTA	GAC	TTT	CTC	CCC	TGA	ATC	GGT	AAC	AAC	AAA	CCC	TTC	TCC	AAC	TCC	CCC	---	---	TCC	CCT	TCA	---	---	---	---	---	---	---	ACT
64.d		TTA	GAC	TTT	CTC	CCC	TGA	ATC	GGC	AAC	AAC	AAA	CCC	TTT	TCC	AAC	TCC	CAC	ACT	GCT	TCC	CTT	TCT	---	---	---	---	---	---	---	---
64.e		TTA	GAC	TTT	CTC	CCC	TGA	ATC	GGC	AAC	AAC	AAA	CCC	TTT	TCC	AAC	TCC	CAC	ACT	GCT	TCC	CTT	TCT	---	---	---	---	---	---	---	---
199.0		TTA	GAC	TTT	CTC	CCC	TGA	ATC	GGC	AAT	GGT	AAA	GCT	TTT	TCC	AAC	TCC	CAC	ACT	GCT	ACC	CTT	TCT	---	---	---	---	---	---	---	---
199.1		TTA	GAC	TTT	CTC	CCC	TGA	ATC	GGC	AAT	GGT	AAA	GCT	TTT	TCC	AAC	TCC	CAC	ACT	GCT	ACC	CTT	TCT	---	---	---	---	---	---	---	---
KOR10163.a		TTA	GAC	TTT	CTC	CCC	TGA	ATC	GGC	AAT	GGT	AAA	CCC	TTT	TCC	AAC	TCC	CAC	ACT	GCT	ACC	CTT	TCT	---	---	---	---	---	---	---	---
KOR10163.b		TTA	GAC	TTT	CTC	CCC	TGA	ATC	GGC	AAT	GGT	AAA	CCC	TTT	TCC	AAC	TCC	CAC	ACT	GCT	ACC	CTT	TCT	---	---	---	---	---	---	---	---
KOR10163.c		TTA	GAC	TTT	CTC	CCC	TGA	ATC	GGC	AAT	GGT	AAA	CCC	TTT	TCC	AAC	TCC	CAC	ACT	GCT	ACC	CTT	TCT	---	---	---	---	---	---	---	---
KOR10163.d		TTA	GAC	TTT	CTC	CCC	TGA	ATC	GGC	AAT	GGT	AAA	CCC	TTT	TCC	AAC	TCC	CAC	ACT	GCT	ACC	CTT	TCT	---	---	---	---	---	---	---	---
KOR10163.e		TTA	GAC	TTT	CTC	CCC	TGA	ATC	GGC	AAT	GGT	AAA	CCC	TTT	TCC	AAC	TCC	CAC	ACT	GCT	ACC	CTT	TCT	---	---	---	---	---	---	---	---
Sea1		TTA	GAC	TTT	CTC	CCC	TGA	ATC	GGC	AAC	AAC	AAA	CCC	TTT	TCC	AAC	TCC	CAC	ACT	GCT	TCC	CTT	TCT	---	---	---	---	---	---	---	---
Sea2		TTA	GAC	TTT	CTC	CCC	TGA	ATC	GGC	AAC	AAC	AAA	CCC	TTT	TCC	AAC	TCC	CCC	---	---	TCC	CCT	TCA	---	---	---	---	---	---	---	ACT
		***	**	***	***	***	***	**	**	**		*	*	*	***	***	***	* *			*		**								
**G37**	**765**	**L**	**D**	**F**	**L**	**P**	**W**	**I**	**G**	**N**	**G**	**K**	**P**	**F**	**S**	**N**	**S**	**P**	**–**	**–**	**S**	**P**	**S**	**–**	**–**	**–**	**–**	**–**	**–**	**–**	**T**
											**N**	**Y**	**S**	**Y**				**H**	**T**	**A**	**T**	**–**		**S**	**S**	**S**	**S**	**S**	**S**	**S**	**–**
													**A**							**F**		**L**									
																						**A**									
**Consensus**	**767**	**L**	**D**	**F**	**L**	**P**	**W**	**I**	**G**	**N**	**G**	**K**	**P**	**F**	**S**	**N**	**S**	**H**	**T**	**A**	**S**	**L**	**S**	**–**	**–**	**–**	**–**	**–**	**–**	**–**	**–**

Serine Repeat Identified in SAPS Analysis Shown in Box III of Figure 7B

**Sequence 1**	S	N	S	P	–	–	S	P	S	–	–	–	–	–	–	–	T
**Sequence 2**	S	N	S	P	–	–	S	P	S	–	–	–	–	–	–	–	T
**Sequence 3**	S	N	S	P	–	–	S	P	S	–	–	–	–	–	–	–	T
**Sequence 4**	S	N	S	H	T	A	S	L	S	–	–	–	–	–	–	–	–
**Sequence 5**	S	N	S	H	T	A	T	L	S	–	–	–	–	–	–	–	–
**Sequence 6**	S	N	S	H	T	A	T	L	S	–	–	–	–	–	–	–	–
**Sequence 7**	S	N	S	P	–	–	S	P	S	S	S	S	–	–	–	–	T
**Sequence 8**	S	N	S	H	T	A	S	–	S	–	–	–	–	–	–	–	–
**Sequence 9**	S	N	S	P	–	–	S	P	S	S	S	S	S	S	S	S	T
**Sequence 10**	S	N	S	H	T	F	S	A	S	–	–	–	–	–	–	–	–

**G37**	**2,356**	**---**	**TCC**	**GCT**	**TCC**	**TCT**	**TCT**	**ACC**	**CCC**	**CTC**	**CCC**	**ACT**	**TTT**	**TCT**	**AAC**	**ATC**	**AAT**	**GTT**	**GGG**	**GTT**	**AAA**	**TCA**	**ATG**	**ATC**	**ACT**	**CAA**	**CAT**	**TTA**	**AAT**	**AAA**	**GAA**
G37-vB		---	TCC	GCT	TCC	TCT	TCT	ACC	CCC	CTC	CCC	ACT	TTT	TCT	AAC	ATC	AAT	GTT	GGG	GTT	AAA	TCA	ATG	ATC	ACT	CAA	CAT	TTA	AAT	AAA	GAA
M30		---	TCT	GCT	TCC	TCA	---	ACC	CCA	CTC	CCC	ACT	TTT	TCT	AAC	ATC	GGG	GTA	GGG	GTT	AAA	TCA	ATG	ATC	ACT	CAA	CAC	TTA	AAC	AAA	GAG
M2282		---	GTT	---	AGT	TCA	AAT	ACC	CCC	CTC	CCC	ACT	TTT	TCT	AAC	ATC	AAT	GTT	GGG	GTT	AAA	TCA	ATG	ATC	ACT	CAA	CAT	TTA	AAC	AAA	GAG
M2288		---	TCT	---	TCT	TCT	---	TCC	CCC	CTC	CCC	ACT	TTT	TCT	AAC	ATC	AAT	GTC	GGG	GTT	AAA	TCA	ATG	ATC	ACT	CAA	CAC	TTA	AAC	AAA	GAG
M2300		---	GTT	---	AGT	TCA	AAT	ACC	CCC	CTC	CCC	ACT	TTT	TCT	AAC	ATC	AAT	GTT	GGG	GTT	AAA	TCA	ATG	ATC	ACT	CAA	CAC	TTA	AAC	AAA	GAG
M2321		---	TCT	GCT	TCC	---	---	ACC	CCC	CTC	CCC	ACT	TTT	TCT	AAC	ATC	AAT	GTT	GGG	GTT	AAA	TCT	GCC	ATT	ACC	ACT	CAT	TTA	AAT	AAA	GAA
M2341		---	TCC	---	TCT	TCT	---	ACC	CCA	CTC	CCC	ACT	TTT	TCT	AAC	ATC	AAT	GTC	GGA	GTT	AAA	TCT	GAT	ATC	ACT	AAA	CAC	TTA	AAC	AAA	GAA
M6257		---	GTT	---	AGT	TCA	ACT	ACC	CCC	CTC	CCC	ACT	TTT	TCT	AAC	ATC	AAT	GTT	GGG	GTT	CAA	TCT	GCG	ATT	ACT	TCT	CAT	CTC	AAC	AAA	GAA
M6280		---	TCC	---	TCT	TCT	---	ACC	CCC	CTC	CCC	ACT	TTT	TCT	AAC	ATC	AAT	GTC	GGG	GTT	AAA	TCA	ATG	ATC	ACT	CAA	CAT	TTA	AAC	AAA	GAA
M6282		---	GTT	---	AGT	TCA	AAT	ACC	CCC	CTC	CCC	ACT	TTT	TCT	AAC	ATC	AAT	GTT	GGG	GTT	AAA	TCT	GAT	ATC	ACT	CAA	CAT	TTA	AAT	AAA	GAG
M6283		GCT	TCC	---	TCA	---	---	ACC	CCC	CTC	CCC	ACT	TTT	TCT	AAC	ATC	AAT	GTC	GGG	GTT	AAA	TCA	ATG	ATC	ACT	AAA	CAC	TTA	AAC	AAA	GAA
M6284		---	GTT	---	AGT	TCA	AAT	ACC	CCC	CTC	CCC	ACT	TTT	TCT	AAC	ATC	AAT	GTT	GGG	GTT	AAA	TCA	ATG	ATC	ACT	CAA	CAT	TTA	AAT	CAG	CAA
M6285		---	GTT	---	AGT	TCA	AAT	ACC	CCC	CTC	CCC	ACT	TTT	TCT	AAC	ATC	AAT	GTT	GGG	GTT	AAA	TCA	ATG	ATC	ACT	CAA	CAC	TTA	AAT	CAG	CAA
6286		---	GTT	---	AGT	TCA	AAT	ACC	CCC	CTC	CCC	ACT	TTT	TCT	AAC	ATC	AAT	GTT	GGG	GTT	AAA	TCA	ATG	ATC	ACT	CAA	CAT	TTA	AAT	AAA	GAA
64.0		---	TCT	GCT	TCC	---	---	ACC	CCC	CTC	CCC	ACT	TTT	TCT	AAC	ATC	AAT	GTT	GGG	GTT	AAA	TCT	GAT	ATC	ACT	AAA	CAC	TTA	AAC	AAA	GAG
64.1		---	GTT	---	AGT	TCA	AAT	ACC	CCC	CTC	CCC	ACT	TTT	TCT	AAC	ATC	AAT	GTT	GGG	GTT	AAA	TCT	GAT	ATC	ACT	AAA	CAC	TTA	AAC	AAA	GAG
64.a		---	TCT	GCT	TCC	---	---	ACC	CCC	CTC	CAC	ACT	TTT	TCT	AAC	ATC	AAT	GTT	GGG	GTT	AAA	TCT	GAT	ATC	ACT	AAA	CAC	TTA	AAC	AAA	GAG
64.b		---	TCT	GCT	TCC	---	---	ACC	CCC	CTC	CCC	ACT	TTT	TCT	AAC	ATC	AAT	GTT	GGG	GTT	AAA	TCT	GAT	ATC	ACT	AAA	CAC	TTA	AAC	AAA	GAG
64.c		---	TCT	GCT	TCC	---	---	ACC	TCC	CTC	CCC	ACT	TTT	TCT	AAC	ATC	AAT	GTT	GGG	GTT	AAA	TCA	ATG	ATC	ACT	CAA	CAC	TTA	AAT	AAA	GAA
64.d		---	GTT	---	AGT	TCA	AAT	ACC	CCC	CTC	CCC	ACT	TTT	TCT	AAC	ATC	AAT	GTT	GGG	GTT	AAA	TCT	GAT	ATC	ACT	AAA	CAC	TTA	AAC	AAA	GAG
64.e		---	GTT	---	AGT	TCA	AAT	ACC	CCC	CTC	CCC	ACT	TTT	TCT	AAC	ATC	AAT	GTT	GGG	GTT	AAA	TCT	GAT	ATC	ACT	AAA	CAC	TTA	AAC	AAA	GAG
199.0		---	GTT	---	AGT	TCA	AAT	ACC	CCC	CTC	CCC	ACT	TTT	TCT	AAC	ATC	AAT	GTT	GGG	GTT	AAA	TCA	ATG	ATC	ACT	AAA	CAC	TTA	AAC	AAA	GAA
199.1		---	GTT	---	AGT	TCA	AAT	ACC	CCC	CTC	CCC	ACT	TTT	TCT	AAC	ATC	AAT	GTT	GGG	GTT	AAA	TCA	ATG	ATC	ACT	AAA	CAC	TTA	AAC	AAA	GAA
KOR10163.a		---	GTT	---	AGT	TCA	AAT	ACC	CCC	CTC	CCC	ACT	TTT	TCT	AAC	ATC	AAT	GTT	GGG	GTT	AAA	TCA	ATG	ATC	ACT	AAA	CAC	TTA	AAC	AAA	GAG
KOR10163.b		---	GTT	---	AGT	TCA	AAT	ACC	CCC	CTC	CCC	ACT	TTT	TCT	AAC	ATC	AAT	GTT	GGG	GTT	AAA	TCA	ATG	ATC	ACT	AAA	CAC	TTA	AAC	AAA	GAG
KOR10163.c		---	GTT	---	AGT	TCA	AAT	ACC	CCC	CTC	CCC	ACT	TTT	TCT	AAC	ATC	AAT	GTT	GGG	GTT	AAA	TCA	ATG	ATC	ACT	AAA	CAC	TTA	AAC	AAA	GAG
KOR10163.d		---	GTT	---	AGT	TCA	AAT	ACC	CCC	CTC	CCC	ACT	TTT	TCT	AAC	ATC	AAT	GTT	GGG	GTT	AAA	TCA	ATG	ACC	ACT	AAA	CAC	TTA	AAC	AAA	GAG
KOR10163.e		---	GTT	---	AGT	TCA	AAT	ACC	CCC	CTC	CCC	ACT	TTT	TCT	AAC	ATC	AAT	GTT	GGG	GTT	AAA	TCA	ATG	ATC	ACT	AAA	CAC	TTA	AAC	AAA	GAG
Sea1		---	GTT	---	AGT	TCA	AAT	ACC	CCC	CTC	CCC	ACT	TTT	TCT	AAC	ATC	AAT	GTT	GGG	GTT	AAA	TCA	ATG	ATC	ACT	CAA	CAC	TTA	AAC	AAA	GAG
Sea2		---	TCT	GCT	TCC	---	---	ACC	CCC	CTC	CCC	ACT	TTT	TCT	AAC	ATC	AAT	GTT	GGG	GTT	AAA	TCT	GAT	ATC	ACT	AAA	CAC	TTA	AAC	AAA	GAG
									*	***	***	***	***	***	***	***		**	**	***	**	**		*	**		**	*	**	*	*
**G37**	**786**	**–**	**S**	**A**	**S**	**S**	**S**	**T**	**P**	**L**	**P**	**T**	**F**	**S**	**N**	**I**	**N**	**V**	**G**	**V**	**K**	**S**	**M**	**I**	**T**	**Q**	**H**	**L**	**N**	**K**	**E**
		A	–	–		–	–	–	S		H						G				Q		A	T		K				Q	Q
			V				N	S															D			T					
							T																			S					
**Consensus**	**790**	**–**	**V**	**–**	**S**	**S**	**N**	**T**	**P**	**L**	**P**	**T**	**F**	**S**	**N**	**I**	**N**	**V**	**G**	**V**	**K**	**S**	**M**	**I**	**T**	**K**	**H**	**L**	**N**	**K**	**E**

Serine Repeat Identified in SAPS Analysis (continued)

**Sequence 1**	–	S	A	S	S	S	**Sequence 1:**	G37 & G37-vB
**Sequence 2**	–	S	A	–	S	S	**Sequence 2:**	M30
**Sequence 3**	–	S	A	–	–	S	**Sequence 3:**	M2321, 64.0, 64.a, 64.b, 64.c, & Sea2
**Sequence 4**	–	V	A	S	S	N	**Sequence 4:**	M2282, 64.1, 64.d, 64.e, & Sea1
**Sequence 5**	–	V	A	S	S	N	**Sequence 5:**	M2300, M6282, M6284, M6285, 6286, 199.0, 199.1, & KOR10163.a-d
**Sequence 6**	–	S	–	S	S	S	**Sequence 6:**	M2288
**Sequence 7**	–	–	–	S	S	S	**Sequence 7:**	M2341
**Sequence 8**	–	V	–	S	S	T	**Sequence 8:**	M6257
**Sequence 9**	–	–	–	S	S	S	**Sequence 9:**	M6280
**Sequence 10**	–	S	A	–	S	S	**Sequence 10:**	M6283

**G37**	**2,443**	**AAC**	**ACC**	**CGG**	**TGG**	**GTG**	**TTT**	**ATA**	**CCT**	**AAC**	**TTT**	**TCA**	**CCT**	**GAC**	**ATC**	**TGA**	**ACA**	**GGA**	**GCA**	**GGG**	**TAT**	**CGC**	**GTT**	**CAA**	**AGT**	**GCT**	**AAT**	**CAG**	**AAA**	**AAC**	**GGC**
G37-vB		AAC	ACC	CGG	TGG	GTG	TTT	ATA	CCT	AAC	TTT	TCA	CCT	GAC	ATC	TGA	ACA	GGA	GCA	GGG	TAT	CGC	GTT	CAA	AGT	GCT	AAT	CAG	AAA	AAC	GGC
M30		AAC	ACC	CGG	TGG	GTG	TTT	ACT	TCT	GGT	AGT	ACA	CCT	GAC	ATC	TGA	ACG	GGA	GCA	GGG	TAT	CGC	AAA	CAA	GGT	AAC	AAT	---	---	AAC	GGC
M2282		AAC	ACC	CGG	TGG	GTG	TTT	ACC	CCT	AAC	TCT	TCA	CCT	GAC	ATC	TGA	ACA	GGA	GCA	GGG	TAT	CGC	AAA	CAA	GGT	AAC	AAT	---	---	AAC	GGC
M2288		AAC	ACC	CGG	TGG	GTG	TTT	ACC	CCT	AAC	TCT	TCA	CCA	GAC	ATT	TGA	ACT	GGG	GCT	GGG	TAT	CGC	AAG	GAT	GCA	TCA	AAC	ACC	---	AGC	GGC
M2300		AAC	ACC	CGG	TGG	GTG	TTT	ACC	CCT	AAC	TCT	TCA	CCA	GAC	ATT	TGA	ACA	GGA	GCA	GGG	TAT	CGC	AAA	CAA	GGT	AAC	AAT	---	---	AAC	GGC
M2321		AAC	ACC	CGG	TGG	GTG	TTT	ACC	CCT	AAC	TCT	TCA	CCA	GAC	ATC	TGA	ACG	GGA	GCA	GGT	TAT	AGA	AAA	GCT	AAT	AAC	AAC	AAT	---	AAT	GGC
M2341		AAC	ACC	CGG	TGG	GTG	TTT	ACA	CCA	GAT	TCT	TCA	CCA	GAC	ATT	TGA	ACG	GGA	GCA	GGG	TAT	CGC	AAA	CAA	GGT	AAC	AAT	---	---	AAT	GGC
M6257		AAC	ACC	CGG	TGG	GTG	TTT	ACC	CCT	AAC	TCT	TCA	CCA	GAC	ATT	TGA	ACA	GGA	GCA	GGG	TAT	CGC	AAA	CAA	GGT	AAC	AAT	---	---	AAC	GGC
M6280		AAC	ACC	CGG	TGG	GTG	TTT	ATA	CCT	AAC	TCT	TCA	CCA	GAC	ATT	TGA	ACG	GGA	GCA	GGG	TAT	CGC	AAA	CAA	GGT	AAC	AAT	---	---	AAT	GGC
M6282		AAC	ACG	CGG	TGG	GTG	TTT	ACC	CCT	AAC	TCT	TCA	CCA	GGC	ATT	TGA	ACG	GGA	GCA	GGG	TAT	CGC	AAA	CAA	GGT	AAC	AAT	---	---	AAC	GGC
M6283		AAC	ACG	CGG	TGG	GTG	TTT	ATA	CCT	AAC	TCT	TCA	CCA	GAC	ATC	TGA	ACG	GGT	GCA	GGT	TAT	AGA	AAA	GCT	AAT	AAC	AAC	AAT	---	AAC	GGT
M6284		AAC	ACC	CGG	TGG	GTG	TTT	ATA	CCT	AAC	TCT	TCA	CCA	GAC	ATT	TGA	ACA	GGA	GCA	GGG	TAT	CGC	AAA	CAA	GGT	AAC	AAT	---	---	AAT	GGC
M6285		AAC	ACC	CGG	TGG	GTG	TTT	ACC	CCT	AAC	TCT	TCA	CCA	GAC	ATT	TGA	ACC	GGG	GCT	GGG	TAT	CGC	AAA	CAA	GGT	AAC	AAT	---	---	AAC	GGT
6286		AAC	ACC	CGG	TGG	GTG	TTT	ATA	CCT	AAC	TCT	TCA	CCT	GAC	ATC	TGA	ACA	GGA	GCA	GGT	TAT	AGA	AAA	GCT	AAT	AAC	AAC	AAT	---	AAC	GGC
64.0		AAC	ACC	CGG	TGG	GTG	TTT	ATA	CCT	AAC	TCT	TCA	CCT	GAC	ATT	TGA	ACG	GGA	GCA	GGT	TAT	AGA	AAA	GCT	AAT	AAC	AAC	AAT	---	AAC	GGC
64.1		AAC	ACC	CGG	TGG	GTG	TTT	ATA	CCT	AAC	TCT	TCA	CCT	GAC	ATC	TGA	ACT	GGG	GCT	GGG	TAT	CGC	AAA	CAA	GGT	AAC	AAT	---	---	AAT	GGC
64.a		AAC	ACC	CGG	TGG	GTG	TTT	ATA	CCT	AAC	TCT	TCA	CCT	GAC	ATT	TGA	ACG	GGA	GCA	GGT	TAT	AGA	AAA	GCT	AAT	AAC	AAC	AAT	---	AAC	GGC
64.b		AAC	ACC	CGG	TGG	GTG	TTT	ATA	CCT	AAC	TCT	TCA	CCT	GAC	ATT	TGA	ACG	GGA	GCA	GGT	TAT	AGA	AAA	GCT	AAT	AAC	AAC	AAT	---	AAC	GGC
64.c		AAC	ACC	CGG	TGG	GTG	TTT	ATA	CCT	AAC	TCT	TCA	CCT	GAC	ATC	TGA	ACG	GGA	GCA	GGG	TAT	CGC	AAA	CAA	GGT	AAC	AAT	---	---	AAC	GGC
64.d		AAC	ACC	CGG	TGG	GTG	TTT	ATA	CCT	AAC	TCT	TCA	CCT	GAC	ATC	TGA	ACT	GGG	GCT	GGG	TAT	CGC	AAA	CAA	GGT	AAC	AAT	---	---	AAT	GGC
64.e		AAC	ACC	CGG	TGG	GTG	TTT	ATA	CCT	AAC	TCT	TCA	CCT	GAC	ATC	TGA	ACT	GGG	GCT	GGG	TAT	CGC	AAA	CAA	GGT	AAC	AAT	---	---	AAT	GGC
199.0		AAC	ACC	CGG	TGG	GTG	TTT	ACC	CCT	AAC	TCT	TCA	CCT	GAC	ATT	TGA	ACG	GGA	GCA	GGT	TAT	AGA	AAA	GCT	AAT	AAC	AAC	AAT	---	AAT	GGC
199.1		AAC	ACC	CGG	TGG	GTG	TTT	ACC	CCT	AAC	TCT	TCA	CCT	GAC	ATT	TGA	ACG	GGA	GCA	GGT	TAT	AGA	AAA	GCT	AAT	AAC	AAC	AAT	---	AAT	GGC
KOR10163.a		AAC	ACC	CGG	TGG	GTG	TTT	ACC	CCT	AAC	TCT	TCA	CCT	GAC	ATC	TGA	ACG	GGA	GCA	GGG	TAT	CGC	AAA	CAA	GGT	AAC	AAT	---	---	AAT	GGC
KOR10163.b		AAC	ACC	CGG	TGG	GTG	TTT	ACC	CCT	AAC	TCT	TCA	CCT	GAC	ATC	TGA	ACG	GGA	GCA	GGG	TAT	CGC	AAA	CAA	GGT	AAC	AAT	---	---	AAT	GGC
KOR10163.c		AAC	ACC	CGG	TGG	GTG	TTT	ACC	CCT	AAC	TCT	TCA	CCT	GAC	ATC	TGA	ACG	GGA	GCA	GGG	TAT	CGC	AAA	CAA	GGT	AAC	AAT	---	---	AAT	GGC
KOR10163.d		AAC	ACC	CGG	TGG	GTG	TTT	ACC	CCT	AAC	TCT	TCA	CCT	GAC	ATC	TGA	ACG	GGA	GCA	GGG	TAT	CGC	AAA	CAA	GGT	AAC	AAT	---	---	AAT	GGC
KOR10163.e		AAC	ACC	CGG	TGG	GTG	TTT	ACC	CCT	AAC	TCT	TCA	CCT	GAC	ATC	TGA	ACG	GGA	GCA	GGG	TAT	CGC	AAA	CAA	GGT	AAC	AAT	---	---	AAT	GGC
Sea1		AAC	ACC	CGG	TGG	GTG	TTT	ATA	CCT	AAC	TTT	TCA	CCA	GAC	ATC	TGA	ACG	GGA	GCA	GGT	TAT	AGA	AAA	GCT	AAT	AAC	AAC	AAT	---	AAC	GGC
Sea2		AAC	ACC	CGG	TGG	GTG	TTT	ACC	CCT	AAC	TCT	TCA	CCA	GAC	ATT	TGA	ACA	GGA	GCA	GGT	TAT	AGA	AAA	GCT	AAT	AAC	AAC	AAT	AAT	AAT	GGC
		***	**	***	***	***	***	*	*		*	**	**	***	**	***	**	**	**	**	***	*					**			*	**
**G37**	**815**	**N**	**T**	**R**	**W**	**V**	**F**	**I**	**P**	**N**	**F**	**S**	**P**	**D**	**I**	**W**	**T**	**G**	**A**	**G**	**Y**	**R**	**V**	**Q**	**S**	**A**	**N**	**Q**	**K**	**N**	**G**
								**T**	**S**	**G**	**S**	**T**		**G**									**K**	**D**	**G**	**N**		**-**	**-**	**S**	
										**D**														**A**	**A**	**S**		**T**	**N**		
																							**N**			**N**			
**Consensus**	**818**	**N**	**T**	**R**	**W**	**V**	**F**	**T**	**P**	**N**	**S**	**S**	**P**	**D**	**I**	**W**	**T**	**G**	**A**	**G**	**Y**	**R**	**K**	**Q**	**G**	**N**	**N**	**-**	**-**	**N**	**G**

**G37**	**3,533**	**ATT**	**CCT**	**TTT**	**GAA**	**CAG**	**GTG**	**AAA**	**CCT**	**AGC**	**AAT**	**AAT**	**AGT**	**---**	**---**	**---**	**---**	**ACC**	**CCC**	**TTT**	**GAT**	**CCC**	**AAT**	**TCA**	**GAT**	**GAT**	**AAT**	**AAA**	**GTC**	**ACA**	**CCA**
G37-vB		ATT	CCT	TTT	GAA	CAG	GTG	AAA	CCT	AGC	AAT	AAT	AGT	---	---	---	---	ACC	CCC	TTT	GAT	CCC	AAT	TCA	GAT	GAT	AAT	AAA	GTC	ACA	CCA
M30		ATC	CCT	TTT	GAT	AAT	GTG	AAA	CCT	AGC	AAT	AGT	AGT	---	---	---	---	ACC	CCC	TTT	AAT	CCC	AAT	TCA	GAT	GAT	AAT	AAA	GTC	ACT	CAA
M2282		ATT	CCT	TTT	GAA	CAG	GTG	AAA	CCT	AGC	AAT	AAT	AGT	---	---	---	---	CAA	CAG	TTT	AAT	CCC	AAT	TCT	TCT	GAA	AAT	CAA	GTC	ACA	CCA
M2288		ATC	TCC	TTG	ACA	AGT	GTG	TTG	CCT	AGT	AGT	AGT	AGT	AGT	AGT	AGT	AGT	CCT	TCG	TTT	AAT	CCC	TCC	TCT	GCT	GAA	AAT	CAA	GTC	ACC	CCA
M2300		ATT	CCC	TTC	GAC	CAG	GTG	AAA	CCT	AGC	AAT	AAT	AGT	---	---	---	---	CAA	CAG	TTT	AAT	CCC	TCC	TCC	ATG	GAA	AAT	CAA	GTC	ACA	CCA
M2321		ATT	CCC	TTC	GAC	CAG	GTG	AAA	CCT	AGT	AGT	AGT	AGT	AGT	---	---	---	ACC	CAG	TTT	AAT	CCC	AAT	TTA	GAT	GAT	AAT	AAA	GTC	ACC	CCA
M2341		ATC	TCC	TTG	ACA	AGT	GTG	TTG	CCT	AGT	AGT	GGT	AGT	---	---	---	---	ACC	CAG	TTT	AAT	CCC	AAT	TCA	GAT	GAT	AAT	AAA	GTC	ACT	CAA
M6257		ATT	CCT	TTG	ACA	AGT	GTG	TTG	CCT	AGT	AGT	---	AGC	---	---	---	---	AAC	ACC	TTT	GAT	CCC	ACC	TCT	GCT	GAA	AAT	CAA	GTC	ACA	CCA
M6280		ATC	CCT	TTT	GAT	AGT	GTG	AAA	CCT	AGT	AGT	AGT	AGT	---	---	---	---	ACC	CCC	TTT	AAT	CCC	ACC	TCC	ATG	GAA	AAT	CAA	GTC	ACA	CCA
M6282		ATT	CCT	TTT	GAA	CAG	GTG	AAA	CCT	AGT	AAT	GGT	AGC	---	---	---	---	AAC	ACG	TTT	GAT	CCC	AAT	TCT	TCT	GAA	AAC	CAA	GTC	ACA	---
M6283		ATC	CCT	TTG	ACA	AGT	GTG	AAA	CCT	AGT	AGT	AGT	AGT	AGT	AGT	AGT	AGT	CCT	TCG	TTT	GAT	CCC	AAT	TCA	GAT	GAT	AAT	AAA	GTC	ACC	CCA
M6284		ATC	TCC	TTG	ACA	AGT	GTG	TTG	CCT	AGT	AGC	AAT	AAT	AGT	---	---	---	CAA	CAG	TTT	AAT	CCC	AAT	TCA	GAT	GAT	AAT	AAA	GTC	ACT	CAA
M6285		ATC	CCT	TTG	ACA	AGT	GTG	TTG	CCT	AGT	AGT	AGT	AGT	AGT	AGT	---	---	ACC	CAG	TTT	AAT	CCC	TCC	TCT	GCT	GAA	AAT	CAA	GTC	---	---
6286		ATT	CCT	TTG	ACA	AGT	GTG	TTG	CCT	AGT	AGT	AGT	AGT	AGT	---	---	---	ACC	CAG	TTT	AAT	CCC	AAT	TCA	GAT	GAT	AAC	AAA	GTC	ACA	CCA
64.0		ATC	CCT	TTT	GAT	AGT	GTG	AAA	CCT	AGC	AAT	AAT	AGT	---	---	---	---	ACC	CCC	TTT	GAT	CCC	AAT	TCA	GAT	GAT	AAT	AAA	GTC	ACA	CCA
64.1		ATC	TCC	TTG	ACA	AGT	GTG	TTG	CCT	AGT	AGC	AAT	AGT	AGT	---	---	---	CAA	CAG	TTT	AAT	CCC	TCC	TCC	ATG	GAA	AAC	CAA	GTC	ACT	---
64.a		ATC	TCC	TTG	ACA	AGT	GTG	TTG	CCT	AGT	AGC	AAT	AGT	AGT	---	---	---	CAA	CAG	TTT	AAT	CCC	TCC	TCC	ATG	GAA	AAC	CAA	GTC	ACT	---
64.b		ATC	CCT	TTT	GAT	AGT	GTG	AAA	CCT	AGC	AAT	AAT	AGT	---	---	---	---	ACC	CCC	TTT	GAT	CCC	AAT	TCA	GAT	GAT	AAT	AAA	GTC	ACA	CCA
64.c		ATC	CCT	TTT	GAT	AAT	GTG	AAA	CCT	AGC	AAT	AAT	AGT	---	---	---	---	ACC	CCC	TTT	GAT	CCC	AAT	TCA	GAT	GAT	AAT	AAA	GTC	ACT	---
64.d		ATC	TCC	TTG	ACA	AGT	GTG	TTG	CCT	AGT	AGC	AAT	AGT	AGT	---	---	---	CAA	CAG	TTT	AAT	CCC	TCC	TCC	ATG	GAA	AAC	CAA	GTC	ACT	---
64.e		ATC	TCC	TTG	ACA	AGT	GTG	TTG	CCT	AGT	AGC	AAT	AGT	AGT	---	---	---	CAA	CAG	TTT	AAT	CCC	TCC	TCC	ATG	GAA	AAC	CAA	GTC	ACT	---
199.0		ATC	CCC	TTA	ACA	AGT	GTG	TTG	CCT	AGT	AGT	AGT	AGT	AGC	---	---	---	AAC	ACG	TTT	AAT	CCC	AAT	TTA	GAT	GAT	AAT	AAA	GTC	ACA	CCA
199.1		ATC	CCC	TTA	ACA	AGT	GTG	TTG	CCT	AGT	AGT	AGT	AGT	AGC	---	---	---	AAC	ACG	TTT	AAT	CCC	AAT	TTA	GAT	GAT	AAT	AAA	GTC	ACA	CCA
KOR10163.a		ATC	TCC	TTG	ACA	AGT	GTG	TTG	CCT	AGT	AGC	AAT	AGT	AGT	---	---	---	ACC	CCC	TTT	GAT	CCC	AAT	TCT	TCT	GAA	AAT	CAA	GTC	ACT	---
KOR10163.b		ATC	TCC	TTG	ACA	AGT	GTG	TTG	CCT	AGT	AGC	AAG	AGT	AGT	---	---	---	ACC	CCC	TTT	GAT	CCC	AAT	TCT	TCT	GAA	AAT	CAA	GTC	ACT	---
KOR10163.c		ATC	TCC	TTG	ACA	AGT	GTG	TTG	CCT	AGT	AGC	AAT	AGT	AGT	---	---	---	ACC	CCC	TTT	GAT	CCC	AAT	TCT	TCT	GAA	AAT	CAA	GTC	ACT	---
KOR10163.d		ATC	TCC	TTG	ACA	AGT	GTG	TTG	CCT	AGT	AGC	AAT	AGT	AGT	---	---	---	ACC	CCC	TTT	GAT	CCC	AAT	TCT	TCT	GAA	AAT	CAA	GTC	ACT	---
KOR10163.e		ATC	TCC	TTG	ACA	AGT	GTG	TTG	CCT	AGT	AGC	AAT	AGT	AGT	---	---	---	ACC	CCC	TTT	GAT	CCC	AAT	TCT	TCT	GAA	AAT	CAA	GTC	ACC	---
Sea1		ATC	CCT	TTT	GAT	AAT	GTG	AAA	CCT	AGC	AAT	GGT	AGT	---	---	---	---	ACC	CCC	TTT	AAT	CCC	AAT	TTA	GAT	GAT	AAT	AAA	GTC	ACT	---
Sea2		ATT	CCC	TTC	GAC	CAG	GTG	AAA	CCT	AGT	AAT	GGT	AGT	---	---	---	---	AAC	ACG	TTT	AAT	CCC	ACC	TCT	GCT	GAA	AAT	CAA	GTC	ACA	CCA
		**	*	**			***		***	**	*		*							***	**	***		*		**	**	**	***		
**G37**	**845**	**I**	**P**	**F**	**E**	**Q**	**V**	**K**	**P**	**S**	**N**	**N**	**S**	**-**	**-**	**-**	**-**	**T**	**P**	**F**	**D**	**P**	**N**	**S**	**D**	**D**	**N**	**K**	**V**	**T**	**P**
			**S**	**L**	**D**	**N**		**L**			**S**	**-**	**N**	**S**	**S**	**S**	**S**	**Q**	**Q**		**N**		**S**	**L**	**S**	**E**		**Q**		**-**	**-**
					**T**	**S**						**S**						**P**	**S**				**T**		**A**						**Q**
												**G**						**N**	**T**						**M**						
												**K**																			
**Consensus**	**846**	**I**	**P**	**L**	**T**	**S**	**V**	**L**	**P**	**S**	**S**	**N**	**S**	**S**	**-**	**-**	**-**	**T**	**P**	**F**	**N**	**P**	**N**	**S**	**D**	**E**	**N**	**Q**	**V**	**T**	**P**

**G37**	**3,611**	**TCA**	**---**	**---**	**---**	**GGT**	**GGC**	**---**	**TCC**	**TCC**	**---**	**AAA**	**---**	**CCA**	**ACC**	**ACC**	**TAT**	**---**	**CCT**	**GCT**	**TTA**	**CCC**	**AAC**	**AGT**	**ATC**	**AGT**	**CCC**	**ACC**	**AGT**	**GAC**	**TGG**
G37-vB		TCA	---	---	---	GGT	GGC	---	TCC	TCC	---	AAA	---	CCA	ACC	ACC	TAT	---	CCT	GCT	TTA	CCC	AAC	AGT	ATC	AGT	CCC	ACC	AGT	GAC	TGG
M30		---	---	---	GGT	GGT	GGC	---	ACT	CAA	ACC	AAA	AAA	ACA	ACC	ACC	TAC	---	ACC	CAT	TTA	CCC	AAC	AGT	ATC	AGT	CCC	ACC	AGT	GAC	TGA
M2282		TCA	---	---	---	GGT	GGC	---	TCC	CAA	GCC	AAA	AAA	---	ACA	ACG	TAT	---	GAC	AAT	TTA	CCC	AAC	AGT	ATC	AGT	CCC	ACC	AGT	GAC	TGG
M2288		---	---	---	GCT	GGT	GGC	---	TCC	TCC	---	AAA	AAA	CCA	ACC	ACC	TAC	---	ACC	CAT	TTA	CCC	AAC	AGT	ATC	AGT	CCC	ACC	AGT	GAC	TGG
M2300		TCA	---	---	---	GGT	GGC	---	TCC	CCA	GCC	AAA	AAA	---	ACA	ACG	TAT	---	GAC	AAT	TTA	CCA	AAC	TCC	ATC	AGT	CCC	ACC	AGT	GAC	TGG
M2321		---	---	---	GCT	GGT	---	AGC	TCC	CCC	---	AAA	AAA	---	ACA	ACG	TAT	---	GAT	GCT	TTA	CCC	AAC	AGT	ATC	AGT	CCC	ACC	AGT	GAC	TGG
M2341		---	---	---	---	GGT	GGT	AGC	TCC	TCA	GCC	AAA	AAA	ACA	ACC	ACC	TAT	TAT	TCC	TTT	TTA	CCC	AAC	AGT	ATC	AGT	CCC	ACC	AGT	GAC	TGG
M6257		TCA	---	---	---	GGT	GGC	---	TCC	TCC	---	AAA	AAA	---	ACA	ACG	TAT	---	GAT	GCT	TTA	CCC	AAC	AGT	ATC	AGT	CCC	ACC	AGT	GAC	TGG
M6280		TCA	---	---	---	GGT	---	AGC	TCC	GCC	---	AAA	AAA	---	ACA	ACG	TAT	---	GAC	AAT	TTA	CCA	AAC	TCC	ATC	AGT	CCC	ACC	AGT	GAC	TGA
M6282		TCA	TCA	TCA	---	GGT	---	AGC	TCC	CCA	GCC	AAA	AAA	ACA	ACC	ACC	TAT	TAT	TCC	TTT	TTA	CCA	AAC	TCC	ATC	AGT	CCC	ACC	AGT	GAC	TGG
M6283		---	---	---	GCT	GGT	---	AGC	TCC	CAA	GCC	AAA	AAA	ACA	ACC	ACC	TAT	---	TCC	TTT	TTA	CCC	AAC	AGT	ATC	AGT	CCC	ACC	AGT	GAC	TGG
M6284		---	---	---	---	GGT	GGC	---	TCC	TCC	---	AAA	AAA	---	ACA	ACG	TAT	---	GAC	AAT	TTA	CCA	AAC	TCC	ATC	AGT	CCC	ACC	AGT	GAC	TGG
M6285		---	---	---	---	---	---	AAC	TCC	---	---	AAA	AAA	---	ACT	ACA	TAC	---	ACC	CAT	TTA	CCA	AAC	TCC	ATC	AGT	CCC	ACC	AGT	GAC	TGG
6286		TCA	---	---	---	GGT	GGC	---	TCC	GCC	---	AAA	AAA	ACA	ACC	ACC	TAT	---	CCT	GCT	TTA	CCA	AAC	TCA	ATC	AGT	CCC	ACC	AGT	GAC	TGG
64.0		TCA	---	---	---	GGT	GGC	---	TCC	TCC	---	AAA	---	CCA	ACC	ACC	TAC	---	ACC	CAT	TTA	CCC	AAC	AGT	ATC	AGT	CCC	ACC	AGT	GAC	TGA
64.1		TCA	---	---	---	GGT	GGC	---	TCC	CCA	GCC	AAA	AAA	ACT	ACC	ACC	TAT	---	CCT	GCT	TTA	CCA	AAC	TCC	ATC	AGT	CCC	ACC	AGT	GAC	TGA
64.a		TCA	---	---	---	GGT	GGC	---	TCC	CCA	GCC	AAA	AAA	ACT	ACC	ACC	TAT	---	CCT	GCT	TTA	CCA	AAC	TCC	ATC	AGT	CCC	ACC	AGT	GAC	TGA
64.b		TCA	---	---	---	GGT	GGC	---	TCC	TCC	---	AAA	---	CCA	ACC	ACC	TAC	---	ACC	CAT	TTA	CCC	AAC	AGT	ATC	AGT	CCC	ACC	AGT	GAC	TGA
64.c		TCA	---	---	---	GGT	---	AGC	TCC	TCC	---	AAA	---	CCA	ACC	ACC	TAC	---	ACC	CAT	TTA	CCC	AAC	AGT	ATC	AGT	CCC	ACC	AGT	GAC	TGA
64.d		TCA	---	---	---	GGT	GGC	---	TCC	CCA	GCC	AAA	AAA	ACT	ACC	ACC	TAT	---	CCT	GCT	TTA	CCA	AAC	TCC	ATC	AGT	CCC	ACC	AGT	GAC	TGA
64.e		TCA	---	---	---	GGT	GGC	---	TCC	CCA	GCC	AAA	AAA	ACT	ACC	ACC	TAT	---	CCT	GCT	TTA	CCA	AAC	TCC	ATC	AGT	CCC	ACC	AGT	GAC	TGA
199.0		TCA	---	---	---	GGT	---	AGC	TCC	TCC	---	AAA	AAA	---	ACA	ACG	TAT	---	GAC	AAT	TTA	CCA	AAC	TCC	ATC	AGT	CCC	ACC	AGT	GAC	TGG
199.1		TCA	---	---	---	GGT	---	AGC	TCC	TCC	---	AAA	AAA	---	ACA	ACG	TAT	---	GAC	AAT	TTA	CCA	AAC	TCC	ATC	AGT	CCC	ACC	AGT	GAC	TGG
KOR10163.a		TCA	---	---	GCT	GGT	GGC	---	TCC	CCA	GCC	AAA	AAA	---	ACA	ACG	TAT	---	GAC	AAT	TTA	CCC	AAC	AGT	ATC	AGT	CCC	ACC	AGT	GAC	TGG
KOR10163.b		TCA	---	---	GCT	GGT	GGC	---	TCC	CCA	GCC	AAA	AAA	---	ACA	ACG	TAT	---	GAC	AAT	TTA	CCC	AAC	AGT	ATC	AGT	CCC	ACC	AGT	GAC	TGG
KOR10163.c		TCA	---	---	GCT	GGT	GGC	---	TCC	CCA	GCC	AAA	AAA	---	ACA	ACG	TAT	---	GAC	AAT	TTA	CCC	AAC	AGT	ATC	AGT	CCC	GCC	AGT	GAC	TGG
KOR10163.d		TCA	---	---	GCT	GGT	GGC	---	TCC	CCA	GCC	AAA	AAA	---	ACA	ACG	TAT	---	GAC	AAT	TTA	CCC	AAC	AGT	ATC	AGT	CCC	ACC	AGT	GAC	TGG
KOR10163.e		TCA	---	---	GCT	GGT	GGC	---	TCC	CCA	GCC	AAA	AAA	---	ACA	ACG	TAT	---	GAC	AAT	TTA	CCC	AAC	AGT	ATC	AGT	CCC	ACC	AGT	GAC	TGG
Sea1		TCA	---	---	---	GGT	---	AGC	TCC	CCA	GCC	AAA	AAA	ACT	ACC	ACC	TAC	---	ACC	CAT	TTA	CCC	AAC	AGT	ATC	AGT	CCC	ACC	AGT	GAC	TGG
Sea2		TCA	---	---	---	GGT	---	AGC	TCC	TCC	---	AAA	AAA	---	ACA	ACG	TAT	---	GAT	GCT	TTA	CCA	AAC	TCC	ATC	AGT	CCC	ACC	AGT	GAC	TGA
									*			***			**	**	**			*	***	**	***		***	***	***	**	***	***	**
**G37**	**871**	**S**	**-**	**-**	**-**	**G**	**G**	**-**	**S**	**S**	**-**	**K**	**-**	**P**	**T**	**T**	**Y**	**-**	**P**	**A**	**L**	**P**	**N**	**S**	**I**	**S**	**P**	**T**	**S**	**D**	**W**
		-	S	S	G	-	-	S	T	-	T		K	-				Y	T	H								A			
					A			N		Q	A			T					D	N											
										P									S	F											
										A																					
**Consensus**	**873**	**S**	**-**	**-**	**-**	**G**	**G**	**-**	**S**	**S**	**-**	**K**	**K**	**-**	**T**	**T**	**Y**	**-**	**D**	**A**	**L**	**P**	**N**	**S**	**I**	**S**	**P**	**T**	**S**	**D**	**W**

**G37**	**3,680**	**ATC**	**AAT**	**GCA**	**TTG**	**ACT**	**TTC**	**ACT**	**AAT**	**AAG**	**AAT**	**AAC**	**CCG**	**CAG**	**CGC**	**AAT**	**CAA**	**CTG**	**TTG**	**CTC**	**AGA**	**AGC**	**TTA**	**CTA**	**GGA**	**ACT**	**ATT**	**CCG**	**GTC**	**TTG**	**ATC**
G37-vB		ATC	AAT	GCA	TTG	ACT	TTC	ACT	AAT	AAG	AAT	AAC	CCG	CAG	CGC	AAT	CAA	CTG	TTG	CTC	AGA	AGC	TTA	CTA	GGA	ACT	ATT	CCG	GTC	TTG	ATC
M30		AGC	AAT	GCG	TTG	ACT	TTC	ACC	AAT	AAG	AAT	AAC	CCG	CAA	AGA	AAT	CAG	TTG	TTG	TTA	AGG	GGG	TTA	CTA	GGA	ACG	ATC	CCG	GTA	TTG	ATC
M2282		ATC	AAC	GCA	TTA	ACC	TTC	ACC	AAT	AAG	AAT	AAC	CCG	CAG	CGC	AAT	CAA	CTG	TTG	TTA	AGA	GCA	CTG	TTA	GGA	ACT	ATT	CCG	GTC	TTG	ATC
M2288		ATC	AAC	GCA	TTA	ACC	TTC	ACC	AAT	AAG	AAT	AAT	CCG	CAG	CGC	AAT	CAG	TTG	TTG	TTA	AGA	GCA	CTG	TTA	GGA	ACG	ATC	CCG	GTC	TTG	ATC
M2300		ATC	AAT	GCA	TTA	ACC	TTC	ACC	AAT	AAG	AAT	AAC	CCC	CAG	CGC	AAT	CAG	TTG	TTG	TTA	AGA	GCA	CTG	TTA	GGA	ACT	ATC	CCG	GTC	TTG	ATC
M2321		ATC	AAT	GCG	TTG	ACT	TTC	ACC	AAT	AAG	AAT	AAC	CCG	CAA	AGA	AAC	CAG	TTG	TTG	TTA	AGA	GCG	TTA	TTA	GGA	ACT	ATT	CCG	GTA	TTG	ATC
M2341		ATC	AAC	GCA	TTG	ACT	TTC	ACT	AAC	AAG	AAT	AAT	CCG	CAG	CGC	AAT	CAG	TTG	TTA	CTA	AGA	GCA	CTG	TTA	GGA	ACG	ATC	CCG	GTA	TTG	ATC
M6257		ATC	AAT	GCG	TTG	ACT	TTC	ACT	AAT	AAG	AAT	AAC	CCG	CAG	CGC	AAT	CAG	TTG	TTG	TTA	AGA	GCA	CTG	TTA	GGA	ACG	ATC	CCG	GTC	TTG	ATC
M6280		AGC	AAT	GCG	TTG	ACT	TTC	ACC	AAC	AAG	AAT	AAC	CCG	CAG	CGC	AAT	CAG	TTG	TTA	CTA	AGA	GCA	CTG	TTA	GGA	ACT	ATT	CCG	GTC	TTG	ATC
M6282		ATC	AAC	GCA	TTA	ACC	TTC	ACT	AAT	AAG	AAC	AAT	CCA	CAA	CGC	AAT	CAG	TTG	TTG	TTA	AGG	GGG	TTG	TTA	GGA	ACT	ATT	CCG	GTA	TTG	ATC
M6283		ATC	AAT	GCG	TTG	ACT	TTC	ACT	AAT	AAG	AAC	AAC	CCG	CAG	CGC	AAT	CAG	TTG	TTG	CTC	AGA	AGC	TTA	CTA	GGA	ACG	ATC	CCG	GTA	TTG	ATC
M6284		ATC	AAC	GCA	TTG	ACT	TTT	ACT	AAT	AAG	AAT	AAC	CCG	CAG	CGC	AAT	CAA	CTG	TTG	TTA	AGA	GCG	TTA	CTA	GGA	ACG	ATC	CCG	GTC	TTG	ATC
M6285		ATC	AAC	GCA	TTA	ACC	TTC	ACT	AAT	AAA	AAT	AAC	CCG	CAG	CGC	AAT	CAG	TTG	TTA	CTC	AGA	AGC	TTA	CTA	GGA	ACG	ATC	CCG	GTA	TTG	ATC
6286		ATC	AAT	GCG	TTG	ACT	TTC	ACT	AAT	AAG	AAT	AAC	CCG	CAG	CGA	AAT	CAG	TTG	TTG	CTC	AGA	AGC	TTA	CTA	GGA	ACG	ATC	CCG	GTA	TTG	ATC
64.0		AGC	AAT	GCG	TTG	ACT	TTC	ACT	AAC	AAG	AAT	AAT	CCG	CAA	CGA	AAT	CAA	CTG	TTG	TTA	AGA	GCG	TTA	TTA	GGA	ACT	ATT	CCG	GTC	TTG	ATC
64.1		AGC	AAT	GCG	TTG	ACT	TTC	ACC	AAT	AAG	AAT	AAC	CCG	CAG	CGC	AAT	CAA	CTG	TTG	TTA	AGA	GCG	TTA	TTA	GGA	ACT	ATT	CCG	GTC	TTG	ATC
64.a		AGC	AAT	GCG	TTG	ACT	TTC	ACT	AAC	AAG	AAT	AAT	CCG	CAA	CGA	AAT	CAA	CTG	TTG	TTA	AGA	GCG	TTA	TTA	AGA	ACT	ATT	CCG	GTC	TTG	ATC
64.b		AGC	AAT	GCG	TTG	ACT	TTC	ACT	AAC	AAG	AAT	AAT	CCG	CAA	CGA	AAT	CAA	CTG	TTG	TTA	AGA	GCG	TTA	TTA	GGA	ACT	ATT	CCG	GTC	TTG	ATC
64.c		AGC	AAT	GCG	TTG	ACT	TTC	ACT	AAC	AAG	AAT	AAT	CCG	CAA	CGA	AAT	CAA	CTG	TTG	TTA	AGA	GCG	TTA	TTA	GGA	ACT	ATT	CCG	GTC	TTG	ATC
64.d		AGC	AAT	GCG	TTG	ACT	TTC	ACC	AAT	AAG	AAT	AAC	CCG	CAG	CGC	AAT	CAA	CTG	TTG	TTA	AGA	GCG	TTA	TTA	GGA	ACT	ATT	CCG	GTC	TTG	ATC
64.e		AGC	AAT	GCG	TTG	ACT	TTC	ACC	AAT	AAG	AAT	AAC	CCG	CAG	CGC	AAT	CAA	CTG	TTG	TTA	AGA	GCG	TTA	TTA	GGA	ACT	ATT	CCG	GTA	TTG	ATC
199.0		ATC	AAC	GCA	TTA	ACC	TTC	ACC	AAT	AAG	AAT	AAC	CCG	CAG	CGC	AAT	CAG	TTG	TTG	TTA	AGG	GGG	TTG	TTA	GGA	ACT	ATT	CCG	GTG	TTG	ATC
199.1		ATC	AAC	GCA	TTA	ACC	TTC	ACC	AAT	AAG	AAT	AAC	CCG	CAG	CGC	AAT	CAG	TTG	TTG	TTA	AGG	GGG	TTG	TTA	GGA	ACT	ATT	CCG	GTG	TTG	ATC
KOR10163.a		ATC	AAC	GCA	TTG	ACT	TTC	ACT	AAC	AAG	AAT	AAC	CCG	CAA	CGA	AAC	CAA	CTG	TTG	CTC	AGA	AGC	TTA	CTA	GGA	ACT	ATT	CCG	GTC	TTG	ATC
KOR10163.b		ATC	AAC	GCA	TTG	ACT	TTC	ACT	AAC	AAG	AAT	AAC	CCG	CAA	CGA	AAC	CAA	CTG	TTG	CTC	AGA	AGC	TTA	CTA	GGA	ACT	ATT	CCG	GTC	TTG	ATC
KOR10163.c		ATC	AAC	GCA	TTG	ACT	TTC	ACT	AAC	AAG	AAT	AAC	CCG	CAA	CGA	AAC	CAA	CTG	TTG	CTC	AGA	AGC	TTA	CTA	GGA	ACT	ATT	CCG	GTC	TTG	ATC
KOR10163.d		ATC	AAC	GCA	TTG	ACT	CTC	ACT	AAC	AAG	AAT	AAC	CCG	CAC	CGA	AAC	CAA	CTG	TTG	CTC	AGA	AGC	TTA	CTA	GGA	ACT	ATT	CCG	GTC	TTG	ATC
KOR10163.e		ATC	AAC	GCA	TTG	ACT	TTC	ACT	AAC	AAG	AAT	AAC	CCG	CAA	CGA	AAC	CAA	CTG	TTG	CTC	AGA	AGC	TTA	CTA	GGA	ACT	ATT	CCG	GTC	TTG	ATC
Sea1		ATC	AAT	GCG	TTG	ACT	TTC	ACT	AAC	AAG	AAT	AAT	CCA	CAA	CGC	AAT	CAA	CTG	TTG	TTA	AGA	GCG	TTA	CTA	GGA	ACT	ATT	CCG	GTA	TTG	ATC
Sea2		AGC	AAT	GCG	TTG	ACT	TTC	ACC	AAT	AAG	AAT	AAC	CCG	CAA	CGC	AAT	CAG	TTG	TTG	TTA	AGA	GCA	CTG	TTA	GGA	ACC	ATC	CCG	GTC	TTG	ATC
		* *	**	**	**	**	*	**	**	**	**	**	**	**	*	**	**	**	**	*	**		*	**	**	**	**	***	**	***	***
**G37**	**894**	**I**	**N**	**A**	**L**	**T**	**F**	**T**	**N**	**K**	**N**	**N**	**P**	**Q**	**R**	**N**	**Q**	**L**	**L**	**L**	**R**	**S**	**L**	**L**	**G**	**T**	**I**	**P**	**V**	**L**	**I**
		**S**					**L**							**H**								**G**			**R**						
																						**A**									
**Consensus**	**896**	**I**	**N**	**A**	**L**	**T**	**F**	**T**	**N**	**K**	**N**	**N**	**P**	**Q**	**R**	**N**	**Q**	**L**	**L**	**L**	**R**	**A**	**L**	**L**	**G**	**T**	**I**	**P**	**V**	**L**	**I**

Repetitive Structure Identified in SAPS Analysis
Shown in Box IV of Figure 7B (& w/ homology to sequence in Box I) **N Q L L**

**G37**	**3,770**	**AAT**	**AAG**	**AGT**	**GGG**	**GAT**	**AGT**	**AAT**	**GAT**	**CAA**	**TTT**	**AAC**	**AAG**	**GAT**	**AGT**	**GAG**	**CAG**	**AAA**	**TGG**	**GAT**	**AAA**	**ACT**	**GAG**	**ACA**	**AAT**	**GAG**	**GGT**	**AAT**	**TTA**	**CCT**	**GGG**
G37-vB		AAT	AAG	AGT	GGG	GAT	AGT	AAT	GAT	CAA	TTT	AAC	AAG	GAT	AGT	GAG	CAG	AAA	TGG	GAT	AAA	ACT	GAG	ACA	AAT	GAG	GGT	AAT	TTA	CCT	GGG
M30		AAT	AAG	AGT	GGA	ACG	GGA	GAT	GAG	---	TTT	AAC	CAT	ACG	AAT	GAT	CAG	AAG	TGG	GAT	AAA	ACT	GAG	ACA	AAT	GAG	GGT	AAT	TTA	CCT	GGG
M2282		AAT	AAG	AGT	GGG	GAT	AGT	AAT	GAT	CAA	TTT	AAC	AAG	GAT	AGT	GAG	CAG	AAA	TGG	GAT	AAA	ACG	AAT	GAA	AAA	GAT	GGG	AAT	TTA	CCT	GGG
M2288		AAT	AAG	AGT	GGA	GGA	AGT	GGG	AAT	GAG	TTT	AAC	CAT	ACG	AAT	GAT	CAG	AAG	TGG	GAT	AAA	ACT	GAG	ACA	AAT	GAG	GGC	AAC	CTC	CCG	GGG
M2300		AAT	AAG	AGT	GGA	ACG	GGA	GAT	GAG	---	TTT	ACC	CAT	ACG	AGT	GAG	CAG	AAA	TGG	GAT	AAA	ACG	AAT	GAA	AAA	GAT	GGG	AAT	TTA	CCT	GGG
M2321		AAT	AAG	AGT	GGG	GAT	AGT	AAT	GAT	CAA	TTT	AAC	AAG	GAT	AGT	GAG	CAG	CAG	TGA	AAC	GAA	ACA	GAA	AAA	CCA	GGA	GGC	AAC	CTC	CCG	GGG
M2341		AAT	AAG	AGT	GGA	ACG	GGA	GAT	GAG	---	TTT	ACC	CAT	ACG	AGT	GAG	CAG	AAG	TGG	GAT	AAA	ACG	AAT	GAA	AAA	GAT	GGG	AAT	TTA	CCT	GGG
M6257		AAT	AAG	AGT	GGG	GAG	AGT	AGT	GAG	CAA	TTT	GAA	CAG	---	AGT	GAT	CAG	AAA	TGG	GAT	AAA	ACG	AAT	GAA	AAA	GAT	GGG	AAT	TTA	CCT	GGG
M6280		AAT	AAG	AGT	GGG	GAG	GGT	GGG	AAT	GAG	TTT	ACC	AAG	GAT	AGT	GAT	CAG	AAG	TGA	AAC	GAA	ACA	GAT	AAA	TTA	GGA	GGT	AAC	CTC	CCG	GGG
M6282		AAT	AAG	AGT	GGG	GAT	AGT	AAT	GAT	CAA	TTT	AAC	AAG	GAT	AGT	GAG	CAG	AAG	TGG	GAT	AAA	ACT	GAG	ACA	AAT	GAG	GGC	AAC	CTC	CCA	GGG
M6283		AAT	AAG	AGT	GGG	GAT	AGT	AAT	GAT	CAA	TTT	AAC	AAG	GAT	AGT	GAG	CAG	AAG	TGG	GAT	AAG	ACA	GAA	ACC	AAA	GAT	GGC	AAC	CTC	CCG	GGG
M6284		AAT	AAG	AGT	GGA	GGA	AGT	GGG	AAT	GAG	TTT	AAC	CAT	ACG	AGT	GAG	CAG	AAA	TGG	GAT	AAA	ACG	AAT	GAA	AAA	GAT	GGG	AAT	TTA	CCT	GGG
M6285		AAT	AAG	AGT	GGG	GAG	GGT	GGG	GAA	GAG	TTT	ACC	CAT	ACG	AGT	GAC	CAG	AAG	TGG	GAT	AAA	ACT	GAG	ACC	AAG	GAA	GGC	AAC	CTC	CCA	GGG
6286		AAT	AAG	AGT	GGA	GGA	AGT	GGG	AAT	GAG	TTT	AAC	CAT	ACG	AGT	GAT	CAG	AAG	TGG	GAT	AAA	ACG	AAT	GAA	AAA	GAT	GGG	AAT	TTA	CCT	GGG
64.0		AAT	AAG	AGT	GGG	GAT	AGT	AAT	GAT	CAA	TTT	AAC	AAG	GAT	AGT	GAG	CAA	AAA	TGA	AAC	GAA	ACA	GAG	AAA	CCA	GGA	GGC	AAC	CTC	CCG	GGG
64.1		AAT	AAG	AGT	GGG	GAT	AGT	AAT	GAT	CAA	TTT	AAC	AAG	GAT	AGT	GAG	CAA	AAA	TGA	AAC	GAA	ACA	GAG	AAA	CCA	GGA	GGC	AAC	CTC	CCG	GGG
64.a		AAT	AAG	AGT	GGG	GAT	AGT	AAT	GAT	CAA	TTT	AAC	AAG	GAT	AGT	GAG	CAA	AAA	TGA	AAC	GAA	ACA	GAG	AAA	CCA	GGA	GGC	AAC	CTC	CCG	GGG
64.b		AAT	AAG	AGT	GGG	GAT	AGT	AAT	GAT	CAA	TTT	AAC	AAG	GAT	AGT	GAG	CAA	AAA	TGA	AAC	GAA	ACA	GAG	AAA	CCA	GGA	GGC	AAC	CTC	CCG	GGG
64.c		AAT	AAG	AGT	GGG	GAT	AGT	AAT	GAT	CAA	TTT	AAC	AAG	GAT	AGT	GAG	CAA	AAA	TGA	AAC	GAA	ACA	GAG	AAA	CCA	GGA	GGC	AAC	CTC	CCG	GGG
64.d		AAT	AAG	AGT	GGG	GAT	AGT	AAT	GAT	CAA	TTT	AAC	AAG	GAT	AGT	GAG	CAA	AAA	TGA	AAC	GAA	ACA	GAG	AAA	CCA	GGA	GGC	AAC	CTC	CCG	GGG
64.e		AAT	AAG	AGT	GGA	GGA	AGT	GGG	AAT	GAG	TTT	AAC	AAG	GAT	AGT	GAG	CAA	AAA	TGA	AAC	GAA	ACA	GAT	AAA	TTA	GGA	GGT	AAC	CTC	CCG	GGG
199.0		AAT	AAG	AGT	GGA	ACG	GGG	GGT	CAG	GAG	TTT	ACC	CAT	ACG	AAT	GAC	CAG	AAA	TGA	AAC	GAA	ACA	GAG	AAA	CCA	GGA	GGC	AAC	CTC	CCG	GGG
199.1		AAT	AAG	AGT	GGA	ACG	GGG	GGT	CAG	GAG	TTT	ACC	CAT	ACG	AAT	GAC	CAG	AAA	TGA	AAC	GAA	ACA	GAG	AAA	CCA	GGA	GGC	AAC	CTC	CCG	GGG
KOR10163.a		AAT	AAG	AGT	GGG	GAT	AGT	AAT	GAT	CAA	TTT	AAC	AAG	GAT	AGT	GAG	CAG	AAA	TGG	GAT	AAA	ACT	GAG	ACA	AAT	GAG	GGT	AAT	TTA	CCT	GGG
KOR10163.b		AAT	AAG	AGT	GGG	GAT	AGT	AAT	GAT	CAA	TTT	AAC	AAG	GAT	AGT	GAG	CAG	AAA	TGG	GAT	AAA	ACT	GAG	ACA	AAT	GAG	GGT	AAT	TTA	CCT	GGG
KOR10163.c		AAT	AAG	AGT	GGG	GAT	AGT	AAT	GAT	CAA	TTT	AAC	AAG	GAT	AGT	GAG	CAG	AAA	TGG	GAT	AAA	ACT	GAG	ACA	AAT	GAG	GGT	AAT	TTA	CCT	GGG
KOR10163.d		AAT	AAG	AGT	GGG	GAT	AGT	AAT	GAT	CAA	TTT	AAC	AAG	GAT	AGT	GAG	CAG	AAA	TGG	GAT	AAA	ACT	GAG	ACA	AAT	GAG	GGT	AAT	TTA	CCT	GGG
KOR10163.e		AAT	AAG	AGT	GGG	GAT	AGT	AAT	GAT	CAA	TTT	AAC	AAG	GAT	AGT	GAG	CAG	AAA	TGG	GAT	AAA	ACT	GAG	ACA	AAT	GAG	GGT	AAT	TTA	CCT	GGG
Sea1		AAT	AAG	AGT	GGA	ACG	GGA	GAT	GAG	---	TTT	ACC	CAT	ACG	AGT	GAG	CAG	AAA	TGG	GAT	AAA	ACG	AAT	GAA	AAA	GAT	GGG	AAT	TTA	CCT	GGG
Sea2		AAT	AAG	AGT	GGA	ACG	GGG	GGT	CAG	GAG	TTT	ACC	CAT	ACG	AGT	GAG	CAG	AAG	TGG	GAT	AAA	ACT	GAG	ACA	AAT	GAG	GGT	AAT	TTA	CCT	GGG
		***	***	***	**				*		***		*		* *	**	**	*	**	*	*	**	*				**	**	*	**	***
**G37**	**924**	**N**	**K**	**S**	**G**	**D**	**S**	**N**	**D**	**Q**	**F**	**N**	**K**	**D**	**S**	**E**	**Q**	**K**	**W**	**D**	**K**	**T**	**E**	**T**	**N**	**E**	**G**	**N**	**L**	**P**	**G**
						**T**	**G**	**D**	**E**	**-**		**T**	**H**	**-**	**N**	**D**		**Q**		**N**	**E**		**N**	**E**	**K**	**D**					
						**G**		**G**	**N**	**E**		**E**	**Q**	**T**									**D**	**K**	**P**	**G**					
						**E**		**S**	**Q**																**L**						
**Consensus**	**926**	**N**	**K**	**S**	**G**	**D**	**S**	**N**	**D**	**Q**	**F**	**N**	**K**	**D**	**S**	**E**	**Q**	**K**	**W**	**D**	**K**	**T**	**E**	**T**	**N**	**E**	**G**	**N**	**L**	**P**	**G**

**G37**	**3,860**	**TTT**	**GGG**	**GAG**	**GTG**	**AAT**	**GGG**
G37-vB		TTT	GGG	GAG	GTG	AAT	GGG
M30		TTT	GGG	GAG	GTG	AAT	GGG
M2282		TTT	GGG	GAG	GTG	AAT	GGG
M2288		TTT	GGG	GAG	GTG	AAT	GGG
M2300		TTT	GGG	GAG	GTG	AAT	GGG
M2321		TTT	GGG	GAG	GTG	AAT	GGG
M2341		TTT	GGG	GAG	GTG	AAT	GGG
M6257		TTT	GGG	GAG	GTG	AAT	GGG
M6280		TTT	GGG	GAG	GTG	AAT	GGG
M6282		TTT	GGG	GAG	GTG	AAT	GGG
M6283		TTT	GGG	GAG	GTG	AAT	GGG
M6284		TTT	GGG	GAG	GTG	AAT	GGG
M6285		TTT	GGG	GAG	GTG	AAT	GGG
6286		TTT	GGG	GAG	GTG	AAT	GGG
64.0		TTT	GGG	GAA	GTG	AAT	GGG
64.1		TTT	GGG	GAA	GTG	AAT	GGG
64.a		TTT	GGG	GAA	GTG	AAT	GGG
64.b		TTT	GGG	GAA	GTG	AAT	GGG
64.c		TTT	GGG	GAA	GTG	AAT	GGG
64.d		TTT	GGG	GAA	GTG	AAT	GGG
64.e		TTT	GGG	GAG	GTG	AAT	GGG
199.0		TTT	GGG	GAG	GTG	AAT	GGG
199.1		TTT	GGG	GAG	GTG	AAT	GGG
KOR10163.a		TTT	GGG	GAG	GTG	AAT	GGG
KOR10163.b		TTT	GGG	GAG	GTG	AAT	GGG
KOR10163.c		TTT	GGG	GAG	GTG	AAT	GGG
KOR10163.d		TTT	GGG	GAG	GTG	AAT	GGG
KOR10163.e		TTT	GGG	GAG	GTG	AAT	GGG
Sea1		TTT	GGG	GAA	GTG	AAT	GGG
Sea2		TTT	GGG	GAG	GTG	AAT	GGG
		***	***	**	***	***	***
**G37**	**954**	**F**	**G**	**E**	**V**	**N**	**G**

**Consensus**	**956**	**F**	**G**	**E**	**V**	**N**	**G**

Conserved Region 3 (bp 2,878-3,306, aa 960-1,102)
25 Sequences Analyzed

**G37**	**2,878**	**TTG**	**TAT**	**AAT**	**GCC**	**GCA**	**TTA**	**CTC**	**CAT**	**ACC**	**TAT**	**GGT**	**TTT**	**TTT**	**GGC**	**ACC**	**AAT**	**ACC**	**AAC**	**TCT**	**ACT**	**GAT**	**CCT**	**AAG**	**ATA**	**GGT**	**TTT**	**AAA**	**GCT**	**GAT**	**AGT**
M30		TTG	TAT	AAT	GCC	GCA	TTA	CTC	CAT	ACC	TAT	GGT	TTT	TTT	GGC	ACC	AAT	ACC	AAC	TCT	ACT	GAT	CCT	AAG	ATA	GGT	TTT	AAA	GCT	GAT	AGT
M2282		TTG	TAT	AAT	GCC	GCA	TTA	CTC	CAT	ACC	TAT	GGT	TTT	TTT	GGC	ACC	AAT	ACC	AAC	TCT	ACT	GAT	CCT	AAG	ATA	GGT	TTT	AAA	GCT	GAT	AGT
M2288		TTG	TAT	AAT	GCC	GCA	TTA	CTT	TAT	ACC	TAT	GGT	TTT	TTT	GGC	ACC	AAT	ACC	AAT	AAC	TCA	GAT	CCC	AAA	ATA	GGC	TTT	AAA	GCT	GAT	AGT
M2300		TTG	TAT	AAT	GCC	GCA	TTA	CTC	CAT	ACC	TAT	GGT	TTT	TTT	GGC	ACC	AAT	ACC	AAC	TCT	ACT	GAT	CCT	AAG	ATA	GGT	TTT	AAA	GCT	GAT	AGT
M2321		TTG	TAT	AAT	GCC	GCA	TTA	CTC	CAT	ACC	TAT	GGC	TTT	TTT	GGC	ACT	AAT	ACC	AAT	AAT	TCA	GAT	CCC	AAG	ATA	GGT	TTT	AAA	GCT	GAT	AGT
M2341		TTG	TAT	AAT	GCC	GCA	TTA	CTC	CAT	ACC	TAT	GGT	TTT	TTT	GGC	ACC	AAT	ACC	AAC	TCT	ACT	GAT	CCT	AAG	ATA	GGT	TTT	AAA	GCT	GAT	AGT
M6257		TTG	TAT	AAT	GCC	GCA	TTA	CTC	CAT	ACC	TAT	GGC	TTT	TTT	GGC	ACT	AAT	ACC	AAT	AAT	TCA	GAT	CCC	AAG	ATA	GGC	TTT	AAA	GCT	GAT	AGT
M6280		TTG	TAT	AAT	GCC	GCA	TTA	CTC	CAT	ACC	TAT	GGT	TTT	TTT	GGC	ACC	AAT	ACC	AAC	TCT	ACT	GAT	CCT	AAG	ATA	GGC	TTT	AAA	GCT	GAT	AGT
M6282		TTG	TAT	AAT	GCC	GCA	TTA	CTC	CAT	ACC	TAT	GGT	TTT	TTT	GGC	ACC	AAT	ACC	AAC	TCT	ACT	GAT	CCT	AAG	ATA	GGC	TTT	AAA	GCT	GAT	AGT
M6283		TTG	TAT	AAT	GCC	GCA	TTA	CTT	TAT	ACC	TAT	GGT	TTT	TTT	GGC	ACC	AAT	ACC	AAT	AAC	TCA	GAT	CCC	AAG	ATA	GGC	TTT	AAA	GCT	GAT	AGT
M6284		TTG	TAT	AAT	GCC	GCA	TTA	CTC	CAT	ACC	TAT	GGT	TTT	TTT	GGC	ACC	AAT	ACC	AAC	TCT	ACT	GAT	CCT	AAG	ATA	GGT	TTT	AAA	GCT	GAT	AGT
M6285		TTG	TAT	AAT	GCC	GCA	TTA	CTC	CAT	ACC	TAT	GGC	TTT	TTT	GGC	ACT	AAT	ACC	AAT	AAT	TCA	GAT	CCC	AAG	ATA	GGT	TTT	AAA	GCT	GAT	AGT
6286		TTG	TAT	AAT	GCC	GCA	TTA	CTT	TAT	ACC	TAT	GGT	TTT	TTT	GGC	ACC	AAT	ACC	AAT	AAC	TCA	GAT	CCC	AAG	ATA	GGC	TTT	AAA	GCT	GAT	AGT
M6320		TTG	TAT	AAT	GCC	GCA	TTA	CTC	CAT	ACC	TAT	GGT	TTT	TTT	GGC	ACC	AAT	ACC	AAC	TCT	ACT	GAT	CCT	AAG	ATA	GGT	TTT	AAA	GCT	GAT	AGT
64.0		TTG	TAT	AAT	GCC	GCA	TTA	CTC	CAT	ACC	TAT	GGT	TTT	TTT	GGC	ACC	AAT	ACC	AAC	TCT	ACT	GAT	CCT	AAG	ATA	GGC	TTT	AAA	GCT	GAT	AGT
64.1		TTG	TAT	AAT	GCC	GCA	TTA	CTC	CAT	ACC	TAT	GGT	TTT	TTT	GGC	ACC	AAT	ACC	AAC	TCT	ACT	GAT	CCT	AAG	ATA	GGC	TTT	AAA	GCT	GAT	AGT
199.0		TTG	TAT	AAT	GCC	GCA	TTA	CTT	TAT	ACC	TAT	GGT	TTT	TTT	GGC	ACC	AAT	ACC	AAT	AAC	TCA	GAT	CCC	AAG	ATA	GGC	TTT	AAA	GCT	GAT	AGT
199.1		TTG	TAT	AAT	GCC	GCA	TTA	CTT	TAT	ACC	TAT	GGT	TTT	TTT	GGC	ACC	AAT	ACC	AAT	AAC	TCA	GAT	CCC	AAG	ATA	GGC	TTT	AAA	GCT	GAT	AGT
MEGA10366		TTG	TAT	AAT	GCC	GCA	TTA	CTT	TAT	ACC	TAT	GGT	TTT	TTT	GGC	ACC	AAT	ACC	AAT	AAC	TCA	GAT	CCC	AAG	ATA	GGC	TTT	AAA	GCT	GAT	AGT
MEGA10378		TTG	TAT	AAT	GCC	GCA	TTA	CTC	CAT	ACC	TAT	GGT	TTT	TTT	GGC	ACC	AAT	ACC	AAC	TCT	ACT	GAT	CCT	AAG	ATA	GGT	TTT	AAA	GCT	GAT	AGT
MEGA10467		TTG	TAT	AAT	GCC	GCA	TTA	CTC	CAT	ACC	TAT	GGT	TTT	TTT	GGC	ACC	AAT	ACC	AAC	TCT	ACT	GAT	CCT	AAG	ATA	GGC	TTT	AAA	GCT	GAT	AGT
MEGA10477		TTG	TAT	AAT	GCC	GCA	TTA	CTT	TAT	ACC	TAT	GGT	TTT	TTT	GGC	ACC	AAT	ACC	AAT	AAC	TCA	GAT	CCC	AAG	ATA	GGC	TTT	AAA	GCT	GAT	AGT
Sea1		TTG	TAT	AAT	GCC	GCA	TTA	CTC	CAT	ACC	TAT	GGT	TTT	TTT	GGC	ACC	AAT	ACC	AAC	TCT	ACT	GAT	CCT	AAG	ATA	GGC	TTT	AAA	GCT	GAT	AGT
Sea2		TTG	TAT	AAT	GCC	GCA	TTA	CTC	CAT	ACC	TAT	GGC	TTT	TTT	GGC	ACT	AAT	ACC	AAT	AAT	TCA	GAT	CCC	AAG	ATA	GGT	TTT	AAA	GCT	GAT	AGT
		***	***	***	***	***	***	**	**	***	***	**	***	***	***	**	***	***	**		*	***	**	**	***	**	***	***	***	***	***
**G37**	**960**	**L**	**Y**	**N**	**A**	**A**	**L**	**L**	**H**	**T**	**Y**	**G**	**F**	**F**	**G**	**T**	**N**	**T**	**N**	**S**	**T**	**D**	**P**	**K**	**I**	**G**	**F**	**K**	**A**	**D**	**S**
									**Y**											**N**	**S**										

**Consensus**	**962**	**L**	**Y**	**N**	**A**	**A**	**L**	**L**	**H**	**T**	**Y**	**G**	**F**	**F**	**G**	**T**	**N**	**T**	**N**	**S**	**T**	**D**	**P**	**K**	**I**	**G**	**F**	**K**	**A**	**D**	**S**

Serine Repeat Identified in SAPS Analysis
Shown in Box V of Figure 7B

**Sequence 1**	**K**	**A**	**D**	**S**
**Sequence 2**	**K**	**A**	**D**	**S**
**Sequence 3**	**K**	**A**	**D**	**S**
**Sequence 4**	**K**	**A**	**D**	**S**
**Sequence 5**	**K**	**A**	**D**	**S**
**Sequence 6**	**K**	**A**	**D**	**S**
**Sequence 7**	**K**	**A**	**D**	**S**

Conserved Region 3 (bp 2,878–3,306; aa 960–1,102)

**G37**	**2,968**	**AGT**	**AGT**	**AGT**	**AGT**	**AGT**	**AGT**	**---**	**---**	**---**	**---**	**---**	**ACA**	**CTA**	**GTA**	**GGT**	**AGT**	**GGG**	**TTA**	**AAC**	**TGA**	**ACT**	**AGT**	**CAG**	**GAT**	**GTA**	**GGT**	**AAT**	**CTT**	**GTT**	**GTA**
M30		AGT	AGT	AGT	AGT	AGT	---	---	---	---	---	---	ACA	CTA	GTA	GGT	AGT	GGG	TTA	AAC	TGA	ACT	AGT	CAG	GAT	GTA	GGT	AAT	CTT	GTT	GTA
M2282		AGT	AGT	AGT	AGT	AGT	AGT	---	---	---	---	---	ACA	CTA	GTA	GGT	AGT	GGG	TTA	AAC	TGA	ACT	AGT	CAG	GAT	GTA	GGT	AAT	CTT	GTT	GTA
M2288		AGT	AGT	AGT	AGT	AGT	AGT	AGT	AGT	AGT	AGT	---	ACA	CTA	GTA	GGT	AGT	GGG	TTA	AAC	TGA	ACT	AGT	CAG	GAT	GTA	GGT	AAT	CTT	GTT	GTA
M2300		AGT	AGT	AGT	AGT	---	---	---	---	---	---	---	ACA	CTA	GTA	GGT	AGT	GGG	TTA	AAC	TGA	ACT	AGT	CAG	GAT	GTA	GGT	AAT	CTT	GTT	GTA
M2321		AGT	AGT	AGT	AGT	AGT	AGT	AGT	---	---	---	---	ACA	CTA	GTA	GGT	AGT	GGG	TTA	AAC	TGA	ACT	AGT	CAG	GAT	GTA	GGT	AAT	CTT	GTT	GTA
M2341		AGT	AGT	AGT	AGT	AGT	AGT	---	---	---	---	---	ACA	CTA	GTA	GGT	AGT	GGG	TTA	AAC	TGA	ACT	AGT	CAG	GAT	GTA	GGT	AAT	CTT	GTT	GTA
M6257		AGT	AGT	AGT	AGT	AGT	AGT	AGT	---	---	---	---	ACA	CTA	GTA	GGT	AGT	GGG	TTA	AAC	TGA	ACT	AGT	CAG	GAT	GTA	GGT	AAT	CTT	GTT	GTA
M6280		AGT	AGT	AGT	AGT	AGT	AGT	---	---	---	---	---	ACA	CTA	GTA	GGT	AGT	GGG	TTA	AAC	TGA	ACT	AGT	CAG	GAT	GTA	GGT	AAT	CTT	GTT	GTA
M6282		AGT	AGT	AGT	AGT	AGT	---	---	---	---	---	---	ACA	CTA	GTA	GGT	AGT	GGG	TTA	AAC	TGA	ACT	AGT	CAG	GAT	GTA	GGT	AAT	CTT	GTT	GTA
M6283		AGT	AGT	AGT	AGT	AGT	---	---	---	---	---	---	ACA	CTA	GTA	GGT	AGT	GGG	TTA	AAC	TGA	ACT	AGT	CAG	GAT	GTA	GGT	AAT	CTT	GTT	GTA
M6284		AGT	AGT	AGT	AGT	AGT	---	---	---	---	---	---	ACA	CTA	GTA	GGT	AGT	GGG	TTA	AAC	TGA	ACT	AGT	CAG	GAT	GTA	GGT	AAT	CTT	GTT	GTA
M6285		AGT	AGT	AGT	AGT	AGT	AGT	AGT	AGT	AGT	---	---	ACA	CTA	GTA	GGT	AGT	GGG	TTA	AAC	TGA	ACT	AGT	CAG	GAT	GTA	GGT	AAT	CTT	GTT	GTA
6286		AGT	AGT	AGT	AGT	AGT	---	---	---	---	---	---	ACA	CTA	GTA	GGT	AGT	GGG	TTA	AAC	TGA	ACT	AGT	CAG	GAT	GTA	GGT	AAT	CTT	GTT	GTA
M6320		AGT	AGT	AGT	AGT	AGT	---	---	---	---	---	---	ACA	CTA	GTA	GGT	AGT	GGG	TTA	AAC	TGA	ACT	AGT	CAG	GAT	GTA	GGT	AAT	CTT	GTT	GTA
64.0		AGT	AGT	AGT	AGT	AGT	AGT	AGT	AGT	AGT	AGT	AGT	ACA	CTA	GTA	GGT	AGT	GGG	TTA	AAC	TGA	ACT	AGT	CAG	GAT	GTA	GGT	AAT	CTT	GTT	GTA
64.1		AGT	AGT	AGT	AGT	AGT	---	---	---	---	---	---	ACA	CTA	GTA	GGT	AGT	GGG	TTA	AAC	TGA	ACT	AGT	CAG	GAT	GTA	GGT	AAT	CTT	GTT	GTA
199.0		AGT	AGT	AGT	AGT	AGT	AGT	---	---	---	---	---	ACA	CTA	GTA	GGT	AGT	GGG	TTA	AAC	TGA	ACT	AGT	CAG	GAT	GTA	GGT	AAT	CTT	GTT	GTA
199.1		AGT	AGT	AGT	AGT	AGT	---	---	---	---	---	---	ACA	CTA	GTA	GGT	AGT	GGG	TTA	AAC	TGA	ACT	AGT	CAG	GAT	GTA	GGT	AAT	CTT	GTT	GTA
MEGA10366		AGT	AGT	AGT	AGT	AGT	AGT	---	---	---	---	---	ACA	CTA	GTA	GGT	AGT	GGG	TTA	AAC	TGA	ACT	AGT	CAG	GAT	GTA	GGT	AAT	CTT	GTT	GTA
MEGA10378		AGT	AGT	AGT	AGT	AGT	AGT	AGT	---	---	---	---	ACA	CTA	GTA	GGT	AGT	GGG	TTA	AAC	TGA	ACT	AGT	CAG	GAT	GTA	GGT	AAT	CTT	GTT	GTA
MEGA10467		AGT	AGT	AGT	AGT	AGT	AGT	AGT	---	---	---	---	ACA	CTA	GTA	GGT	AGT	GGT	TTA	AAC	TGA	ACT	AGT	CAG	GAT	GTA	GGT	AAT	CTT	GTT	GTA
MEGA10477		AGT	AGT	AGT	AGT	AGT	---	---	---	---	---	---	ACA	CTA	GTA	GGT	AGT	GGG	TTA	AAC	TGA	ACT	AGT	CAG	GAT	GTA	GGT	AAT	CTT	GTT	GTA
Sea1		AGT	AGT	AGT	AGT	---	---	---	---	---	---	---	ACA	CTA	GTA	GGT	AGT	GGG	TTA	AAC	TGA	ACT	AGT	CAG	GAT	GTA	GGT	AAT	CTT	GTT	GTA
Sea2		AGT	AGT	AGT	AGT	AGT	AGT	AGT	AGT	AGT	---	---	ACA	CTA	GTA	GGT	AGT	GGG	TTA	AAC	TGA	ACT	AGT	CAG	GAT	GTA	GGT	AAT	CTT	GTT	GTA
		***	***	***	***								***	***	***	***	***	**	***	***	***	***	***	***	***	***	***	***	***	***	***
**G37**	**990**	**S**	**S**	**S**	**S**	**S**	**S**	**–**	**–**	**–**	**–**	**–**	**T**	**L**	**V**	**G**	**S**	**G**	**L**	**N**	**W**	**T**	**S**	**Q**	**D**	**V**	**G**	**N**	**L**	**V**	**V**
						**–**	**–**	**S**	**S**	**S**	**S**	**S**																			
**Consensus**	**992**	**S**	**S**	**S**	**S**	**S**	**S**	**–**	**–**	**–**	**–**	**–**	**T**	**L**	**V**	**G**	**S**	**G**	**L**	**N**	**W**	**T**	**S**	**Q**	**D**	**V**	**G**	**N**	**L**	**V**	**V**

Serine Repeat Identified in SAPS Analysis (continued)

**Sequence 1**	S	S	S	S	–	–	–	–	–	–	–	T	L	V
**Sequence 2**	S	S	S	S	S	–	–	–	–	–	–	T	L	V
**Sequence 3**	S	S	S	S	S	S	–	–	–	–	–	T	L	V
**Sequence 4**	S	S	S	S	S	S	S	–	–	–	–	T	L	V
**Sequence 5**	S	S	S	S	S	S	S	S	S	–	–	T	L	V
**Sequence 6**	S	S	S	S	S	S	S	S	S	S	–	T	L	V
**Sequence 7**	S	S	S	S	S	S	S	S	S	S	S	T	L	V

Sequence 1: M2300 & Sea1
Sequence 2: M30, M6282, M6283, M6284, 6286, M6230, 64.1, 199.1, & MEGA10467
Sequence 3: G37, M2282, M2341, M6280, 199.0, & MEGA10366
Sequence 4: M2321, M6257, MEGA10378, & MEGA10467
Sequence 5: M6285 & Sea2
Sequence 6: M2288
Sequence 7: 64.0

**G37**	**3,043**	**ATC**	**AAT**	**GAC**	**ACC**	**AGC**	**TTT**	**GGG**	**TTT**	**CAA**	**CTT**	**GGT**	**GGT**	**TGG**	**TTT**	**ATT**	**ACC**	**TTC**	**ACT**	**GAC**	**TTT**	**ATC**	**AGA**	**CCA**	**AGA**	**ACT**	**GGT**	**TAT**	**CTA**	**GGG**	**ATT**
M30		ATC	AAT	GAC	ACC	AGC	TTT	GGG	TTT	CAA	CTT	GGT	GGT	TGG	TTT	ATT	ACC	TTC	ACT	GAC	TTT	ATC	AGA	CCA	AGA	ACT	GGT	TAT	CTA	GGG	ATT
M2282		ATC	AAT	GAC	ACC	AGC	TTT	GGG	TTT	CAA	CTT	GGT	GGT	TGG	TTT	ATT	ACC	TTC	ACT	GAC	TTT	ATC	AGA	CCA	AGA	ACT	GGT	TAT	CTA	GGG	ATT
M2288		ATC	AAT	GAC	ACT	AGC	TTT	GGG	TTT	CAA	CTT	GGT	GGT	TGA	TTT	ATT	ACC	TTC	ACT	GAC	TTT	ATC	AGA	CCA	AGA	ACT	GGT	TAT	CTA	GGG	ATT
M2300		ATC	AAT	GAC	ACC	AGC	TTT	GGG	TTT	CAA	CTT	GGT	GGT	TGG	TTT	ATT	ACC	TTT	ACT	GAC	TTT	ATC	AGA	CCA	AGA	ACT	GGT	TAT	CTA	GGG	ATT
M2321		ATC	AAT	GAC	ACC	AGC	TTT	GGG	TTT	CAA	CTT	GGT	GGT	TGG	TTT	ATT	ACC	TTC	ACT	GAC	TTT	ATC	AGA	CCA	AGA	ACT	GGT	TAT	CTA	GGG	ATT
M2341		ATC	AAT	GAC	ACC	AGC	TTT	GGG	TTT	CAA	CTT	GGT	GGT	TGG	TTT	ATT	ACC	TTC	ACT	GAC	TTT	ATC	AGA	CCA	AGA	ACT	GGT	TAT	CTA	GGG	ATT
M6257		ATC	AAT	GAC	ACC	AGC	TTT	GGG	TTT	CAA	CTT	GGT	GGT	TGG	TTT	ATT	ACC	TTC	ACT	GAC	TTT	ATC	AGA	CCA	AGA	ACT	GGT	TAT	CTA	GGG	ATT
M6280		ATC	AAT	GAC	ACC	AGC	TTT	GGG	TTT	CAA	CTT	GGT	GGT	TGG	TTT	ATT	ACC	TTC	ACT	GAC	TTT	ATC	AGA	CCA	AGA	ACT	GGT	TAT	CTA	GGA	ATT
M6282		ATC	AAT	GAC	ACC	AGC	TTT	GGG	TTT	CAA	CTT	GGT	GGT	TGG	TTT	ATT	ACC	TTC	ACT	GAC	TTT	ATC	AGA	CCA	AGA	ACT	GGT	TAT	CTA	GGA	ATT
M6283		ATC	AAT	GAC	ACT	AGC	TTT	GGG	TTT	CAA	CTT	GGT	GGT	TGA	TTT	ATT	ACC	TTC	ACT	GAC	TTT	ATC	AGA	CCA	AGA	ACT	GGT	TAT	CTA	GGG	ATT
M6284		ATC	AAT	GAC	ACC	AGC	TTT	GGG	TTT	CAA	CTT	GGT	GGT	TGG	TTT	ATT	ACC	TTC	ACT	GAC	TTT	ATC	AGA	CCA	AGA	ACT	GGT	TAT	CTA	GGG	ATT
M6285		ATC	AAT	GAC	ACC	AGC	TTT	GGG	TTT	CAA	CTT	GGT	GGT	TGG	TTT	ATT	ACC	TTC	ACT	GAC	TTT	ATC	AGA	CCA	AGA	ACT	GGT	TAT	CTA	GGG	ATT
6286		ATC	AAT	GAC	ACT	AGC	TTT	GGG	TTT	CAA	CTT	GGT	GGT	TGA	TTT	ATT	ACC	TTC	ACT	GAC	TTT	ATC	AGA	CCA	AGA	ACT	GGT	TAT	CTA	GGG	ATT
M6320		ATC	AAT	GAC	ACC	AGC	TTT	GGG	TTT	CAA	CTT	GGT	GGT	TGG	TTT	ATT	ACC	TTC	ACT	GAC	TTT	ATC	AGA	CCA	AGA	ACT	GGT	TAT	CTA	GGG	ATT
64.0		ATC	AAT	GAC	ACC	AGC	TTT	GGG	TTT	CAA	CTT	GGT	GGT	TGG	TTT	ATT	ACC	TTC	ACT	GAC	TTT	ATC	AGA	CCA	AGA	ACT	GGT	TAT	CTA	GGA	ATT
64.1		ATC	AAT	GAC	ACC	AGC	TTT	GGG	TTT	CAA	CTT	GGT	GGT	TGG	TTT	ATT	ACC	TTC	ACT	GAC	TTT	ATC	AGA	CCA	AGA	ACT	GGT	TAT	CTA	GGA	ATT
199.0		ATC	AAT	GAC	ACT	AGC	TTT	GGG	TTT	CAA	CTT	GGT	GGT	TGG	TTT	ATT	ACC	TTC	ACT	GAC	TTT	ATC	AGA	CCA	AGA	ACT	GGT	TAT	CTA	GGG	ATT
199.1		ATC	AAT	GAC	ACT	AGC	TTT	GGG	TTT	CAA	CTT	GGT	GGT	TGG	TTT	ATT	ACC	TTC	ACT	GAC	TTT	ATC	AGA	CCA	AGA	ACT	GGT	TAT	CTA	GGG	ATT
MEGA10366		ATC	AAT	GAC	ACT	AGC	TTT	GGG	TTT	CAA	CTT	GGT	GGT	TGA	TTT	ATT	ACC	TTC	ACT	GAC	TTT	ATC	AGA	CCA	AGA	ACT	GGT	TAT	CTA	GGG	ATT
MEGA10378		ATC	AAT	GAC	ACC	AGC	TTT	GGG	TTT	CAA	CTT	GGT	GGT	TGG	TTT	ATT	ACC	TTC	ACT	GAC	TTT	ATC	AGA	CCA	AGA	ACT	GGT	TAT	CTA	GGG	ATT
MEGA10467		ATC	AAT	GAC	ACC	AGC	TTT	GGG	TTT	CAA	CTT	GGT	GGT	TGG	TTT	ATT	ACC	TTC	ACT	GAC	TTT	ATC	AGA	TCA	AGA	ACT	GGT	TAT	CTA	GGG	ATT
MEGA10477		ATC	AAT	GAC	ACT	AGC	TTT	GGG	TTT	CAA	CTT	GGT	GGT	TGA	TTT	ATT	ACC	TTC	ACT	GAC	TTT	ATC	AGA	CCA	AGA	ACT	GGT	TAT	CTA	GGG	ATT
Sea1		ATC	AAT	GAC	ACC	AGC	TTT	GGG	TTT	CAA	CTT	GGT	GGT	TGG	TTT	ATT	ACC	TTC	ACT	GAC	TTT	ATC	AGA	CCA	AGA	ACT	GGT	TAT	CTA	GGA	ATT
Sea2		ATC	AAT	GAC	ACC	AGC	TTT	GGG	TTT	CAA	CTT	GGT	GGT	TGG	TTT	ATT	ACC	TTC	ACT	GAC	TTT	ATC	AGA	CCA	AGA	ACT	GGT	TAT	CTA	GGG	ATT
		***	***	***	**	***	***	***	***	***	***	***	***	**	***	***	***	**	***	***	***	***	***	**	***	***	***	***	***	**	***
**G37**	**1,015**	**I**	**N**	**D**	**T**	**S**	**F**	**G**	**F**	**Q**	**L**	**G**	**G**	**W**	**F**	**I**	**T**	**F**	**T**	**D**	**F**	**I**	**R**	**P**	**R**	**T**	**G**	**Y**	**L**	**G**	**I**
																								**S**							

**Consensus**	**1,017**	I	N	D	T	S	F	G	F	Q	L	G	G	W	F	I	T	F	T	D	F	I	R	P	R	T	G	Y	L	G	I

```
G37         3,133  ACC TTA AGT AGC TTA CAA GAT CAA ACC ATT ATC TGA GCA GAT CAG CCT TGA ACT AGT TTC AAA GGC AGT TAT CTA GAC AGT GAT GGT ACC
M30                ACC TTA AGT AGC TTA CAA GAT CAA ACC ATT ATC TGA GCA GAT CAG CCT TGA ACT AGT TTC AAA GGC AGT TAT CTA GAC AGT GAT GGT ACC
M2282              ACC TTA AGT AGC TTA CAA GAT CAA ACC ATT ATC TGA GCA GAT CAG CCT TGA ACT AGT TTC AAA GGC AGT TAT CTA GAC AGT GAT GGT ACC
M2288              ACC TTA AGT AGC TTA CAA GAT CAA AAC ATT ATC TGA GCA GAT CAA CCT TGA ACT AGT TTC AAA GGG AGT TAT CTA GAC AGT GAT GGT ACC
M2300              ACC TTA AGT AGC TTA CAA GAT CAA ACC ATT ATC TGA GCA GAT CAG CCT TGA ACT AGT TTC AAA GGC AGT TAT CTA GAC AGT GAT GGT ACC
M2321              ACC CTA AGT AGC TTA CAA GAT CAA ACC ATT ATC TGA GCA GAT CAA CCT TGA ACT AGT TTC AAA GGC AGT TAT CTA GAC AGT GAT GGT ACC
M2341              ACC TTA AGT AGC TTA CAA GAT CAA ACC ATT ATC TGA GCA GAT CAG CCT TGA ACT AGT TTC AAA GGC AGT TAT CTA GAC AGT GAT GGT ACC
M6257              ACC CTA AGT AGC TTA CAA GAT CAA ACC ATT ATC TGA GCA GAT CAA CCT TGA ACT AGT TTC AAA GGC AGT TAT CTA GAC AGT GAT GGT ACC
M6280              ACC TTA AGT AGC TTA CAA GAT CAA AAC ATT ATC TGA GCA GAT CAG CCT TGA ACT AGT TTC AAA GGG AGT TAT CTA GAC AGT GAT GGT ACC
M6282              ACC TTA AGT AGC TTA CAA GAT CAA AAC ATT ATC TGA GCA GAT CAG CCT TGA ACT AGT TTC AAA GGG AGT TAT CTA GAC AGT GAT GGT ACC
M6283              ACC TTA AGT AGC TTA CAA GAT CAA AAC ATT ATC TGA GCA GAT CAA CCT TGA ACT AGT TTC AAA GGG AGT TAT CTA GAC AGT GAT GGT ACC
M6284              ACC TTA AGT AGC TTA CAA GAT CAA ACC ATT ATC TGA GCA GAT CAG CCT TGA ACT AGT TTC AAA GGC AGT TAT CTA GAC AGT GAT GGT ACC
M6285              ACC CTA AGT AGC TTA CAA GAT CAA ACC ATT ATC TGA GCA GAT CAA CCT TGA ACT AGT TTC AAA GGC AGT TAT CTA GAC AGT GAT GGT ACC
6286               ACC TTA AGT AGC TTA CAA GAT CAA AAC ATT ATC TGA GCA GAT CAA CCT TGA ACT AGT TTC AAA GGG AGT TAT CTA GAC AGT GAT GGT ACC
M6320              ACC TTA AGT AGC TTA CAA GAT CAA ACC ATT ATC TGA GCA GAT CAG CCT TGA ACT AGT TTC AAA GGC AGT TAT CTA GAC AGT GAT GGT ACC
64.0               ACC TTA AGT AGC TTA CAA GAT CAA AAC ATT ATC TGA GCA GAT CAG CCT TGA ACT AGT TTC AAA GGG AGT TAT CTA GAC AGT GAT GGT ACC
64.1               ACC TTA AGT AGC TTA CAA GAT CAA AAC ATT ATC TGA GCA GAT CAG CCT TGA ACT AGT TTC AAA GGG AGT TAT CTA GAC AGT GAT GGT ACC
199.0              ACC TTA AGT AGC TTA CAA GAT CAA AAC ATT ATC TGA GCA GAT CAG CCT TGA ACT AGT TTC AAA GGG AGT TAT CTA GAC AGT GAT GGT ACC
199.1              ACC TTA AGT AGC TTA CAA GAT CAA AAC ATT ATC TGA GCA GAT CAG CCT TGA ACT AGT TTC AAA GGG AGT TAT CTA GAC AGT GAT GGT ACC
MEGA10366          ACC TTA AGT AGC TTA CAA GAT CAA AAC ATT ATC TGA GCA GAT CAA CCT TGA ACT AGT TTC AAA GGG AGT TAT CTA GAC AGT GAT GGT ACC
MEGA10378          ACC TTA AGT AGC TTA CAA GAT CAA ACC ATT ATC TGA GCA GAT CAG CCT TGA ACT AGT TTC AAA GGC AGT TAT CTA GAC AGT GAT GGT ACC
MEGA10467          ACC TTA AGT AGC TTA CAA GAT CAA ACC ATT ATC TGA GCA GAT CAG CCT TGA ACT AGT TTC AAA GGC AGT TAT CTA GAC AGT GAT GGT ACC
MEGA10477          ACC TTA AGT AGC TTA CAA GAT CAA AAC ATT ATC TGA GCA GAT CAA CCT TGA ACT AGT TTC AAA GGG AGT TAT CTA GAC AGT GAT GGT ACC
Sea1               ACC TTA AGT AGC TTA CAA GAT CAA AAC ATT ATC TGA GCA GAT CAG CCT TGA ACT AGT TTC AAA GGG AGT TAT CTA GAC AGT GAT GGT ACC
Sea2               ACC CTA AGT AGC TTA CAA GAT CAA ACC ATT ATC TGA GCA GAT CAA CCT TGA ACT AGT TTC AAA GGC AGT TAT CTA GAC AGT GAT GGT ACC
                   ***  ** *** *** *** *** *** *** * * *** *** *** *** *** **  *** *** *** *** *** *** **  *** *** *** *** *** *** *** ***
G37         1,045   T   L   S   S   L   Q   D   Q   T   I   I   W   A   D   Q   P   W   T   S   F   K   G   S   Y   L   D   S   D   G   T
                                                    N

Consensus   1,047   T   L   S   S   L   Q   D   Q   T   I   I   W   A   D   Q   P   W   T   S   F   K   G   S   Y   L   D   S   D   G   T
```

**G37**	**3,223**	**CCT**	**AAA**	**TCA**	**CTG**	**TGA**	**GAT**	**CCA**	**ACT**	**GCT**	**TTA**	**AAA**	**TCC**	**CTT**	**CCA**	**AAT**	**AGT**	**TCA**	**ACT**	**ACC**	**TAT**	**---**	**GAT**	**ACC**	**AAT**	**CCT**	**ACC**	**CTC**	**TCA**	**CCC**
M30		CCT	AAA	TCA	CTG	TGA	GAT	CCA	ACT	GCT	TTA	AAA	TCC	CTT	CCA	AAT	AGT	TCA	ACT	ATC	TAT	---	GAT	ACC	AAT	CCT	ACC	CTC	TCA	CCC
M2282		CCT	AAA	TCA	CTG	TGA	GAT	CCA	ACT	GCT	TTA	AAA	TCC	CTT	CCA	AAT	AGT	TCA	ACT	ACC	TAT	---	GAT	ACC	AAT	CCT	ACC	CTC	TCA	CCC
M2288		CCT	AAA	TCA	CTG	TGA	GAT	CCA	ACT	GCT	TTA	AAA	CAA	CTA	CCA	ACT	AGT	TCA	ACT	ATC	TCT	---	GAT	ACC	AAT	CCT	ACC	CTC	CCC	CCC
M2300		CCT	AAA	TCA	CTG	TGA	GAT	CTA	ACT	GCT	TTA	AAA	TCC	CTT	CCA	AAT	AGT	TCA	ACT	ACC	TAT	---	GAT	ACC	AAT	CCT	ACC	CTC	TCA	CCC
M2321		CCT	AAA	TCA	CTG	TGA	GAT	CCA	ACT	GCT	TTA	AAA	TCC	TTA	TCA	ACT	ACT	TCT	---	---	AAT	---	GAT	AAT	TTT	CCT	ACC	CTC	TCC	CCC
M2341		CCT	AAA	TCA	CTG	TGA	GAT	CCA	ACT	GCT	TTA	AAA	TCC	CTT	CCA	AAT	AGT	TCA	ACT	ACC	TAT	---	GAT	ACC	AAT	CCT	ACC	CTC	TCA	CCC
M6257		CCT	AAA	TCA	CTG	TGA	GAT	CCA	ACT	GCT	TTA	AAA	TCC	TTA	TCA	ACT	ACT	TCT	---	---	AAT	---	GAT	AAT	TTT	CCT	ACC	CTC	TCC	CCC
M6280		CCT	AAA	TCA	CTG	TGA	GAT	CCA	ACT	GCT	TTA	AAA	TCC	CTT	CCA	AAT	AGT	TCA	ACT	ACC	TCT	---	GAT	ACC	AAT	CCT	ACC	CTC	TCA	CCC
M6282		CCT	AAA	TCA	CTG	TGA	GAT	CCA	ACT	GCT	TTA	AAA	TCC	CTT	CCA	AAT	AGT	TCA	ACT	ACC	TCT	---	GAT	ACC	AAT	CCT	ACC	CTC	TCA	CCC
M6283		CCT	AAA	TCA	CTG	TGA	GAT	CCA	ACT	GCT	TTA	AAA	CAA	CTA	CCA	ACT	AGT	TCA	ACT	ACC	TCT	---	GAT	ACC	AAT	CCT	ACC	CTC	TCC	CCC
M6284		CCT	AAA	TCA	CTG	TGA	GAT	CCA	ACT	GCT	TTA	AAA	TCC	CTT	CCA	AAT	AGT	TTA	ACT	ACC	TAT	---	GAT	ACC	AAT	CCT	ACC	CTC	TCA	CCC
M6285		CCT	AAA	TCA	CTG	TGA	GAT	CCA	ACT	GCT	TTA	AAA	TCC	TTA	TCA	ACT	ACT	TCT	---	---	AAT	---	GAT	AAT	TTT	CCT	ACC	CTC	TCC	CCC
6286		CCT	AAA	TCA	CTG	TGA	GAT	CCA	ACT	GCT	TTA	AAA	CAA	CTA	CCA	ACT	AGT	TCA	ACT	ACC	TCT	---	GAT	ACC	AAT	CCT	ACC	CTC	TCC	CCC
M6320		CCT	AAA	TCA	CTG	TGA	GAT	CCA	ACT	GCT	TTA	AAA	TCC	CTT	CCA	AAT	AGT	TCA	ACT	ACC	TAT	---	GAT	ACC	AAT	CCT	ACC	CTC	TCA	CCC
64.0		CCT	AAA	TCA	CTG	TGA	GAT	CCA	ACT	GCT	TTA	AAA	TCC	CTT	CCA	AAT	AGT	TCA	ACT	ACC	TCT	---	GAT	ACC	AAT	CCT	ACC	CTC	TCA	CCC
64.1		CCT	AAA	TCA	CTG	TGA	GAT	CCA	ACT	GCT	TTA	AAA	TCC	CTT	CCA	AAT	AGT	TCA	ACT	ACC	TCT	---	GAT	ACC	AAT	CCT	ACC	CTC	TCA	CCC
199.0		CCT	AAA	TCA	CTG	TGA	GAT	CCA	ACT	GCT	TTA	AGA	TCC	CTT	CCA	AAT	AGT	TCA	ACT	ACC	TCT	---	GAT	ATC	AAT	CCT	ACC	CTC	TCA	CCC
199.1		CCT	AAA	TCA	CTG	TGA	GAT	CCA	ACT	GCT	TTA	AGA	TCC	CTT	CCA	AAT	AGT	TCA	ACT	ACC	TCT	---	GAT	ATC	AAT	CCT	ACC	CTC	TCA	CCC
MEGA10366		CCT	AAA	TCA	CTG	TGA	GAT	CCA	ACT	GCT	TTA	AAA	CAA	CTA	CCA	ACT	AGT	TCA	ACT	ACC	TTT	---	GAT	ACC	AAT	CCT	ACC	CTC	TCC	CCC
MEGA10378		CCT	AAA	TCA	CTG	TGA	GAT	CCA	ACT	GCT	TTA	AAA	TCC	CTT	CCA	AAT	AGT	TCA	ACT	ACC	TAT	---	GAT	ACC	AAT	CCT	ACC	CTC	TCA	CCC
MEGA10467		CCT	AAA	TCA	CTG	TGA	GAT	CCA	ACT	GCT	TTA	AAA	TCC	CTT	CCA	AAT	AGT	TCA	ACT	ACC	TCT	---	GAT	ACC	AAT	CCT	ACC	CTC	TTA	CCC
MEGA10477		CCT	AAA	TCA	CTG	TGA	GAT	CCA	ACT	GCT	TTA	AAA	CAA	CTA	CCA	ACT	AGT	TCA	ACT	ACC	TCT	TCT	GAT	ACC	AAT	CCT	ACC	CTC	TCC	CCC
Sea1		CCT	AAA	TCA	CTG	TGA	GAT	CCA	ACT	GCT	TTA	AAA	TCC	CTT	CCA	AAT	AGT	TCA	ACT	ACC	TCT	---	GAT	ACC	AAT	CCT	ACC	CTC	TCA	CCC
Sea2		CCT	AAA	TCA	CTG	TGA	GAT	CCA	ACT	GCT	TTA	AAA	TCC	TTA	TCA	ACT	ACT	TCT	---	---	AAT	---	GAT	AAT	TTT	CCT	ACC	CTC	TCC	CCC
		***	***	***	***	***	***	* *	***	***	***	* *		*	***	**	**	*			*		***	*	*	***	***	***		***
**G37**	**1,075**	**P**	**K**	**S**	**L**	**W**	**D**	**P**	**T**	**A**	**L**	**K**	**S**	**L**	**P**	**N**	**S**	**S**	**T**	**T**	**Y**	**-**	**D**	**T**	**N**	**P**	**T**	**L**	**S**	**P**
								**L**				**R**	**Q**		**S**	**T**	**T**	**L**	**-**	**-**	**S**	**S**		**N**	**F**				**P**	
																				**I**	**N**			**I**					**L**	
																					**F**									
**Consensus**	**1,077**	**P**	**K**	**S**	**L**	**W**	**D**	**P**	**T**	**A**	**L**	**K**	**S**	**L**	**P**	**N**	**S**	**S**	**T**	**T**	**S**	**-**	**D**	**T**	**N**	**P**	**T**	**L**	**S**	**P**

Repeat Region G (bp 3,307-3,549, aa 1,103-1,183)
33 Sequences Analyzed

**G37**	**3,307**	**TCC**	**TTC**	**CAA**	**CTC**	**TAC**	**---**	**CAA**	**CCC**	**AAC**	**AAG**	**GTG**	**AAG**	**---**	**---**	**GCT**	**TAC**	**CAA**	**ACC**	**ACT**	**AAC**	**ACC**	**TAC**	**AAC**	**AAG**	**TTA**	**ATT**	**GAA**	**CCA**	**GTT**	**---**
G37Sv3.2		TCC	TTC	CAA	CTC	TAC	---	CAA	CCC	AAC	AAG	GTG	AAG	---	---	GCT	TAC	CAA	ACC	ACT	AAC	ACC	TAC	AAC	AGG	TTA	ATT	GAA	CCT	GAC	AAG
M30		TCC	TTC	CAA	CTC	TAC	---	CAA	CCC	AAC	AAG	GTG	AAA	AGT	GGT	CAA	TAC	CAA	ACC	ACT	AAC	ACC	TAC	AAC	AGG	TTA	ATT	GAA	CCG	GAA	---
M2282		TCC	TTC	---	CTC	TAC	TTT	CAA	CCC	AAC	AAG	GTG	AAA	AGT	GGT	CAA	TAT	CAA	CAA	AAT	AAC	ACC	TAC	AAC	AGG	TTA	ATT	GAA	CCT	GAC	AAG
M2288		TCC	TTC	---	CTC	TAC	TTT	CAA	CCC	AAC	AAG	GTG	AAA	AGT	GGT	CAA	TAC	CAA	ACC	ACC	AAC	ACC	TAC	AAC	AAG	TTA	ATT	GAA	CCT	GAC	AAG
M2300		TCC	TTC	CAA	CTC	TAC	---	CAA	CCC	AAC	AAG	GTG	AAG	---	---	GCT	TAC	CAA	ACC	ACT	AAC	ACC	TAC	AAC	AAG	TTA	ATT	GAA	CCT	GAC	AAG
M2321		TCC	TTC	---	CTC	TAC	TTT	CAA	CCC	AAC	AAG	GTG	AAG	---	---	CAA	TAC	AAT	GCA	TCA	AAC	ACC	TAC	AAC	AAG	TTA	ATT	GAG	CCT	GAC	AAG
M2341		TCC	TTC	CAA	CTC	TAC	---	CAA	CCC	AAC	AAG	GTG	AAG	---	---	GCT	TAC	CAA	ACC	ACT	AAC	ACC	TAC	AAC	AGG	TTA	ATT	GAA	CCG	GAA	---
M6257		TCC	TTC	---	CTC	TAC	TTT	CAA	CCC	AAC	AAG	GTG	AAG	---	---	GCT	TAC	CAA	ACC	ACT	AAC	ACC	TAC	AAC	AGG	TTA	ATT	GAA	CCG	GAA	---
M6280		TCC	TTC	---	CTC	TAC	TTT	CAA	CCC	AAC	AAG	GTG	AAG	---	---	GCT	TAC	CAA	ACC	ACT	AAC	ACC	TAC	AAC	AGG	TTA	ATT	GAG	CCT	GAC	---
M6282		TCC	TTC	CAG	CTC	TAC	---	CAA	CCC	AAC	AAG	GTG	AAA	AGT	GGT	CAA	TAC	CAA	ACC	ACC	AAC	ACC	TAC	AAC	AAG	TTG	ATT	GAG	CCT	GAC	AAG
M6283		TCC	TTC	CAG	CTC	TAC	---	CAA	CCC	AAC	AAG	GTG	AAA	---	---	CAA	TAC	AAT	GCA	TCT	AAC	ACC	TAC	AAC	AAG	TTA	ATT	GAG	CCT	GAC	---
M6284		TCC	TTC	CAA	CTC	TAC	---	CAA	CCC	AAC	AAG	GTG	AAA	AGT	GGT	CAA	TAT	CAA	CAA	AAT	AAC	ACC	TAC	AAC	AAG	TTA	ATT	GAA	CCA	GTT	---
M6285		TCC	TTC	---	CTC	TAC	TTT	CAA	CCC	AAC	AAG	GTG	AAG	---	---	CAA	TAC	AAT	GCA	TCC	AAC	ACC	TAC	CAC	AGG	TTA	ATT	GAA	CCT	GAC	AAG
6286		TCC	TTC	---	CTC	TAC	TTT	CAA	CCC	AAC	AAG	GTG	AAA	ACA	AAT	GCT	TAC	CAA	ACC	ACT	AAC	ACC	TAC	AAC	AAG	TTA	ATT	GAA	CCG	GAG	CAG
M6320		TCC	TTC	---	CTC	TAC	TTT	CAA	CCC	AAC	AAG	GTG	AAA	AGT	GGT	CAA	TAT	CAA	CAA	AAT	AAC	ACC	TAC	AAC	AAG	TTA	ATT	GAA	CCT	GAC	---
64.0		TCC	TTC	---	CTC	TAC	TTT	CAA	CCC	AAC	AAG	GTG	AAA	ACA	AAT	GCT	TAC	CAA	ACC	ACT	AAC	ACC	TAC	AAC	AAG	TTA	ATT	GAA	CCT	GAC	AAG
64.1		TCC	TTC	---	CTC	TAC	TTT	CAA	CCC	AAC	AAG	GTG	AAA	ACA	AAT	GCT	TAC	CAA	ACC	ACT	AAC	ACC	TAC	AAC	AAG	TTA	ATT	GAA	CCT	GAC	AAG
199.0		TCC	TTC	---	CTC	TAC	TTT	CAA	CCC	AAC	AAG	GTG	AAG	---	---	GCT	TAC	CAA	ACC	ACT	AAC	ACC	TAC	AAC	AAG	TTA	ATT	GAA	CCG	GAG	CAG
199.1		TCC	TTC	---	CTC	TAC	TTT	CAA	CCC	AAC	AAG	GTG	AAA	ACA	AAT	GCT	TAC	CAA	ACC	ACT	AAC	ACC	TAC	AAC	AAG	TTA	ATT	GAA	CCG	GAG	CAG
199.1b		TCC	TTC	---	CTC	TAC	TTT	CAA	CCC	AAC	AAG	GTG	AAA	ACA	AAT	GCT	TAC	CAA	ACC	ACT	AAC	ACC	TAC	AAC	AAG	TTA	ATT	GAA	CCG	GAG	CAG
MEGA10400.a		TCC	TTC	CAA	CTC	TAC	---	CAA	CCC	AAC	AAG	GTG	AAA	AGT	GGT	CAA	TAC	CAA	ACC	ACT	AAC	ACC	TAC	AAC	AGG	TTA	ATT	GAA	CCT	GAC	AAG
MEGA10400.b		TCC	TTC	CAA	CTC	TAC	---	CAA	CCC	AAC	AAG	GTG	AAA	AGT	GGT	CAA	TAC	CAA	ACC	ACT	AAC	ACC	TAC	AAC	AGG	TTA	ATT	GAA	CCT	GAC	AAG
KOR10081.a		TCC	TTC	---	CTC	TAC	TTT	CAA	CCC	AAC	AAG	GTG	AAA	---	---	GCT	TAC	AAT	GCA	TCC	AAC	ACC	TAC	CAC	AGG	TTA	ATT	GAG	CCT	GAC	AAG
KOR10081.b		TCC	TTC	---	CTC	TAC	TTT	CAA	CCC	AAC	AAG	GTG	AAA	---	---	GCT	TAC	AAT	GCA	TCC	AAC	ACC	TAC	CAC	AGG	TTA	ATT	GAG	CCT	GAC	AAG
KOR10081.c		TCC	TTC	---	CTC	TAC	TTT	CAA	CCC	AAC	AAG	GTG	AAA	---	---	GCT	TAC	AAT	GCA	TCC	AAC	ACC	TAC	CAC	AGG	TTA	ATT	GAG	CCT	GAC	AAG
KOR10081.d		TCC	TTC	---	CTC	TAC	TTT	CAA	CCC	AAC	AAG	GTG	AAA	---	---	GCT	TAC	AAT	GCA	TCC	AAC	ACC	TAC	CAC	AGG	TTA	ATT	GAG	CCT	GAC	AAG
KOR10081.e		TCC	TTC	---	CTC	TAC	TTT	CAA	CCC	AAC	AAG	GTG	AAA	---	---	CAA	TAC	AAT	GCA	TCC	AAC	ACC	TAC	CAC	AGG	TTA	ATT	GAG	CCT	GAC	AAG
KOR10081.g		TCC	TTC	---	CTC	TAC	TTT	CAA	CCC	AAC	AAG	GTG	AAA	---	---	CAA	TAC	AAT	GCA	TCC	AAC	ACC	TAC	CAC	AGG	TTA	ATT	GAG	CCT	GAC	AAG
KOR10081.h		TCC	TTC	---	CTC	TAC	TTT	CAA	CCC	AAC	AAG	GTG	AAA	---	---	CAA	TAC	AAT	GCA	TCC	AAC	ACC	TAC	CAC	AGG	TTA	ATT	GAG	CCT	GAC	AAG
KOR10081.i		TCC	TTC	---	CTC	TAC	TTT	CAA	CCC	AAC	AAG	GTG	AAA	---	---	GCT	TAC	AAT	GCA	TCC	AAC	ACC	TAC	CAC	AGG	TTA	ATT	GAG	CCT	GAC	AAG
Sea1		TCC	TTC	---	CTC	TAC	TTT	CAA	CCC	AAC	AAG	GTG	AAA	ACA	AAT	GCT	TAC	CAA	AGC	ACT	AAC	ACC	TAC	AAC	AGG	TTA	ATT	GAA	CCA	GTT	---
Sea2		TCC	TTC	CAA	CTC	TAC	---	CAA	CCC	AAC	AAG	GTG	AAA	AGT	GGT	CAA	TAT	CAA	CAA	AAT	AAC	ACC	TAC	AAC	AAG	TTA	ATT	GAA	CCG	GAA	---
		***	***		***	***		***	***	***	***	***					**	*			***	***	***	**	* *	**	***	**	**	*	
**G37**	**1,103**	**S**	**F**	**Q**	**L**	**Y**	**-**	**Q**	**P**	**N**	**K**	**V**	**K**	**-**	**-**	**A**	**Y**	**Q**	**T**	**T**	**N**	**T**	**Y**	**N**	**K**	**L**	**I**	**E**	**P**	**V**	**-**
				**-**			**F**							**S**	**G**	**Q**		**N**	**Q**	**N**				**H**	**R**					**D**	**K**
														**T**	**N**				**A**	**S**										**E**	**Q**
																			**S**												
**Consensus**	**1,105**	**S**	**F**	**-**	**L**	**Y**	**F**	**Q**	**P**	**N**	**K**	**V**	**K**	**-**	**-**	**A**	**Y**	**Q**	**T**	**T**	**N**	**T**	**Y**	**N**	**R**	**L**	**I**	**E**	**P**	**D**	**K**

Variable Region G (bp 3,307-3,549; aa 1,103-1,183)

**G37**	**3,385**	**---**	**GAT**	**GCA**	**ACA**	**AGT**	**GCA**	**GCA**	**ACT**	**AAC**	**ATG**	**ACC**	**AGT**	**TTG**	**TTA**	**AAA**	**CTC**	**CTA**	**ACA**	**ACT**	**AAA**	**AAC**	**ATC**	**AAA**	**GCG**	**AAA**	**TTG**	**GGG**	**AAG**	**---**	**GGA**
G37Sv3.2		TGA	CAA	TCA	AGT	AGT	GAT	TTG	AAC	AAT	ATG	ACC	AAC	TTG	TTA	AAA	CTC	CTA	ACA	ACT	AAA	AAC	ATC	AAA	GCG	AAA	TTG	GGG	AAG	---	GAC
M30		---	AGT	GCA	ACA	AGT	GCA	GCA	ACT	AAC	ATG	ACC	AGT	TTG	TTA	AAC	ATG	TTG	TCT	AGT	AAA	AAC	ATC	AAA	CAA	AAG	TTG	GGG	AAG	---	GGA
M2282		TGA	CAA	TCA	AGT	AGT	GAT	TTG	AAC	AAT	ATG	ACC	AGT	TTG	TTA	AAG	CTG	TTG	TCT	AGT	AAA	AAC	ATC	AAA	CAG	AAG	TTG	GGG	AAG	---	GAC
M2288		TGA	CAA	TCA	AGT	AGT	GAT	TTG	AGT	GGG	ATG	AAT	AAC	TTG	TTA	AAA	CTA	CTG	ACA	ACT	AAA	AAT	GTG	AAA	GCA	AAG	TTG	GGG	AAG	GGA	GCA
M2300		TGA	CAA	TCA	AAT	AGT	GAT	TTG	ACT	AAC	ATG	ACC	AGT	TTG	TTA	AAC	ATG	TTG	TCT	AGT	AAA	AAC	ATC	AAA	CAA	AAG	TTG	GGG	AAG	---	GAC
M2321		TGA	CAA	TCA	AGT	AGT	GAT	TTG	ACT	AAC	ATG	ACC	AGT	TTG	TTA	AAA	CTC	CTA	ACA	ATT	AAA	AAC	ATC	AAA	CAA	AAG	TTG	GGG	AAG	---	---
M2341		---	AGT	GCA	ACA	AGT	GCA	GCA	ACT	AAC	ATG	ACC	AGC	TTG	TTA	AAG	CTG	TTG	TCT	AGT	AAA	AAC	ATC	AAA	CAA	AAG	TTG	GGG	AAG	GGG	GGA
M6257		---	AGT	GCA	ACA	AGT	GCA	GCA	ACT	AAC	ATG	ACT	AGT	TTG	TTA	AAA	CTC	CTA	ACA	ACT	AAA	AAC	ATC	AAA	CAA	AAG	TTG	GGG	AAG	---	GGA
M6280		---	AAT	GCA	ACA	AGT	GCA	GCA	ACT	AAC	ATG	ACT	AGT	TTG	TTA	AAA	CTC	CTA	ACA	ACT	AAA	AAC	ATC	AAA	CAA	AAG	TTG	GGG	AAG	---	---
M6282		TGA	GAA	TCA	TCA	AGT	GAT	TTG	ACT	AAC	ATG	ACC	AAC	TTG	TTA	AAG	CTG	TTG	TCT	AGT	AAA	AAC	ATC	AAA	GAA	AAG	TTG	GGG	AAG	---	GAC
M6283		---	AAT	GCA	ACA	AGT	GCA	GCA	ACT	AAC	ATG	ACC	AGC	TTG	TTA	AAC	CTG	TTG	TCT	AGT	AAA	AAC	ATC	AAA	CAG	AAG	TTG	GGG	AAG	---	GAC
M6284		---	GAT	GCA	ACA	AGT	GCA	GCG	AGC	AGC	ATG	ACC	AAC	TTG	TTA	AAG	CTG	TTG	TCT	AGT	AAA	AAC	ATC	AAA	CAA	AAG	TTG	GGG	AAG	---	---
M6285		TGA	CAA	TCA	AAT	AGT	GAT	TTG	AGT	GGG	ATG	AAT	AAC	TTG	TTA	AAA	CTC	CTA	ACA	ATT	AAA	AAC	ATC	AAA	CAA	AAG	TTG	GGG	AAG	---	---
6286		TGA	AAC	CAA	GCA	AGT	GAT	TTG	AGT	GGG	ATG	AAT	AAC	TTG	TTA	AAG	CTG	TTG	TCT	AGT	AAA	AAC	ATC	AAA	CAA	AAG	TTG	GGG	AAG	---	GGA
M6320		---	AAT	GCA	ACA	AGT	GCA	GCA	ACT	AAC	ATG	ACT	AAC	TTG	TTA	AAG	CTG	TTG	TCT	AGT	AAA	AAC	ATC	AAA	CAA	AAA	TTG	GGG	AAG	---	GAC
64.0		TGA	CAA	TCA	AGT	AGT	GAT	TTG	AAC	AAT	ATG	ACC	AAC	TTG	TTA	AAA	CTA	CTG	ACA	ACT	AAA	AAT	GTG	AAA	GCA	AAG	TTG	GGG	AAG	---	GAC
64.1		TGA	CAA	TCA	AGT	AGT	GAT	TTG	AAC	AAT	ATG	ACC	AAC	TTG	TTA	AAA	CTA	CTG	ACA	ACT	AAA	AAT	GTG	AAA	GCA	AAG	TTG	GGG	AAG	---	GAC
199.0		TGA	AAC	CAA	GCA	AGT	GAT	TTG	AGT	GGG	ATG	AAT	AAC	TTG	TTA	AAA	CTA	CTA	ACA	ACT	AAA	AAT	GTG	AAA	GCA	AAG	TTG	GGG	AAG	---	GAC
199.1		TGA	AAC	CAA	GCA	AGT	GAT	TTG	AGT	GGG	ATG	AAT	AAC	TTG	TTA	AAA	CTA	CTA	ACA	ACT	AAA	AAT	GTG	AAA	GCA	AAG	TTG	GGG	AAG	---	GAC
199.1b		TGA	AAC	CAA	GCA	AGT	GAT	TTG	AGT	GGG	ATG	AAT	AAC	TTG	TTA	AAA	CTA	CTA	ACA	ACT	AAA	AAT	GTG	AAA	GCA	AAG	TTG	GGG	AAG	---	GAC
MEGA10400.a		TGA	CAA	TCA	AGT	AGT	GAT	TTG	ACT	AAC	ATG	ACC	AGT	TTG	TTA	AAG	CTA	CTG	ACA	ACT	AAA	AAC	ATC	AAA	CAG	AAG	TTG	GGG	AAG	---	GAC
MEGA10400.b		TGA	CAA	TCA	AGT	AGT	GAT	TTG	ACT	AAC	ATG	ACC	AGT	TTG	TTA	AAA	CTA	CTG	ACA	ACT	AAA	AAC	ATC	AAA	CAA	AAG	TTG	GGG	AAG	---	GAC
KOR10081.a		TGA	AAC	CAA	GCA	AGT	GAT	TTG	AGT	GGG	ATG	AAT	AAC	TTG	TTA	AAC	ATG	TTG	TCT	AGT	AAA	AAC	ATC	AAA	CAA	AAG	TTG	GGG	AAG	---	---
KOR10081.b		TGA	AAC	CAA	GCA	AGT	GAT	TTG	AGT	GGG	ATG	AAT	AAC	TTG	TTA	AAC	ATG	TTG	TCT	AGT	AAA	AAC	ATC	AAA	CAA	AAG	TTG	GGG	AAG	---	---
KOR10081.c		TGA	AAC	CAA	GCA	AGT	GAT	TTG	AGT	GGG	ATG	AAT	AAC	TTG	TTA	AAG	CTC	CTA	ACA	ACT	AAA	AAC	ATC	AAA	CAA	AAG	TTG	GGG	AAG	---	---
KOR10081.d		TGA	AAC	CAA	GCA	AGT	GAT	TTG	AGT	GGG	ATG	AAT	AAC	TTG	TTA	AAG	CTG	TTG	TCT	AGT	AAA	AAC	ATC	AAA	CAA	AAG	TTG	GGG	AAG	---	---
KOR10081.e		TGA	CAA	TCA	AAT	AGT	GAT	TTG	AAC	AAT	ATG	ACC	AAC	TTG	TTA	AAG	CTC	CTA	ACA	ACT	AAA	AAC	ATC	AAA	CAA	AAG	TTG	GGG	AAG	---	---
KOR10081.g		TGA	CAA	TCA	AAT	AGT	GAT	TTG	AAC	AAT	ATG	ACC	AAC	TTG	TTA	AAG	CTC	CTA	ACA	ACT	AAA	AAC	ATC	AAA	CAA	AAG	TTG	GGG	AAG	---	---
KOR10081.h		TGA	AAC	CAA	GCA	AGT	GAT	TTG	AGT	GGG	ATG	AAT	AAC	TTG	TTA	AAC	ATG	TTG	TCT	AGT	AAA	AAC	ATC	AAA	CAA	AAG	TTG	GGG	AAG	---	GGA
KOR10081.i		TGA	AAC	CAA	GCA	AGT	GAT	TTG	AGT	GGG	ATG	AAT	AAC	TTG	TTA	AAC	ATG	TTG	TCT	AGT	AAA	AAC	ATC	AAA	CAA	AAG	TTG	GGG	AAG	---	---
Sea1		---	GAT	GCA	ACA	AGT	GCA	GCA	ACT	AAC	ATG	ACC	AGC	TTG	TTA	AAA	CTC	CTA	ACA	ACT	AAA	AAT	GTG	AAA	GCA	AAG	TTG	GGG	AAG	---	GAC
Sea2		---	AGT	GCA	ACA	AGT	GCA	GCA	ACT	AAC	ATG	ACC	AGT	TTG	TTA	AAA	CTC	CTA	ACA	ATT	AAA	AAC	ATC	AAA	CAG	AAG	TTG	GGG	AAG	---	GAC
						***	*		*		***	*	*	***	***	**	*	*	*	* *	***	**	*	***		**	***	***	***		
**G37**	**1,129**	**-**	**D**	**A**	**T**	**S**	**A**	**A**	**T**	**N**	**M**	**T**	**S**	**L**	**L**	**K**	**L**	**L**	**T**	**T**	**K**	**N**	**I**	**K**	**A**	**K**	**L**	**G**	**K**	**-**	**G**
		W	Q	S	S		D	L	N	G		N	N			N	M		S	S			V		Q					G	-
			S	Q	N				S	S										I					E						D
			N		A																										A
			E																												
**Consensus**	**1,132**	**W**	**N**	**S**	**T**	**S**	**D**	**L**	**T**	**N**	**M**	**T**	**N**	**L**	**L**	**K**	**L**	**L**	**T**	**T**	**K**	**N**	**I**	**K**	**Q**	**K**	**L**	**G**	**K**	**-**	**D**

Variable Region G (bp 3,307-3,549; aa 1,103-1,183)

**G37**	**3,469**	**ACA**	**GCT**	---	**TCT**	**TCG**	**CAG**	**GGA**	**AAT**	**AAT**	**AAT**	**GGA**	**GGG**	**GGT**	**GTT**	**AGT**	**CAA**	**ACG**	**ATT**	**AAC**	**ACC**	**ATC**	**ACC**	**ACT**	**ACG**	**GGA**	**AAT**	**ATT**	**AGT**
G37Sv3.2		ACC	---	---	CAA	TCA	ATG	GGA	AAT	AAT	AAT	GGA	GGG	GGT	GTT	AGT	CAA	ACG	ATT	AAC	ACC	ATC	ACC	ACT	ACG	GGA	AAT	ATT	AGT
M30		ACA	GCA	---	---	ATG	CAG	GGA	AAT	AAT	GGT	GGA	GGG	GGT	GTT	AGT	CAA	ACG	ATT	AAC	ACC	ATC	ACC	ACT	ACG	GGA	AAT	ATT	AGT
M2282		ACC	---	---	CAA	TCA	ATG	GGA	AAT	AAT	AAT	GGA	GGG	GGT	GTT	AGT	CAA	ACG	ATT	AAC	ACC	ATC	ACC	ACT	ACG	GGA	AAT	ATT	AGT
M2288		---	GCT	---	TCT	TCG	CAG	GGA	---	---	AGT	GGG	GGG	GGT	GTT	AGT	CAA	ACG	ATT	AAT	ACC	ATC	GCT	ACT	ACG	GGG	AAT	ATT	AGT
M2300		ACC	---	---	CAA	TCA	ATG	GGA	AAT	AAT	AAT	GGA	GGG	GGT	GTT	AGT	CAA	ACG	ATT	AAC	ACC	ATC	ACC	ACT	ACG	GGA	AAT	ATT	AGT
M2321		ACA	GCC	---	CAA	TCT	CAG	GAA	AAT	---	AGT	GGG	GGG	GGT	GTT	AGT	CAA	ACG	ATT	AAC	ACC	ATT	ACC	ACT	ACG	GGA	AAT	ATT	AGT
M2341		ACA	GCA	---	---	ATG	CAG	GGA	AAT	AAT	GGT	GGA	GGG	GGT	GTT	AGT	CAA	ACG	ATT	AAC	ACC	ATT	ACC	ACT	ACG	GGG	AAT	ATT	AGT
M6257		ACA	GCT	---	TCT	TCG	CAG	GGA	AAT	AAT	AAT	GGA	GGG	GGT	GTT	AGT	CAA	ACG	ATT	AAC	ACC	ATC	ACC	ACT	ACG	GGA	AAT	ATT	AGT
M6280		ACA	GCC	---	CAA	TCT	CAG	GAA	AAT	---	AGT	GGG	GGG	GGT	GTT	AGT	CAA	ACG	ATT	AAT	ACC	ATC	ACC	ACT	ACG	GGA	AAT	ATT	AGT
M6282		ACC	---	---	CAA	TCA	ATG	GGA	AAT	---	AAT	GGA	GGG	GGT	GTT	AGT	CAA	ACG	ATT	AAC	ACC	ATT	ACC	ACT	ACG	GGA	AAT	ATT	AGT
M6283		ACC	---	---	CAA	TCA	ATG	GGA	AAT	AAT	GGT	GGA	GGG	GGT	GTT	AGT	CAA	ACG	ATT	AAC	ACC	ATC	GCT	ACT	ACG	GGG	AAT	ATT	AGT
M6284		ACA	GCC	---	CAA	TCT	CAG	GAA	AAT	---	AGT	GGA	GGG	GGT	GTT	AGT	CAA	ACG	ATT	AAC	ACC	ATT	ACC	ACT	ACG	GGA	AAT	ATT	AGT
M6285		ACA	GCC	---	CAA	TCT	CAG	GAA	AAT	---	AGT	GGG	GGG	GGT	GTT	AGT	CAA	ACG	ATT	AAC	ACC	ATC	ACC	ACT	ACG	GGA	AAT	ATT	AGT
6286		ACA	GCA	---	---	ATG	CAG	GGA	AAT	---	AAT	GGA	GGG	GGT	GTT	AGT	CAA	ACG	ATT	AAC	ACC	ATC	ACC	ACT	ACG	GGA	AAT	ATT	AGT
M6320		ACC	---	---	CAA	TCA	ATG	GGA	AAT	AAT	AAT	GGA	GGG	GGT	GTT	AGT	CAA	ACG	ATT	AAC	ACC	ATC	ACC	ACT	ACG	GGA	AAT	ATT	AGT
64.0		ACC	---	---	CAA	TCA	ATG	GGA	AAT	---	AAT	GGA	GGG	GGT	GTT	AGT	CAA	ACG	ATT	AAC	ACC	ATC	ACC	ACT	ACG	GGA	AAT	ATT	AGT
64.1		ACC	---	---	CAA	TCA	ATG	GGA	AAT	---	AAT	GGA	GGG	GGT	GTT	AGT	CAA	ACG	ATT	AAC	ACC	ATC	ACC	ACT	ACG	GGA	AAT	ATT	AGT
199.0		ACC	---	---	CAA	TCA	ATG	GGA	AAT	---	AAT	GGA	GGG	GGT	GTT	AGT	CAA	ACG	ATT	AAC	ACC	ATT	ACC	ACT	ACG	GGG	AAT	ATT	AGT
199.1		ACC	---	---	CAA	TCA	ATG	GGA	AAT	---	AAT	GGA	GGG	GGT	GTT	AGT	CAA	ACG	ATT	AAC	ACC	ATT	ACC	ACT	ACG	GGG	AAT	ATT	AGT
199.1b		ACC	---	---	CAA	TCA	ATG	GGA	AAT	---	AAT	GGA	GGG	GGT	GTT	AGT	CAA	ACG	ATT	AAC	ACC	ATT	ACC	ACT	ACG	GGG	AAT	ATT	AGT
MEGA10400.a		ACA	GCC	ACC	CAA	TCT	CAG	GAA	AAT	---	AGT	GGG	GGG	GGT	GTT	AGT	CAA	ACG	ATT	AAC	ACC	ATC	ACC	ACT	ACG	GGA	AAT	ATT	AGT
MEGA10400.b		ACA	GCC	ACC	CAA	TCA	ATG	GGA	AAT	AAT	AAT	GGA	GGG	GGT	GTT	AGT	CAA	ACG	ATT	AAC	ACC	ATC	ACC	ACT	ACG	GGA	AAT	ATT	AGT
KOR10081.a		---	---	---	CAA	TCT	CAG	GAA	AAT	---	AGT	GGG	GGG	GGT	GTT	AGT	CAA	ACG	ATT	AAC	ACC	ATC	ACC	ACT	ACG	GGA	AAT	ATT	AGT
KOR10081.b		---	GCC	---	CAA	TCT	CAG	GAA	AAT	---	AGT	GTG	GGG	GGT	GTT	AGT	CAA	ACG	ATT	AAC	ACC	ATC	ACC	ACT	ACG	GGA	AAT	ATT	AGT
KOR10081.c		ACA	GCC	GCC	CAA	TCT	CAG	GAA	AAT	---	AGT	GGG	GGG	GGT	GTT	AGT	CAA	ACG	ATT	AAC	ACC	ATC	ACC	ACT	ACG	GGA	AAT	ATT	AGT
KOR10081.d		ACA	GCC	---	CAA	TCT	CAG	GAA	AAT	---	AGT	GGG	GGG	GGT	GTT	AGT	CAA	ACG	ATT	AAC	ACC	ATC	ACC	ACT	ACG	GGA	AAT	ATT	AGT
KOR10081.e		ACA	GCC	---	CAA	TCT	CAG	GAA	AAT	---	AGT	GGG	GGG	GGT	GTT	AGT	CAA	ACG	ATT	AAC	ACC	ATC	ACC	ACT	ACG	GGA	AAT	ATT	AGT
KOR10081.g		ACA	GCC	GCC	CAA	TCT	CAG	GAA	AAT	---	AGT	GGG	GGG	GGT	GTT	AGT	CAA	ACG	ATT	AAC	ACC	ATC	ACC	ACT	ACG	GGA	AAT	ATT	AGT
KOR10081.h		ACA	GCA	---	---	ATG	CAG	GGA	---	---	AGT	GGG	GGG	GGT	GTT	AGT	CAA	ACG	ATT	AAC	ACC	ATC	ACC	ACT	ACG	GGA	AAT	ATT	AGT
KOR10081.i		ACA	GCC	---	CAA	TCT	CAG	GAA	---	AGT	AGT	GGG	GGG	GGT	GTT	AGT	CAA	ACG	ATT	AAC	ACC	ATC	ACC	ACT	ACG	GGA	AAT	ATT	AGT
Sea1		ACC	---	---	CAA	TCA	ATG	GGA	AAT	---	AAT	GGA	GGG	GGT	GTT	AGT	CAA	ACG	ATT	AAC	ACC	ATT	ACC	ACT	ACG	GGA	AAT	ATT	AGT
Sea2		ACC	---	---	AAA	TCA	ATG	GGA	AAT	AAT	AAT	GGA	GGG	GGT	GTT	AGT	CAA	ACG	ATT	AAC	ACC	ATC	ACC	ACT	ACG	GGA	AAT	ATT	AGT
							*	* *			*	*	***	***	***	***	***	***	***	**	***	**	*	***	***	**	***	***	***
**G37**	**1,157**	**T**	**A**	**-**	**S**	**S**	**Q**	**G**	**N**	**N**	**N**	**G**	**G**	**G**	**V**	**S**	**Q**	**T**	**I**	**N**	**T**	**I**	**T**	**T**	**T**	**G**	**N**	**I**	**S**
		**-**	**-**	**T**	**-**	**M**	**M**	**E**	**-**	**-**	**G**	**V**											**A**						
				**A**	**Q**					**S**	**S**																		
					**K**																								
**Consensus**	**1,161**	**T**	**A**	**-**	**Q**	**S**	**Q**	**G**	**N**	**-**	**N**	**G**	**G**	**G**	**V**	**S**	**Q**	**T**	**I**	**N**	**T**	**I**	**T**	**T**	**T**	**G**	**N**	**I**	**S**

Conserved C-terminus (bp 3,550-4,065 aa 1,184-1,444)
25 Sequences Analyzed

**G37**	**3,550**	**GAA**	**GGT**	**CTA**	**AAA**	**GAA**	**GAA**	**ACT**	**AGT**	**ATT**	**CAA**	**GCA**	**GAA**	**ACA**	**CTT**	**AAA**	**AAG**	**TTC**	**TTT**	**GAT**	**---**	**AGT**	**AAA**	**CAA**	**AAC**	**AAT**	**AAG**	**AGT**	**GAA**	**ATA**	**GGG**
M30		GAA	GGT	CTA	AAA	GAA	GAA	ACT	AGT	ATT	CAA	GCA	GAA	ACA	CTT	AAA	AAG	TTC	TTT	GAT	---	AGT	AAA	CAA	AAC	AAT	AAG	AGT	GAA	ATA	GGG
M2282		GAA	GGT	CTA	AAA	GAA	GAA	ACT	AGT	ATT	CAA	GCA	GAA	ACA	CTT	AAA	AAG	TTC	TTT	GAT	---	AGT	AAA	CAA	AAC	AAT	AAG	AGT	AAA	ATA	GGG
M2288		GAA	GGT	CTA	AAA	GAA	GAA	ACT	AGT	ATT	CAA	GCA	GAA	ACA	CTT	AAA	AAG	TTC	TTT	GAT	---	AGT	AAA	CAA	AAC	AAT	AAG	AGT	GAA	ATA	GGG
M2300		GAA	GGT	CTA	AAA	GAA	GAA	ACT	AGT	ATT	CAA	GCA	GAA	ACA	CTT	AAA	AAG	TTC	TTT	GAT	---	AGT	AAA	CAA	AAC	AAT	AAG	AGT	GAA	ATA	GGG
M2321		GAA	GGT	CTA	AAA	GAA	GAA	ACT	AGT	ATT	CAA	GCA	GAA	ACA	CTT	AAA	AAG	TTC	TTT	GAT	---	AGT	AAA	CAA	AAC	AAT	AAG	AGT	GAA	ATA	GGG
M2341		GAA	GGT	CTA	AAA	GAA	GAA	ACT	AGT	ATT	CAA	GCA	GAA	ACA	CTT	AAA	AAG	TTC	TTT	GAT	---	AGT	AAA	CAA	AAC	AAT	AAG	AGT	GAA	ATA	GGG
M6257		GAA	GGT	CTA	AAA	GAA	GAA	ACT	AGT	ATT	CAA	GCA	GAA	ACA	CTT	AAA	AAG	TTC	TTT	GAT	---	AGT	AAA	CAA	AAC	AAT	AAG	AGT	GAA	ATA	GGG
M6280		GAA	GGT	CTA	AAA	GAA	GAA	ACT	AGT	ATT	CAA	GCA	GAA	ACA	CTT	AAA	AAG	TTC	TTT	GAT	---	AGT	AAA	CAA	AAC	AAT	AAG	AGT	GAA	ATA	GGG
M6282		GAA	GAT	CTA	AAA	GAA	GAA	ACT	AGT	ATT	CAA	GCA	GAA	ACA	CTT	AAA	AAG	TTC	TTT	GAT	---	AGT	AAA	CAA	AAC	AAT	AAG	AGT	GAA	ATA	GGG
M6283		GAA	GGT	CTA	AAA	GAA	GAA	ACT	AGT	ATT	CAA	GCA	GAA	ACA	CTT	AAA	AAG	TTC	TTT	GAT	AAT	AGT	AAA	CAA	AAC	AAT	AAG	AAT	GAA	ATA	GGG
M6284		GAA	GGT	CTA	AAA	GAA	GAA	ACT	AGT	ATT	CAA	GCA	GAA	ACA	CTT	AAA	AAG	TTC	TTT	GAT	---	AGT	AAA	CAA	AAC	AAT	AAG	AGT	GAA	ATA	GGG
M6285		GAA	GGT	CTA	AAA	GAA	AAA	ACT	AGT	ATT	CAA	GCA	GAA	ACA	CTT	AAA	AAG	TTC	TTT	GAT	---	AGT	AAA	CAA	AAC	AAT	AAG	AGT	GAA	ATA	GGG
6286		GAA	GGT	CTA	AAA	GAA	GAA	AGT	AGT	ATT	CAA	GCA	GAA	ACA	CTT	AAA	AAG	TTC	TTT	GAT	---	GGT	AAA	CAA	AAC	AAT	AAG	AAT	GAA	ATA	GGG
M6320		GAA	GGT	CTA	AAA	GAA	GAA	ACT	AGT	ATT	CAA	GCA	GAA	ACA	CTT	AAA	AAG	TTC	TTT	GAT	---	AGT	AAA	CAA	AAC	AAT	AAG	AGT	GAA	ATA	GGG
64.0		GAA	GGT	CTA	AAA	GAA	GAA	ACT	AGT	ATT	CAA	GCA	GAA	ACA	CTT	AAA	AAG	TTC	TTT	GAT	---	AGT	AAA	CAA	AAC	AAT	AAG	AGT	GAA	ATA	GGG
64.1		GAA	GGT	CTA	AAA	GAA	GAA	ACT	AGT	ATT	CAA	GCA	GAA	ACA	CTT	AAA	AAG	TTC	TTT	GAT	---	AGT	AAA	CAA	AAC	AAT	AAG	AGT	GAA	ATA	GGG
199.0		GAA	GGT	CTA	AAA	GAA	GAA	ACT	AGT	ATT	CAA	GCA	GAA	ACA	CTT	AAA	AAG	TTC	TTT	GAT	---	AGT	AAA	CAA	AAC	AAT	AAG	AGT	GAA	ATA	GGG
199.1		GAA	GGT	CTA	AAA	GAA	GAA	ACT	AGT	ATT	CAA	GCA	GAA	ACA	CTT	AAA	AAG	TTC	TTT	GAT	---	AGT	AAA	CAA	AAC	AAT	AAG	AGT	GAA	ATA	GGG
MEGA10366		GAA	GGT	CTA	AAA	GAA	GAA	AGT	AGT	ATT	CAA	GCA	GAA	ACA	CTT	AAA	AAG	TTC	TTT	GAT	---	GGT	AAA	CAA	AAC	AAT	AAG	AAT	GAA	ATA	GGG
MEGA10378		GAA	GGT	CTA	AAA	GAA	GAA	ACT	AGT	ATT	CAA	GCA	GAA	ACA	CTT	AAA	AAG	TTC	TTT	GAT	---	AGT	AAA	CAA	AAC	AAT	AAG	AGT	GAA	ATA	GGG
MEGA10467		GAA	GGT	CTA	AAA	GAA	GAA	ACT	AGT	ATT	CAA	GCA	GAA	ACA	CTT	AAA	AAG	TTC	TTT	GAT	---	AGT	AAA	CAA	AAC	AAT	AAG	AGT	GAA	ATA	GGG
MEGA10477		GAA	GGT	CTA	AAA	GAA	GAA	ACT	AGT	ATT	CAA	GCA	GAA	ACA	CTT	AAA	AAG	TTC	TTT	GAT	---	AGT	AAA	CAA	AAC	AAT	AAG	AAT	GAA	ATA	GGG
Sea1		GAA	GGT	CTA	AAA	GAA	GAA	ACT	AGT	ATT	CAA	GCA	GAA	ACA	CTT	AAA	AAG	TTC	TTT	GAT	---	AGT	AAA	CAA	AAC	AAT	AAG	AGT	GAA	ATA	GGG
Sea2		GAA	GGT	CTA	AAA	GAA	GAA	ACT	AGT	ATT	CAA	GCA	GAA	ACA	CTT	AAA	AAG	TTC	TTT	GAT	---	AGT	AAA	CAA	AAC	AAT	AAG	AGT	GAA	ATA	GGG
		***	* *	***	***	***	**	* *	***	***	***	***	***	***	***	***	***	***	***	***		***	***	***	***	***	***	* *	**	***	***
**G37**	**1,184**	**E**	**G**	**L**	**K**	**E**	**E**	**T**	**S**	**I**	**Q**	**A**	**E**	**T**	**L**	**K**	**K**	**F**	**F**	**D**	**–**	**S**	**K**	**Q**	**N**	**N**	**K**	**S**	**E**	**I**	**G**
			**D**				**K**	**S**													**N**	**G**						**N**	**K**		
**Consensus**	**1,188**	**E**	**G**	**L**	**K**	**E**	**E**	**T**	**S**	**I**	**Q**	**A**	**E**	**T**	**L**	**K**	**K**	**F**	**F**	**D**	**–**	**S**	**K**	**Q**	**N**	**N**	**K**	**S**	**E**	**I**	**G**

**G37**	**3,637**	**ATA**	**---**	**GGT**	**GAT**	**AGT**	**ACA**	**TTT**	**ACC**	**AAG**	**ATG**	**GAT**	**GGT**	**AAA**	**CTA**	**ACT**	**GGC**	**GTA**	**GTA**	**TCT**	**ACT**	**CCC**	**CTT**	**GTT**	**AAC**	**CTT**	**ATC**	**AAT**	**GGC**	**CAG**	**GGA**
M30		ATA	---	GGT	GAT	AGT	ACA	TTT	ACC	AAG	ATG	GAT	GGT	AAA	CTA	ACT	GGC	GTA	GTA	TCT	ACT	CCC	CTT	GTT	AAC	CTT	ATC	AAT	GGC	CAG	GGA
M2282		ATA	---	GGT	GAT	AGT	ACA	TTT	ACC	AAG	ATG	GAT	GGT	AAA	CTA	ACT	GGC	GTA	GTA	TCT	ACT	CCC	CTT	GTT	AAC	CTT	ATC	AAT	GGC	CAG	GGA
M2288		ATA	---	GGT	GAT	AGT	ACA	TTT	ACC	AAG	ATG	GAT	GGT	AAA	CTA	ACT	GGC	GTA	GTA	TCT	ACT	CCT	CTT	GTT	AAC	CTT	ATC	AAT	GGT	CAG	GGA
M2300		ATA	GGA	GGT	GAT	AGT	ACA	TTT	ACC	AAG	ATG	GAT	GGT	AAA	CTA	ACT	GGC	GTA	GTA	TCT	ACT	CCC	CTT	GTT	AAC	CTT	ATC	AAT	GGC	CAG	GGA
M2321		ATA	---	GGT	GAT	AGT	ACA	TTT	ACC	AAG	ATG	GAT	GGT	AAA	CTA	ACT	GGC	GTA	GTA	TCT	ACT	CCT	CTT	GTT	AAC	CTT	ATC	AAT	GGT	CAG	GGA
M2341		ATA	---	GGT	GAT	AGT	ACA	TTT	ACC	AAG	ATG	GAT	GGT	AAA	CTA	ACT	GGA	GTA	GTA	TCT	ACT	CCT	CTT	GTT	AAC	CTT	ATC	AAT	GGT	CAG	GGA
M6257		ATA	---	GGT	GAT	AGT	ACA	TTT	ACC	AAG	ATG	GAT	GGT	AAA	CTA	ACT	GGC	GTA	GTA	TCT	ACT	CCT	CTT	GTT	AAC	CTT	ATC	AAT	GGT	CAG	GGA
M6280		ATA	---	GGT	GAT	AGT	ACA	TTT	ACC	AAG	ATG	AAT	GGT	AAA	CTA	ACT	GGC	GTA	GTA	TCT	ACT	CCC	CTT	GTT	AAC	CTT	ATC	AAT	GGC	CAG	GGA
M6282		ATA	---	GGT	GAT	AGT	ACA	TTT	ACC	AAG	ATG	GAT	GGT	AAA	CTA	ACT	GGC	GTA	GTA	TCT	ACT	CCT	CTT	GTT	AAC	CTT	ATC	AAT	GGT	CAG	GGA
M6283		ATA	---	GGT	GAT	AGT	ACA	TTT	ACC	AAG	ATG	GAT	GGT	AAA	CTA	ACT	GGC	GTA	GTA	TCT	ACT	CCC	CTT	GTT	AAC	CTT	ATT	AAT	GGC	CAG	GGA
M6284		ATA	---	GGT	GAT	AGT	ACA	TTT	ACC	AAG	ATG	GAT	GGT	AAA	CTA	ACT	GGC	GTA	GTA	TCT	ACT	CCC	CTT	GTT	AAC	CTT	ATC	AAT	GGC	CAG	GGA
M6285		ATA	---	GGT	GAT	AGT	ACA	TTT	ACC	AAG	ATG	GAT	GGT	AAA	CTA	ACT	GGC	GTA	GTA	TCT	ACT	CCT	CTT	GTT	AAC	CTT	ATC	AAT	GGT	CAG	GGA
6286		ATA	---	GGT	GAT	AGT	ACA	TTT	ACC	AAG	ATG	GAT	GGT	AAA	CTA	ACT	GGC	GTA	GTA	TCT	ACT	CCC	CTT	GTT	AAC	CTT	ATC	AAT	GGC	CAG	GGA
M6320		ATA	---	GGT	GAT	AGT	ACA	TTT	ACC	AAG	ATG	GAT	GGT	AAA	CTA	ACT	GGC	GTA	GTA	TCT	ACT	CCC	CTT	GTT	AAC	CTT	ATC	AAT	GGC	CAG	GGA
64.0		ATA	---	GGT	GAT	AGT	ACA	TTT	ACC	AAG	ATG	GAT	GGT	AAA	CTA	ACT	GGC	GTA	GTA	TCT	ACT	CCC	CTT	GTT	AAC	CTT	ATC	AAT	GGC	CAG	GGA
64.1		ATA	---	GGT	GAT	AGT	ACA	TTT	ACC	AAG	ATG	GAT	GGT	AAA	CTA	ACT	GGC	GTA	GTA	TCT	ACT	CCC	CTT	GTT	AAC	CTT	ATC	AAT	GGC	CAG	GGA
199.0		ATA	---	GGT	GAT	AGT	ACA	TTT	ACC	AAG	ATG	GAT	GGT	AAA	CTA	ACT	GGC	GTA	GTA	TCT	ACT	CCC	CTT	GTT	AAC	CTT	ATC	AAT	GGC	CAG	GGA
199.1		ATA	---	GGT	GAT	AGT	ACA	TTT	ACC	AAG	ATG	GAT	GGT	AAA	CTA	ACT	GGC	GTA	GTA	TCT	ACT	CCC	CTT	GTT	AAC	CTT	ATC	AAT	GGC	CAG	GGA
MEGA10366		ATA	---	GGT	GAT	AGT	ACA	TTT	ACC	AAG	ATG	GAT	GGT	AAA	CTA	ACT	GGC	GTA	GTA	TCT	ACT	CCC	CTT	GTT	AAC	CTT	ATT	AAT	GGC	CAG	GGA
MEGA10378		ATA	---	GGT	GAT	AGT	ACA	TTT	ACC	AAG	ATG	GAT	GGT	AAA	CTA	ACT	GGC	GTA	GTA	TCT	ACT	CCC	CTT	GTT	AAC	CTT	ATC	AAT	GGC	CAG	GGA
MEGA10467		ATA	---	GGT	GAT	AGT	ACA	TTT	ACC	AAG	ATG	GAT	GGT	AAA	CTA	ACT	GGC	GTA	GTA	TCT	ACT	CCC	CTT	GTT	AAC	CTT	ATC	AAT	GGC	CAG	GGA
MEGA10477		ATA	---	GGT	GAT	AGT	ACA	TTT	ACC	AAG	ATG	GAT	GGT	AAA	CTA	ACT	GGC	GTA	GTA	TCT	ACT	CCC	CTT	GTT	AAC	CTT	ATT	AAT	GGC	CAG	GGA
Sea1		ATA	---	GGT	GAT	AGT	ACA	TTT	ACC	AAG	ATG	GAT	GGT	AAA	CTA	ACT	GGC	GTA	GTA	TCT	ACT	CCC	CTT	GTT	AAC	CTT	ATC	AAT	GGC	CAG	GGA
Sea2		ATA	---	GGT	GAT	AGT	ACA	TTT	ACC	AAG	ATG	GAT	GGT	AAA	CTA	ACT	GGC	GTA	GTA	TCT	ACT	CCT	CTT	GTT	AAC	CTT	ATC	AAT	GGT	CAG	GGA
		***		***	***	***	***	***	***	***	***	**	***	***	***	***	**	***	***	***	***	**	***	***	***	***	**	***	**	***	***
**G37**	**1,213**	**I**	**-**	**G**	**D**	**S**	**T**	**F**	**T**	**K**	**M**	**D**	**G**	**K**	**L**	**T**	**G**	**V**	**V**	**S**	**T**	**P**	**L**	**V**	**N**	**L**	**I**	**N**	**G**	**Q**	**G**
			**G**									**N**																			
**Consensus**	**1,217**	**I**	**-**	**G**	**D**	**S**	**T**	**F**	**T**	**K**	**M**	**D**	**G**	**K**	**L**	**T**	**G**	**V**	**V**	**S**	**T**	**P**	**L**	**V**	**N**	**L**	**I**	**N**	**G**	**Q**	**G**

Repetitive Structure Identified in SAPS Analysis **L V N L**
Shown in Box VI of Figure 7B (& w/ homology to sequence on pg. 48)

Conserved C-terminus (bp 3,550-4,065; aa 1,184-1,444)

**G37**	**3,724**	**GCA**	**ACT**	**AGT**	**GAT**	**AGT**	**GAT**	**ACT**	**GAA**	**AAA**	**ATT**	**AGC**	**TTT**	**AAA**	**CCT**	**GGT**	**AAC**	**CAG**	**ATT**	**GAC**	**TTT**	**AAT**	**AGG**	**TTA**	**TTC**	**ACC**	**TTA**	**CCA**	**GTA**	**ACT**	**GAA**
M30		GCA	ACT	AGT	GAT	AGT	GAT	ACT	GAA	AAA	ATT	AGC	TTT	AAA	CCT	GGT	AAC	CAG	ATT	GAC	TTT	AAT	AGG	TTA	TTC	ACC	TTA	CCA	GTA	ACT	GAA
M2282		GCA	ACT	AGT	GAT	AGT	GAT	ACT	GAA	AAA	ATT	AGC	TTT	AAA	CCT	GGT	AAC	CAG	ATT	GAC	TTT	AAT	AGG	TTA	TTC	ACC	TTA	CCA	GTA	ACT	GAA
M2288		GCA	ACT	AGT	GAT	AGT	GAT	ACT	GAA	AAA	ATT	AGC	TTT	AAA	CCT	GGT	AAC	CAG	ATT	GAC	TTT	AAT	AGG	TTA	TTT	ACC	TTA	CCA	GTA	ACT	GAA
M2300		GCA	ACT	AGT	GAT	AGT	GAT	ACT	GAA	AAA	ATT	AGC	TTT	AAA	CCT	GGT	AAC	CAG	ATT	GAC	TTT	AAT	AGG	TTA	TTC	ACC	TTA	CCA	GTA	ACT	GAA
M2321		GCA	ACT	AGT	GAT	AGT	GAT	ACT	GAA	AAA	ATT	AGC	TTT	AAA	CCT	GGT	AAC	CAG	ATT	GAC	TTT	AAT	AGG	TTA	TTT	ACC	TTA	CCA	GTA	ACT	GAA
M2341		GCA	ACT	AGT	GAT	AGT	GAT	ACT	GAA	AAA	ATT	AGC	TTT	AAA	CCT	GGT	AAC	CAG	ATT	GAC	TTT	AAT	AGG	TTA	TTC	ACC	TTA	CCA	GTA	ACT	GAA
M6257		GCA	ACT	AGT	GAT	AGT	GAT	ACT	GAA	AAA	ATT	AGC	TTT	AAA	CCT	GGT	AAC	CAG	ATT	GAC	TTT	AAT	AGG	TTA	TTT	ACC	TTA	CCA	GTA	ACT	GAA
M6280		GCA	ACT	AGT	GAT	AGT	GAT	ACT	ACT	GAC	CTT	AGC	TTT	AAA	CCT	GGT	AAC	CAG	ATT	GAC	TTT	AAC	CGG	TTA	TTC	ACC	TTA	CCA	GTA	ACT	GAA
M6282		GCA	ACT	AGT	GAT	AGT	GAT	ACT	GAA	AAA	ATT	AGC	TTT	AAA	TCT	GGT	AAC	CAG	ATT	GAC	TTT	AAT	AGG	TTA	TTT	ACC	TTA	CCA	GTA	ACT	GAA
M6283		GCA	ACT	AGT	GAT	AGT	GAT	ACT	GAA	AAA	ATT	AGC	TTT	AAA	CCT	GGT	AAC	CAG	ATT	GAC	TTT	AAT	AGG	TTA	TTT	ACC	TTA	CCA	GTA	ACT	GAA
M6284		GCA	ACT	AGT	GAT	AGT	GAT	ACT	GAA	AAA	ATT	AGC	TTT	AAA	CCT	GGT	AAC	CAG	ATT	GAC	TTT	AAT	AGG	TTA	TTC	ACC	TTA	CCA	GTA	ACT	GAA
M6285		GCA	ACT	AGT	GAT	AGT	GAT	ACT	GAA	AAA	ATT	AGC	TTT	AAA	CCT	GGT	AAC	CAG	ATT	GAC	TTT	AAT	AGG	TTA	TTT	ACC	TTA	CCA	GTA	ACT	GAA
6286		GCA	ACT	AGT	GAT	AGT	TAT	ACT	ACT	GAC	CTT	AGC	TTT	AAA	CCT	GGT	AAC	CAG	ATT	GAC	TTT	AAC	CGG	TTA	TTC	ACC	TTA	CCA	GTA	ACT	GAA
M6320		GCA	ACT	AGT	GAT	AGT	GAT	ACT	GAA	AAA	ATT	AGC	TTT	AAA	CCT	GGT	AAC	CAG	ATT	GAC	TTT	AAT	AGG	TTA	TTC	ACC	TTA	CCA	GTA	ACT	GAA
64.0		GCA	ACT	AGT	GAT	AGT	GAT	ACT	ACT	GAC	CTT	AGC	TTT	AAA	CCT	GGT	AAC	CAG	ATT	GAC	TTT	AAC	CGG	TTA	TTC	ACC	TTA	CCA	GTA	ACT	GAA
64.1		GCA	ACT	AGT	GAT	AGT	GAT	ACT	ACT	GAC	CTT	AGC	TTT	AAA	CCT	GGT	AAC	CAG	ATT	GAC	TTT	AAC	CGG	TTA	TTC	ACC	TTA	CCA	GTA	ACT	GAA
199.0		GCA	ACT	AGT	GAT	AGT	GAT	ACT	ACT	GAC	CTT	AGC	TTT	AAA	CCT	GGT	AAC	CAG	ATT	GAC	TTT	AAC	CGG	TTA	TTC	ACC	TTA	CCA	GTA	ACT	GAA
199.1		GCA	ACT	AGT	GAT	AGT	GAT	ACT	ACT	GAC	CTT	AGC	TTT	AAA	CCT	GGT	AAC	CAG	ATT	GAC	TTT	AAC	CGG	TTA	TTC	ACC	TTA	CCA	GTA	ACT	GAA
MEGA10366		GCA	ACT	AGT	GAT	AGT	CAT	ACT	ACT	GAC	CTT	AGC	TTT	AAA	CCT	GGT	AAC	CAG	ATT	GAC	TTT	AAC	CGG	TTA	TTC	ACC	TTA	CCA	GTA	ACT	GAA
MEGA10378		GCA	ACT	AGT	GAT	AGT	GAT	ACT	GAA	AAA	ATT	AGC	TTT	AAA	CCT	GGT	AAC	CAG	ATT	GAC	TTT	AAT	AGG	TTA	TTC	ACC	TTA	CCA	GTA	ACT	GAA
MEGA10467		GCA	ACT	AGT	GAT	AGT	GAT	ACT	GAA	AAA	ATT	AGC	TTT	AAA	CCT	GGT	AAC	CAG	ATT	GAC	TTT	AAT	AGG	TTA	TTC	ACC	TTA	CCA	GTA	ACT	GAA
MEGA10477		GCA	ACT	AGT	GAT	AGT	GAT	ACT	GAA	AAA	ATT	AGC	TTT	AAA	CCT	GGT	AAC	CAG	ATT	GAC	TTT	AAT	AGG	TTA	TTT	ACC	TTA	CCA	GTA	ACT	GAA
Sea1		GCA	ACT	AGT	GAT	AGT	GAT	ACT	ACT	GAC	CTT	AGC	TTT	AAA	CCT	GGT	AAC	CAG	ATT	GAC	TTT	AAC	CGG	TTA	TTC	ACC	TTA	CCA	GTA	ACT	GAA
Sea2		GCA	ACT	AGT	GAT	AGT	GAT	ACT	GAA	AAA	ATT	AGC	TTT	AAA	CCT	GGT	AAC	CAG	ATT	GAC	TTT	AAT	AGG	TTA	TTT	ACC	TTA	CCA	GTA	ACT	GAA
		***	***	***	***	***	**	***		*	**	***	***	***	**	***	***	***	***	***	***	**	**	***	**	***	***	***	***	***	***
**G37**	**1,242**	**A**	**T**	**S**	**D**	**S**	**D**	**T**	**E**	**K**	**I**	**S**	**F**	**K**	**P**	**G**	**N**	**Q**	**I**	**D**	**F**	**N**	**R**	**L**	**F**	**T**	**L**	**P**	**V**	**T**	**E**
							**Y**		**T**	**H**	**L**				**S**																
							**H**																								
**Consensus**	**1,242**	**A**	**T**	**S**	**D**	**S**	**D**	**T**	**E**	**K**	**I**	**S**	**F**	**K**	**P**	**G**	**N**	**Q**	**I**	**D**	**F**	**N**	**R**	**L**	**F**	**T**	**L**	**P**	**V**	**T**	**E**

Conserved C-terminus (bp 3,550-4,065; aa 1,184-1,444)

**G37**	**3,814**	**CTA**	**TTT**	**GAT**	**CCT**	**AAC**	**ACG**	**ATG**	**TTT**	**GTC**	**TAT**	**GAC**	**CAG**	**TAT**	**GTA**	**CCA**	**CTA**	**TTG**	**GTT**	**AAC**	**TTA**	**CCT**	**AGT**	**GGC**	**TTT**	**GAT**	**CAA**	**GCT**	**TCA**	**ATC**	**CGC**
M30		CTA	TTT	GAT	CCT	AAC	ACG	ATG	TTT	GTC	TAT	GAC	CAG	TAT	GTA	CCA	CTA	TTG	GTT	AAC	TTA	CCT	AGT	GGC	TTT	GAT	CAA	GCT	TCA	ATC	CGC
M2282		CTA	TTT	GAT	CCT	AAC	ACG	ATG	TTT	GTC	TAT	GAC	CAG	TAT	GTA	CCA	CTA	TTG	GTT	AAC	TTA	CCT	AGT	GGC	TTT	GAT	CAA	GCT	TCA	ATC	CGC
M2288		CTA	TTT	GAT	CCT	AAC	ACG	ATG	CTT	GTC	CAT	GAC	CAG	TAT	GTA	CCA	CTA	TTG	GTT	AAC	TTA	CCT	AGT	GGC	TTT	GAT	CAA	GCT	TCA	ATC	CGC
M2300		CTA	TTT	GAT	CCT	AAC	ACG	ATG	TTT	GTC	TAT	GAC	CAG	TAT	GTA	CCA	CTA	TTG	GTT	AAC	TTA	CCT	AGT	GGC	TTT	GAT	CAA	GCT	TCA	ATC	CGC
M2321		CTA	TTT	GAT	CCT	AAC	ACG	ATG	CTT	GTC	TAT	GAC	CAG	TAT	GTA	CCA	CTA	TTG	GTT	AAC	TTA	CCT	AGT	GGC	TTT	GAT	CAA	GCT	TCA	ATC	CGC
M2341		CTA	TTT	GAT	CCT	AAC	ACG	ATG	TTT	GTC	TAT	GAC	CAG	TAT	GTA	CCA	CTA	TTG	GTT	AAC	TTA	CCT	AGT	GGC	TTT	GAT	CAA	GCT	TCA	ATC	CGC
M6257		CTA	TTT	GAT	CCT	AAC	ACG	ATG	CTT	GTC	TAT	GAC	CAG	TAT	GTA	CCA	CTA	TTG	GTT	AAC	TTA	CCT	AGT	GGC	TTT	GAT	CAA	GCT	TCA	ATC	CGC
M6280		CTA	TTT	GAT	CCT	AAC	ACG	ATG	TTT	GTC	TAT	GAC	CAG	TAT	GTA	CCA	CTA	TTG	GTT	AAC	TTA	CCT	AGT	GGC	TTT	GAT	CAA	GCT	TCA	ATC	CGC
M6282		CTA	TTT	GAT	CCT	AAC	ACG	ATG	TTT	GTC	TAT	AAC	CAG	TAT	GTA	CCA	CTA	TTG	GTT	AAC	TTA	CCT	AGT	GAC	TTT	GAT	CAA	GCT	TCA	ATC	CGC
M6283		CTA	TTT	GAT	CCT	AAC	ACG	ATG	CTT	GTC	TAT	GAC	CAG	TAT	GTA	CCA	CTA	TTG	GTT	AAC	TTA	CCT	AGT	GGC	TTT	GAT	CAA	GCT	TCA	ATC	CGC
M6284		CTA	TTT	GAT	CCT	AAC	ACG	ATG	TTT	GTC	TAT	GAC	CAG	TAT	GTA	CCA	CTA	TTG	GTT	AAC	TTA	CCT	AGT	GGC	TTT	GAT	CAA	GCT	TCA	ATC	CGC
M6285		CTA	TTT	GAT	CCT	AAC	ACG	ATG	CTT	GTC	TAT	GAC	CAG	TAT	GTA	CCA	CTA	TTG	GTT	AAC	TTA	CCT	AGT	GGC	TTT	GAT	CAA	GCT	TTA	ATC	CGC
6286		CTA	TTT	GAT	CCT	AAC	ACG	ATG	TTT	GTC	TAT	GAT	CAG	TAT	GTA	CCA	CTA	TTG	GTT	AAC	TTA	CCT	AGT	GGC	TTT	GAT	CAA	GCT	TCA	ATC	CGC
M6320		CTA	TTT	GAT	CCT	AAC	ACG	ATG	TTT	GTC	TAT	GAC	CAG	TAT	GTA	CCA	CTA	TTG	GTT	AAC	TTA	CCT	AGT	GGC	TTT	GAT	CAA	GCT	TCA	ATC	CGC
64.0		CTA	TTT	GAT	CCT	AAC	ACG	ATG	TTT	GTC	TAT	GAC	CAG	TAT	GTA	CCA	CTA	TTG	GTT	AAC	TTA	CCT	AGT	GGC	TTT	GAT	CAA	GCT	TCA	ATC	CGC
64.1		CTA	TTT	GAT	CCT	AAC	ACG	ATG	TTT	GTC	TAT	GAC	CAG	TAT	GTA	CCA	CTA	TTG	GTT	AAC	TTA	CCT	AGT	GGC	TTT	GAT	CAA	GCT	TCA	ATC	CGC
199.0		CTA	TTT	GAT	CCT	AAC	ACG	ATG	TTT	GTC	TAT	GAC	CAG	TAT	GTA	CCA	CTA	TTG	GTT	AAC	TTA	CCT	AGT	GGC	TTT	GAT	CAA	GCT	TCA	ATC	CGC
199.1		CTA	TTT	GAT	CCT	AAC	ACG	ATG	TTT	GTC	TAT	GAC	CAG	TAT	GTA	CCA	CTA	TTG	GTT	AAC	TTA	CCT	AGT	GGC	TTT	GAT	CAA	GCT	TCA	ATC	CGC
MEGA10366		CTA	TTT	GAT	CCT	AAC	ACG	ATG	TTT	GTC	TAT	GAC	CAG	TAT	GTA	CCA	CTA	TTG	GTT	AAC	TTA	CCT	AGT	GGC	TTT	GAT	CAA	GCT	TCA	ATC	CGC
MEGA10378		CTA	TTT	GAT	CCT	AAC	ACG	ATG	TTT	GTC	TAT	GAC	CAG	TAT	GTA	CCA	CTA	TTG	GTT	AAC	TTA	CCT	AGT	GGC	TTT	GAT	CAA	GCT	TCA	ATC	CGC
MEGA10467		CTA	TTT	GAT	CCT	AAC	ACG	ATG	TTT	GTC	TAT	GAC	CAG	TAT	GTA	CCA	CTA	TTG	GTT	AAC	TTA	CCT	AGT	GGC	TTT	GAT	CAA	GCT	TCA	ATC	CGC
MEGA10477		CTA	TTT	GAT	CCT	AAC	ACG	ATG	CTT	GTC	TAT	GAC	CAG	TAT	GTA	CCA	CTA	TTG	GTT	AAC	TTA	CCT	AGT	GGC	TTT	GAT	CAA	GCT	TCA	ATC	CGC
Sea1		CTA	TTT	GAT	CCT	AAC	ACG	ATG	TTT	GTC	TAT	GAC	CAG	TAT	GTA	CCA	CTA	TTG	GTT	AAC	TTA	CCT	AGT	GGC	TTT	GAT	CAA	GCT	TCA	ATC	CGC
Sea2		CTA	TTT	GAT	CCT	AAC	ACG	ATG	CTT	GTC	TAT	GAC	CAG	TAT	GTA	CCA	CTA	TTG	GTT	AAC	TTA	CCT	AGT	GGC	TTT	GAT	CAA	GCT	TCA	ATC	CGC
		***	***	***	***	***	***	***	**	***	**	*	***	***	***	***	***	***	***	***	***	***	***	* *	***	***	***	***	* *	***	***
**G37**	**1,272**	**L**	**F**	**D**	**P**	**N**	**T**	**M**	**F**	**V**	**Y**	**D**	**Q**	**Y**	**V**	**P**	**L**	**L**	**V**	**N**	**L**	**P**	**S**	**G**	**F**	**D**	**Q**	**A**	**S**	**I**	**R**
									**L**		**H**	**N**												**D**					**L**		
**Consensus**	**1,276**	**L**	**F**	**D**	**P**	**N**	**T**	**M**	**F**	**V**	**Y**	**D**	**Q**	**Y**	**V**	**P**	**L**	**L**	**V**	**N**	**L**	**P**	**S**	**G**	**F**	**D**	**Q**	**A**	**S**	**I**	**R**

Repetitive Structure Identified in SAPS Analysis **L V N L**
Shown in Box VI of Figure 7B (& w/ homology to sequence on pg. 46)

Conserved C-terminus (bp 3,550-4,065; aa 1,184-1,444)

**G37**	**3,904**	**TTA**	**AAG**	**GTA**	**ATT**	**AGT**	**TAC**	**TCA**	**GTA**	**GAA**	**AAC**	**CAA**	**ACC**	**TTA**	**GGA**	**GTT**	**AGA**	**TTA**	**GAG**	**TTC**	**AAA**	**GAT**	**CCT**	**CAA**	**ACC**	**CAA**	**CAG**	**TTT**	**ATC**	**CCG**	**GTA**
M30		TTA	AAG	GTA	ATT	AGT	TAC	TCA	GTA	GAA	AAC	CAA	ACC	TTA	GGA	GTT	AGA	TTA	GAG	TTC	AAA	GAT	CCT	CAA	ACC	CAA	CAG	TTT	ATC	CCG	GTA
M2282		TTA	AAG	GTA	ATT	AGT	TAC	TCA	GTA	GAA	AAC	CAA	ACC	TTA	GGA	GTT	AGA	TTA	GAG	TTC	AAA	GAT	CCT	CAA	ACC	CAA	CAG	TTT	ATC	CCG	GTA
M2288		TTA	AAG	GTA	ATT	AGT	TAC	TCA	GTA	GAA	AAC	CAA	ACC	TTA	GGA	GTA	AGA	TTA	GAG	TTC	AAA	GAT	CCT	GAT	ACT	AAC	CAG	TTT	ATT	CCG	GTA
M2300		TTA	AAG	GTA	ATT	AGT	TAC	TCA	GTA	GAA	AAC	CAA	ACC	TTA	GGA	GTT	AGA	TTA	GAG	TTC	AAA	GAT	CCT	CAA	ACC	CAA	CAG	TTT	ATC	CCG	GTA
M2321		TTA	AAG	GTA	ATT	AGT	TAC	TCA	GTA	GAA	AAC	CAA	ACC	TTA	GGA	GTA	AGA	TTA	GAG	TTC	AAA	GAT	CCT	GAT	ACT	AAC	CAG	TTT	ATT	CCG	GTA
M2341		TTA	AAG	GTA	ATT	AGT	TAC	TCA	GTA	GAA	AAC	CAA	ACC	TTA	GGA	GTT	AGA	TTA	GAG	TTC	AAA	GAT	CCT	CAA	ACC	CAA	CAG	TTT	ATC	CCG	GTA
M6257		TTA	AAG	GTA	ATT	AGT	TAC	TCA	GTA	GAA	AAC	CAA	ACC	TTA	GGA	GTA	AGA	TTA	GAG	TTC	AAA	GAT	CCT	GAT	ACT	AAC	CAG	TTT	ATT	CCG	GTA
M6280		TTA	AAG	GTA	ATT	AGT	TAC	TCA	GTA	GAA	AAC	CAA	ACC	TTG	GGA	GTT	AGA	TTA	GAG	TTC	AAA	GAT	CCT	CAA	ACC	CAA	CAG	TTT	ATC	CCG	GTA
M6282		TTA	AAG	GTA	ATT	AGT	TAC	TCA	GTA	GAA	AAC	CAA	ACC	TTA	GGA	GTT	AGA	TTA	GAG	TTC	AAA	GAT	CCT	CAA	ACC	CAA	CAG	TTT	ATC	CCG	GTA
M6283		TTA	AAG	GTA	ATT	AGT	TAC	TCA	GTA	GAA	AAC	CAA	ACC	TTA	GGA	GTA	AGA	TTA	GAG	TTC	AAA	GAT	CCT	GAT	ACT	AAC	CAG	TTT	ATT	CCG	GTA
M6284		TTA	AAG	GTA	ATT	AGT	TAC	TCA	GTA	GAA	AAC	CAA	ACC	TTA	GGA	GTT	AGA	TTA	GAG	TTC	AAA	GAT	CCT	CAA	ACC	CAA	CAG	TTT	ATC	CCG	GTA
M6285		TTA	AAG	GTA	ATT	AGT	TAC	TCA	GTA	GAA	AAC	CAA	ACC	TTA	GGA	GTA	AGA	TTA	GAG	TTC	AAA	GAT	TCT	AAT	ACT	AAC	CAG	TTT	ATT	CCG	GTA
6286		TTA	AAG	GTA	ATT	AGT	TAC	TCA	GTA	GAA	AAC	CAA	ACC	TTG	GGA	GTT	AGA	TTA	GAG	TTC	AAA	GAT	CCT	AAT	ACC	CAA	CAG	TTT	ATC	CCG	GTA
M6320		TTA	AAG	GTA	ATT	AGT	TAC	TCA	GTA	GAA	AAC	CAA	ACC	TTA	GGA	GTT	AGA	TTA	GAG	TTC	AAA	GAT	CCT	CAA	ACC	CAA	CAG	TTT	ATC	CCG	GTA
64.0		TTA	AAG	GTA	ATT	AGT	TAC	TCA	GTA	GAA	AAC	CAA	ACC	TTG	GGA	GTT	AGA	TTA	GAG	TTC	AAA	GAT	CCT	CAA	ACC	CAA	CAG	TTT	ATC	CCG	GTA
64.1		TTA	AAG	GTA	ATT	AGT	TAC	TCA	GTA	GAA	AAC	CAA	ACC	TTG	GGA	GTT	AGA	TTA	GAG	TTC	AAA	GAT	CCT	CAA	ACC	CAA	CAG	TTT	ATC	CCG	GTA
199.0		TTA	AAG	GTA	ATT	AGT	TAC	TCA	GTA	GAA	AAC	CAA	ACC	TTG	GGA	GTT	AGA	TTA	GAG	TTC	AAA	GAT	CCT	CGA	ACC	CAA	CAG	TTT	ATC	CCG	GTA
199.1		TTA	AAG	GTA	ATT	AGT	TAC	TCA	GTA	GAA	AAC	CAA	ACC	TTG	GGA	GTT	AGA	TTA	GAG	TTC	AAA	GAT	CCT	CGA	ACC	CAA	CAG	TTT	ATC	CCG	GTA
MEGA10366		TTA	AAG	GTA	ATT	AGT	TAC	TCA	GTA	GAA	AAC	CAA	ACC	TTG	GGA	GTT	AGA	TTA	GAG	TTC	AAA	GAT	CCT	CAA	ACC	CAA	CAG	TTT	ATC	CCG	GTA
MEGA10378		TTA	AAG	GTA	ATT	AGT	TAC	TCA	GTA	GAA	AAC	CAA	ACC	TTA	GGA	GTT	AGA	TTA	GAG	TTC	AAA	GAT	CCT	CAA	ACC	CAA	CAG	TTT	ATC	CCG	GTA
MEGA10467		TTA	AAG	GTA	ATT	AGT	TAC	TCA	GTA	GAA	AAC	CAA	ACC	TTA	GGA	GTT	AGA	TTA	GAG	TTC	AAA	GAT	CCT	CAA	ACC	CAA	CAG	TTT	ATC	CCG	GTA
MEGA10477		TTA	AAG	GTA	ATT	AGT	TAC	TCA	GTA	GAA	AAC	CAA	ACC	TTA	GGA	GTA	AGA	TTA	GAG	TTC	AAA	GAT	CCT	AAT	ACT	AAC	CAG	TTT	ATT	CCG	GTA
Sea1		TTA	AAG	GTA	ATT	AGT	TAC	TCA	GTA	GAA	AAC	CAA	ACC	TTG	GGA	GTT	AGA	TTA	GAG	TTC	AAA	GAT	CCT	CAA	ACC	CAA	CAG	TTT	ATC	CCG	GTA
Sea2		TTA	AAG	GTA	ATT	AGT	TAC	TCA	GTA	GAA	AAC	CAA	ACC	TTA	GGA	GTA	AGA	TTA	GAG	TTC	AAA	GAT	CCT	GAT	ACT	AAC	CAG	TTT	ATT	CCG	GTA
		***	***	***	***	***	***	***	***	***	***	***	***	**	***	**	***	***	***	***	***	***	**		**	*	***	***	**	***	***
**G37**	**1,302**	**L**	**K**	**V**	**I**	**S**	**Y**	**S**	**V**	**E**	**N**	**Q**	**T**	**L**	**G**	**V**	**R**	**L**	**E**	**F**	**K**	**D**	**P**	**Q**	**T**	**Q**	**Q**	**F**	**I**	**P**	**V**
																							**S**	**D**		**N**					
																								**N**							
																								**R**							

**Consensus**	**1,306**	**L**	**K**	**V**	**I**	**S**	**Y**	**S**	**V**	**E**	**N**	**Q**	**T**	**L**	**G**	**V**	**R**	**L**	**E**	**F**	**K**	**D**	**P**	**Q**	**T**	**Q**	**Q**	**F**	**I**	**P**	**V**

Conserved C-terminus (bp 3,550-4,065; aa 1,184-1,444)

```
G37        3,994  CTA AAT GCA TCA AGT ACA GGT CCC CAA ACT GTC TTT CAA CCC TTT AAC CAG TGG GCA GAC TAT GTC TTA CCT TTG ATT GTA ACT GTT CCT
M30               CTA AAT GCA TCA AGT ACA GGT CCC CAA ACT GTC TTT CAA CCC TTT AAC CAG TGG GCA GAC TAT GTC TTA CCT TTG ATT GTA ACT GTT CCT
M2282             CTA AAT GCA TCA AGT ACA GGT CCC CAA ACT GTC TTT CAA CCC TTT AAC CAG TGG GCA GAC TAT GTC TTA CCT TTG ATT GTA ACT GTT CCT
M2288             CTA AAT GCA TCA AGT ACT GGT CCC CAA ACT GTC TTT CAA CCC TTT AAT CAG TGG GCA GAC TAT GTC TTA CCT TTG ATT GTA ACT GTT CCT
M2300             CTA AAT GCA TCA AGT ACA GGT CCC CAA ACT GTC TTT CAA CCC TTT AAC CAG TGG GCA GAC TAT GTC TTA CCT TTG ATT GTA ACT GTT CCT
M2321             CTA AAT GCA TCA AGT ACT GGT CCC CAA ACT GTC TTT CAA CCC TTT AAT CAG TGG GCA GAC TAT GTC TTA CCT TTG ATT GTA ACT GTT CCT
M2341             CTA AAT GCA TCA AGT ACA GGT CCC CAA ACT GTC TTT CAA CCC TTT AAC CAG TGG GCA GAC TAT GTC TTA CCT TTG ATT GTA ACT GTT CCT
M6257             CTA AAT GCA TCA AGT ACT GGT CCC CAA ACT GTC TTT CAA CCC TTT AAT CAG TGG GCA GAC TAT GTC TTA CCT TTG ATT GTA ACT GTT CCT
M6280             CTA AAT GCA TCA AGT ACA GGT CCC CAA ACT GTC TTT CAA CCC TTT AAC CAG TGG GCA GAC TAT GTC TTA CCT TTG ATT GTA ACT GTT CCT
M6282             CTA AAT GCA TCA AGT ACA GGT CCC CAA ACT GTC TTT CAA CCC TTT AAC CAG TGG GCA GAC TAT GTC TTA CCT TTG ATT GTA ACT GTT CCT
M6283             CTA AAT GCA TCA AGT ACT GGT CCC CAA ACT GTC TTT CAA CCC TTT AAC CAG TGG GCA GAC TAT GTC TTA CCT TTG ATT GTA ACT GTT CCT
M6284             CTA AAT GCA TCA AGT ACA GGT CCC CAA ACT GTC TTT CAA CCC TTT AAC CAG TGG GCA GAC TAT GTC TTA CCT TTG ATT GTA ACT GTT CCT
M6285             CTA AAT GCA TCA AGT ACT GGT CCC CAA ACT GTC TTT CAA CCC TTT AAT CAG TGG GCA GAC TAT GTC TTA CCT TTG ATT GTA ACT GTT CCT
6286              CTA AAT GCA TCA AGT ACT GGT CCC CAA ACT GTC TTT CAA CCC TTT AAC CAG TGG GCA GAC TAT GTC TTA CCT TTG ATT GTA ACT GTT CCT
M6320             CTA AAT GCA TCA AGT ACA GGT CCC CAA ACT GTC TTT CAA CCC TTT AAC CAG TGG GCA GAC TAT GTC TTA CCT TTG ATT GTA ACT GTT CCT
64.0              CTA AAT GCA TCA AGT ACA GGT CCC CAA ACT GTC TTT CAA CCC TTT AAC CAG TGG GCA GAC TAT GTC TTA CCT TTG ATT GTA ACT GTT CCT
64.1              CTA AAT GCA TCA AGT ACA GGT CCC CAA ACT GTC TTT CAA CCC TTT AAC CAG TGG GCA GAC TAT GTC TTA CCT TTG ATT GTA ACT GTT CCT
199.0             CTA AAT GCA TCA AGT ACA GGT CCC CAA ACT GTC TTT CAA CCC TTT AAC CAG TGG GCA GAC TAT GTC TTA CCT TTG ATT GTA ACT GTT CCT
199.1             CTA AAT GCA TCA AGT ACA GGT CCC CAA ACT GTC TTT CAA CCC TTT AAC CAG TGG GCA GAC TAT GTC TTA CCT TTG ATT GTA ACT GTT CCT
MEGA10366         CTA AAT GCA TCA AGT ACA GGT CCC CAA ACT GTC TTT CAA CCC TTT AAC CAG TGG GCA GAC TAT GTC TTA CCT TTG ATT GTA ACT GTT CCT
MEGA10378         CTA AAT GCA TCA AGT ACA GGT CCC CAA ACT GTC TTT CAA CCC TTT AAC CAG TGG GCA GAC TAT GTC TTA CCT TTG ATT GTA ACT GTT CCT
MEGA10467         CTA AAT GCA TCA AGT ACA GGT CCC CAA ACT GTC TTT CAA CCC TTT AAC CAG TGG GCA GAC TAT GTC TTA CCT TTG ATT GTA ACT GTT CCT
MEGA10477         CTA AAT GCA TCA AGT ACT GGT CCC CAA ACT GTC TTT CAA CCC TTT AAC CAG TGG GCA GAC TAT GTC TTA CCT TTG ATT GTA ACT GTT CCT
Sea1              CTA AAT GCA TCA AGT ACA GGT CCC CAA ACT GTC TTT CAA CCC TTT AAC CAG TGG GCA GAC TAT GTC TTA CCT TTG ATT GTA ACT GTT CCT
Sea2              CTA AAT GCA TCA AGT ACT GGT CCC CAA ACT GTC TTT CAA CCC TTT AAT CAG TGG GCA GAC TAT GTC TTA CCT TTG ATT GTA ACT GTT CCT
                  *** *** *** *** *** **  *** *** *** *** *** *** *** *** *** **  *** *** *** *** *** *** *** *** *** *** *** *** *** ***
G37        1,332   L   N   A   S   S   T   G   P   Q   T   V   F   Q   P   F   N   Q   W   A   D   Y   V   L   P   L   I   V   T   V   P

Consensus  1,336   L   N   A   S   S   T   G   P   Q   T   V   F   Q   P   F   N   Q   W   A   D   Y   V   L   P   L   I   V   T   V   P
```

Conserved C-terminus (bp 3,550-4,065; aa 1,184-1,444)

**G37**	**4,084**	**ATA**	**GTA**	**GTG**	**ATT**	**ATC**	**CTT**	**AGT**	**GTT**	**ACT**	**TTG**	**GGA**	**TTA**	**ACG**	**ATT**	**GGA**	**ATT**	**CCA**	**ATG**	**CAC**	**AGA**	**AAC**	**AAA**	**AAG**	**GCA**	**TTA**	**CAA**	**GCA**	**GGG**	**TTT**	**GAT**
M30		ATA	GTA	GTG	ATT	ATC	CTT	AGT	GTT	ACT	TTG	GGA	TTA	ACG	ATT	GGA	ATT	CCA	ATG	CAC	AGA	AAC	AAA	AAG	GCA	TTA	CAA	GCA	GGG	TTT	GAT
M2282		ATA	GTA	GTG	ATT	ATC	CTT	AGT	GTT	ACT	TTG	GGA	TTA	ACG	ATT	GGA	ATT	CCA	ATG	CAC	AGA	AAC	AAA	AAG	GCA	TTA	CAA	GCA	GGG	TTT	GAT
M2288		ATA	GTA	GTG	ATT	ATC	CTT	AGT	GTT	ACT	TTG	GGA	TTA	ACG	ATT	GGA	ATT	CCA	ATG	CAC	AGA	AAC	AAA	AAG	GCA	TTA	CAA	GCA	GGG	TTT	GAT
M2300		ATA	GTA	GTG	ATT	ATC	CTT	AGT	GTT	ACT	TTG	GGA	TTA	ACG	ATT	GGA	ATT	CCA	ATG	CAC	AGA	AAC	AAA	AAG	GCA	TTA	CAA	GCA	GGG	TTT	GAT
M2321		ATA	GTA	GTG	ATT	ATC	CTT	AGT	GTT	ACT	TTG	GGA	TTA	ACG	ATT	GGA	ATT	CCA	ATG	CAC	AGA	AAC	AAA	AAG	GCA	TTA	CAA	GCA	GGG	TTT	GAT
M2341		ATA	GTA	GTG	ATT	ATC	CTT	AGT	GTT	ACT	TTG	GGA	TTA	ACG	ATT	GGA	ATT	CCA	ATG	CAC	AGA	AAC	AAA	AAG	GCA	TTA	CAA	GCA	GGG	TTT	GAT
M6257		ATA	GTT	GTC	ATT	ATC	CTT	AGT	GTT	ACT	TTG	GGA	TTA	ACG	ATT	GGA	ATC	CCA	ATG	CAC	AGA	AAC	AAA	AAG	GCA	TTA	CAA	GCA	GGG	TTT	GAT
M6280		ATA	GTA	GTC	ATT	ATC	CTT	AGT	GTT	ACT	TTG	GGA	TTA	ACG	ATT	GGA	ATT	CCA	ATG	CAC	AGA	AAC	AAA	AAG	GCA	TTA	CAA	GCA	GGG	TTT	GAT
M6282		ATA	GTT	GTC	ATT	ATC	CTT	AGT	GTT	ACT	TTG	GGA	TTA	ACG	ATT	GGA	ATT	CCA	ATG	CAC	AGA	AAC	AAA	AAG	GCA	TTA	CAA	GCA	GGG	TTT	GAT
M6283		ATA	GTT	GTC	ATT	ATC	CTT	AGT	GTT	ACT	TTG	GGA	TTA	ACG	ATT	GGA	ATT	CCA	ATG	CAC	AGA	AAC	AAA	AAG	GCA	TTA	CAA	GCA	GGG	TTT	GAT
M6284		ATA	GTA	GTG	ATT	ATC	CTT	AGT	GTT	ACT	TTG	GGA	TTA	ACG	ATT	GGA	ATT	CCA	ATG	CAC	AGA	AAC	AAA	AAG	GCA	TTA	CAA	GCA	GGG	TTT	GAT
M6285		ATA	GTA	GTG	ATT	ATC	CTT	AGT	GTT	ACT	TTG	GGA	TTA	ACG	ATT	GGA	ATT	CCA	ATG	CAC	AGA	AAC	AAA	AAG	GCA	TTA	CAA	GCA	GGG	TTT	GAT
6286		ATA	GTT	GTC	ATT	ATC	CTT	AGT	GTT	ACT	TTG	GGA	TTA	ACG	ATT	GGA	ATT	CCA	ATG	CAC	AGA	AAC	AAA	AAG	GCA	TTA	CAA	GCA	GGG	TTT	GAT
M6320		ATA	GTA	GTG	ATT	ATC	CTT	AGT	GTT	ACT	TTG	GGA	TTA	ACG	ATT	GGA	ATT	CCA	ATG	CAC	AGA	AAC	AAA	AAG	GCA	TTA	CAA	GCA	GGG	TTT	GAT
64.0		ATA	GTA	GTC	ATT	ATC	CTT	AGT	GTT	ACT	TTG	GGA	TTA	ACG	ATT	GGA	ATT	CCA	ATG	CAC	AGA	AAC	AAA	AAG	GCA	TTA	CAA	GCA	GGG	TTT	GAT
64.1		ATA	GTA	GTC	ATT	ATC	CTT	AGT	GTT	ACT	TTG	GGA	TTA	ACG	ATT	GGA	ATT	CCA	ATG	CAC	AGA	AAC	AAA	AAG	GCA	TTA	CAA	GCA	GGG	TTT	GAT
199.0		ATA	GTA	GTC	ATT	ATC	CTT	AGT	GTT	ACT	TTG	GGA	TTA	ACG	ATT	GGA	ATT	CCA	ATG	CAC	AGA	AAC	AAA	AAG	GCA	TTA	CAA	GCA	GGG	TTT	GAT
199.1		ATA	GTA	GTC	ATT	ATC	CTT	AGT	GTT	ACT	TTG	GGA	TTA	ACG	ATT	GGA	ATT	CCA	ATG	CAC	AGA	AAC	AAA	AAG	GCA	TTA	CAA	GCA	GGG	TTT	GAT
MEGA10366		ATA	GTT	GTC	ATT	ATC	CTT	AGT	GTT	ACT	TTG	GGA	TTA	ACG	ATT	GGA	ATC	CCA	ATG	CAC	AGA	AAC	AAA	AAG	GCA	TTA	CAA	GCA	GGG	TTT	GAT
MEGA10378		ATA	GTA	GTG	ATT	ATC	CTT	AGT	GTT	ACT	TTG	GGA	TTA	ACG	ATT	GGA	ATT	CCA	ATG	CAC	AGA	AAC	AAA	AAG	GCA	TTA	CAA	GCA	GGG	TTT	GAT
MEGA10467		ATA	GTA	GTG	ATT	ATC	CTT	AGT	GTT	ACT	TTG	GGA	TTA	ACG	ATT	GGA	ATC	CCA	ATG	CAT	AGA	AAC	AAA	AAG	GCA	TTA	CAA	GCA	GGG	TTT	GAT
MEGA10477		ATA	GTT	GTC	ATT	ATC	CTT	AGT	GTT	ACT	TTG	GGA	TTA	ACG	ATT	GGA	ATT	CCA	ATG	CAC	AGA	AAC	AAA	AAG	GCA	TTA	CAA	GCA	GGG	TTT	GAT
Sea1		ATA	GTA	GTC	ATT	ATC	CTT	AGT	GTT	ACT	TTG	GGA	TTA	ACG	ATT	GGA	ATT	CCA	ATG	CAC	AGA	AAC	AAA	AAG	GCA	TTA	CAA	GCA	GGG	TTT	GAT
Sea2		ATA	GTA	GTG	ATT	ATC	CTT	AGT	GTT	ACT	TTG	GGA	TTA	ACG	ATT	GGA	ATT	CCA	ATG	CAC	AGA	AAC	AAA	AAG	GCA	TTA	CAA	GCA	GGG	TTT	GAT
		***	**	**	***	***	***	***	***	***	***	***	***	***	***	***	**	***	***	**	***	***	***	***	***	***	***	***	***	***	***
**G37**	**1,362**	**I**	**V**	**V**	**I**	**I**	**L**	**S**	**V**	**T**	**L**	**G**	**L**	**T**	**I**	**G**	**I**	**P**	**M**	**H**	**R**	**N**	**K**	**K**	**A**	**L**	**Q**	**A**	**G**	**F**	**D**
**Consensus**	**1,366**	**I**	**V**	**V**	**I**	**I**	**L**	**S**	**V**	**T**	**L**	**G**	**L**	**T**	**I**	**G**	**I**	**P**	**M**	**H**	**R**	**N**	**K**	**K**	**A**	**L**	**Q**	**A**	**G**	**F**	**D**

Conserved C-terminus (bp 3,550-4,065; aa 1,184-1,444)

**G37**	**4,174**	**CTT TCT AAC AAA AAG GTT GAT GTC TTG ACC AAA GCA GTT GGT AGT GTC TTT AAA GAG ATC ATT AAC AGA ACA GGG ATC TCT AAC GCT CCT**
M30		CTT TCT AAC AAA AAG GTT GAT GTC TTG ACC AAA GCA GTT GGT AGT GTC TTT AAA GAG ATC ATT AAC AGA ACA GGG ATC TCT AAC GCT CCT
M2282		CTT TCT AAC AAA AAG GTT GAT GTC TTG ACC AAA GCA GTT GGT AGT GTC TTT AAA GAG ATC ATT AAC AGA ACA GGG ATC TCT AAC GCT CCT
M2288		CTT TCT AAC AAA AAG GTT GAT GTT TTG ACC AAA GCA GTT GGT AGT GTC TTT AAA GAG ATC ATT AAC AGA ACA GGG ATC TCT AAC GCT CCT
M2300		CTT TCT AAC AAA AAG GTT GAT GTC TTG ACC AAA GCA GTT GGT AGT GTC TTT AAA GAG ATC ATT AAC AGA ACA GGG ATC TCT AAC GCT CCT
M2321		CTT TCT AAC AAA AAG GTT GAT GTT TTG ACC AAA GCA GTT GGT AGT GTC TTT AAA GAG ATC ATT AAC AGA ACA GGG ATC TCT AAC GCT CCT
M2341		CTC TCT AAC AAG AAG GTT GAT GTC TTG ACC AAA GCC GTT GGT AGT GTC TTT AAA GAG ATC ATT AAC AGA ACA GGG ATC TCT AAC GCT CCT
M6257		CTC TCT AAC AAG AAG GTT GAT GTC TTG ACC AAA GCA GTT GGT AGT GTC TTT AAA GAG ATC ATT AAC AGA ACA GGG ATC TCT AAC GCT CCT
M6280		CTC TCT AAC AAG AAG GTT GAT GTC TTG ACC AAA GCA GTT GGT AGT GTC TTT AAA GAG ATC ATT AAC AGA ACA GGG ATC TCT AAC GCT CCT
M6282		CTT TCT AAC AAA AAG GTT GAT GTT TTG ACC AAA GCA GTT GGT AGT GTC TTT AAA GAG ATC ATT AAC AGA ACA GGG ATC TCT AAC GCT CCT
M6283		CTT TCT AAC AAA AAG GTT GAT GTT TTG ACC AAA GCA GTT GGT AGT GTC TTT AAA GAG ATC ATT AAC AGA ACA GGG ATC TCT AAC GCT CCT
M6284		CTT TCT AAC AAA AAG GTT GAT GTC TTG ACC AAA GCA GTT GGT AGT GTC TTT AAA GAG ATC ATT AAC AGA ACA GGG ATC TCT AAC GCT CCT
M6285		CTT TCT AAC AAA AAG GTT GAT GTT TTG ACC AAA GCA GTT GGT AGT GTC TTT AAA GAG ATC ATT AAC AGA ACA GGG ATC TCT AAC GCT CCT
6286		CTC TCT AAC AAG AAG GTT GAT GTC TTG ACC AAA GCA GTT GGT AGT GTC TTT AAA GAG ATC ATT AAC AGA ACA GGG ATC TCT AAC GCT CCT
M6320		CTT TCT AAC AAA AAG GTT GAT GTC TTG ACC AAA GCA GTT GGT AGT GTC TTT AAA GAG ATC ATT AAC AGA ACA GGG ATC TCT AAC GCT CCT
64.0		CTC TCT AAC AAG AAG GTT GAT GTC TTG ACC AAA GCA GTT GGT AGT GTC TTT AAA GAG ATC ATT AAC AGA ACA GGG ATC TCT AAC GCT CCT
64.1		CTC TCT AAC AAG AAG GTT GAT GTC TTG ACC AAA GCA GTT GGT AGT GTC TTT AAA GAG ATC ATT AAC AGA ACA GGG ATC TCT AAC GCT CCT
199.0		CTC TCT AAC AAG AAG GTT GAT GTC TTG ACC AAA GCA GTT GGT AGT GTC TTT AAA GAG ATC ATT AAC AGA ACA GGG ATC TCT AAC GCT CCT
199.1		CTC TCT AAC AAG AAG GTT GAT GTC TTG ACC AAA GCA GTT GGT AGT GTC TTT AAA GAG ATC ATT AAC AGA ACA GGG ATC TCT AAC GCT CCT
MEGA10366		CTC TCT AAC AAG AAG GTT GAT GTC TTG ACC AAA GCA GTT GGT AGT GTC TTT AAA GAG ATC ATT AAC AGA ACA GGG ATC TCT AAC GCT CCT
MEGA10378		CTT TCT AAC AAA AAG GTT GAT GTC TTG ACC AAA GCA GTT GGT AGT GTC TTT AAA GAG ATC ATT AAC AGA ACA GGG ATC TCT AAC GCT CCT
MEGA10467		CTC TCT AAC AAG AAG GTT GAT GTC TTG ACC AAA GCC GTT GGT AGT GTC TTT AAA GAG ATC ATT AAC AGA ACA GGG ATC TCT AAC GCT CCT
MEGA10477		CTT TCT AAC AAA AAG GTT GAT GTT TTG ACC AAA GCA GTT GGT AGT GTC TTT AAA GAG ATC ATT AAC AGA ACA GGG ATC TCT AAC GCT CCT
Sea1		CTC TCT AAC AAG AAG GTT GAT GTC TTG ACC AAA GCA GTT GGT AGT GTC TTT AAA GAG ATC ATT AAC AGA ACA GGG ATC TCT AAC GCT CCT
Sea2		CTT TCT AAC AAA AAG GTT GAT GTT TTG ACC AAA GCA GTT GGT AGT GTC TTT AAA GAG ATC ATT AAC AGA ACA GGG ATC TCT AAC GCT CCT
		** *** *** ** *** *** *** ** *** *** *** ** *** *** *** *** *** *** *** *** *** *** *** *** *** *** *** *** *** ***
**G37**	**1,392**	**L S N K K V D V L T K A V G S V F K E I I N R T G I S N A P**
**Consensus**	**1,396**	**L S N K K V D V L T K A V G S V F K E I I N R T G I S N A P**

Conserved C-terminus (bp 3,550-4,065; aa 1,184-1,444)

**G37**	**4,264**	**AAG**	**AAG**	**TTA**	**AAA**	**CAA**	**GCT**	**ACC**	**CCA**	**ACC**	**AAA**	**CCA**	**ACT**	**CCT**	**AAA**	**ACC**	**CCA**	**CCA**	**AAA**	**CCT**	**CCA**	**GTA**	**AAA**	**CAA**	**TAA**
M30		AAG	AAG	TTA	AAA	CAA	GCT	ACC	CCA	ACC	AAA	CCA	ACT	CCT	AAA	ACC	CCA	CCA	AAA	CCT	CCA	GTA	AAA	CAA	TAA
M2282		AAG	AAG	TTA	AAA	CAA	GCT	ACC	CCA	ACC	AAA	CCA	ACT	CCT	AAA	ACC	CCA	CCA	AAA	CCT	CCA	GTA	AAA	CAA	TAA
M2288		AAG	AAG	TTA	AAA	CAA	GCT	ACA	CCA	ACC	AAA	CCA	ACT	CCT	AAA	ACC	CCA	CCA	AAA	CCT	CCA	GTA	AAA	CAA	TAA
M2300		AAG	AAG	TTA	AAA	CAA	GCT	ACC	CCA	ACC	AAA	CCA	ACT	CCT	AAA	ACC	CCA	CCA	AAA	CCT	CCA	GTA	AAA	CAA	TAA
M2321		AAG	AAG	TTA	AAA	CAA	GCT	ACA	CCA	ACC	AAA	CCA	ACT	CCT	AAA	ACC	CCA	CCA	AAA	CCT	CCA	GTA	AAA	CAA	TAA
M2341		AAG	AAG	TTA	AAA	CAA	GCT	ACC	CCA	ACC	AAA	CCA	ACT	CCT	AAA	ACC	CCA	CCA	AAA	CCT	CCA	GTA	AAA	CAA	TAA
M6257		AAG	AAG	TTA	AAA	CAA	GCT	ACC	CCA	ACC	AAA	CCA	ACT	CCT	AAA	ACC	CCA	CCA	AAA	CCT	CCA	GTA	AAA	CAA	TAA
M6280		AAG	AAG	TTA	AAA	CAA	GCT	ACC	CCA	ACC	AAA	CCA	ACT	CCT	AAA	ACC	CCA	CCA	AAA	CCT	CCA	GTA	AAA	CAA	TAA
M6282		AAG	AAG	TTA	AAA	CAA	GCT	ACC	CCA	ACC	AAA	CCA	ACT	CCT	AAA	ACC	CCA	CCA	AAA	CCT	CCA	GTA	AAA	CAA	TAA
M6283		AAG	AAG	TTA	AAA	CAA	GCT	ACC	CCA	ACC	AAA	CCA	ACT	CCT	AAA	ACC	CCA	CCA	AAA	CCT	CCA	GTA	AAA	CAA	TAA
M6284		AAG	AAG	TTA	AAA	CAA	GCT	ACC	CCA	ACC	AAA	CCA	ACT	CCT	AAA	ACC	CCA	CCA	AAA	CCT	CCA	GTA	AAA	CAA	TAA
M6285		AAG	AAG	TTA	AAA	CAA	GCT	ACA	CCA	ACC	AAA	CCA	ACT	CCT	AAA	ACC	CCA	CCA	AAA	CCT	CCA	GTA	AAA	CAA	TAA
6286		AAG	AAG	TTA	AAA	CAA	GCT	ACC	CCA	ACC	AAA	CCA	ACT	CCT	AAA	ACC	CCA	CCA	AAA	CCT	CCA	GTA	AAA	CAA	TAA
M6320		AAG	AAG	TTA	AAA	CAA	GCT	ACC	CCA	ACC	AAA	CCA	ACT	CCT	AAA	ACC	CCA	CCA	AAA	CCT	CCA	GTA	AAA	CAA	TAA
64.0		AAG	AAG	TTA	AAA	CAA	GCT	ACC	CCA	ACC	AAA	CCA	ACT	CCT	AAA	ACC	CCA	CCA	AAA	CCT	CCA	GTA	AAA	CAA	TAA
64.1		AAG	AAG	TTA	AAA	CAA	GCT	ACC	CCA	ACC	AAA	CCA	ACT	CCT	AAA	ACC	CCA	CCA	AAA	CCT	CCA	GTA	AAA	CAA	TAA
199.0		AAG	AAG	TTA	AAA	CAA	GCT	ACC	CCA	ACC	AAA	CCA	ACT	CCT	AAA	ACC	CCA	CCA	AAA	CCT	CCA	GTA	AAA	CAA	TAA
199.1		AAG	AAG	TTA	AAA	CAA	GCT	ACC	CCA	ACC	AAA	CCA	ACT	CCT	AAA	ACC	CCA	CCA	AAA	CCT	CCA	GTA	AAA	CAA	TAA
MEGA10366		AAG	AAG	TTA	AAA	CAA	GCT	ACC	CCA	ACC	AAA	CCA	ACT	CCT	AAA	ACC	CCA	CCA	AAA	CCT	CCA	GTA	AAA	CAA	TAA
MEGA10378		AAG	AAG	TTA	AAA	CAA	GCT	ACC	CCA	ACC	AAA	CCA	ACT	CCT	AAA	ACC	CCA	CCA	AAA	CCT	CCA	GTA	AAA	CAA	TAA
MEGA10467		AAG	AAG	TTA	AAA	CAA	GCT	ACC	CCA	ACC	AAA	CCA	ACT	CCT	AAA	ACC	CCA	CCA	AAA	CCT	CCA	GTA	AAA	CAA	TAA
MEGA10477		AAG	AAG	TTA	AAA	CAA	GCT	ACC	CCA	ACC	AAA	CCA	ACT	CCT	AAA	ACC	CCA	CCA	AAA	CCT	CCA	GTA	AAA	CAA	TAA
Sea1		AAG	AAG	TTA	AAA	CAA	GCT	ACC	CCA	ACC	AAA	CCA	ACT	CCT	AAA	ACC	CCA	CCA	AAA	CCT	CCA	GTA	AAA	CAA	TAA
Sea2		AAG	AAG	TTA	AAA	CAA	GCT	ACA	CCA	ACC	AAA	CCA	ACT	CCT	AAA	ACC	CCA	CCA	AAA	CCT	CCA	GTA	AAA	CAA	TAA
		***	***	***	***	***	***	**	***	***	***	***	***	***	***	***	***	***	***	***	***	***	***	***	***
**G37**	**1,422**	**K**	**K**	**L**	**K**	**Q**	**A**	**T**	**P**	**T**	**K**	**P**	**T**	**P**	**K**	**T**	**P**	**P**	**K**	**P**	**P**	**V**	**K**	**Q**	*****
**Consensus**	**1,426**	**K**	**K**	**L**	**K**	**Q**	**A**	**T**	**P**	**T**	**K**	**P**	**T**	**P**	**K**	**T**	**P**	**P**	**K**	**P**	**P**	**V**	**K**	**Q**	*****

Lysine Repeat Identified in SAPS Analysis (sequence conserved among all strains)
Shown in Figure 7A & Box VII of Figure 7B

K K L K Q A T P T K P T P K T P P K P P V K Q *
