# Supplementary material for: Analysis of the Mycoplasma genitalium MgpB Adhesin to Predict Membrane Topology, Investigate Antibody Accessibility, Characterize Amino Acid Diversity, and Identify Functional and Immunogenic Epitopes
Source: PLoS One. 2015 Sep 18;10(9):e0138244. doi: 10.1371/journal.pone.0138244 (PMC4575044; doi:10.1371/journal.pone.0138244)
Supplement: S3 Fig — Internal transmembrane domains identified with the TopPred program display lower hydrophobicity scores than the single C-terminal transmembrane domain agreed upon by the other algorithms tested (S1 Table). Four of these internal transmembrane domains that overlap with the previously defined M3, M4, and M5 [34], in addition to a new domain spanning aa 780–800, are identified “putatively” with hydrophobicity scores exceeding the lower cutoff (green line); two others that overlap with M1 and M2 have slightly higher scores exceeding the upper cutoff (red line) and are predicted with “certainty”, although their hydrophobicity is still lower than that observed in the C-terminal transmembrane domain. These internal transmembrane domains were not identified by six of the eight algorithms tested; those overlapping with M1, M2 and M5 identified here by TopPred were also predicted by the TMpred program (S1 Table). (PDF) [file pone.0138244.s007.pdf]

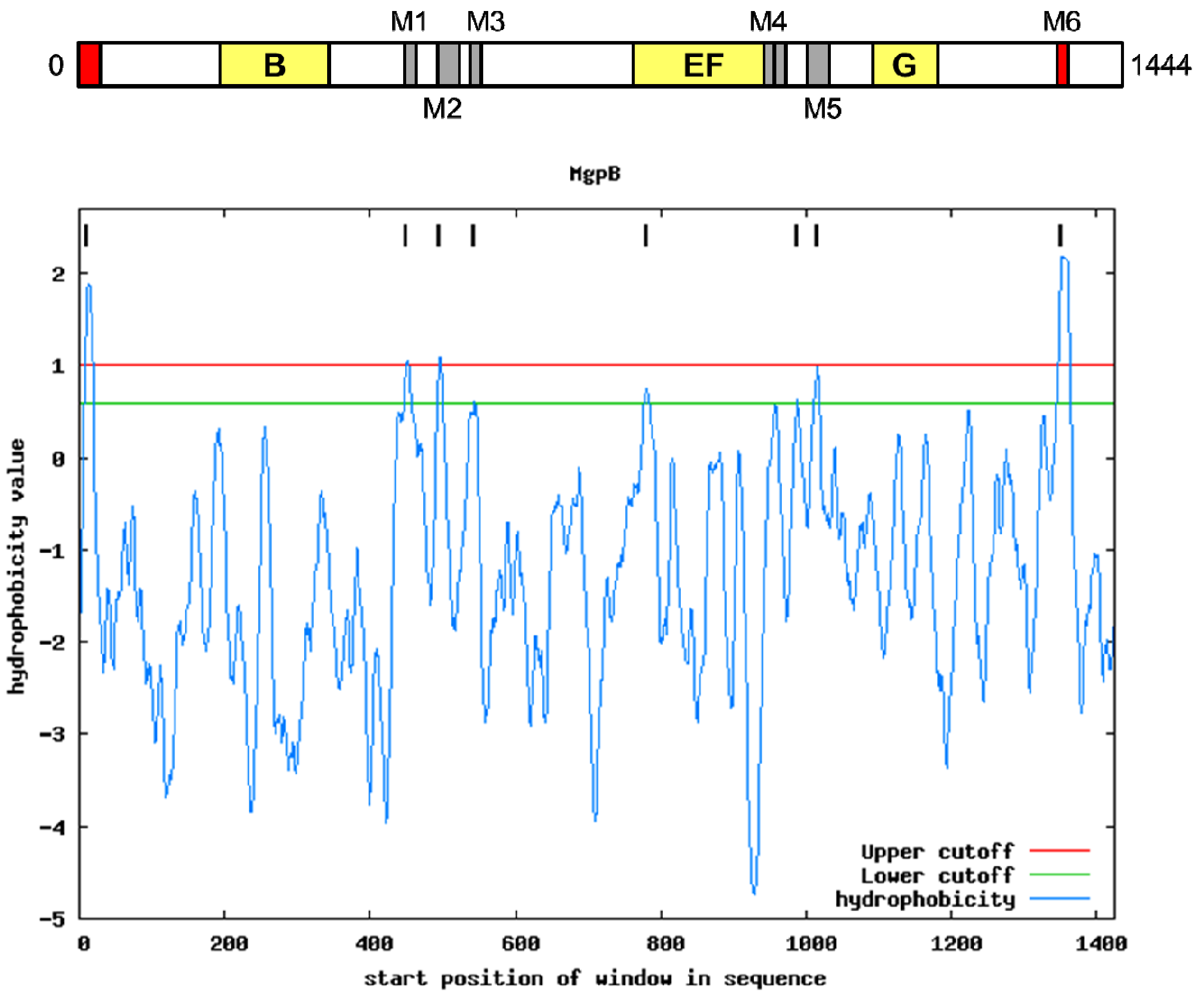

S3 Figure. Hydrophobicity of Internal Transmembrane Domains within MgpB Identified by TopPred

Internal transmembrane domains identified with the TopPred program display lower hydrophobicity scores than the single C-terminal transmembrane domain agreed upon by the other algorithms tested (S1 Table). Four of these internal transmembrane domains that overlap with the previously defined M3, M4, and M5 [34], in addition to a new domain spanning aa 780-800, are identified “putatively” with hydrophobicity scores exceeding the lower cutoff (green line); two others that overlap with M1 and M2 have slightly higher scores exceeding the upper cutoff (red line) and are predicted with “certainty”, although their hydrophobicity is still lower than that observed in the C-terminal transmembrane domain. These internal transmembrane domains were not identified by six of the eight algorithms tested; those overlapping with M1, M2 and M5 identified here by TopPred were also predicted by the TMpred program (S1 Table).
