# Supplementary material for: Analysis of the Mycoplasma genitalium MgpB Adhesin to Predict Membrane Topology, Investigate Antibody Accessibility, Characterize Amino Acid Diversity, and Identify Functional and Immunogenic Epitopes
Source: PLoS One. 2015 Sep 18;10(9):e0138244. doi: 10.1371/journal.pone.0138244 (PMC4575044; doi:10.1371/journal.pone.0138244)
Supplement: S1 References — (PDF) [file pone.0138244.s008.pdf]

S1 References. List of Additional References Used in Supplemental Figures & Tables not Cited in Main Text.

73. Hofmann K, Stoffel W. TMbase - A database of membrane spanning proteins segments. Biological Chemistry. 1993;374:166.

74. Krogh A, Larsson B, von Heijne G, Sonnhammer EL. Predicting transmembrane protein topology with a hidden Markov model: application to complete genomes. J Mol Biol. 2001 Jan 19;305(3):567-80.

75. Tusnady GE, Simon I. The HMMTOP transmembrane topology prediction server. Bioinformatics. 2001 Sep;17(9):849-50.

76. Bernsel A, Viklund H, Falk J, Lindahl E, von Heijne G, Elofsson A. Prediction of membrane-protein topology from first principles. Proc Natl Acad Sci U S A. 2008 May 20;105(20):7177-81.

77. Bernsel A, Viklund H, Hennerdal A, Elofsson A. TOPCONS: consensus prediction of membrane protein topology. Nucleic Acids Res. 2009 Jul;37(Web Server issue):W465-8.

78. Claros MG, von Heijne G. TopPred II: an improved software for membrane protein structure predictions. Comput Appl Biosci. 1994 Dec;10(6):685-6.

79. Cserzo M, Eisenhaber F, Eisenhaber B, Simon I. On filtering false positive transmembrane protein predictions. Protein Eng. 2002 Sep;15(9):745-52.

80. Hirokawa T, Boon-Chieng S, Mitaku S. SOSUI: classification and secondary structure prediction system for membrane proteins. Bioinformatics. 1998;14(4):378-9.

81. Inamine JM, Loechel S, Hu PC. Analysis of the nucleotide sequence of the P1 operon of *Mycoplasma pneumoniae*. Gene. 1988 Dec 15;73(1):175-83.

82. McGowin CL, Ma L, Jensen JS, Mancuso MM, Hamasuna R, Adegboye D, et al. Draft genome sequences of four axenic *Mycoplasma genitalium* strains isolated from Denmark, Japan, and Australia. J Bacteriol. 2012 Nov;194(21):6010-1.
