# Supplementary material for: Analysis of the Mycoplasma genitalium MgpB Adhesin to Predict Membrane Topology, Investigate Antibody Accessibility, Characterize Amino Acid Diversity, and Identify Functional and Immunogenic Epitopes
Source: PLoS One. 2015 Sep 18;10(9):e0138244. doi: 10.1371/journal.pone.0138244 (PMC4575044; doi:10.1371/journal.pone.0138244)
Supplement: S1 Table — (PDF) [file pone.0138244.s001.pdf]

**S1 Table. Signal Peptide Sequences, Transmembrane Domains, and the Resulting Membrane Topology Predictions for the MgpB Protein**

| Program | Predicted Transmembrane Domains (TM) Coordinates | | | | | | | Orientation | | Algorithm Ref. |
|---|---|---|---|---|---|---|---|---|---|---|
| | SP | M1 | M2 | M3 | M4 | M5 | M6 | Cytoplasmic | Extracellular | |
| Hydrophobic Moment Plot[1] | 14-34 | 449-469 | 500-520 | 537-557 | 953-973 | 1012-1032 | 1353-1373 | na | na | [34] |
| TMpred[2] | 13-34 | 450-469 | 498-520 | | | 1013-1035 | 1352-1377 | 35-449, 521-1012, 1378-1444 | 1-12, 470-497, 1036-1351 | [73] |
| TMHMM[3] | 12-34 | | | | | | 1353-1375 | 1-11, 1376-1444 | 35-1352 | [74] |
| HMMTOP[3] | 18-37 | | | | | | 1359-1378 | 1-17, 1379-1444 | 38-1358 | [75] |
| SCAMPI[4] | 14-34 | | | | | | 1353-1373 | 1-15, 1374-1444 | 35-1352 | [76] |
| TOPCONS[5] | 13-33 | | | | | | 1359-1379 | 1-12, 1379-1444 | 34-1358 | [77] |
| TopPred 1.10[6] | 12-32[7] | 451-471[9] | 495-515[9] | 544-564[8] | 987-1007[10] | 1015-1035[10] | 1353-1373[9] | na | na | [78] |
| DAS[9] | 17-28 | | | | | | 1354-1375 | na | na | [79] |
| SOSUI[10] | 12-34 | | | | | | 1354-1376 | na | na | [80] |

[1] Amino acid coordinates (based on the G37 type strain) of the signal peptide (SP) and TM domains predicted based upon hydrophobicity, later designated as M1 through M6 following the start of the mature protein at aa 59 [34, 39]
[2] Orientation "strongly" predicted by TMpred, which makes predictions through comparisons to TMbase, a database of naturally occurring TM proteins
[3] Predictions based upon the Hidden Markov model, which identifies temporal patterns including helical cores, helical caps, loops, and globular domains
[4] Predictions made by identifying positively charged residues and calculating the free energy contributions of amino acids
[5] Consensus prediction generated after analyzing sequence with five different algorithms
[6] Predictions based upon hydrophobicity and concentrations of positively charged residues
[7] "Certain" TM domains predicted by TopPred
[8] "Putative" TM domains predicted by TopPred program - another TM domain (aa 780-800) is not included because it was not identified in original analysis [39]
[9] Predictions based upon hydrophobicity
[10] Predictions based upon hydrophobicity and the location of amphiphilic side chains
