# Supplementary material for: Analysis of the Mycoplasma genitalium MgpB Adhesin to Predict Membrane Topology, Investigate Antibody Accessibility, Characterize Amino Acid Diversity, and Identify Functional and Immunogenic Epitopes
Source: PLoS One. 2015 Sep 18;10(9):e0138244. doi: 10.1371/journal.pone.0138244 (PMC4575044; doi:10.1371/journal.pone.0138244)
Supplement: S2 Table — (PDF) [file pone.0138244.s002.pdf]

S2 Table. Primers Used for Construction of HIS-tagged Recombinant MgpB Proteins

Recombinant Protein	Region Spanning MgpB (aa)[1]	Amplification Primers[2]	DNA Template	PCR Ingredients	Cycling Conditions
rMgpB-1	64-192	F[3]: 5'-GACGACGACAAGATCTTAACCCCTTGGACTTGGAACAATAAC-3'<br>R: 5'-GAGGAGAAGCCCGGTACTACTTCTACTGGAACTTTACCTTTG-3'	G37-C	40 uL Platinum PCR Supermix[4] with 0.2 uM primers	94°C for 4 min; 35 cycles of 30 sec at 94°C, 30 sec at 60°, and 90 sec at 72°C; 72°C for 10 min
rMgpB-B	185-352	F: 5'-GACGACGACAAGATAGGTAAAGTTCCAGTAGAAGTAGTT-3'<br>R: 5'-GAGGAGAAGCCCGGTGTATGGTTTTCACTGTAGGG-3'	G37-C	1.25 U *Pfu*[5], 1x *Pfu* buffer, 200 uM each dNTP, and 0.2 uM primers in 100 uL total[6]	94°C for 4 min; 35 cycles of 1 min at 94°C, 1 min at 65°, and 1 min at 72°C; 72°C for 10 min
rMgpB-2a	338-502	F: 5'-GACGACGACAAGATTGCGACGGTGGTTAGTTTC-3'<br>R: 5'-GAGGAGAAGCCCGGACCTGTCACGCTAGACTT-3'	pBSK-$mgpB^{S}$		
rMgpB-2b	498-749	F: 5'-GACGACGACAAGATGTCTAGCGTGACAGGTTGG-3'<br>R: 5'-GAGGAGAAGCCCGGAAAACGCTGGACTACATT-3'	pBSK-$mgpB^{S}$		
rMgpB-EF	757-970	F: 5'-GACGACGACAAGATTGATAGTTCTACCTTCGATCAGTTC-3'<br>R: 5'-GAGGAGAAGCCCGGTAAACCATAGGTATGGAGTAA-3'	G37-C		
rMgpB-3	965-1,098	F: 5'-GACGACGACAAGATACTGCTGCACACATACGGC-3'<br>R: 5'-GAGGAGAAGCCCGGTGGATTAGTATCGTAGGTG-3'	pBSK-$mgpB^{S}$		
rMgpB-G	1,092-1,209	F: 5'-GACGACGACAAGATAACTACCTATGATACCAATCCTACC-3'<br>R: 5'-GAGGAGAAGCCCGGTTCACTCTTATTGTTTTGTTTACT-3'	G37-C		
rMgpB-4a	1,187-1,368	F: 5'-GACGACGACAAGATGAAGGAGGAGACGAGCAT-3'<br>R: 5'-GAGGAGAAGCCCGGAGACAGGATAATTACCACGAT-3'	pBSK-$mgpB^{S}$		
rMgpB-4b	1,365-1,444	F: 5'-GACGACGACAAGATTATCCTGTCTGTGACGCTGG-3'<br>R: 5'-GAGGAGAAGCCCGGTTGTTTAACTGGTGGTTTTG-3'	pBSK-$mgpB^{S}$		

[1] A schematic diagram showing the location of each construct are displayed in S1A Fig. or Fig. 1
[2] Ek/LIC overhangs for insertion into pET-30 Ek/LIC vector are double underlined
[3] The rMgpB-1 forward primer contains a single base mismatch (gray, underlined) in order to mutate "TGA" to "TGG", thereby allowing for expression in *E. coli*
[4] Platinum PCR Supermix (Life Technologies, Grand Island, NY) contains 1.65 mM $MgCl_2$
[5] Native *Pfu* DNA polymerase (Stratagene, La Jolla, CA)
[6] $MgCl_2$ was added to each reaction as follows: 2.0 mM (rMgpB-4b), 2.5 mM (rMgpB-B), 3.0 mM (rMgpB-2a, -2b, -EF, and -3), 3.5 mM (rMgpB-G), and 4.0 mM (rMgpB-4a)
