# Supplementary material for: Analysis of the Mycoplasma genitalium MgpB Adhesin to Predict Membrane Topology, Investigate Antibody Accessibility, Characterize Amino Acid Diversity, and Identify Functional and Immunogenic Epitopes
Source: PLoS One. 2015 Sep 18;10(9):e0138244. doi: 10.1371/journal.pone.0138244 (PMC4575044; doi:10.1371/journal.pone.0138244)
Supplement: S3 Table — (PDF) [file pone.0138244.s003.pdf]

S3 Table. Genbank Accession Numbers for *mgpB* Sequences

Strain, Patient ID, Isolate, etc.	Genbank Accession #	*mgpB* Expression Site[1]							Reference
		Conserved N-terminus aa 1-192 (n = 34)[2]	Variable Region B aa 193-339 (n = 42)	Conserved Region 2 aa 340-764 (n = 25)	Variable Region EF aa 765-959 (n = 30)	Conserved Region 3 aa 960-1,102 (n = 25)	Variable Region G aa 1,103-1,183 (n = 33)	Conserved C-terminus aa 1,184-1,444 (n = 25)	
G37	M31431.1	X	X	X	X	X	X	X	[81]
G37-S; region B, variant 1-2	DQ248067		X						[20]
G37-S; region B, variant 1-3	DQ248068		X						[20]
G37-S; region G, variant 3-2	DQ248069						X		[20]
G37-vB; variant region B	EF458037		X						[21]
G37-vB; variant region EF	EF458038				X				[21]
G37-DK	X91075		X						[19]
G37; wk 8 B variant (primate A01220)	JX455755		X						[32]
Seattle-1	KP318805	X	X	X	X	X	X	X	this work
Seattle-2	KP318806	X	X	X	X	X	X	X	this work
M30	GU226196	X	X	X	X	X	X	X	[22]
M2282	FJ872584	X	X	X	X	X	X	X	[22]
M2288	FJ872585	X	X	X	X	X	X	X	[22]
M2300	FJ872586	X	X	X	X	X	X	X	[22]
M2321	FJ872587	X	X	X	X	X	X	X	[22]
M2341	FJ872588	X	X	X	X	X	X	X	[22]
M6257	GU226197	X	X	X	X	X	X	X	[22]
M6280	GU226198	X	X	X	X	X	X	X	[22]
M6282	GU226199	X	X	X	X	X	X	X	[22]
M6283	GU226200	X	X	X	X	X	X	X	[22]
M6284	GU226201	X	X	X	X	X	X	X	[22]
M6285	GU226202	X	X	X	X	X	X	X	[22]
6286	GU226203	X	X	X	X	X	X	X	[22]
M6320	CP003772	X	X	X	[3]	X	X	X	[82]
KOR10046[4]; strain typing	DQ248087	X							[20]
KOR10081; strain typing	DQ248088	X							[20]
KOR10081; region G, variant a	KP318807						X		this work
KOR10081; region G, variant b	KP318808						X		this work
KOR10081; region G, variant c	KP318809						X		this work
KOR10081; region G, variant d	KP318810						X		this work
KOR10081; region G, variant e	KP318811						X		this work
KOR10081; region G, variant g	KP318812						X		this work
KOR10081; region G, variant h	KP318813						X		this work
KOR10081; region G, variant i	KP318814						X		this work
KOR10089; strain typing	DQ248089	X							[20]

[1] The analysis of amino acid diversity among sequences was performed based on the location of conserved and repeat sequences; sequences used in the analysis of a particular section are noted with an "X" highlighted in yellow below each region. For instance, sequences from G37 span the full-length of MgpB, while others (ie. G37 variant 1-2) consist of partial sequences and are therefore only used for individual regions.

[2] The total number of sequences analyzed for each region

[3] M6320 was not included in the region EF alignment; the insertion of a single nucleotide shifts the reading frame resulting in premature truncation of MgpB

[4] KOR patients were enrolled in a longitudinal study of commercial sex workers in Nairobi, Kenya [7]; *mgpB* sequences came directly from cervical samples when portions of the expression site were amplified and sequenced

KOR10090; strain typing	DQ248090	X							[20]
KOR10114; strain typing	DQ248091	X							[20]
KOR10139; strain typing	DQ248092	X							[20]
KOR10139; region B; variant a	DQ248070		X						[20]
KOR10139; region B; variant b	DQ248071		X						[20]
KOR10139; region B; variant c	DQ248072		X						[20]
KOR10139; region B; variant d	DQ248073		X						[20]
KOR10139; region B; variant e	DQ248074		X						[20]
KOR10139; region B; variant f	DQ248075		X						[20]
KOR10139; region B; variant g	DQ248076		X						[20]
KOR10139; region B; variant h	DQ248077		X						[20]
KOR10139; region B; variant j	DQ248079		X						[20]
KOR10139; region B; variant k	DQ248080		X						[20]
KOR10139; region B; variant l	DQ248081		X						[20]
KOR10139; region B; variant m	DQ248082		X						[20]
KOR10139; region B; variant n	DQ248083		X						[20]
KOR10139; region B; variant o	DQ248084		X						[20]
KOR10139; region B; variant p	DQ248085		X						[20]
KOR10139; region B; variant q	DQ248086		X						[20]
KOR10163; strain typing	DQ248093	X							[20]
KOR10163; region EF, variant a	KP318815				X				this work
KOR10163; region EF, variant b	KP318816				X				this work
KOR10163; region EF, variant c	KP318817				X				this work
KOR10163; region EF, variant d	KP318818				X				this work
KOR10163; region EF, variant e	KP318819				X				this work
KOR10177; strain typing	DQ248094	X							[20]
KOR10274; strain typing	DQ248095	X							[20]
MGM10400[5]; region G, variant a	KP318820						X		this work
MGM10400; region G, variant b	KP318821						X		this work
MGM10366; conserved regions	KP318822	X		X		X		X	this work
MGM10378; conserved regions	KP318823	X		X		X		X	this work
MGM10467; conserved regions	KP318824	X		X		X		X	this work
MGM10477; conserved regions	KP318825	X		X		X		X	this work
64.0	FJ872591	X	X	X	X	X	X	X	[22]
64.0; region EF, variant a	HQ011257				X				[22]
64.0; region EF, variant b	HQ011258				X				[22]
64.0; region EF, variant c	HQ011259				X				[22]
64.1	FJ8725952	X	X	X	X	X	X	X	[22]
64.1; region EF, variant d	HQ011260				X				[22]
64.1; region EF, variant e	HQ011261				X				[22]
199.0	FJ872589	X	X	X	X	X	X	X	[22]
199.1	FJ872590	X	X	X	X	X	X	X	[22]
199.1; region G, variant b	HQ011256						X		[22]

[5] Men in the MGM study were followed longitudinally as part of a treatment trial [46]; *mgpB* sequences were amplified and sequenced directly from samples
