# Supplementary material for: Analysis of the Mycoplasma genitalium MgpB Adhesin to Predict Membrane Topology, Investigate Antibody Accessibility, Characterize Amino Acid Diversity, and Identify Functional and Immunogenic Epitopes
Source: PLoS One. 2015 Sep 18;10(9):e0138244. doi: 10.1371/journal.pone.0138244 (PMC4575044; doi:10.1371/journal.pone.0138244)
Supplement: S4 Table — The MgpB protein from M. genitalium G37, analyzed by SAPs, identifies over- or under-represented amino acids in the full-length sequence (shown to the left of the thick black line), or within overlapping fragments of ~240 aa each (shown individually to the right). In the chart above, red cells noted with a “+” or “++” identify amino acids overrepresented compared to >95% or >99% of proteins within the reference set, respectively. Similarly, gray cells highlighted with “-”or “—”indicate amino acids underrepresented compared to <5% or <1% of proteins within the reference set, respectively. Each region was compared to reference sets of B. subtilis (“B. sub”), E. coli, or a random sample of proteins in the PDB database (“all”) reference sets. (PDF) [file pone.0138244.s004.pdf]

S4 Table. Statistical Analysis of Over- and Underrepresented Amino Acid throughout MgpB

Amino Acid		MgpB (aa 1-1,444)			aa 1-240			aa 120-361			aa 241-481			aa 362-602			aa 482-722			aa 603-843			aa 723-963			aa 844-1,084			aa 964-1,204			aa 1,085-1,324			aa 1,205-1,444		
		B. sub	*E. coli*	All	*B. sub*	*E. coli*	All	*B. sub*	*E. coli*	All	*B. sub*	*E. coli*	All	*B. sub*	*E. coli*	All	*B. sub*	*E. coli*	All	*B. sub*	*E. coli*	All	*B. sub*	*E. coli*	All	*B. sub*	*E. coli*	All	*B. sub*	*E. coli*	All	*B. sub*	*E. coli*	All	*B. sub*	*E. coli*	All
Non-Polar, Hydrophobic	Ala	0	-	0	0	0	0	0	0	0	0	0	0	0	0	0	0	0	0	0	0	0	-	-	-	-	--	-	0	-	0	-	-	0	-	-	0
	Val	0	0	0	0	0	0	0	0	0	0	0	0	0	0	0	0	0	0	0	0	0	-	-	0	--	--	-	--	--	-	0	0	0	0	0	0
	Leu	0	0	0	0	0	0	0	0	0	-	-	0	0	0	0	0	0	0	0	0	0	0	0	0	0	0	0	0	0	0	0	0	0	0	0	0
	Ile	0	0	0	--	-	0	--	-	0	--	-	0	-	0	0	-	0	0	-	0	0	-	0	0	0	0	0	0	0	0	0	0	0	0	0	0
	Phe	0	0	0	0	0	0	0	0	0	0	0	0	0	0	0	0	0	0	0	0	0	0	0	0	0	0	0	0	0	0	0	0	0	0	0	0
	Trp	0	0	0	0	0	0	0	0	0	0	0	0	++	+	+	+	0	0	+	0	0	0	0	0	+	0	0	0	0	0	0	0	0	0	0	0
	Met	-	0	0	-	-	0	0	0	0	0	0	0	0	0	0	-	0	0	-	-	0	--	--	-	--	--	--	--	--	-	-	0	0	-	0	0
	Pro	+	0	0	+	0	0	0	0	0	+	0	0	++	+	0	0	0	0	++	+	0	++	++	+	++	+	0	0	0	0	0	0	0	++	++	0
Polar, Uncharged	Gly	0	0	0	0	0	0	0	0	0	0	0	0	0	0	0	0	0	0	0	0	0	0	0	0	0	0	0	0	0	0	0	0	0	0	0	0
	Ser	0	0	0	0	0	0	+	+	0	+	+	0	0	0	0	0	0	0	+	+	0	++	++	+	++	++	+	++	++	+	+	+	0	0	0	0
	Thr	+	+	0	0	0	0	0	0	0	0	0	0	0	0	0	0	0	0	0	0	0	+	+	0	+	++	+	++	++	++	++	++	++	+	+	+
	Cys	0	0	0	0	0	0	0	0	0	0	0	0	0	0	0	0	0	0	0	0	0	0	0	0	0	0	0	0	0	0	0	0	0	0	0	0
	Tyr	0	0	0	0	0	0	0	0	0	0	0	0	0	0	0	0	+	0	0	0	0	0	0	0	0	0	0	0	0	0	0	0	0	0	0	0
	Asn	++	++	+	++	++	++	0	0	0	0	0	0	+	+	0	++	++	+	++	++	++	++	++	++	++	++	+	0	+	0	+	++	+	0	0	0
	Gln	0	0	0	0	0	0	0	0	0	0	0	0	0	0	0	0	0	0	0	0	0	0	0	0	0	0	0	0	0	0	0	0	0	0	0	0
Polar, Charged	Asp	0	0	0	0	0	0	0	0	0	0	0	0	0	0	0	0	0	0	0	0	0	0	0	0	0	0	0	0	0	0	0	0	0	0	0	0
	Glu	0	0	0	0	0	0	0	0	0	0	0	0	0	0	0	0	0	0	0	0	0	-	0	0	-	-	-	-	-	-	0	0	0	0	0	0
	Lys	0	0	0	0	0	0	0	+	0	0	++	+	0	0	0	0	0	0	0	0	0	0	0	0	0	0	0	0	0	0	0	0	0	0	0	0
	Arg	0	-	0	0	0	0	0	0	0	0	-	0	0	0	0	0	0	0	0	-	0	0	0	0	-	-	-	--	--	--	-	--	-	0	-	0
	His	0	0	0	0	0	0	0	0	0	0	0	0	0	0	0	0	0	0	0	0	0	-	-	-	-	-	-	-	-	-	--	--	--	-	-	-

The MgpB protein from *M. genitalium* G37, analyzed by SAPs, identifies over- or under-represented amino acids in the full-length sequence (shown to the left of the thick black line), or within overlapping fragments of ~240 aa each (shown individually to the right). In the chart above, red cells noted with a "+" or "++" identify amino acids overrepresented compared to >95% or >99% of proteins within the reference set, respectively. Similarly, gray cells highlighted with "-" or "--" indicate amino acids underrepresented compared to <5% or <1% of proteins within the reference set, respectively. Each region was compared to reference sets of *B. subtilis* ("*B. sub*"), *E. coli*, or a random sample of proteins in the PDB database ("all") reference sets.
